# Melithaeidae of Japan (Octocorallia, Alcyonacea) re-examined with descriptions of 11 new species

**DOI:** 10.3897/zookeys.522.10294

**Published:** 2015-09-23

**Authors:** Asako K. Matsumoto, Leen P. van Ofwegen

**Affiliations:** 1 Faculty of Economics and Informatics, Educational Foundation, SHOUHEIKOU, Higashi Nippon International University, 37 Suganezawa, Kamata, Taira, Iwaki, Fukushima 970-8023, Japan; 2 Planetary Exploration Research Center (PERC), Chiba Institute of Technology (Chitech), Tsudanuma 2-17-1, Narashino, Chiba 275-0016, Japan; 3 Leen P. van Ofwegen, Department of Marine Zoology, Naturalis Biodiversity Center, Darwinweg 2, P.O. Box 9517, 2300 RA Leiden, The Netherlands

**Keywords:** *Melithaea*, taxonomy, neotype, synonymy, deep water

## Abstract

Japanese melithaeid type material is re-examined and re-described. The sclerites of the different species are depicted using Scanning Electron Microscopy. All Japanese species of the family Melithaeidae treated here belong to the genus *Melithaea* and are endemic to Japanese waters. Old museum material and newly collected specimens from Japanese waters are identified after comparison with this type material. Acabaria modesta var. abyssicola is regarded a separate species, here named *Melithaea abyssicola* (Kükenthal, 1909). In addition, 11 new species are described: *Melithaea boninensis* **sp. n.**, *Melithaea doederleini* **sp. n.**, *Melithaea isonoi* **sp. n.**, *Melithaea keramaensis* **sp. n.**, *Melithaea oyeni* **sp. n.**, *Melithaea ryukyukensis* **sp. n.**, *Melithaea sagamiensis* **sp. n.**, *Melithaea satsumaensis* **sp. n.**, *Melithaea suensoni* **sp. n.**, *Melithaea tanseii* **sp. n.**, and *Melithaea tokaraensis* **sp. n.**. *Pleurocorallium confusum* Moroff, 1902, *Pleurocoralloides formosum* Moroff, 1902, *Melitodes flabellifera* Kükenthal, 1908, and *Melitodes densa* Kükenthal, 1908 are synonymized with *Melithaea japonica* (Verrill, 1865). We have designated a neotype for *Melithaea mutsu* Minobe, 1929. A key to the Japanese melithaeids is presented.

## Introduction

Gorgoniam corals of the family Melithaeidae (Anthozoa: Octocorallia) are wide-spread and common on rocky sea bottoms of the Indo-Pacific Ocean (Bayer 1956, Ofwegen 1987, Williams 1992, Grasshoff 1999, 2000, Ofwegen et al. 2000, Reijnen et al. 2014). The family is distributed from tropical to cold waters, from the sea level to hundreds of meters depth in Japanese waters (Matsumoto et al. 2007, this paper).

Although the family is easily separated from other octocoral families, melithaeid species are quite difficult to distinguish from each other because of similarity of sclerite forms. In Japan and adjacent waters a total of 15 species and three varieties of melithaeid coral were described previously. Verrill (1865) was the first to describe a Japanese melithaeid, *Mopsella japonica* Verrill, 1865) (now *Melithaea japonica*), from Simoda (= Shimoda, Izu Peninsula) collected by Stimpson during the USA North Pacific Exploring Expedition. Later on it was put in the genus *Acabaria* (see remarks species re-description). Wright and Studer (1889) were the next authors to describe a melithaeid species, *Melitodes nodosa* Wright & Studer, 1889 (now *Melithaea nodosa*) from Hyalonema-ground (Nishi-no-Yodomi), Sagami Bay, 345 fms depth (631 m). Shortly after that Brundin (1896) described *Psilacabaria frondosa* Brundin, 1896 (now *Melithaea frondosa*) from the Hirudo Strait(= Hirado Strait, Nagasaki). The genus *Psilacabaria*, Ridley 1884 was synonymized with *Acabaria* Gray, 1859 by Kükenthal (1909). Next Moroff (1902) described two new species of octocorals from Japan, *Pleurocorallium confusum* Moroff, 1902 (synonymised with *Melithaea japonica* in this paper) and *Pleurocoralloides formosus* Moroff, 1902 (synonymised with *Melithaea japonica* in this paper); the latter in a new genus. According to Bayer and Cairns (2003) both species belong to *Acabaria*. Type specimens of both were collected in Sagami Bay. Kükenthal (1908, 1909) contributed most to the Japanese melithaeid fauna, describing no less than eight species: *Melitodes flabellifera* (Kükenthal, 1908) (synonymised with *Melithaea japonica* in this paper) from shallow water, with two varieties, Melithaea flabellifera var. reticulata and Melithaea flabellifera var. cylindrata; *Melithaea densa* Kükenthal, 1908 (synonymised with *Melithaea japonica* in this paper) from shallow water; *Melithaea arborea* Kükenthal, 1908 from shallow water in Sagami Bay; *Acabaria tenuis* Kükenthal, 1908 (now *Melithaea tenuis*) from Sagami Bay, 600 m depth and Okinose bank, 80-250 m depth; *Acabaria undulata* Kükenthal, 1908 (now *Melithaea undulata*) from Sagami Bay, 700 m depth; *Acabaria habereri* Kükenthal, 1908 (now *Melithaea habereri*) from Sagami Bay; *Acabaria modesta* Kükenthal, 1908 (now *Melithaea modesta*) from Sagami Bay, 80-250 m depth, with one variety *abyssicola*, also from Sagami Bay but from 600 m depth, and finally *Acabaria corymbosa* Kükenthal, 1908 (now *Melithaea corymbosa*) from Misaki. Nutting (1912) recorded *Mopsella dichotoma* (Linnaeus, 1758) (now *Melithaea dichotoma*), originally decribed from South Africa, from several localities around Japan. The last to describe a melithaeid species from Japan was Minobe (1929), *Melitodes mutsu* Minobe, 1929 (now *Melithaea mutsu*) from Mutsu Bay.

Unfortunately, all these species were poorly described and figured making identification of melithaeids in Japanese waters next to impossible. Therefore an attempt was made to re-describe these species and at the same time identify newly collected material for better understanding of species variation.

## Material and methods

Per specimen a small piece of the distal part of a branch was dissolved in a 4% household bleach solution to isolate sclerites. The sclerites were washed with demineralised water, dried on a hot plate, mounted on SEM stubs, and coated with Pd/Au for SEM imaging. For this, either a JEOL JSM6490LV scanning electron microscope was operated at high vacuum at 10 kV, or a a JEOL JSM6510LA scanning electron microscope with a Quick Carbon Coater SC-701C, SANYU ELECTRON was used.

Material transferred to the Naturalis Biodiversity Center (RMNH) is presented in the material section with the original AKM number between brackets.

RMNH Coel. 41916 (AKM 615; *Melithaea japonica*), RMNH Coel. 41942 (AKM 664; *Melithaea tenuis*), RMNH Coel. 41943 (AKM 980; *Melithaea tenuis*), RMNH Coel. 41936 (AKM 743; *Melithaea satsumaensis* sp. n.), RMNH Coel. 41925 (AKM 1148; *Melithaea keramaensis* sp. n.), RMNH Coel. 41908 (AKM 1175; *Melithaea corymbosa*), RMNH Coel. 41920 (AKM 1200; *Melithaea japonica*) and RMNH Coel. 41923 (AKM 1252; *Melithaea japonica*) have been used in the molecular study of Reijnen et al. Of these RMNH Coel. 41916, RMNH Coel. 41942, RMNH Coel. 41920 and RMNH Coel. 41923 had identical sequences, here now identified as *Melithaea japonica*. RMNH Coel. 41942 was the only identified Japanese species in the molecular study, as *Melithaea tenuis*.

We follow Reijnen et al. (2014) regarding generic classification, with only two valid genera in the Melithaeidae, *Melithaea* and *Asperaxis*, with the latter genus only reported from Australia.

Descriptions of old Japanese material collected by Japanese used “hiro” (Japanese fathom) as the depth unit. One Japanese fathom (hiro) is usually 1.43 m, occasionally 1.51 m, whereas, it is 1.818 m for the length unit on land. The old depth unit fathom is also converted to 1.8288 m. When it was not clear whether the collector used fathom or hiro, the converted depth has wider ranges.

### Abbreviations

AKM Asako K. Matsumoto collection, Planetary Exploration Research Center (PERC), Chiba Institute of Technology (Chitech), Japan

BIK The Biological Institute on Kuroshio, Kochi, Japan

BMNH British Museum of Natural History, London, UK

MCZ Museum of Comparative zoology Harvard University, Cambridge, USA

ME (UPSZTY) Museum of Evolution, Uppsala, Sweden

MZS Musée Zoologique de Strasbourg, 29 boulevard de la Victoire, Strasbourg, France

NBC (RMNH) Naturalis Biodiversity Center, formerly Rijksmuseum van Natuurlijke Historie, Darwinweg 2, P.O. Box 9517, 2300 RA Leiden, The Netherlands

NHMW Naturhistorisches Museum Wien, Austria

SMBL Seto Marine Biological Laboratory, Field Science Education and Research Center, Kyoto University, Shirahama-cyo 459, Nishi-muro-gun, Wakayama Prefecture, 649-2211, Japan

SMF Forschungsinstitut und Naturmuseum Senckenberg, Frankfurt, Germany

UMZC University Museum of Zoology Cambridge, Downing Street, Cambridge, CB2 3EJ, UK

UMUTZ University Museum of University of Tokyo, Hongo 7-3-1, Bunkyo-ku, 113-0033, Tokyo, Japan

ZMB Museum für Naturkunde der Humboldt-Universität, Berlin, Germany

ZMH Zoologisches Museum Hamburg, Germany

ZMUC Zoological Museum University of Copenhagen, Universitetsparken 15, DK-2100 Copenhagen, Denmark

ZSM Zoologische Staatssammlung München, Münchhausenstraße 21, 81247 Munich, Germany

### Key to Melithaea species of Japan

**Table:** 

1	Colony with abundant anastomoses	*Melithaea boninensis* sp. n. / Melithaea habereri*
–	Anastomoses may be present but never many	**2**
2	Mostly spindles in coenenchyme, few capstans and derivatives may be present	**3**
–	Capstans and derivatives abundant	**4**
3	Calyces without clubs	***Melithaea modesta***
–	Calyces with leaf clubs	***Melithaea nodosa***
4	Calyces without clubs	***Melithaea mutsu***
–	Calyces with clubs	**5**
5	Capstans and derivatives in small numbers	**6**
–	Capstans and derivatives predominant	**8**
6	Spindles wide and long, up to 0.30 mm long	**7**
–	Spindles mostly short, up to 0.10 mm long, with prominent tubercles	***Melithaea corymbosa***
7	Capstans slightly unilaterally spinose	***Melithaea undulata***
–	Capstans strongly unilaterally spinose	***Melithaea isonoi* sp. n.**
8	Double disks and clubs resembling flower buds absent	**9**
–	Double disks or clubs resembling flower buds present	**14**
9	Clubs and unilaterally spinose spheroids with very spiny head	***Melithaea frondosa***
–	Calyces with thorn clubs or leaf clubs	**10**
10	Calyces with thorn clubs	***Melithaea arborea* / *Melithaea japonica*** **
–	Calyces with leaf clubs	**11**
11	Disk spindles present	**12**
–	No disk spindles	**13**
12	Coenenchymal spindles slender, up to 0.04 mm wide	***Melithaea tenuis***
–	Coenenchymal spindles thick, up to 0.06 mm wide, almost twice as wide as in *Melithaea tenuis*	***Melithaea tanseii* sp. n.**
13	Coenenchymal spindles slender, up to 0.04 mm wide	***Melithaea sagamiensis* sp. n.**
–	Coenenchymal spindles thick, up to 0.07 mm wide	***Melithaea suensoni* sp. n.**
14	Calyx clubs up to 0.25 mm long, polyp sclerites weakly tuberculate	***Melithaea tokaraensis* sp. n.**
–	Calyx clubs only up to 0.15 mm long, polyp sclerites with tubercles or leaves	**15**
15	Coenenchymal clubs resemble flower buds	***Melithaea doederleini* sp. n.**
–	Coenenchymal clubs differently shaped	**16**
16	Unilaterally foliate spheroids present	***Melithaea satsumaensis* sp. n.**
–	No unilaterally foliate spheroids present	**17**
17	Double disks very wide, up to 0.10 mm	***Melithaea oyeni* sp. n.**
–	Double disks mostly 0.05 mm wide	**18**
18	Spindles up to 0.20 mm long, disk spindles up to 0.10 mm long	**19**
–	Spindles and disk spindles up to 0.15 mm long	***Melithaea keramaensis* sp. n.**
19	Spindles can be very wide, more than 0.05 mm wide	***Melithaea ryukyuensis* sp. n.**
–	Spindles at the most 0.05 mm wide	***Melithaea abyssicola***

* Because the material of *Melithaea habereri* is lost and the description is rather poor, no destinction could be made between *Melithaea boninensis* sp. n. and *Melithaea habereri*.

** The difference between *Melithaea arborea* and *Melithaea japonica* is unclear (see remarks of *Melithaea arborea*).

## Description

### Melithaea abyssicola

Taxon classificationAnimaliaAlcyonaceaMelithaeidae

(Kükenthal, 1909)

Figures 1 , 2 , 3 , 4 , 5 , 6 , 7 , 8

Acabaria modesta var. abyssicola : Kükenthal 1909: 68 (Sagami Bay, Japan); Kükenthal 1919: 184; 1924: 79; Aurivillius 1931: 28 (Sagami Bay, 400–500 m).
?Acabaria modesta abyssicola : Rho et al. 1980: 55 (Korea Strait).

#### Material examined.

Holotype **ZSM 20040057**, Sagami Bay, 600 m, coll. Doflein 1904/05; previously unidentified museum material: **BMNH 1921.10.26.5**, Misaki, Sagami Bay, 333 fms (609 m), coll. A.V. Insole, May 1921; **UMUTZ-CnidG-21**, Gokeba, Sagami Bay, 150–20 hiro (227–29 m), coll. K. Aoki, 18 June 1902; **UMUTZ-CnidG-28**, Kahiwajima Is., Tosa, Kochi Prefecture, coll. K. Kinoshita, June 1909; **UMUTZ-CnidG-29**, same data as UMUTZ-CnidG-28; **UMUTZ-CnidG-30**, same data as UMUTZ-CnidG-28; **UMUTZ-CnidG-33**, same data as UMUTZ-CnidG-28; **UMUTZ-CnidG-101**, St.5, 170 fms (possibly Japanese fms) (243–257 m), coll. I. Ijima, 2 April 1895; **UMUTZ-CnidG-232**, same data as UMUTZ-CnidG-28; **RMNH Coel. 41900 (AKM 430)**, Zeni-su, off Izu Islands, 33°53'N 138°43'E, *R/V Tansei-maru*, KT04-06, st. ZN-3, 267.3–288.3 m, coll. A.K. Matsumoto, 3 April 2004; **RMNH Coel. 41901 (AKM 571)**, Otsuki, Tosa, Kochi Prefecture, 32°43'N, 132°48'E, local fishermen’s boat, *Kiryo-maru*, st. 3, coral-net, 84.75–83.1 m, coll. A.K. Matsumoto, 7 October 2004; ?**BMNH 1921.10.26.24-2**, Misaki, Sagami Bay, 500–600 fms (715-1097 m), coll. A.V. Insole No. 45.

#### Description.

**Figure 1.**
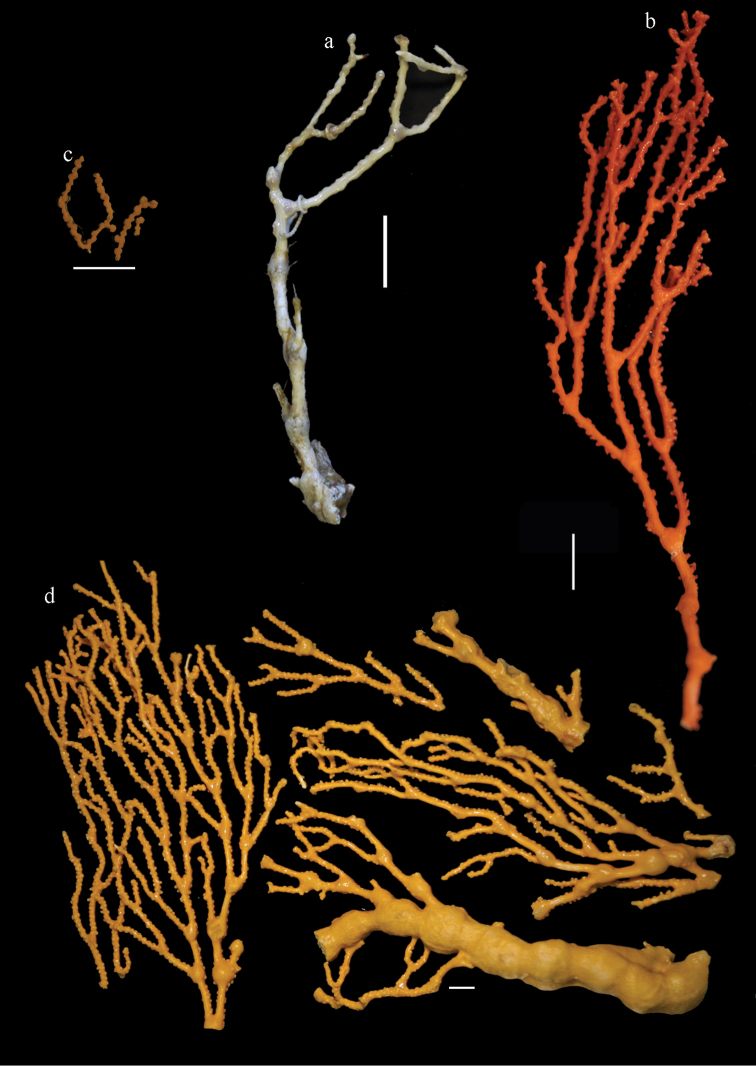
*Melithaea abyssicola*, colonies; **a** ZSM 20040057, holotype **b** AKM 571 **c** BMNH 1921.10.26.24-2 **d** UMUTZ-CnidG-28.

**Figure 2.**
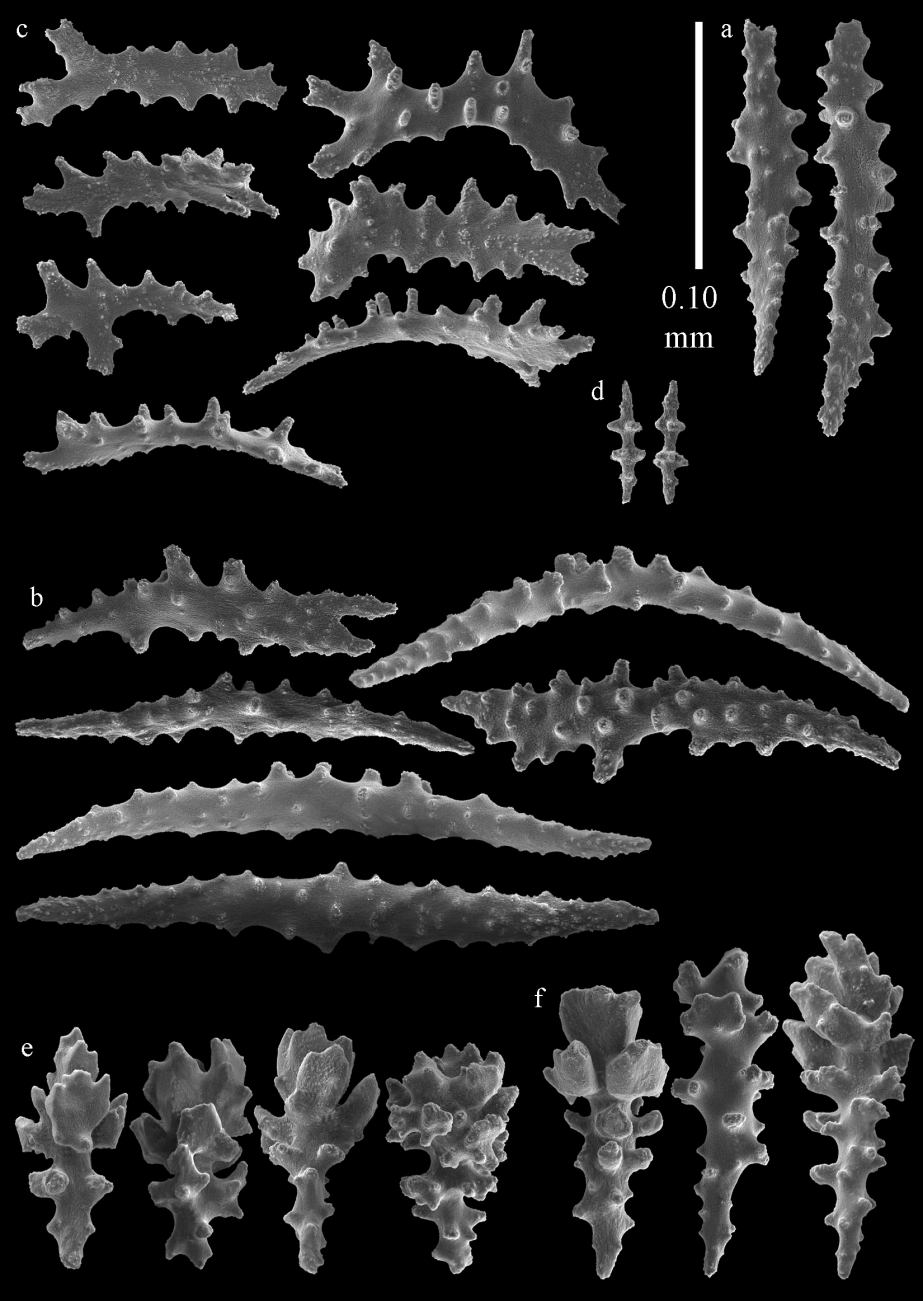
Sclerites of *Melithaea abyssicola*, ZSM 20040057, holotype; **a** point spindles **b** collaret spindles **c** tentacle sclerites **d** pharynx rods **e** clubs of coenenchyme **f** clubs of calyx.

**Figure 3.**
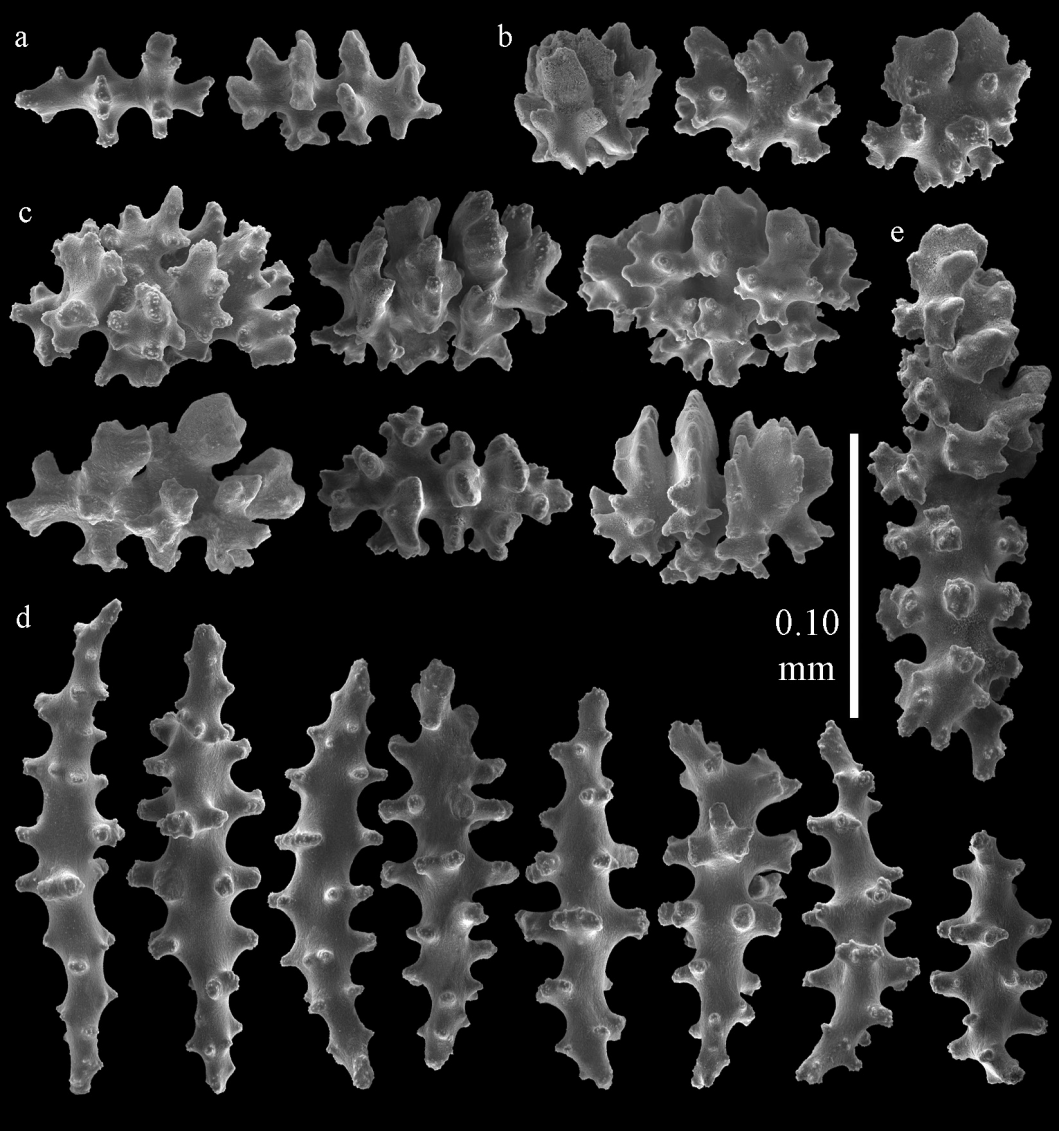
Sclerites of *Melithaea abyssicola*, ZSM 20040057, holotype; **a** capstans **b** double disks **c** disk spindles **d** spindles **e** intermediate sclerite.

Colony branched in one plane with few anastomoses (Fig. 1a). Points with slightly bent spindles up to 0.17 mm long, distal end with more developed tubercles (Fig. 2a). Collaret with bent spindles up to 0.30 mm long, middle part with more developed tubercles (Fig. 2b). Tentacles with platelets, the larger ones crescent-shaped with irregular projections (Fig. 2c). These platelets are up to 0.15 mm long. Pharynx with straight spiny rods, up to 0.05 mm long (Fig. 2d). Coenenchyme with predominantly capstans (Fig. 3a), double disks (Fig. 3b) and disk spindles (Fig. 3c), 0.05–0.10 mm long, and small clubs of similar length (Figure 2e). Spindles, 0.10–0.20 mm long, with simple tubercles, are also present (Fig. 3d); some sclerites are intermediate between clubs and spindles (Fig. 3e). The calyces with additional clubs, up to 0.14 mm long (Fig. 2f).

#### Color.

Colony white, sclerites colorless.

#### Variation.

**Figure 4.**
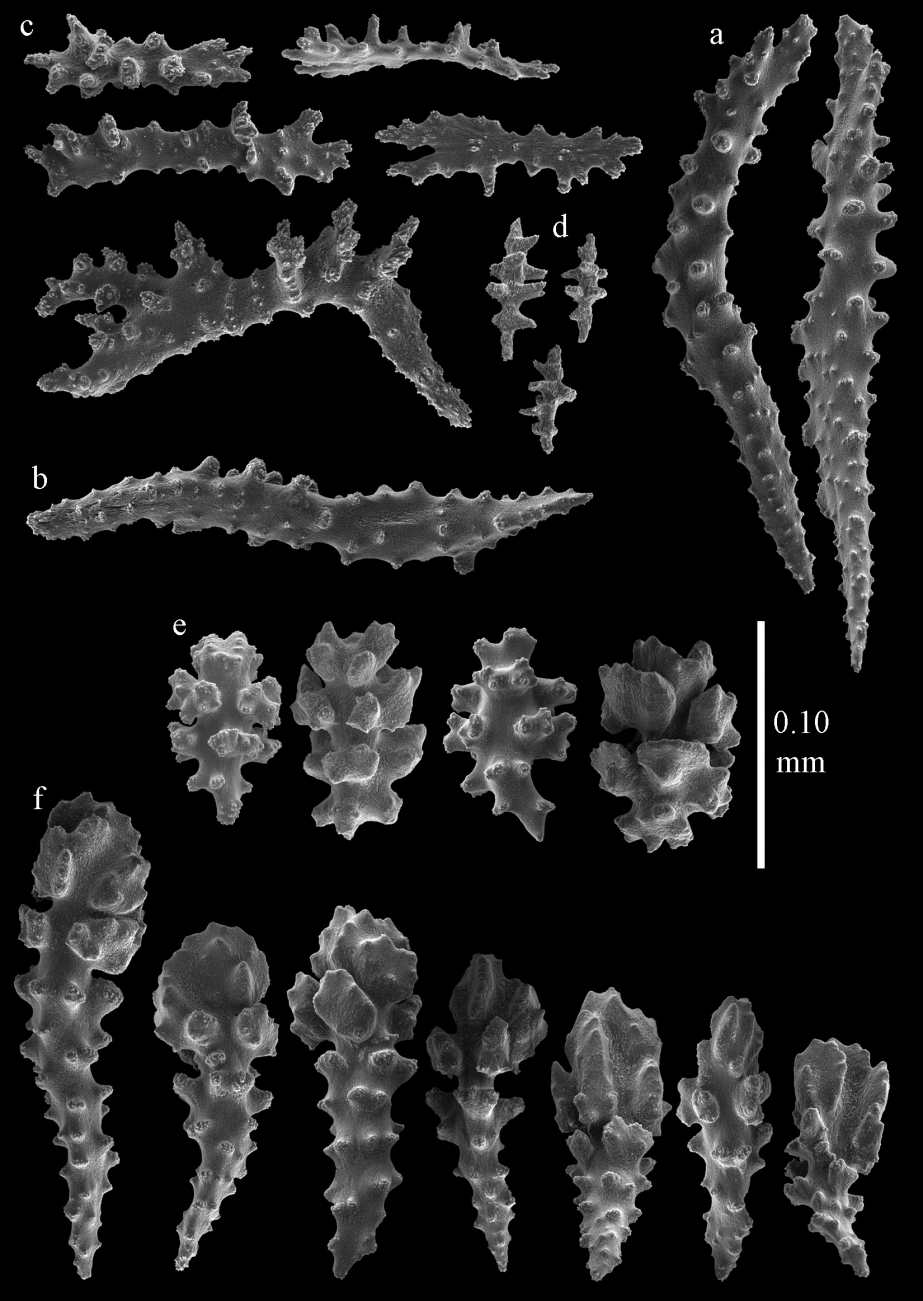
Sclerites of *Melithaea abyssicola*, RMNH Coel. 41901; **a** point spindles **b** collaret spindle **c** tentacle sclerites **d** pharynx rods **e** clubs of coenenchyme **f** clubs of calyx.

**Figure 5.**
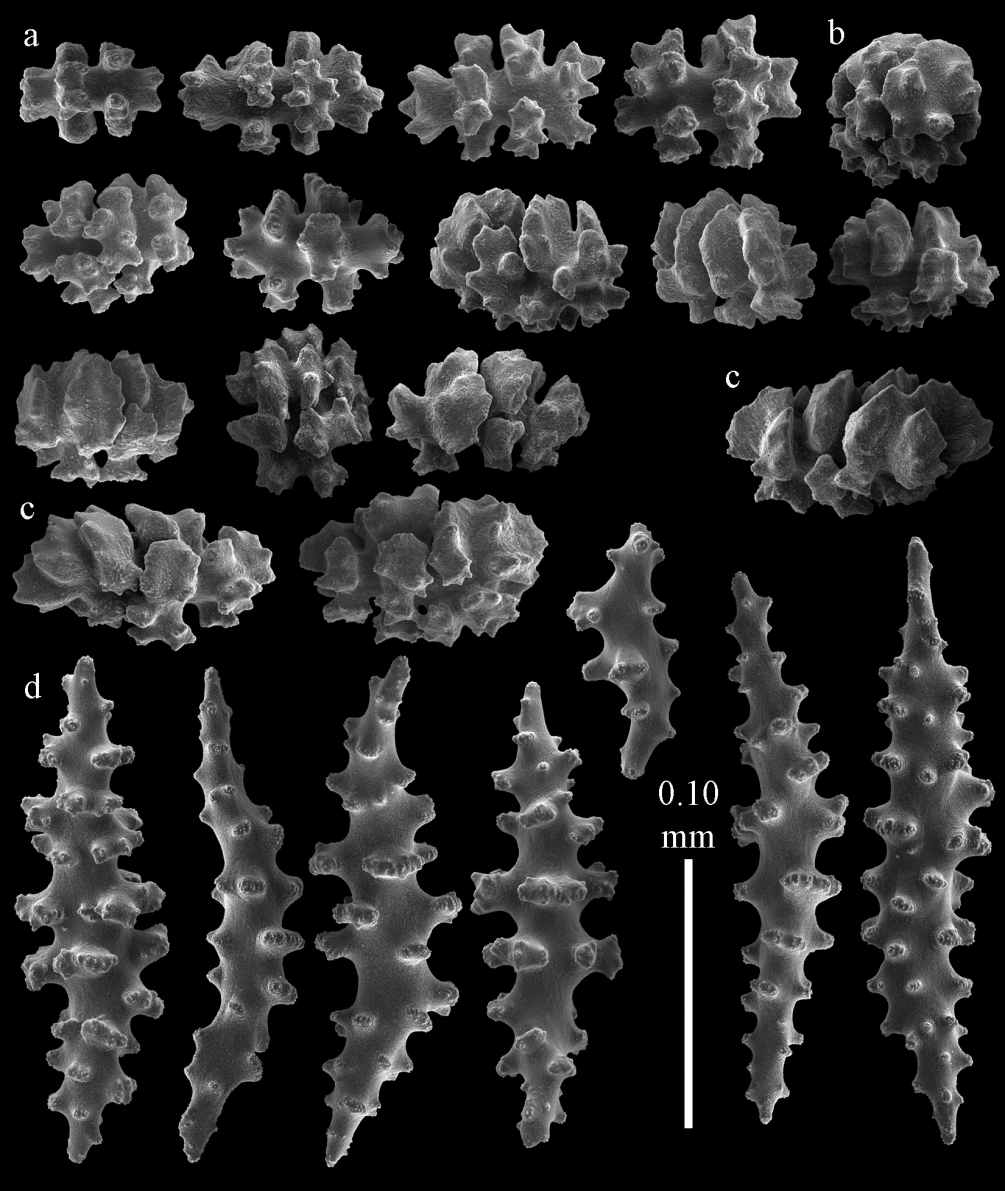
Sclerites of *Melithaea abyssicola*, RMNH Coel. 41901; **a** capstans **b** double disks **c** disk spindles **d** spindles.

RMNH Coel. 41900, UMUTZ-CnidG-28 (Fig. 1d), UMUTZ-CnidG-30, UMUTZ-CnidG-33, RMNH Coel. 41900 and BMNH 1921.10.26.24-2 are yellow with yellow sclerites; UMUTZ-CnidG-21, UMUTZ-CnidG-101 and UMUTZ-CnidG-232 are orange colonies; UMUTZ-CnidG-29 is red. RMNH Coel. 41901 is orange with red polyps (Fig. 1b), polyp sclerites pink, all others yellow. The sclerites of RMNH Coel. 41901 are similar to the holotype (Figs 4, 5) but it has somewhat longer coenenchymal spindles, up to 0.25 mm long (Fig. 5d).

#### Distribution.

*Melithaea abyssicola* occurs in Sagami Bay, off the Izu Islands, and Tosa (Kochi Prefecture)(Fig. 8).

#### Remarks.

Kükenthal (1909) probably made this a variety of *Acabaria modesta* Kükenthal, 1908 because the colonies and sclerites of these two species have the same color. However, morphologically the sclerites of these two species are completely different by *Melithaea modesta* lacking clubs, double disks and disk spindles.

The species resembles *Melithaea sagamiensis* sp. n., but differs in having much smaller double disks, up to 0.05 mm long.

**Figure 6.**
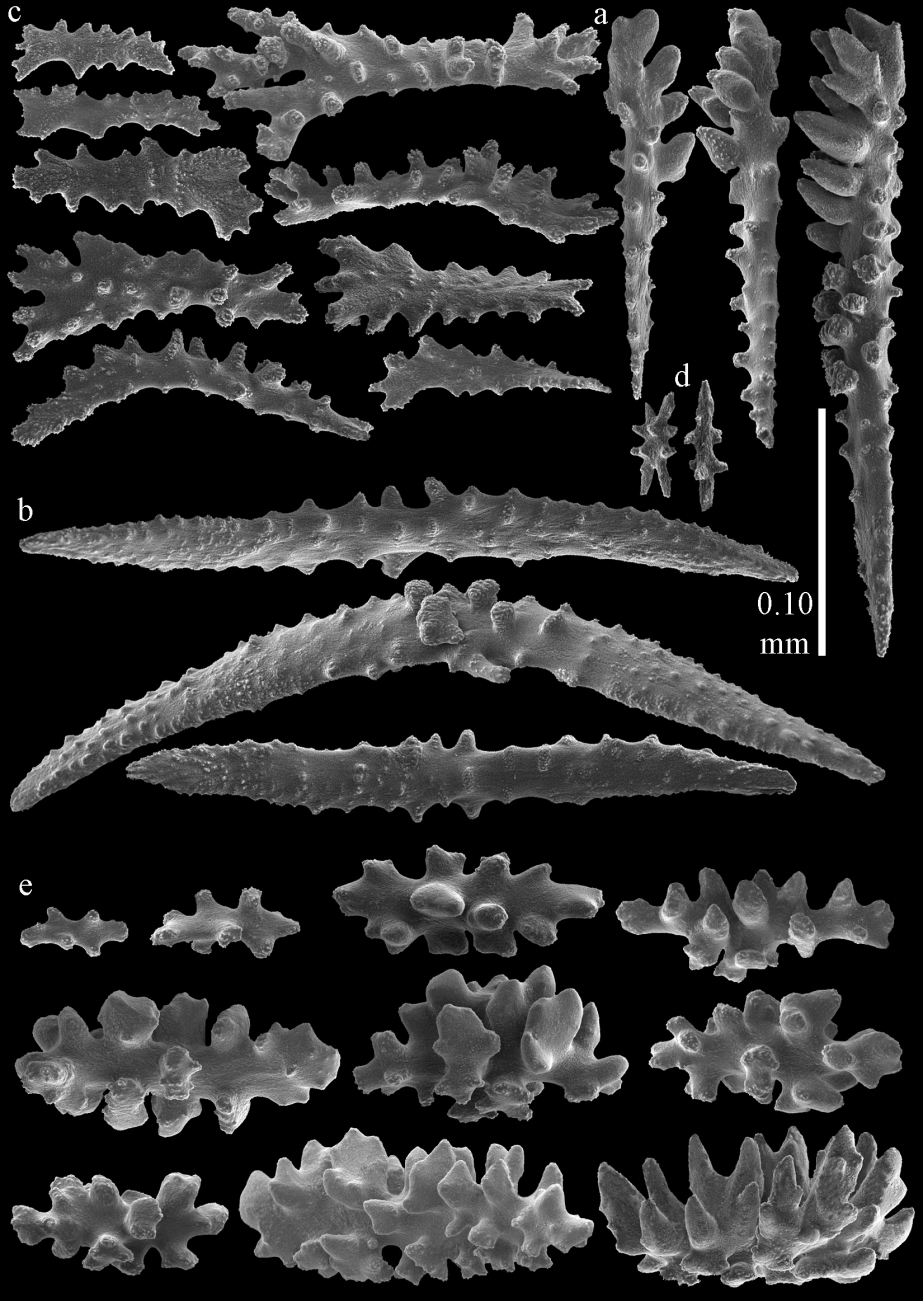
Sclerites of *Melithaea abyssicola*, BMNH 1921.10.26.24-2; **a** point spindles **b** collaret spindles **c** tentacle sclerites **d** pharynx rods **e** capstans and disk spindles of coenenchyme.

**Figure 7.**
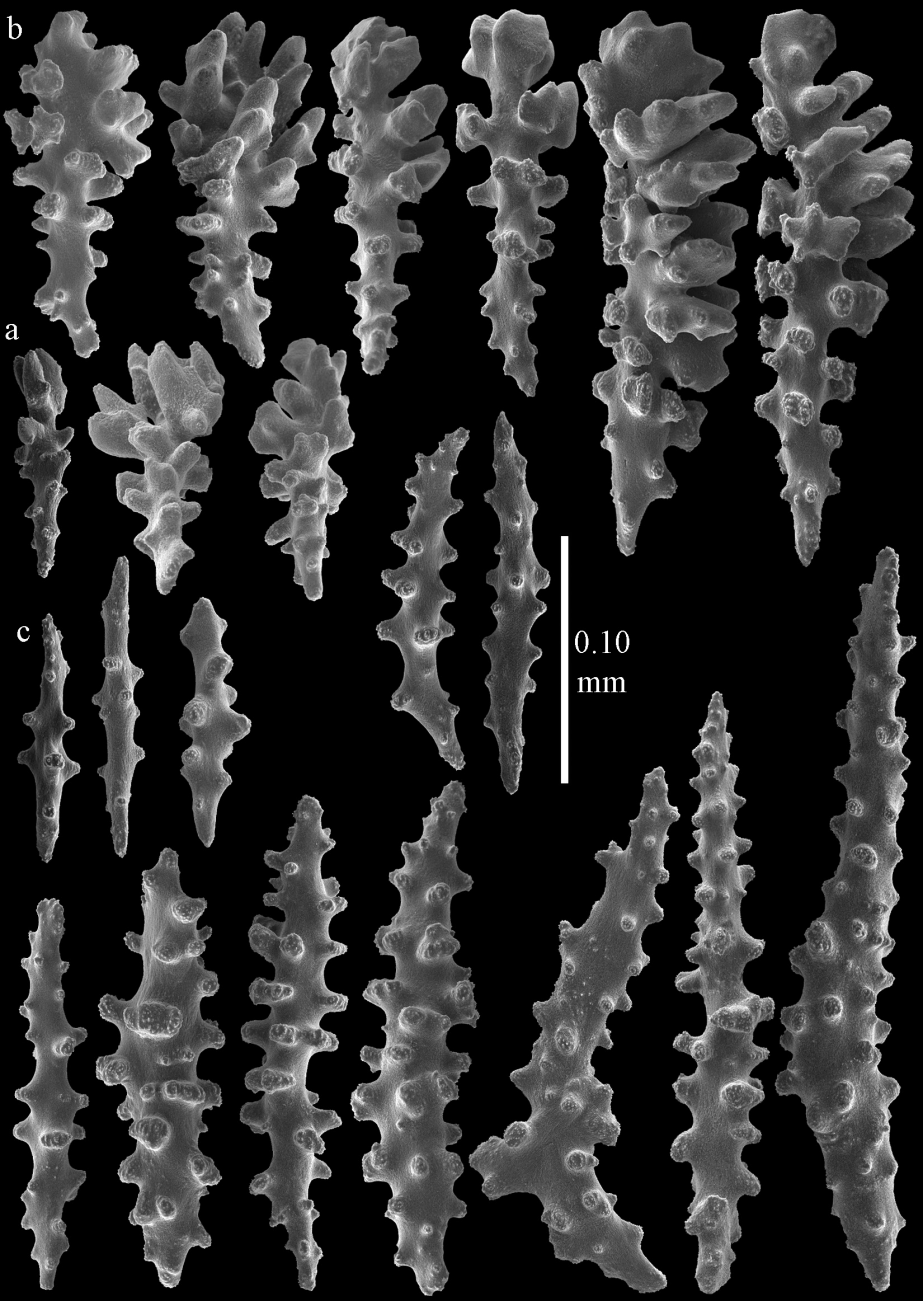
Sclerites of *Melithaea abyssicola*, BMNH 1921.10.26.24-2; **a** clubs of coenenchyme **b** clubs of calyx **c** spindles.

We have tentatively included BMNH 1921.10.26.24-2 (Fig. 1c) in *Melithaea abyssicola* as it was collected together with BMNH 1921.10.26.5 by the same collector at the same locality, only at different depths. However, the specimen has clubs and disk spindles but lacks the double disks and shows sclerite damage caused by formalin (Figs 6, 7).

**Figure 8.**
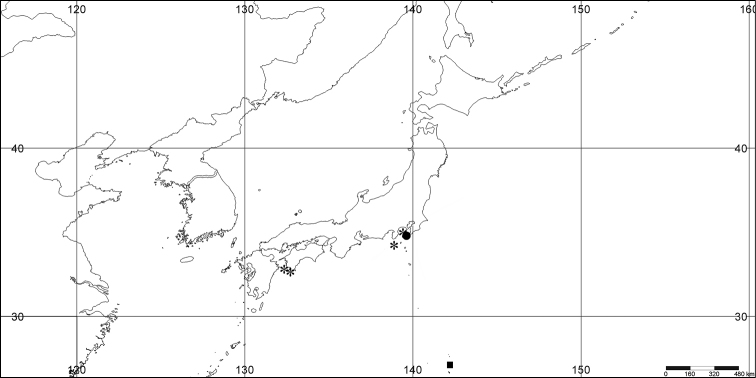
Distribution of *Melithaea abyssicola* (*), *Melithaea arborea* (●), and *Melithaea boninensis* sp. n. (■).

### Melithaea arborea

Taxon classificationAnimaliaAlcyonaceaMelithaeidae

Kükenthal, 1908

**Figure 9.**
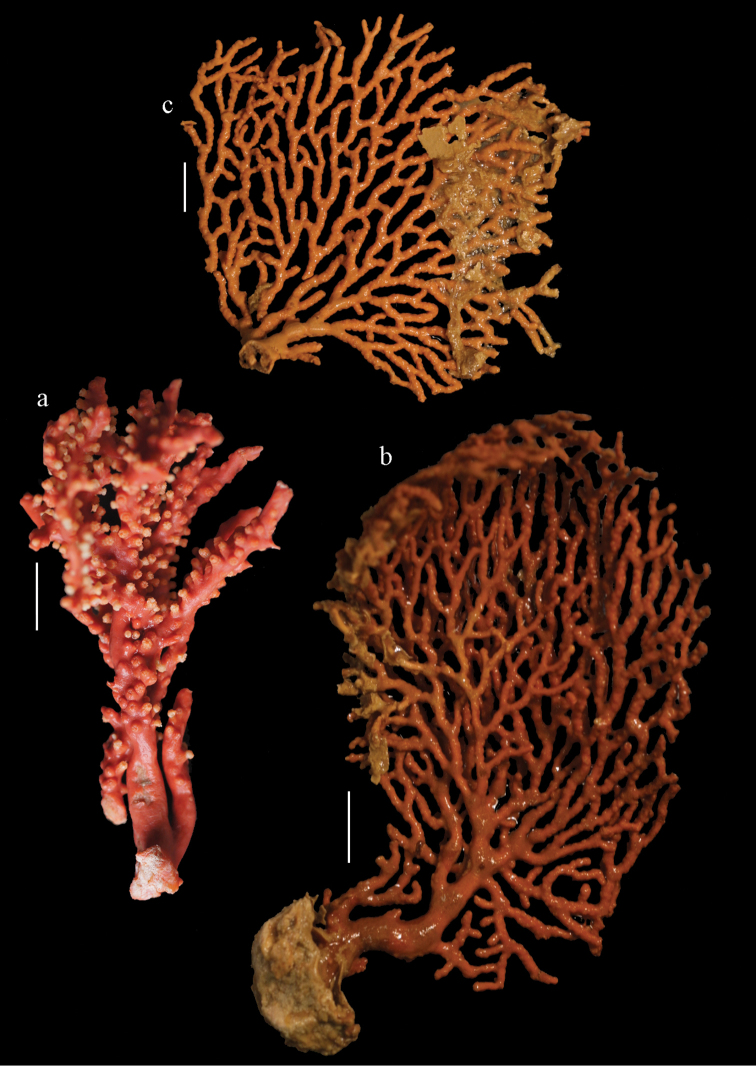
**a** *Melithaea arborea*, ZMH C3305 holotype **b** *Melithaea boninensis* sp. n., holotype UMUTZ-CnidG-205 **c** paratype UMUTZ-CnidG-255. Scale bars 1 cm.

**Figure 10.**
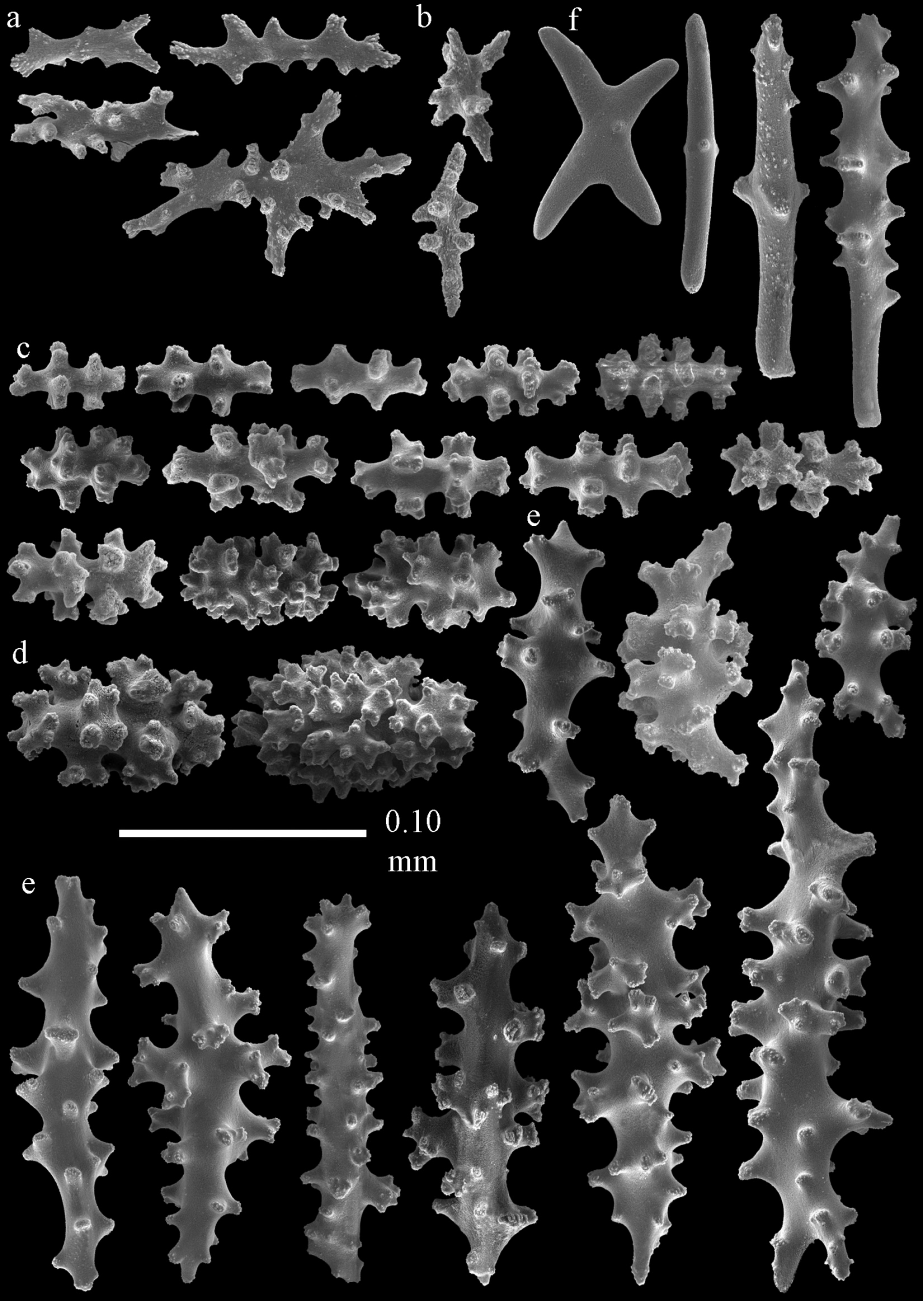
Sclerites of *Melithaea arborea*, ZMH C3305; **a** tentacle sclerites **b** pharynx rods **c** capstans **d** spheroids **e** spindles **f** axis rods.

**Figure 11.**
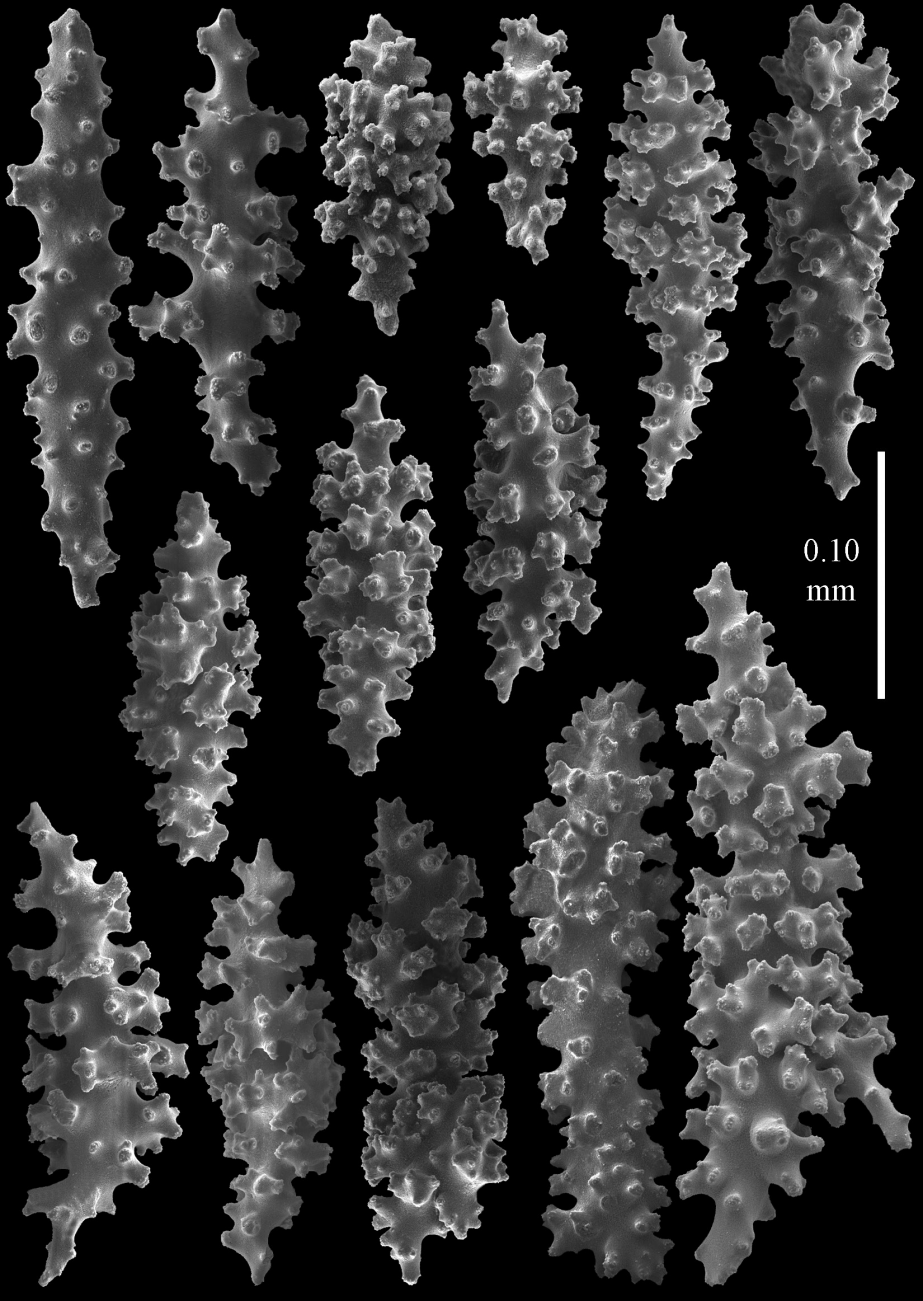
Spindles of *Melithaea arborea*, ZMH C3305.

Figures 8 , 9a , 10 , 11

Melitodes arborea : Kükenthal 1908: 193; 1909: 59, figs 61–63, Pl. 4 fig. 26 (Japan); Kükenthal 1919: 150; 1924: 62; Hickson 1937: 122.

#### Material examined.

Holotype **ZMH C3305**, Sagami Bay (collection number in Kükenthal (1908) incorrect as 63305), coll. A. Austin.

#### Re-description.

Colony bushy (Fig. 9a). Tentacles with platelets, the larger ones crescent-shaped with irregular projections (Fig. 10a). These platelets are up to 0.10 mm long. Pharynx with straight spiny rods, up to 0.05 mm long (Fig. 10b). Coenenchyme with capstans (Fig. 10c), about 0.05 mm long, the bigger ones are spheroids (Fig. 10d); small clubs of similar length; spindles, 0.10–0.30 mm long, with simple or complex tubercles (Figs 10e, 11). The axis has smooth and sparsely tuberculate rods (Fig. 10f).

#### Color.

Red with paler polyps, sclerites orange, tentacle sclerites colorless.

#### Distribution.

The name of collector A. Austin could actually be Alan Owston (Isono 1988), an English trader (import and export merchant and naturalist), who used to collect material of deep-water species. Therefore we suspect *Melithaea arborea* to grow in deeper water. So far it is only found in Sagami Bay (Fig. 8).

#### Remarks.

The colony depicted by Kükenthal (1908) could actually be the basal part of a much larger colony. As with *Melithaea japonica*, many sclerites are disintegrated, and therefore we could not depict the small clubs of the coenenchyme. Also, the sample available to us had hardly any polyp sclerites, since only a few tentacle rods were present (Fig. 10a). Therefore, we assume that they also had disintegrated. Kükenthal (1908) described collaret and point sclerites as being 0.20 mm long, the tentacle rods 0.15 mm long. He did not mention the presence of capstans and small clubs. We found no sclerites resembling clubs referable to calyces and only a few sclerites with a tendency to be unilaterally spinose.

According to Kükenthal (1908) the species resembles mostly *Melithaea japonica*. Indeed the sclerites of these two species are very similar, *Melithaea japonica* showed somewhat more developed unilaterally spinose sclerites and some sclerites resembling clubs. Bearing in mind the colony fragment of *Melithaea arborea* resembles a basal part we do not exclude the possibility *Melithaea arborea* and *Melithaea japonica* represent one and the same species. Therefore, they were given the same position in the key to species identification.

### Melithaea boninensis sp. n.

Taxon classificationAnimaliaAlcyonaceaMelithaeidae

http://zoobank.org/4343DDC1-4FAE-439A-BE5F-2D7B21BD41F7

**Figure 12.**
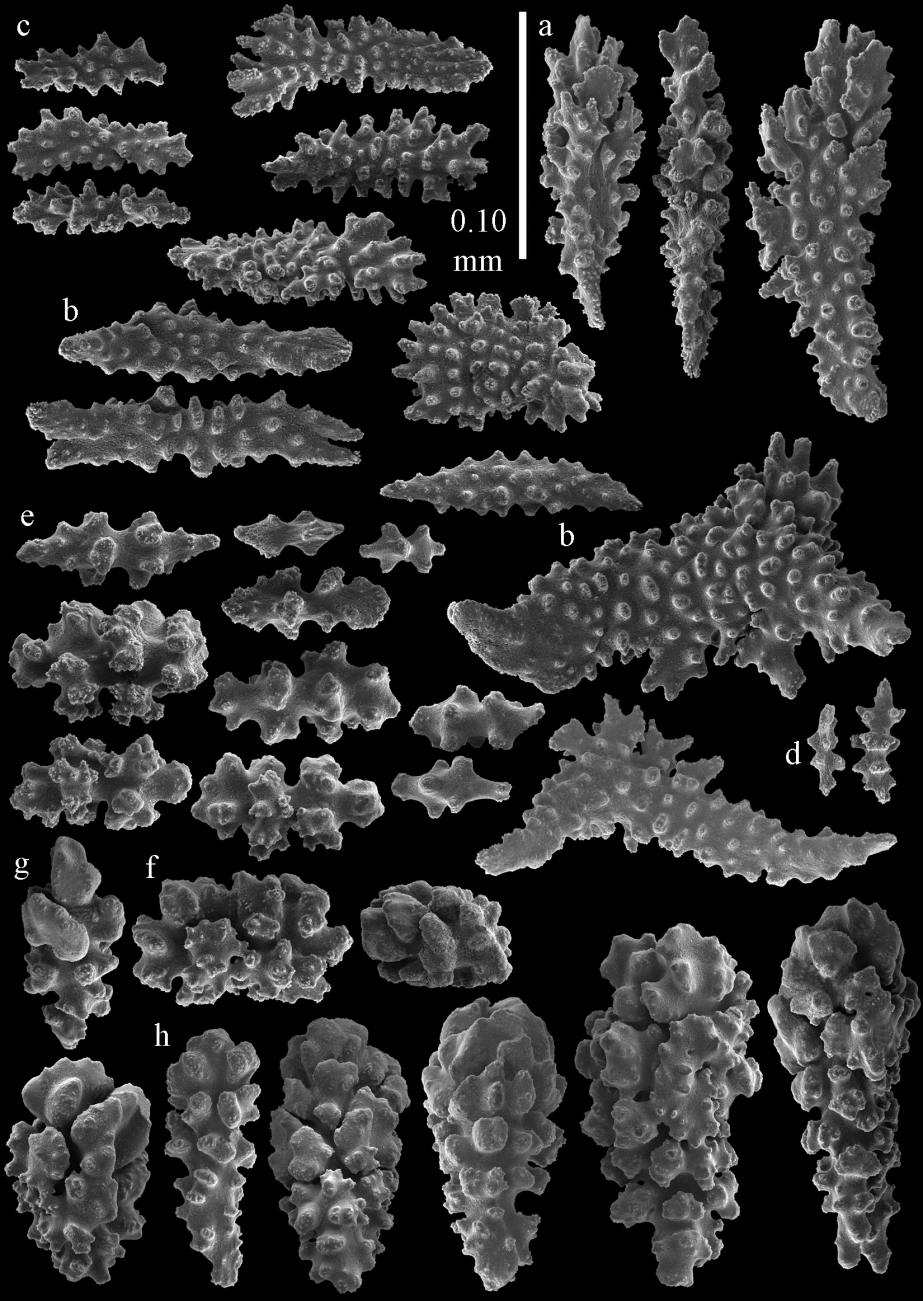
Sclerites of *Melithaea boninensis* sp. n., UMUTZ-CnidG-205; **a** point spindles **b** collaret spindles **c** tentacle sclerites **d** pharynx rods **e** capstans **f** unilaterally foliate spheroids **g** clubs of coenenchyme **h** clubs of calyx.

**Figure 13.**
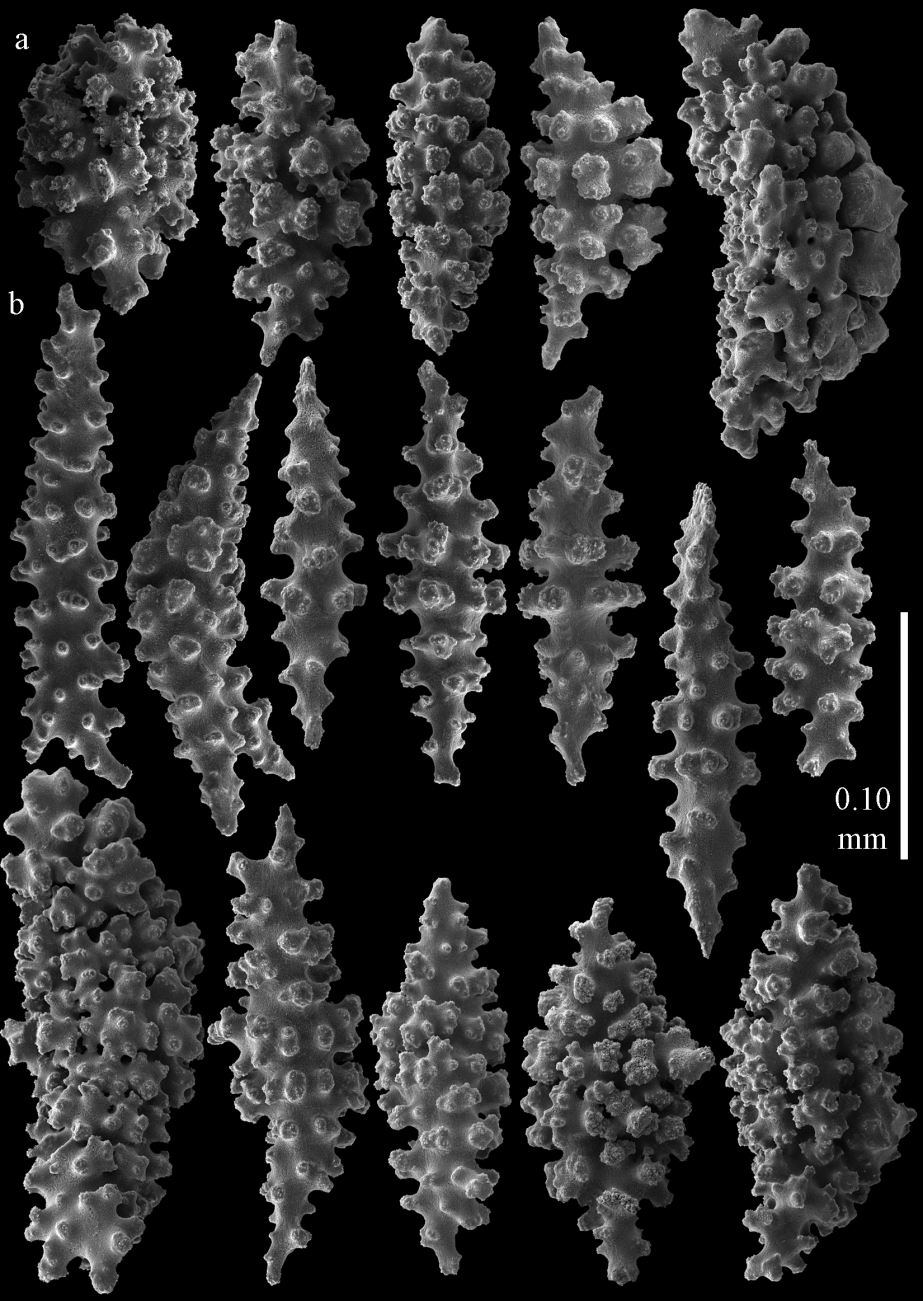
Sclerites of *Melithaea boninensis* sp. n., UMUTZ-CnidG-205; **a** unilaterally foliate spindles of coenenchyme **b** spindles of coenenchyme.

Figures 8 , 9b–c , 12 , 13

#### Material examined.

Holotype **UMUTZ-CnidG-205**, Ogasawara Isls. (= Bonin Isls.), Japan, coll. S. Hirota and Sekiguchi, 11 April 1894; paratype **UMUTZ-CnidG-255**, same data as holotype.

#### Description.

The holotype is 8 cm long and 4.5 cm wide, branching is in two parallel planes, and with a holdfast (Fig. 9b). The stem is 5 mm wide, the end branches only 1 mm wide. The colony has many anastomoses. The polyps are situated all around the branches, the calyces are dome-shaped, and the polyps are retracted. Points with slightly bent spindles up to 0.15 mm long, distal end with leaves (Fig. 12a). Collaret with bent spindles up to 0.20 mm long, middle part with more tubercles or side branches (Fig. 12b). Tentacles with platelets, the larger ones crescent-shaped with irregular projections (Fig. 12c). These platelets are up to 0.10 mm long. Pharynx with straight spiny rods, up to 0.05 mm long (Fig. 12d). Coenenchyme with capstans (Fig. 12e), unilaterally foliate spheroids (Fig. 12f), 0.05–0.10 mm long and small clubs of similar length (Fig. 12g); spindles (Fig. 13b) and unilaterally foliate spindles (Fig. 13a) are 0.10–0.20 mm long. The calyces with longer clubs, up to 0.15 mm long (Fig. 12h). Most coenenchymal sclerites have complex tubercles.

#### Color.

The colony and sclerites are orange.

#### Distribution.

The species is only known from the Ogasawara Islands (= Bonin Islands) (Fig. 8).

**Etymology.** The species is named after the type locality, the Bonin Islands.

#### Remarks.

This is the first record of *Melithaea* from this island group. The colony shape of paratype UMUTZ-CnidG-255, collected at the same time with G205, looks similar toZ-CnidG-205, but it has disintegrated sclerites. The colony is slightly smaller and brighter orange-colored (Fig. 9c). Its sclerites are similar to those of the holotype. This is the only species that looks like *Melithaea habereri* (Kükenthal, 1908), having many anastomoses, but its sclerites are quite different, many with leaves, while Kükenthal described spiny sclerites for *Melithaea habereri*.

### Melithaea corymbosa

Taxon classificationAnimaliaAlcyonaceaMelithaeidae

(Kükenthal, 1908)

Figures 14 , 15 , 16 , 17 , 18 , 19 , 20 , 21 , 22 , 23 , 24 , 25 , 26 , 27

Acabaria corymbosa : Kükenthal 1908: 197; 1909: 70, figs 81-83, pl. 6 fig. 31 (Misuki (= Misaki), Japan); Kükenthal 1919: 187; 1924: 81; Aurivillius 1931: 24 (Sagami Bay); Hickson 1937: 177.

#### Material examined.

Syntype **ZMB 5814**, Misaki (Japan), coll. Doflein, 1904/05; previously unidentified museum material: **ZMUC ANT-000587**, Okinose, Sagami Bay, Japan, 100 fms (143–183 m), coll. Dr. Th. Mortensen, 26 June 1914; **ZMUC ANT-000588**; Sagami Bay, Japan, 80–120 fms (114–219 m), coll. Dr. Th. Mortensen, 6–19 June 1914; **ZMUC ANT-000590**, Okinose, Sagami Sea, 100 fms (143–183 m), coll. Dr. Th. Mortensen, 15 June 1914; **ZMUC ANT-000646**, Off Nagasaki, 32°15'N, 128°12'E, 90 fms (165 m), coll. Dr. Th. Mortensen, 15 May 1914; **ZMUC ANT-000654**, Okinose, Sagami Sea, 100 fms (143–183 m),, coll. Dr. Th. Mortensen, 23 June 1914; **ZMUC ANT-000659**, Okinose, Sagami Bay, 200 fms (286–366 m), coll. Dr. Th. Mortensen, 1 July 1914; **ZMUC ANT-000661**, same data as ZMUC ANT-000654; **ZMUC ANT-000650**, Off Misaki, Sagami Bay, ca.250 fms (ca.358–457 m), coll. Dr. Th. Mortensen, 10 June 1914; **UMUTZ-CnidG-15**, Japan; **UMUTZ-CnidG-17**, Gorgonian cave at Koajiro, Misaki, Sagami Bay, 16 July 1897; **UMUTZ-CnidG-23 (G-23a)**, same data as UMUTZ-CnidG-17; **UMUTZ-CnidG-268 (G-37d)**, Misaki, Sagami Bay, coll. K. Kinoshita, summer 1906; **UMUTZ-CnidG-41**, Awa Kominato, Boso Peninsula, Chiba Prefecture, coll. sp. no. 78, April 1885; **UMUTZ-CnidG-271 (G-41b)**, same data as UMUTZ-CnidG-41; **UMUTZ-CnidG-199**, Sengenzuka-Aoyamadashi line, Sagami Bay, 100 hiro (143–151 m), coll. H. Matsumoto and H. Chiba, 20 July 1913; **RMNH Coel. 41902 (AKM 223)**, South of Mera-se bank, Sagami Bay, 34°59.6'N, 139°41.1'E - 34°59.7'N, 139°41.1'E, 81–78 m, *R/V Shinyo-maru*, St.1, coll. A.K. Matsumoto, 17 October 2003; **AKM 225**, same data as RMNH Coel.41902; **AKM 242**, South of Mera-se Minami knoll, 34°54.8'N, 139°39.7'E - 34°54.8'N, 139°39.9'E, 348–312 m, *R/V Shinyo-maru*, coll. A.K. Matsumoto, 18 October 2003; **AKM 245**, South of Mera-se bank, Sagami Bay, 34°54N 139°39E, 315-365m, *R/V Shinyo-maru*, coll. A.K. Matsumoto, 18 October 2003; **AKM 246**, South of Mera-se Minami knoll, 34°54.2'N, 139°39.9'E - 34°54.3'N, 139°39.3'E, 348–312 m, *R/V Shinyo-maru*, coll. A.K. Matsumoto, 18 October 2003; **AKM 248**, same data as AKM245; **AKM 253**, Sagami Bay, 33°26.3'N, 139°42.3'E - 33°26.5'N, 139°42.0'E, 157–172 m, *R/V Shinyo-maru*, K-32, St. 18, coll. A.K. Matsumoto, 21 October 2003; **AKM 299**, Sagami Sea, 33°27'N, 139°42'E, 200–211 m, *R/V Shinyo-maru*, coll. A.K. Matsumoto, 21 October 2003; **RMNH Coel. 41903 (AKM 513)**, Otsuki, Tosa, Kochi Prefecture, 132°50.44'E 32°37.66'N, - 132°47.88'E 32°37.56'N, 114 m, local fishermen’s boat, *Kiryo-maru*, st.1, coral net, coll. A.K. Matsumoto, 7 October 2004; **AKM 578**, off Ohakozaki cape, Otsuchi, Iwate Prefecture, 39°21.338N 142°00.721E, 75 m, *R/V Yayoi*, St. 2.3.4, coll. A.K. Matsumoto, 22 February 2005; **AKM 595**, entrance of Otsuchi Bay, Otsuchi, Iwate Prefecture, 39°21.858N 141°59.972E, 65.6 m, *R/V Yayoi*, St.1, coll. A.K. Matsumoto, 12 September 2005; **RMNH Coel. 41904 (AKM 840b)**, East of Jogashima Spur, 35°03.52'N, 139°37.43'E - 35°04.17'N, 139°37.52'E, 397–286 m, *R/V Tansei-maru*, KT07-31, st. 8, coll. A.K. Matsumoto, 25 November 2007; **RMNH Coel. 41905 (AKM 886)**, Hachijo Is., Izu Isls., 33°20.9082'N, 139°41.1841'E – 33°21.0775'N, 139°40.4931'E, 213–185 m, *R/V Tansei-maru*, KT07-31 (Kuramochi leg.), St.14 (L-7-200), Chain Bag Dredge, coll. A.K. Matsumoto, 26 November 2007; **RMNH Coel. 41906 (AKM 928)**, Hachijo Is., Izu Isls., 33°22.5320'N, 139°40.492'E – 33°22.3111'N, 139°40.2511'E, 202–145 m, *R/V Tansei-maru*, KT07-31, St.15 (L-7-100), Chain Bag Dredge, coll. A.K. Matsumoto, 26 November 2007; **RMNH Coel. 41907 (AKM 949)**, Toshima Is., Izu Isls., 34°33.1102'N, 139°17.4102'E – 34°33.6524'N, 139°17.6725'E, 143 m, *R/V Tansei-maru*, KT07-31, Kuramochi leg., St.22 (L-3-100), Chain Bag Dredge, coll. A.K. Matsumoto, 27 November 2007; **RMNH Coel. 41908 (AKM 1175)**, off Kerama Is. Okinawa Prefecture, East China Sea, 127°27.70'E – 127°27.95'E, 26°04.59'N, – 26°04.56'N, 160–153 m, *R/V Tansei-maru*, KT08-33 cruise, St. KR-07, Chain Bag Dredge, coll. A.K. Matsumoto, 16 December 2008; **RMNH Coel. 41909 (AKM 1176)**, same data as AKM 1175; **RMNH Coel. 41910 (AKM 1320)**, off Kerama Islands. Okinawa Prefecture, East China Sea, 26°00'N, 127°12'E, 100–97 m, *R/V Tanisei-maru*, KT08-33, st. KR-3, coll. A.K. Matsumoto, 18 December 2008; **RMNH Coel. 41911 (AKM 1321)**, same data as AKM 1320; **AKM 1519**, off Funakoshi Bay, Iwate Prefecture, 101 m, *R/V Yayoi*, St. 2-5, CO N, coll. A.K. Matsumoto, 26 April 2010; **AKM 1525**, off Oshima Is. Entrance of Otsuchi Bay and Funakoshi Bay, Iwate Prefecture, 39°22.085'N, 142°01.152'E, 97 m, by *R/V Yayoi*, 1 m biological dredge. coll. A.K. Matsumoto, 26 April 2010; **AKM 1545**, off Ohako-zaki Cape, Otsuchi, Iwate Prefecture, 86.5 m, *R/V Yayoi*, st. 2-2, coll. A.K. Matsumoto, 27 April 2010; **RMNH Coel. 41912** (**AKM 1602**), South East off Taito-saki, Boso Peninsula, 35°09.31'N, 140°48.57'E – 35°09.60'N, 140°49.40'E, 311–325 m, *R/V Tansei-maru*, KT95-05, st. TB14, coll. S. Ohta, 26 April 1995; **RMNH Coel. 41913 (AKM 1603)**, Okinoyama Basin, Sagami Bay, 86–88 m, *R/V Tansei-maru*, KT87-19, st. OKI, 1 m ORI biological dredge, coll. S. Ohta, 10 December 1987.

#### Re-description.

**Figure 14.**
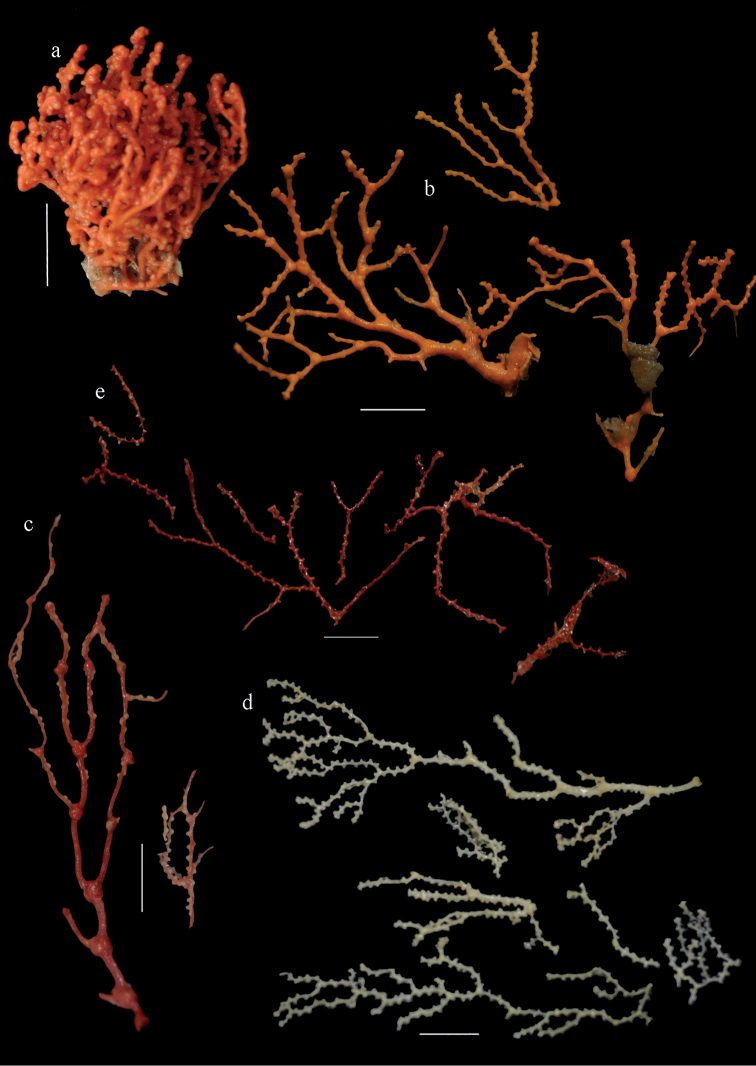
*Melithaea corymbosa*, **a** ZMB 5814, syntype **b** ZMUC ANT-000587 **c** RMNH Coel. 41903 **d** RMNH Coel. 41908 **e** RMNH Coel. 41911. Scale bars 1 cm.

**Figure 15.**
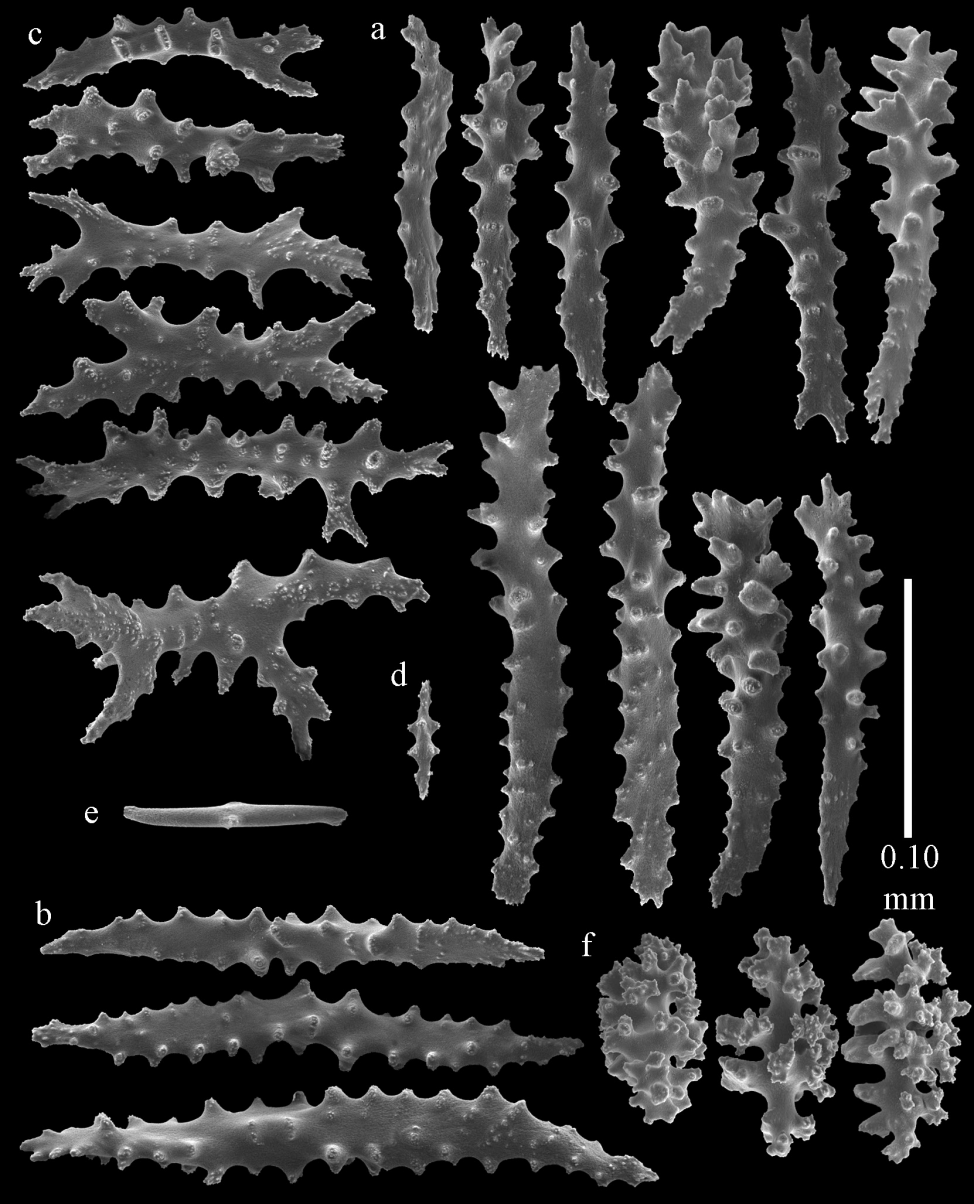
Sclerites of *Melithaea corymbosa*, ZMB5814 syntype; **a** point spindles **b** collaret spindles **c** tentacle sclerites **d** pharynx rod **e** axis rod **f** unilaterally spinose spindles of coenenchyme.

**Figure 16.**
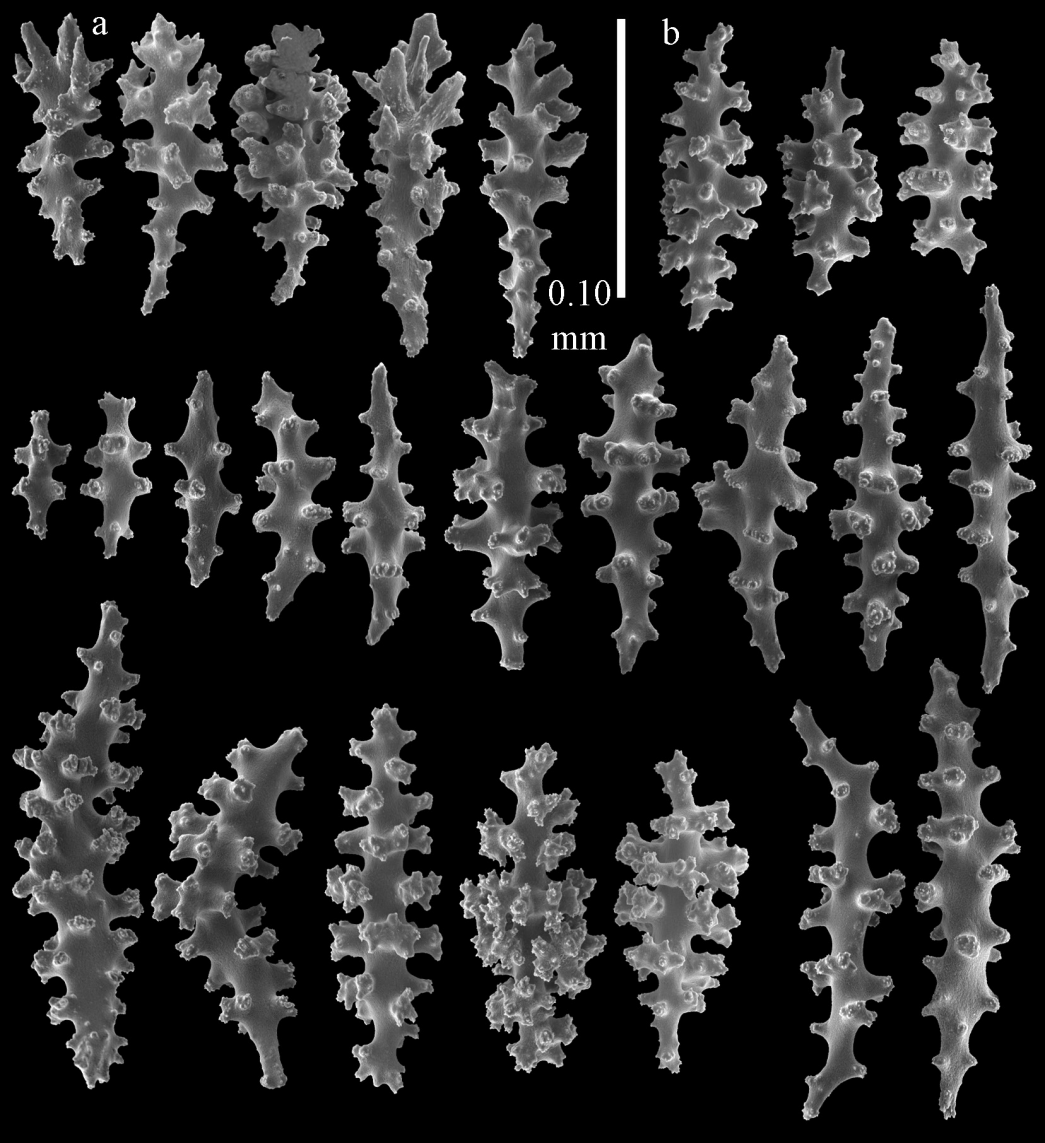
Sclerites of *Melithaea corymbosa*, ZMB5814 syntype; **a** clubs of calyces **b** spindles of coenenchyme.

Colony bushy with anastomoses; end branches flattened (Fig. 14a). Points with slightly bent spindles up to 0.20 mm long, distal end with spines (Fig. 15a). Collaret with bent spindles up to 0.25 mm long, middle part with more tubercles (Fig. 15b). Tentacles with platelets, the larger ones crescent-shaped with irregular projections (Fig. 15c). These platelets are up to 0.17 mm long. Pharynx with straight spiny rods, up to 0.05 mm long (Fig. 15d). Coenenchyme with predominantly spindles, 0.10-0.18 mm long (Fig. 16b), with simple or complex tubercles. A few capstans and unilaterally spinose spindles also present (Fig. 13f). Calyces with thorn clubs, 0.10-0.12 mm long (Fig. 16a).

#### Color.

Colony red with yellow tentacles; tentacle and pharynx sclerites colorless, all others pink.

#### Variation.

**Figure 17.**
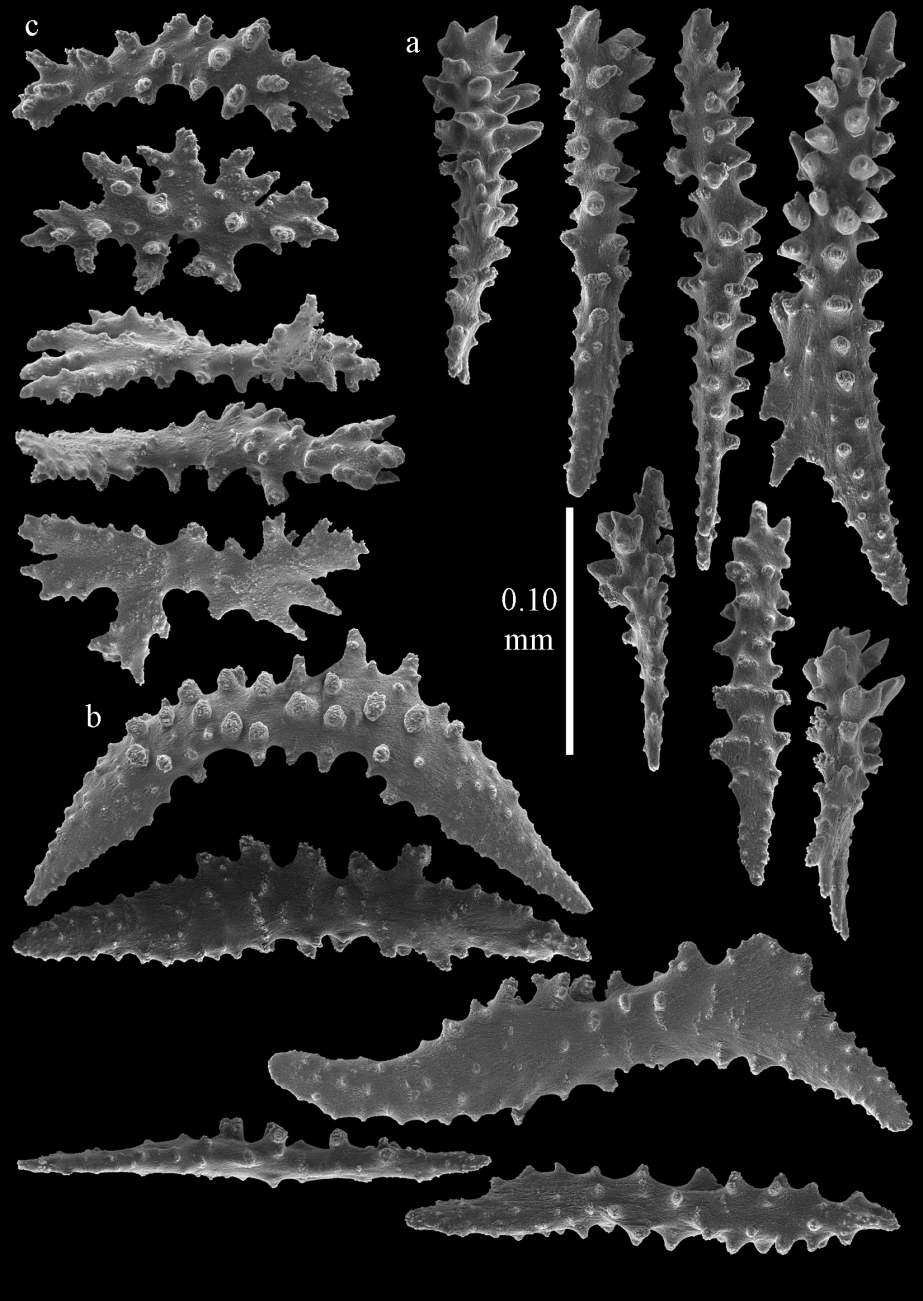
Sclerites of *Melithaea corymbosa*, ZMUC ANT-000587; **a** point spindles **b** collaret spindles **c** tentacle sclerites.

**Figure 18.**
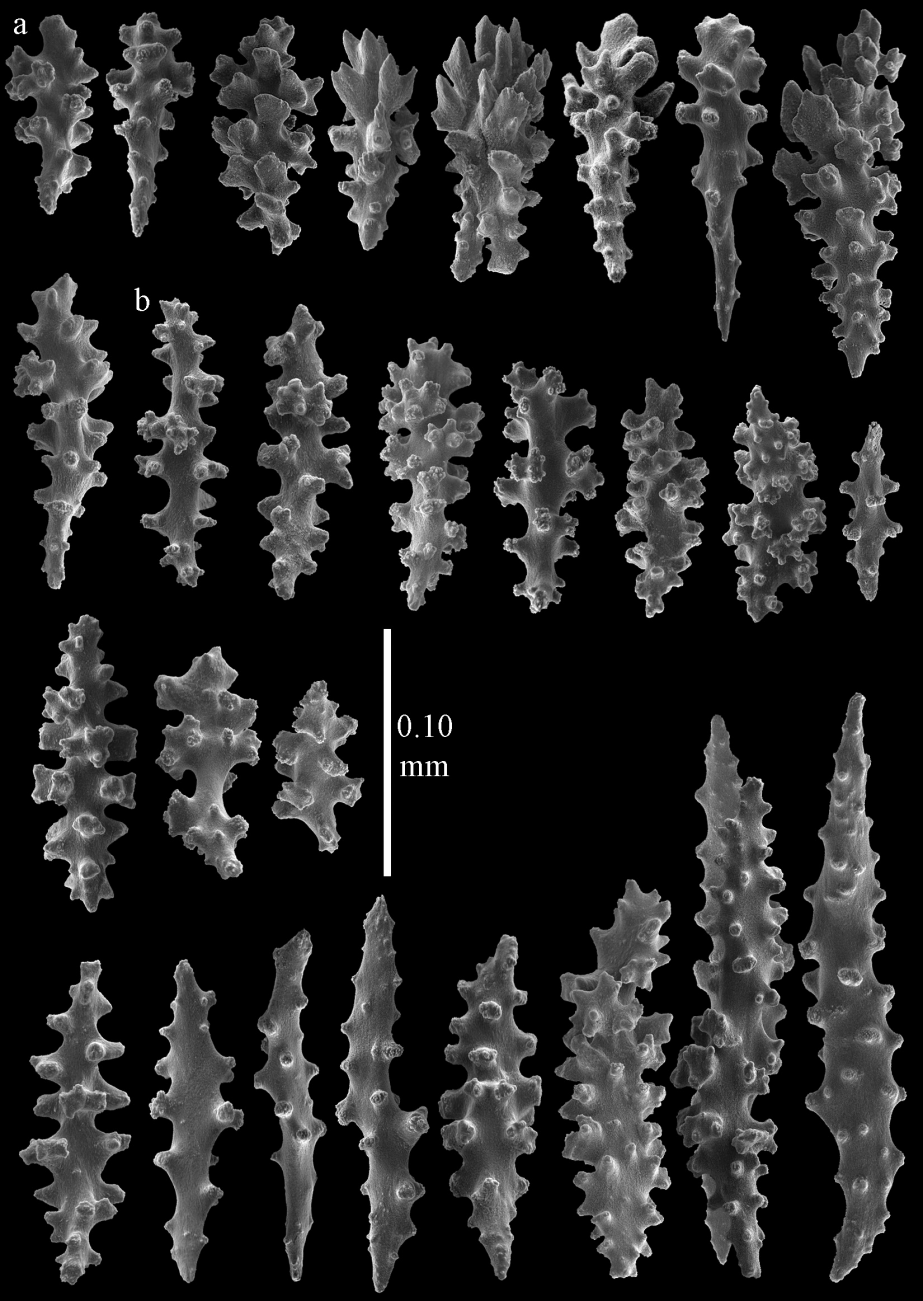
Sclerites of *Melithaea corymbosa*, ZMUC ANT-000587; **a** clubs of calyces **b** spindles of coenenchyme.

**Figure 19.**
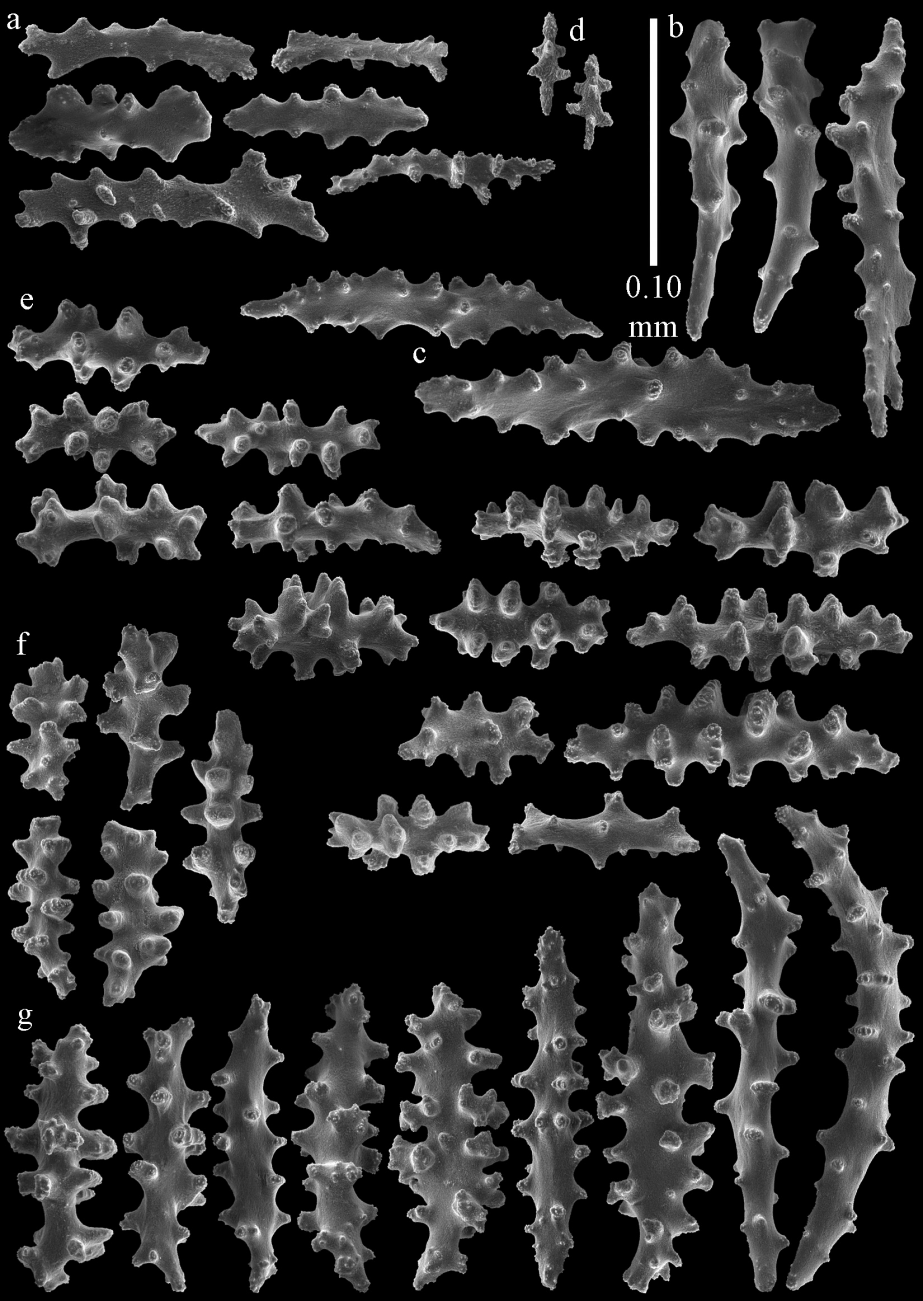
Sclerites of *Melithaea corymbosa*, RMNH Coel. 41912; **a** tentacle rods **b** point spindles **c** collaret spindles **d** pharynx rods **e** unilaterally spinose spindles of coenenchyme **f** clubs **g** spindles of coenenchyme.

**Figure 20.**
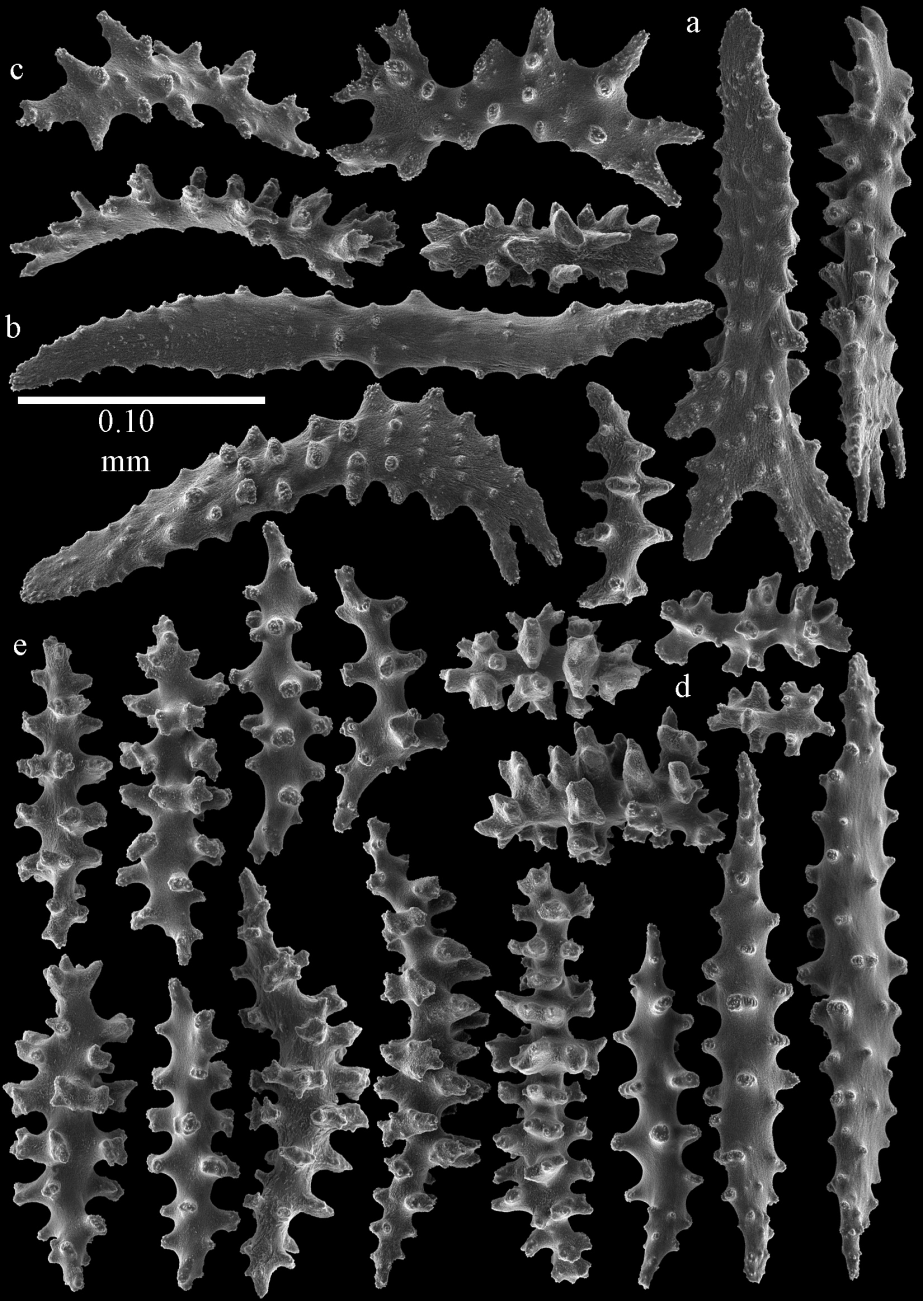
Sclerites of *Melithaea corymbosa*, RMNH Coel. 41913; **a** point spindles **b** collaret spindles **c** tentacle sclerites **d** unilaterally spinose spindles of coenenchyme **e** spindles of coenenchyme.

ZMUC ANT-000587 (Fig. 14b) has a different colony shape than the syntype but the same locality, color and sclerites (Figs 17, 18). The species shows much color variation, RMNH Coel. 41902 has colorless and pink coenenchymal sclerites and yellow polyp ones. RMNH Coel. 41907 has orange coenenchymal sclerites and pink polyp ones. ZMUC ANT-000654 has colorless and pink coenenchymal sclerites, orange collaret and point sclerites and yellow tentacle ones; RMNH Coel. 41904 and RMNH Coel. 41905 show orange coenenchymal sclerites and colorless polyp ones; ZMUC ANT-000646 has a mixture of yellow and orange sclerites in both coenenchyme and polyps; RMNH Coel. 41906 is yellow with yellow sclerites. RMNH Coel. 41912 has an unique color pattern in *Melithaea corymbosa*, red colony with white axis. Its sclerites are also slightly different, the polyp sclerites are less tuberculate, clubs of calyces are smaller, longer ones being very scarce, and the unilaterally spinose spindles are less developed (Fig. 19). This is probably due to the preservation in formalin. RMNH Coel. 41913 has extremely well developed unilaterally spinose spindles (Fig. 20).

#### Distribution.

Pacific coast of Japan; Sagami Bay; Izu Isls.; Boso Peninsula (Chiba Prefecture); Otsuchi (Sanriku, Iwate Prefecture); Otsuki (Tosa, Kochi Prefecture); and East China Sea; off Nagasaki; off Kerama Is.(Fig. 27).

#### Remarks.

The other syntype is in Hamburg, ZMH C3299.

**Figure 21.**
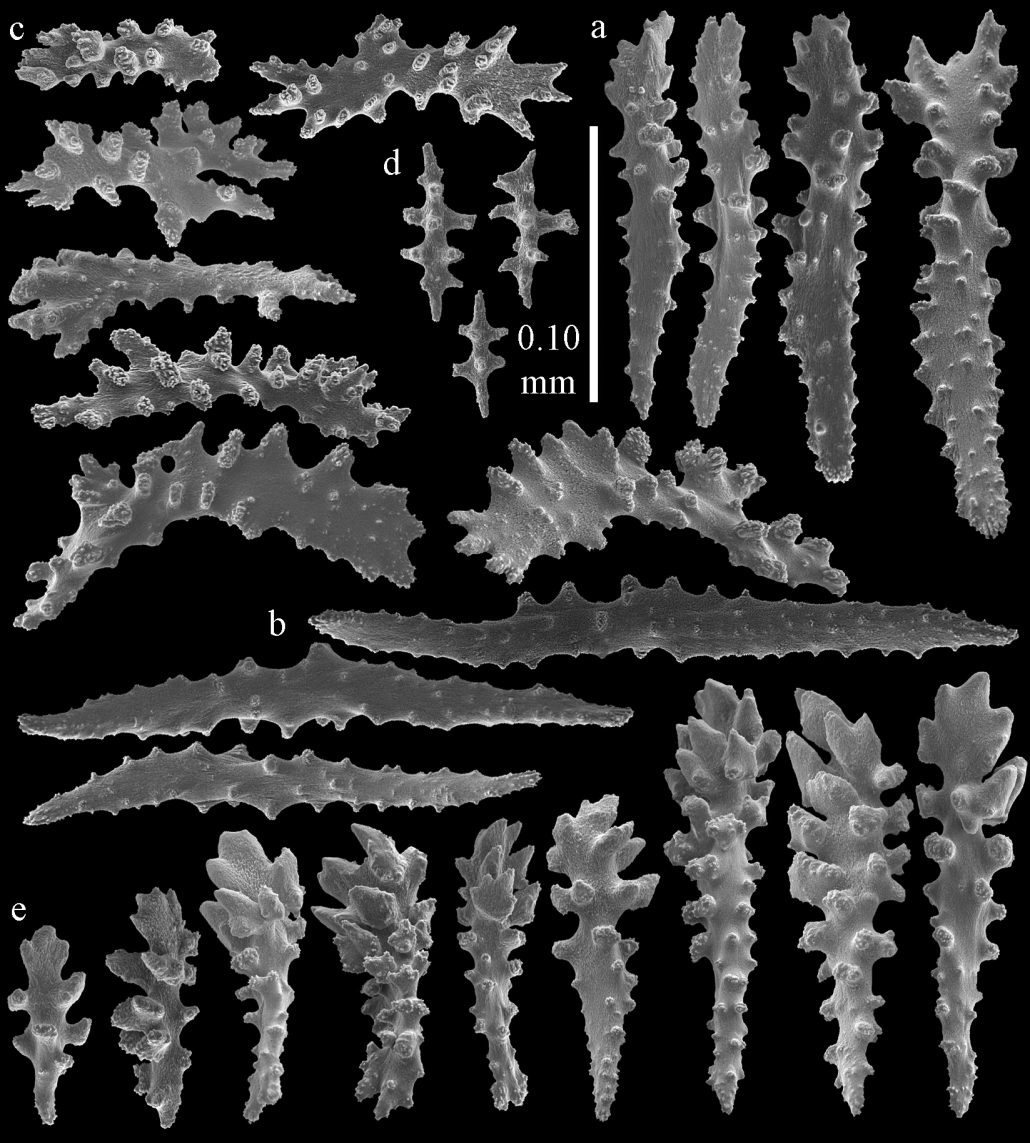
Sclerites of *Melithaea corymbosa*, RMNH Coel. 41903; **a** point spindles **b** collaret spindles **c** tentacle sclerites **d** pharynx rods **e** clubs.

**Figure 22.**
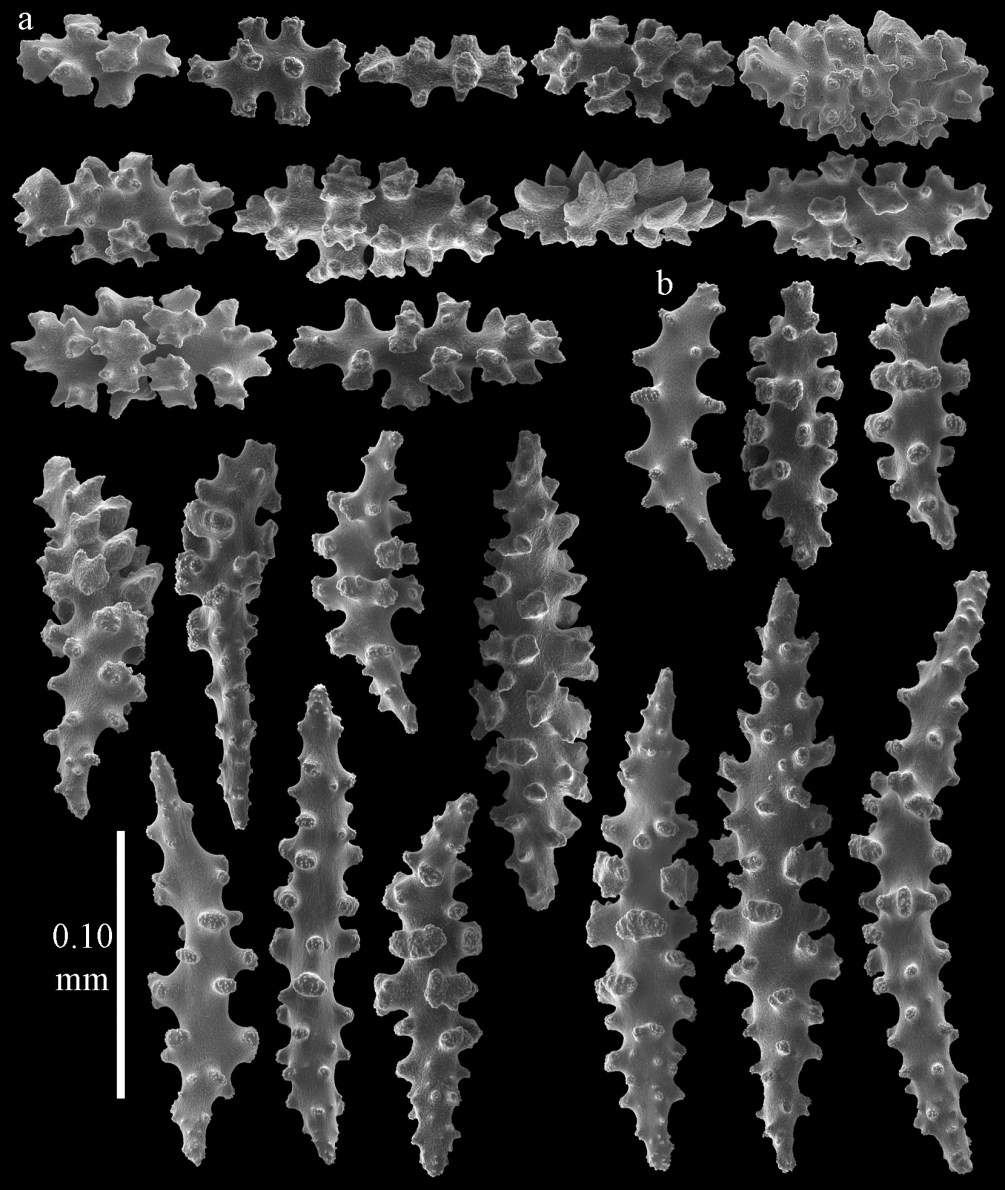
Sclerites of *Melithaea corymbosa*, RMNH Coel. 41903; **a** capstans, derivatives of capstans, and unilaterally spinose spindles of coenenchyme **b** spindles of coenenchyme.

**Figure 23.**
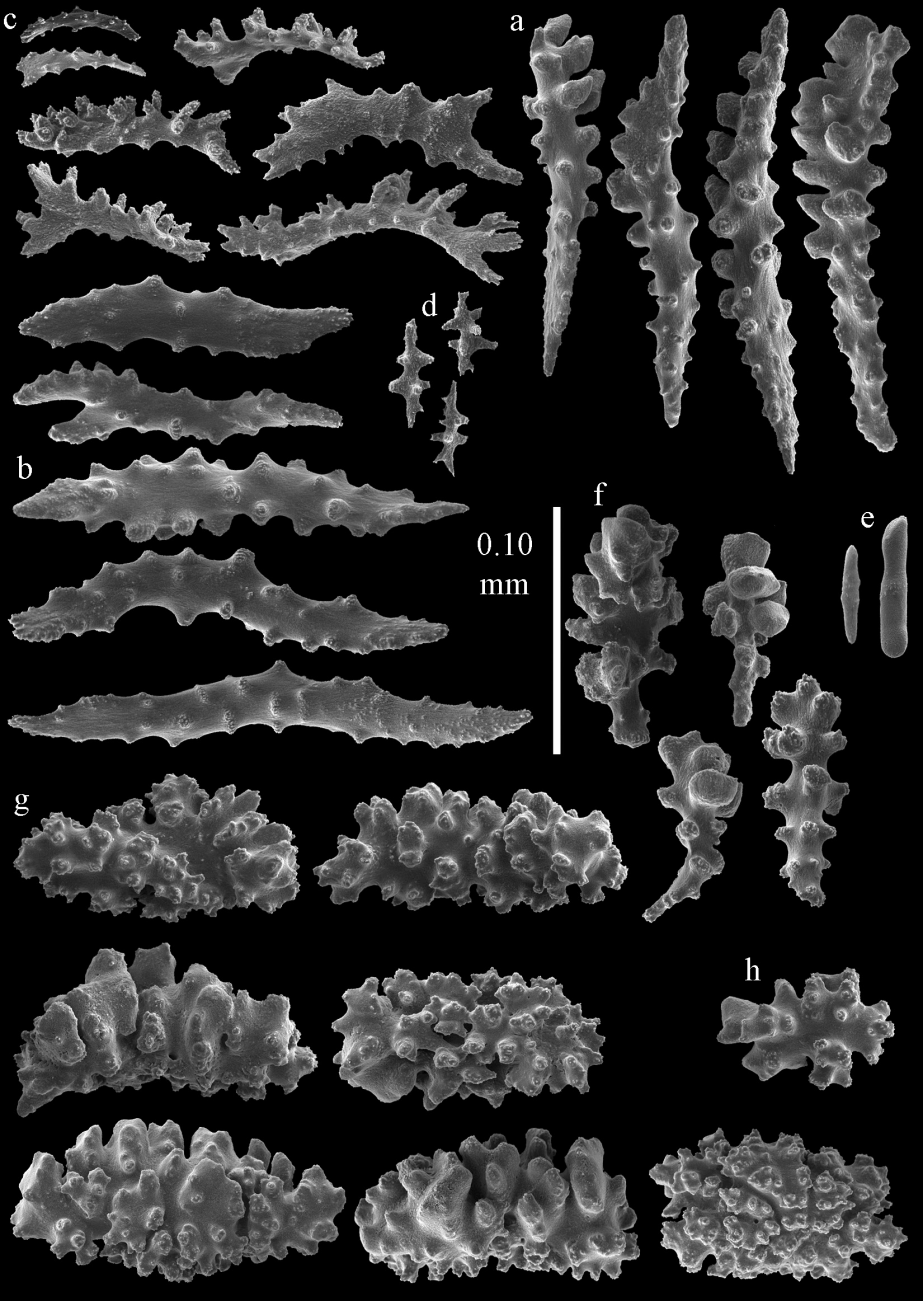
Sclerites of *Melithaea corymbosa*, RMNH Coel. 41908; **a** point spindles **b** collaret spindles **c** tentacle sclerites **d** pharynx rods **e** axis rods **f** clubs **g** unilaterally spinose spheroids **h** capstan.

**Figure 24.**
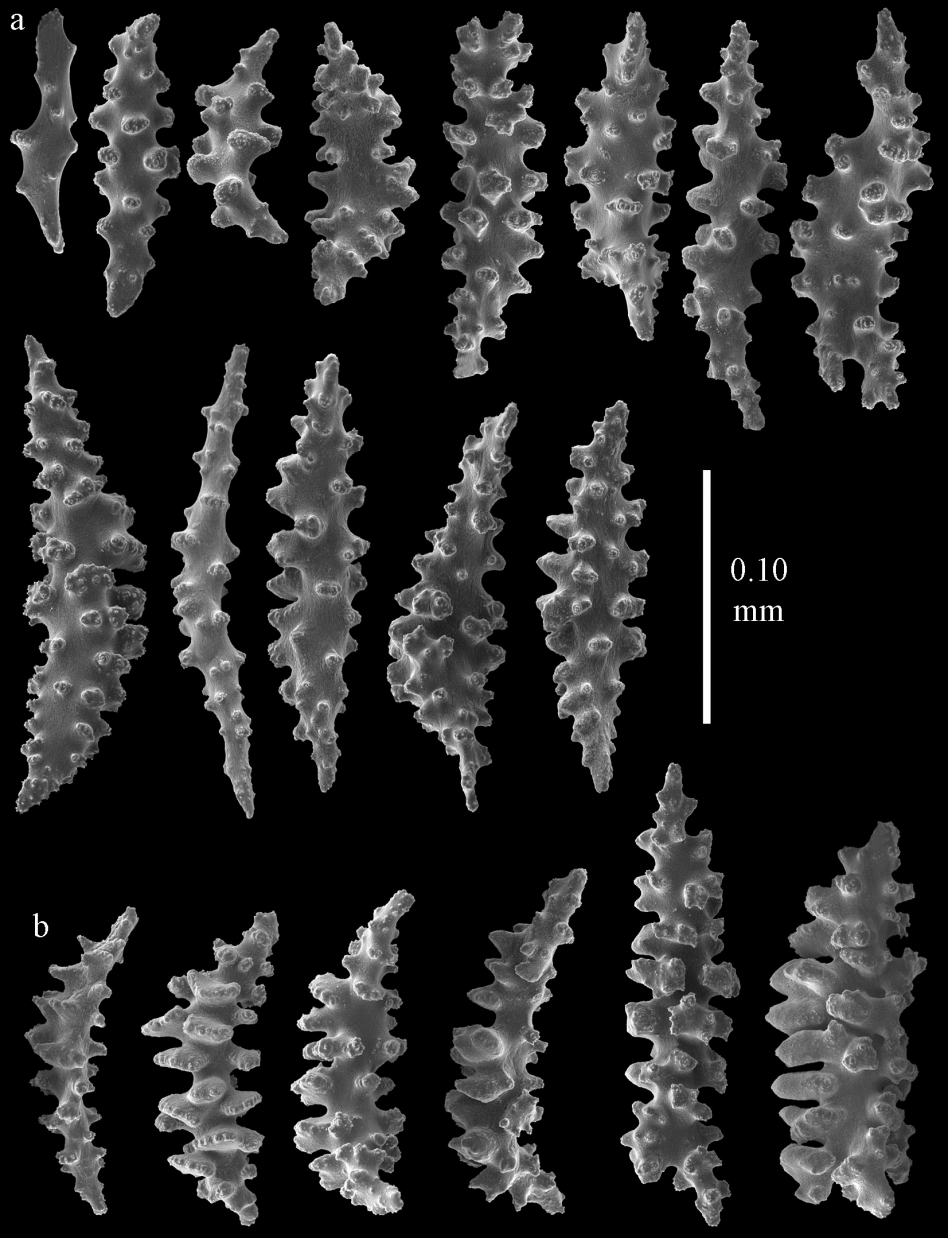
Sclerites of *Melithaea corymbosa*, RMNH Coel. 41908; **a** spindles of coenenchyme **b** unilaterally spinose spindles of coenenchyme.

**Figure 25.**
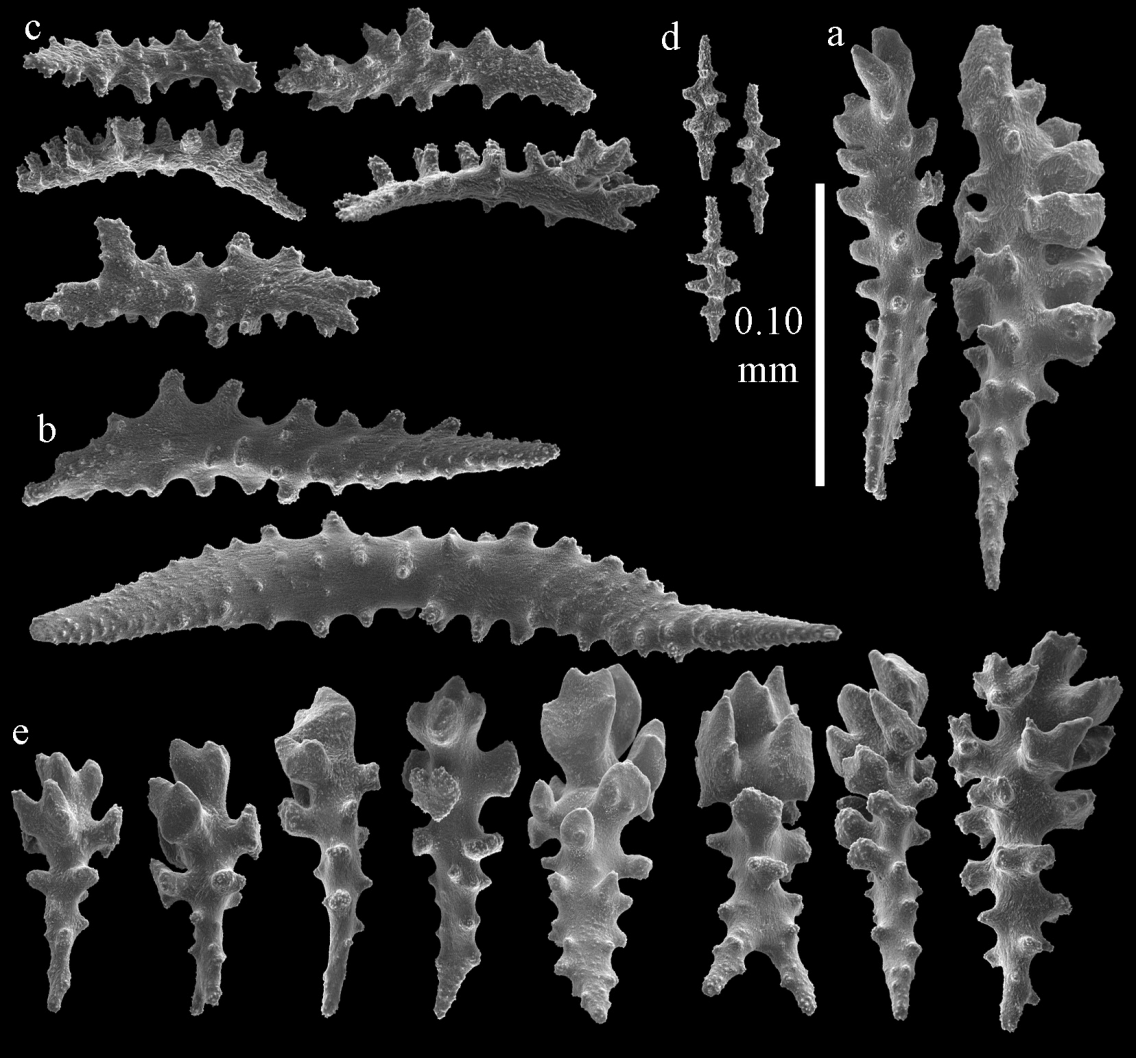
Sclerites of *Melithaea corymbosa*, RMNH Coel. 41911; **a** point spindles **b** collaret spindles **c** tentacle sclerites **d** pharynx rods **e** clubs.

**Figure 26.**
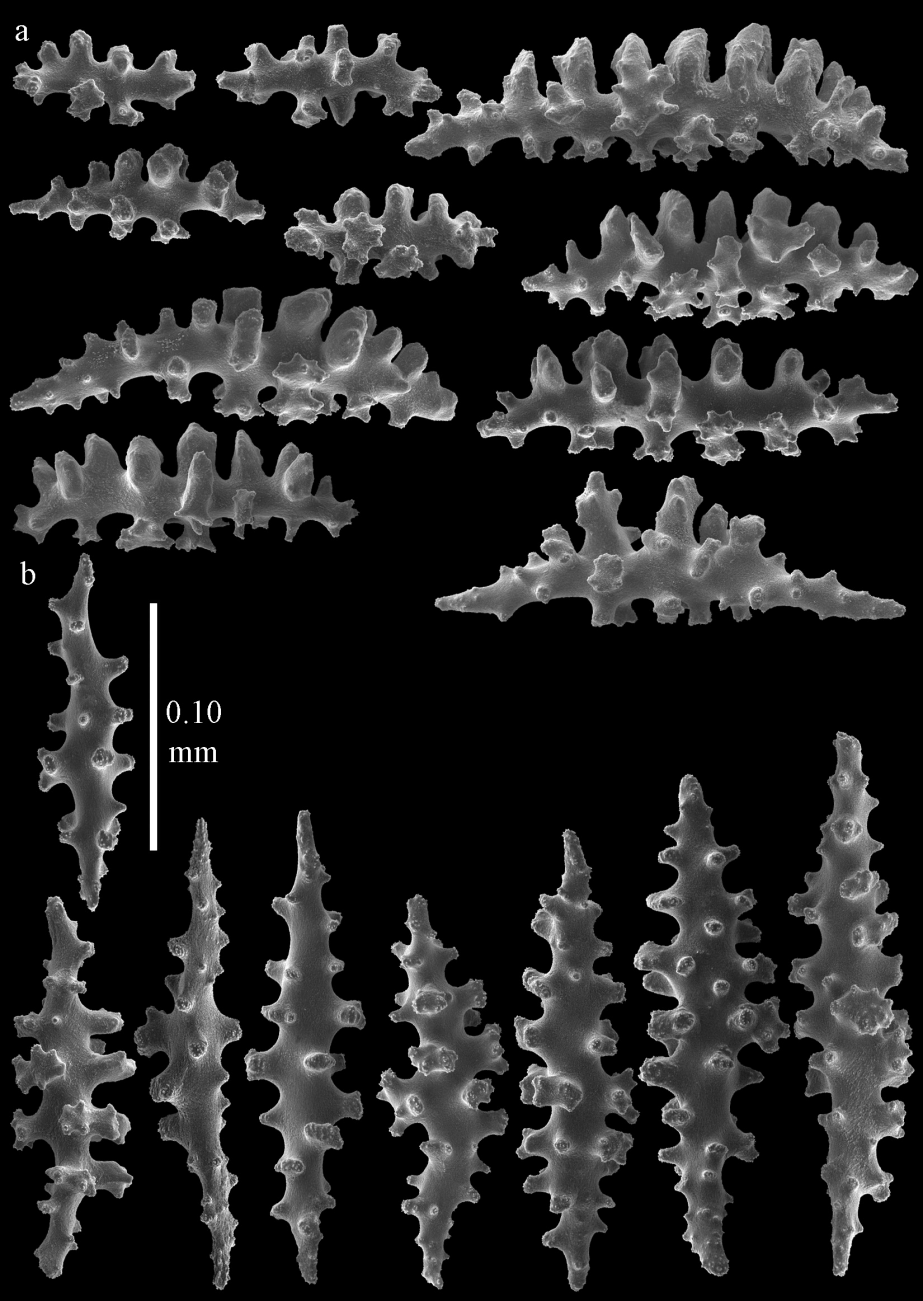
Sclerites of *Melithaea corymbosa*, RMNH Coel. 41911; **a** unilaterally spinose spindles of coenenchyme **b** spindles of coenenchyme.

We included a number of specimens in *Melithaea corymbosa*, which show differences from the description above. Because of the limited material and rather small differences we refrain from describing them as new species. The specimens differ as follows: RMNH Coel. 41903 has a red colony color with white polyps (Fig. 14c), all coenenchymal sclerites are colorless, the axis sclerites are pink. It differs from *Melithaea corymbosa* in having more capstans and derivatives of capstans (Figs 21, 22). RMNH Coel. 41908 (Fig. 14d) and RMNH Coel. 41909 are white colonies with colorless sclerites. They differ from *Melithaea corymbosa* in having many small clubs with rounded heads (Figs 23, 24). RMNH Coel. 41910 and RMNH Coel. 41911 (Fig. 14e) have more unilaterally spinose spindles than normal for *Melithaea corymbosa* (Figs 25, 26). Both colonies come from the same locality but have different color patterns. RMNH Coel. 41910 is orange with white calyces and polyps; sclerites of polyps and calyces colorless, others orange; RMNH Coel. 41911 is red with orange sclerites. ZMUC ANT-000646 has an orange colony with white polyps, sclerites yellow with colorless polyp sclerites. It differs from *Melithaea corymbosa* in having more capstans and derivatives of capstans. In this respect it resembles RMNH Coel. 41903, from which it differs in having overall more tuberculate sclerites.

**Figure 27.**
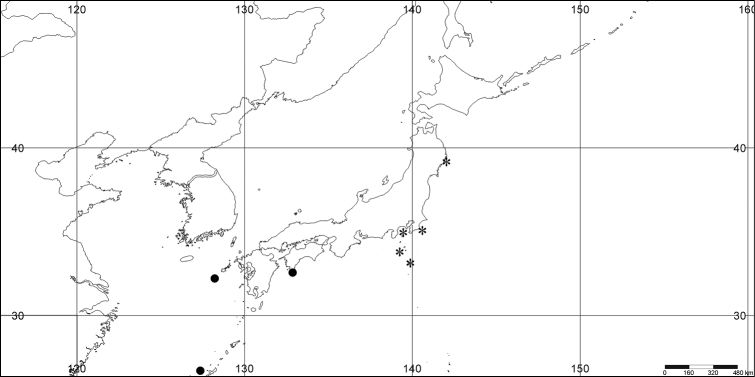
Distribution of *Melithaea corymbosa* (*), problematic specimens (●).

### Melithaea doederleini sp. n.

Taxon classificationAnimaliaAlcyonaceaMelithaeidae

http://zoobank.org/DE3897ED-B96D-497B-8918-ED2E51BB84DB

**Figure 28.**
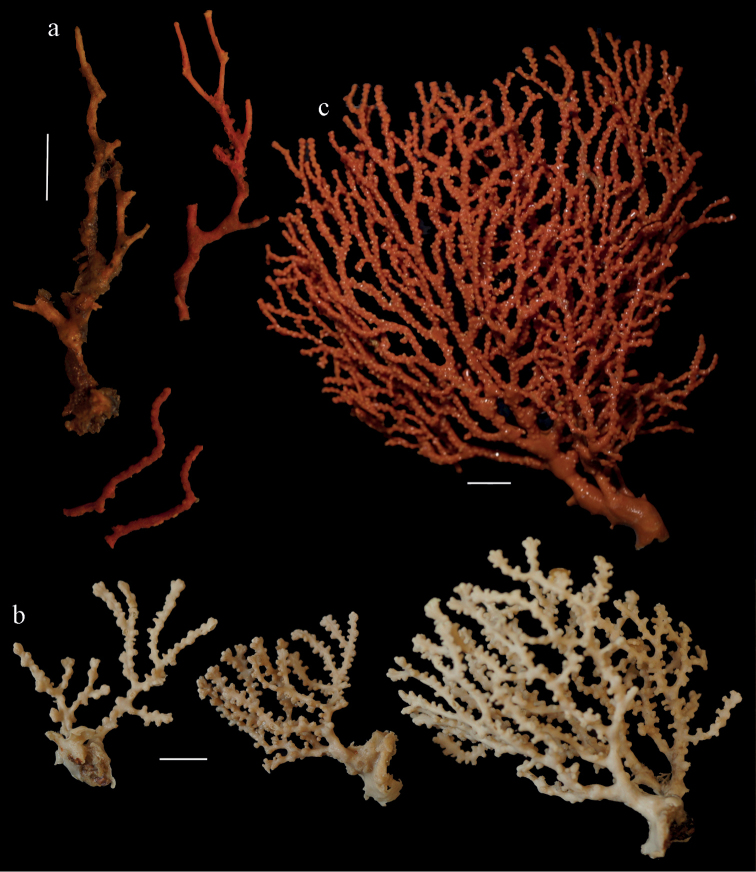
**a** *Melithaea doederleini* sp. n., MZS-Cni61 holotype **b** *Melithaea frondosa* (Brundin, 1896), UPSZTY 2164 syntypes **c** *Melithaea isonoi* sp. n., UMUTZ-CnidG-34 holotype.

**Figure 29.**
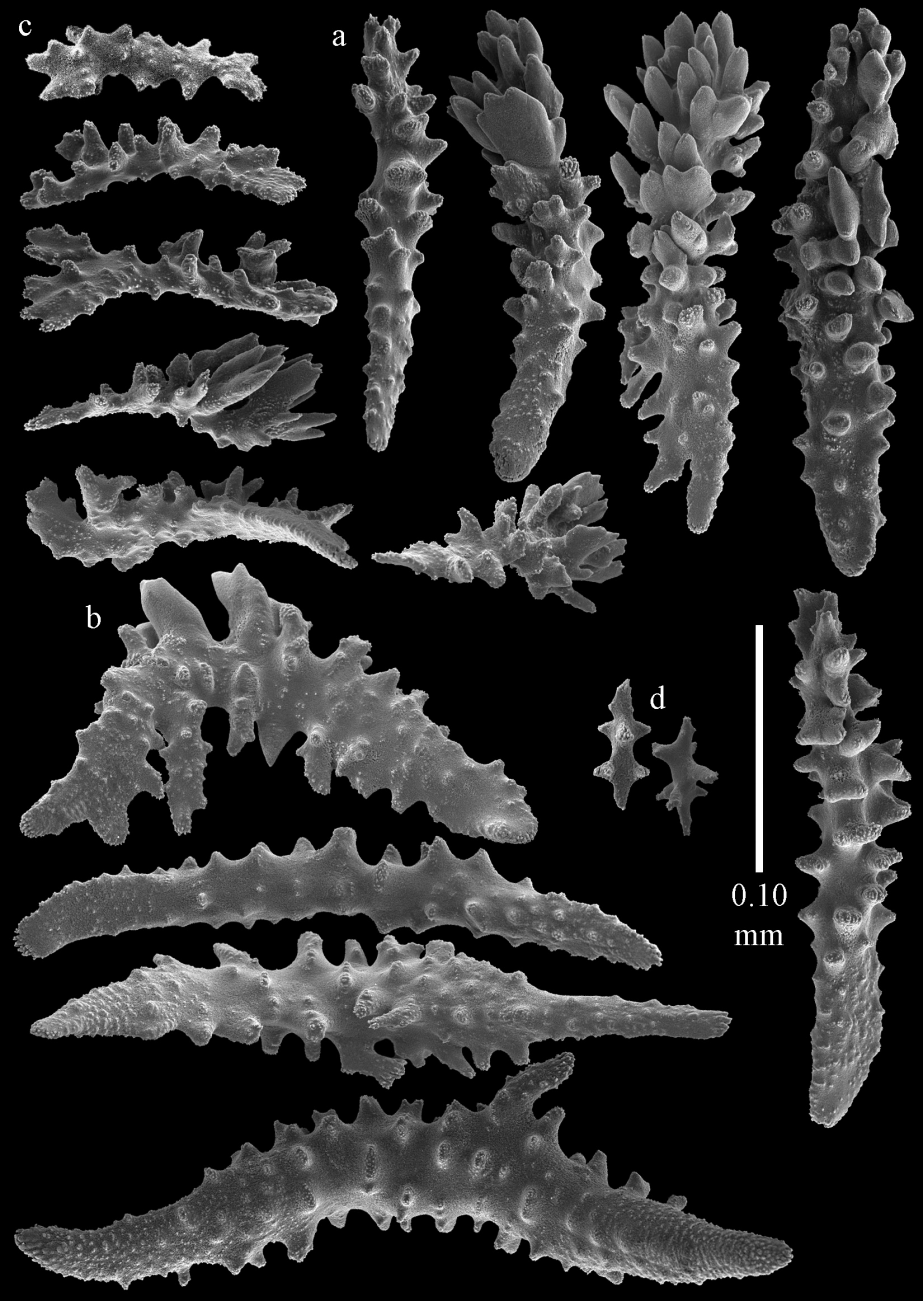
Sclerites of *Melithaea doederleini* sp. n., MZS-Cni61; **a** point spindles **b** collaret spindles **c** tentacle sclerites **d** pharynx rods.

**Figure 30.**
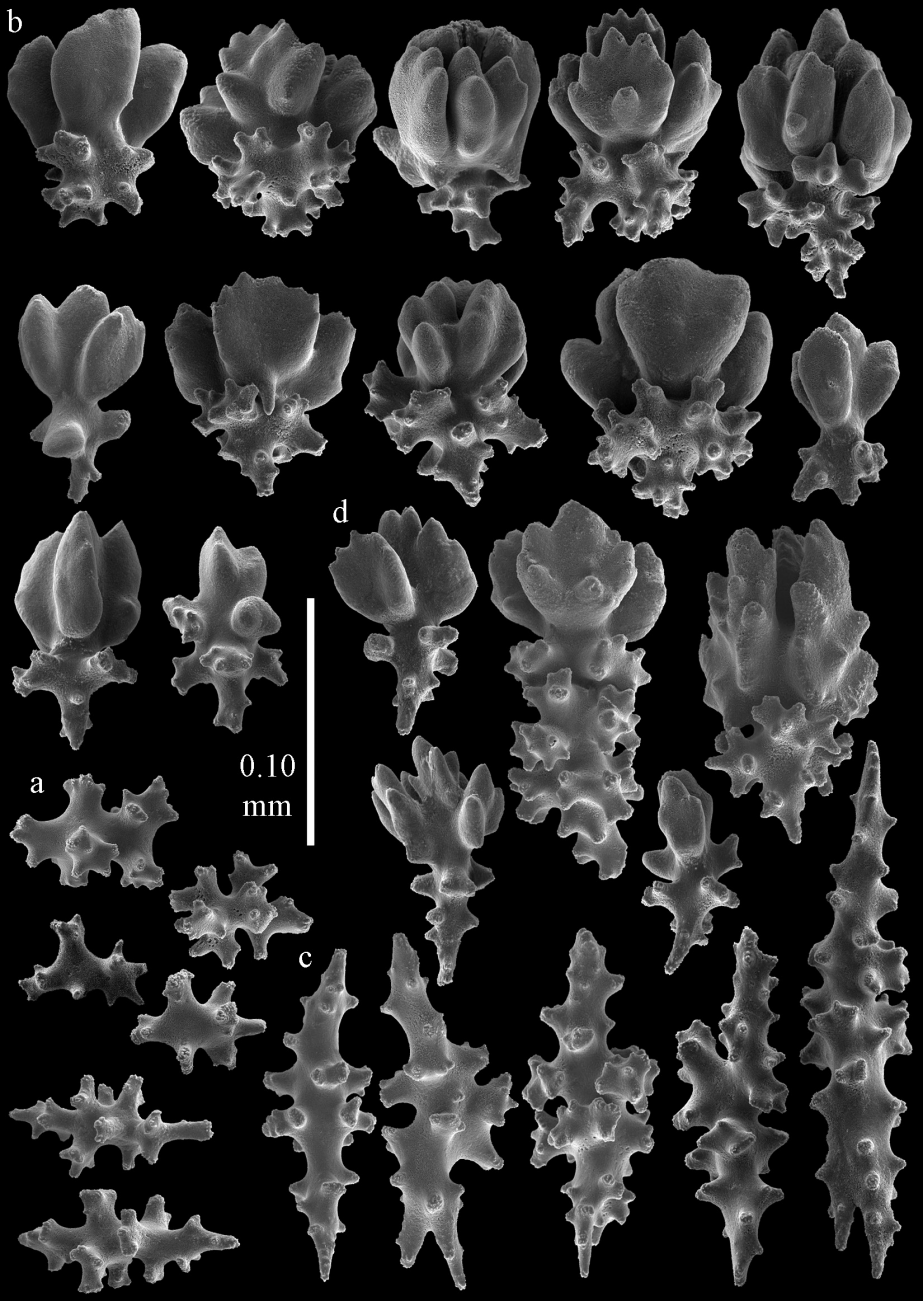
Sclerites of *Melithaea doederleini* sp. n., MZS-Cni61; **a** capstans **b** clubs of coenenchyme **c** spindles of coenenchyme **d** clubs of calyces.

Figures 28a , 29 , 30 , 35

#### Material examined.

Holotype **MZS-Cni61**, Sagami Bay, 60-100 fms (143-183 m), coll. Doederlein, 1882.

#### Description.

Colony broken up, consisting of four fragments (Fig. 28a). Points with slightly bent spindles up to 0.25 mm long, distal end with leaves (Fig. 29a). Collaret with bent spindles up to 0.30 mm long, middle part with more developed tubercles (Fig. 29b). Tentacles with platelets, the larger ones crescent-shaped with irregular projections (Fig. 29c). These platelets are up to 0.15 mm long. Pharynx with straight spiny rods, up to 0.05 mm long (Fig. 29d). Coenenchyme with predominantly capstans (Fig. 30a), and small clubs resembling flower buds (Fig. 30b), up to 0.10 mm long. Spindles, 0.10-0.20 mm long, with simple tubercles, are also present. (Fig. 30c). The calyces with additional clubs, up to 0.15 mm long (Fig. 30d).

#### Color.

Colony fragments red, polyp sclerites light yellow, all others orange.

#### Distribution.

Sagami Bay (Fig. 35).

#### Etymology.

The species is named after the collector, Ludwig H.P. Döderlein.

#### Remarks.

The coenenchymal clubs of this species look like flower buds, similar to those described for *Melitaea retifera* Lamarck, 1916 by Ofwegen et al. (2000), but that species has unilaterally foliate spheroids, a type of sclerite not present in the present material.

### Melithaea frondosa

Taxon classificationAnimaliaAlcyonaceaMelithaeidae

(Brundin, 1896)

**Figure 31.**
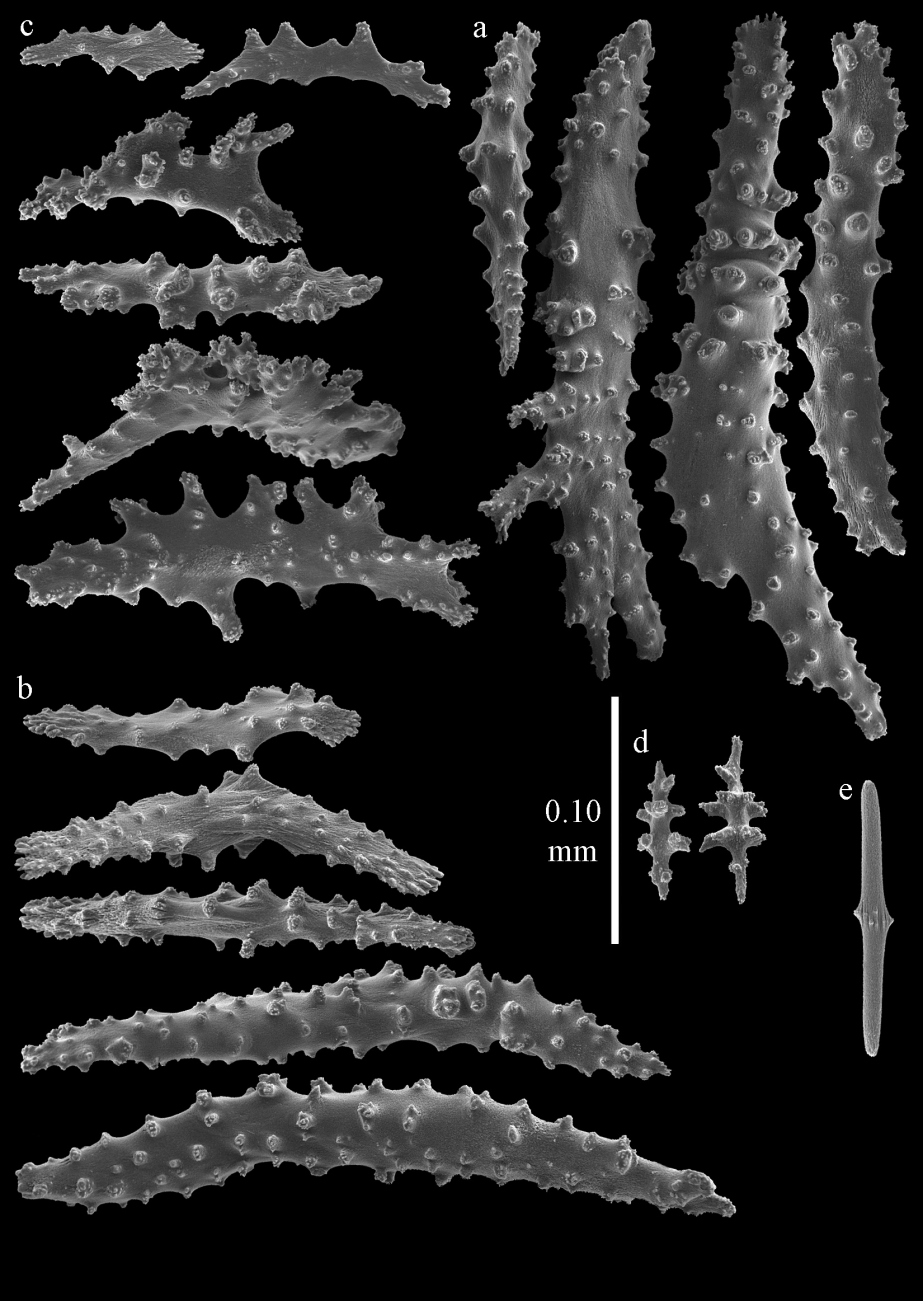
Sclerites of *Melithaea frondosa*, UPSZTY 2164; **a** point spindles **b** collaret spindles **c** tentacle sclerites **d** pharynx rods **e** axial rod.

**Figure 32.**
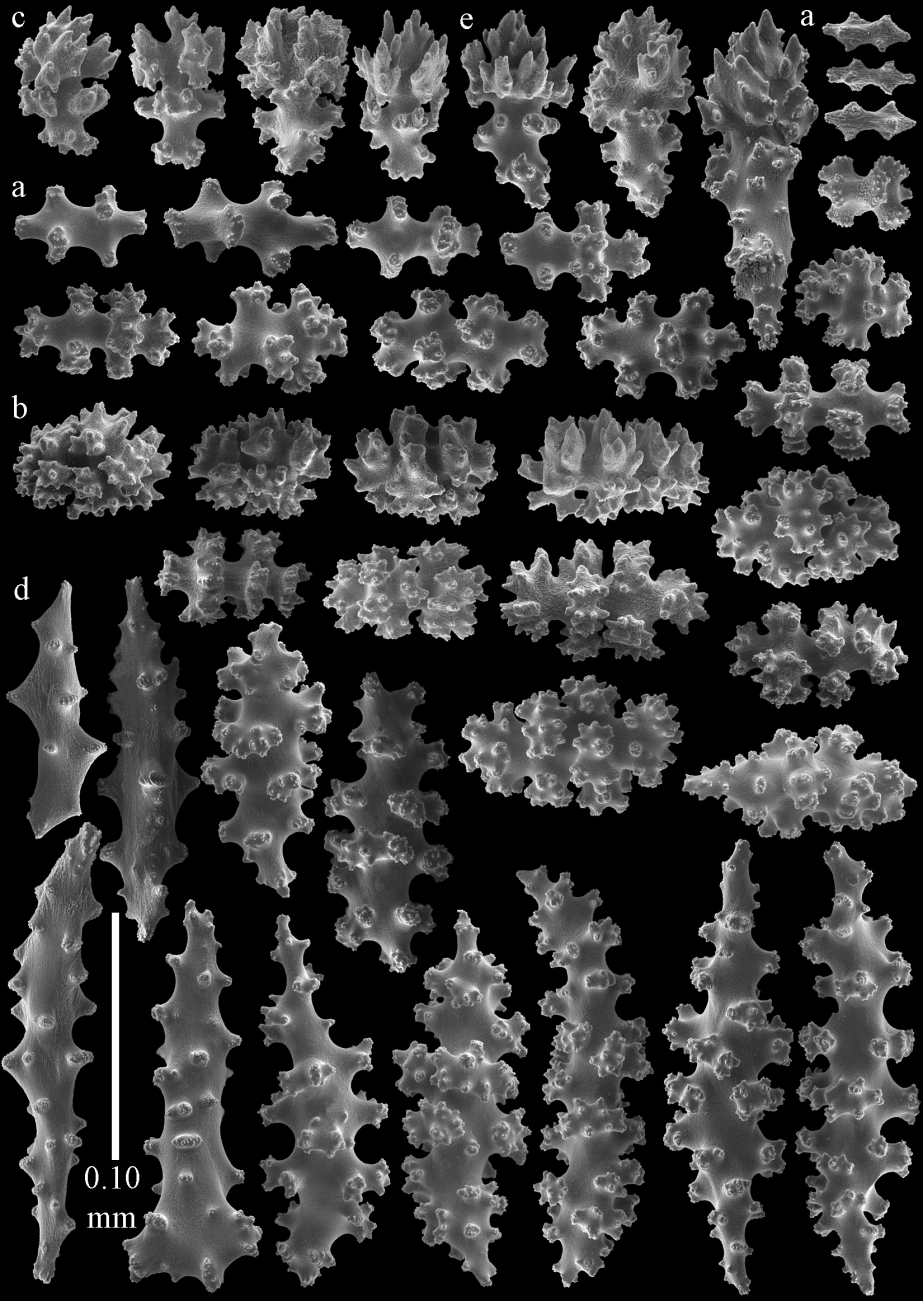
Sclerites of *Melithaea frondosa*, UPSZTY 2164; **a** capstans **b** unilaterally spinose spheroids **c** clubs of coenenchyme **d** spindles of coenenchyme **e** clubs of calyces.

Figures 28b , 31 , 32 , 35

Psilacabaria frondosa : Brundin 1896: 14, pl. 1 fig. 5, pl. 2 fig. 5 (Hirudo Strait (= Hirado Strait), Japan).
Acabaria frondosa : Kükenthal 1909: 61; Kükenthal 1919: 185; 1924: 80; Hickson 1937: 181.

#### Material examined.

Syntypes **UPSZTY 2164** (old number UUZM 67), Hirudo Strait (= Hirado Strait), Nagasaki, Japan, 33°10'N, 129°18'E, coll. Kapt. Suenson.

#### Re-description.

Colonies branched in parallel planes, no anastomoses (Fig. 28b). End branches with bilateral polyp arrangement. Points with slightly bent spindles up to 0.30 mm long, distal end with more developed tubercles (Fig. 31a). Collaret with bent spindles up to 0.30 mm long, middle part with more developed tubercles (Fig. 31b). Tentacles with platelets, the larger ones crescent-shaped with irregular projections (Fig. 31c). These platelets are up to 0.20 mm long. Pharynx with straight spiny rods, up to 0.05 mm long (Fig. 31d). Coenenchyme with capstans (Fig. 32a), unilaterally spinose spheroids (Fig. 32b) 0.05–0.10 mm long; small clubs of similar length (Fig. 32c); spindles 0.10–0.20 mm long (Fig. 32d). The calyces with longer clubs, up to 0.14 mm long (Fig. 32e). Most sclerites have complex tubercles. The axis has smooth and sparsely tuberculate rods (Fig. 31f).

#### Color.

White with colorless sclerites.

#### Distribution.

Only known from Hirado Strait, Nagasaki, East China Sea (Fig. 35).

#### Remarks.

The clubs and spinose spheroids with very spiny heads are characteristic for the species.

### Melithaea habereri

Taxon classificationAnimaliaAlcyonaceaMelithaeidae

(Kükenthal, 1908)

Acabaria habereri : Kükenthal 1908: 197; Kükenthal 1909: 65, figs 70–72, pl. 5 fig. 29 (Sagami Bay, Japan); Kükenthal 1919: 179; Kükenthal 1924: 76.
Acabaria aff. habereri : Aurivillius 1931: 26, fig. 4 (Sagami Bay, 700 m).
Acabaria harbereri [sic]: Hickson 1937: 177.
? Acabaria habereri : Rho et al. 1980: 52 (Korea Strait).

#### Material examined.

None, according to Kükenthal (1908) the material was deposited in München but it was not found there.

#### Re-description

- after Kükenthal (1908): Colony branched in parallel planes, many anastomoses. Points and collaret sclerites 0.18 mm long, point sclerites are spiny clubs. Colony with spindles, in the calyx 0.15-0.18 mm long, in the coenenchyme about 0.18 mm long.

#### Color.

Yellowish orange.

#### Remarks.

According to Kükenthal (1908) this species mostly resembles *Melithaea undulata*. From the description it most resembles *Melithaea corymbosa*. One other Japanese melithaeid shows many anastomoses, namely *Melithaea boninensis* sp. n. For differences see our discussion on that species.

### Melithaea isonoi sp. n.

Taxon classificationAnimaliaAlcyonaceaMelithaeidae

http://zoobank.org/F00B7FCB-6C54-49B6-B340-C3632C6CA7A2

**Figure 33.**
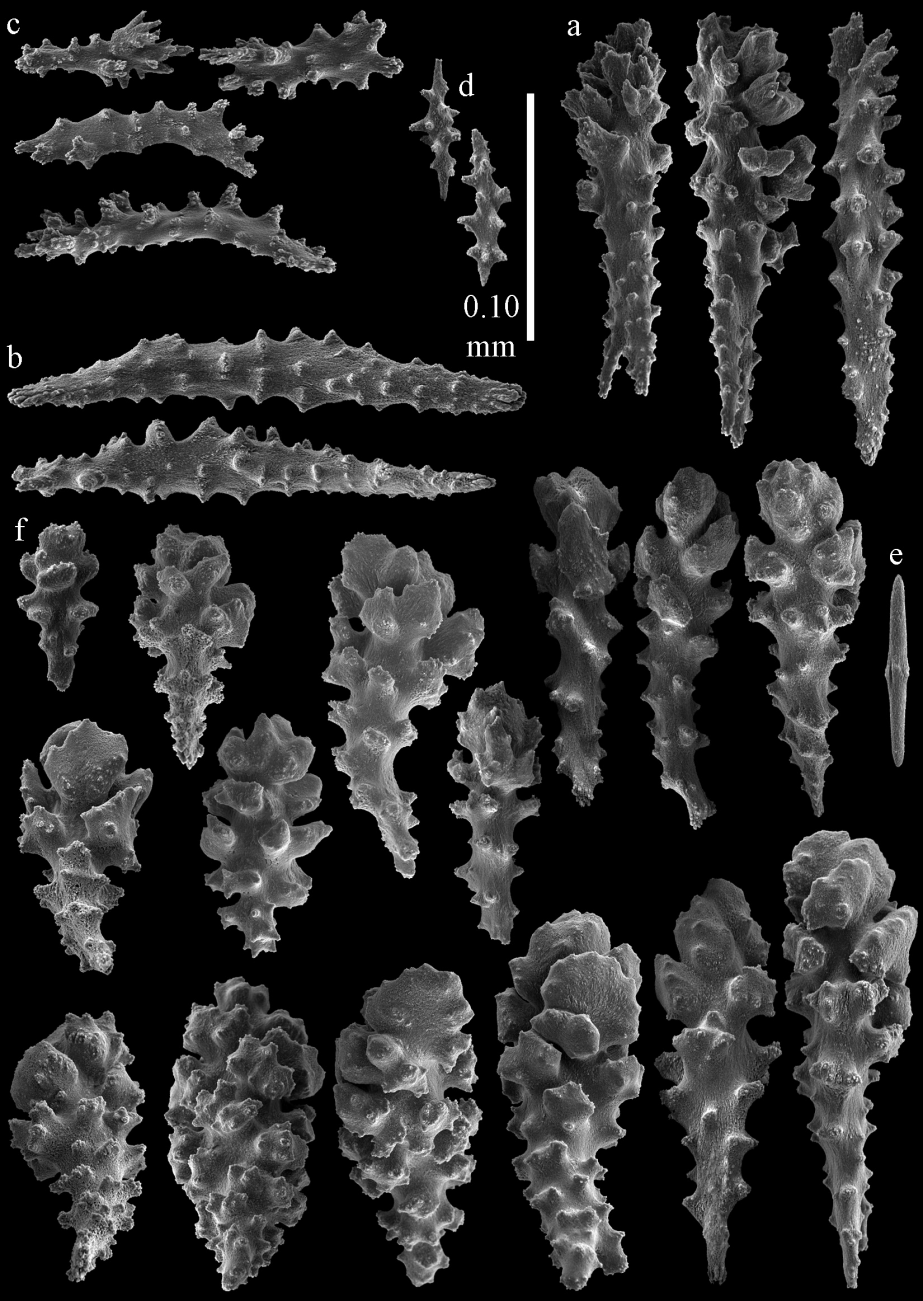
Sclerites of *Melithaea isonoi* sp. n., UMUTZ-CnidG-34; **a** point spindles **b** collaret spindles **c** tentacle sclerites **d** pharynx rods **e** axial rod; f clubs of calyx.

**Figure 34.**
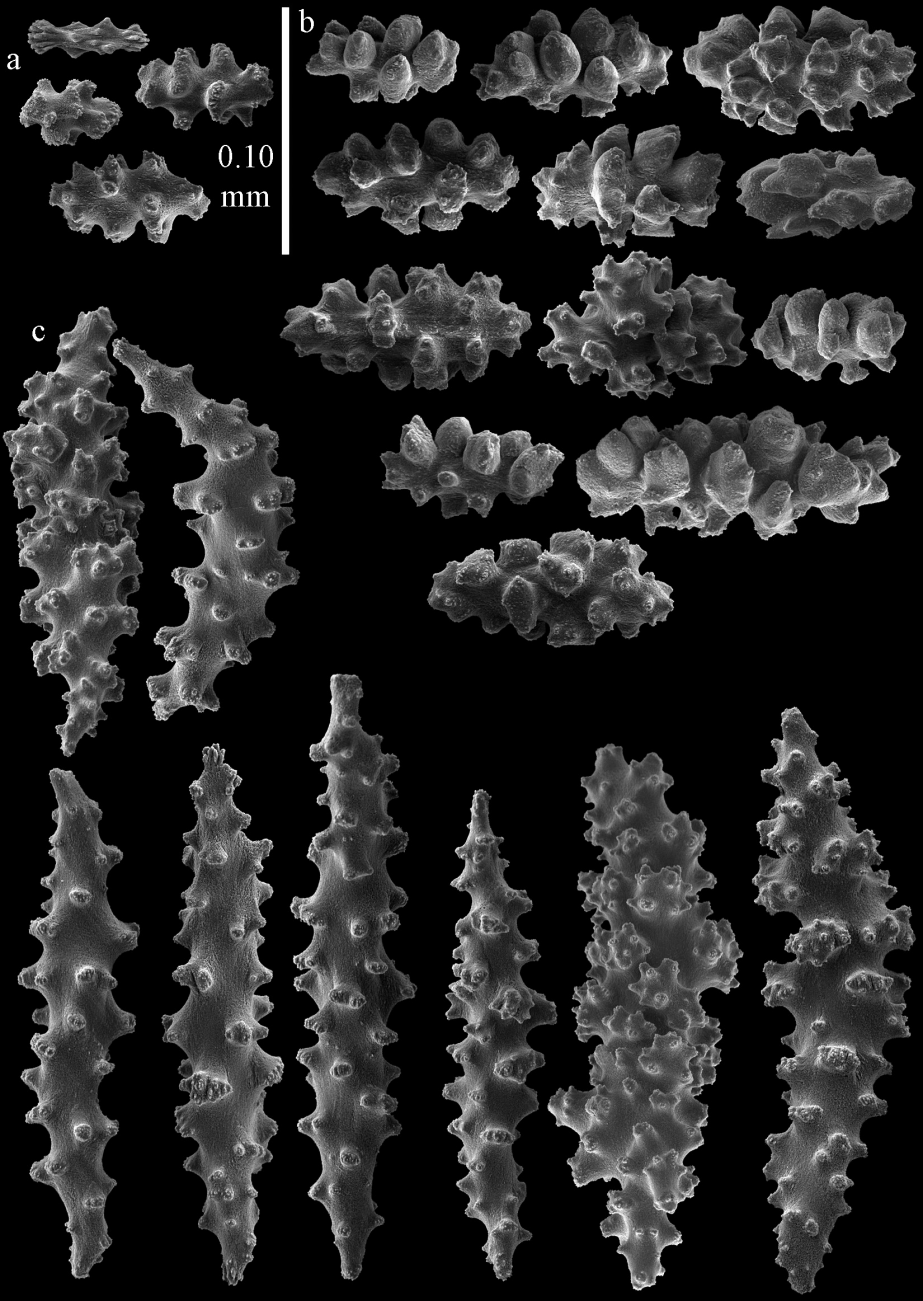
Sclerites of *Melithaea isonoi* sp. n., UMUTZ-CnidG-34; **a** capstans **b** unilaterally spinose spheroids **c** spindles of coenenchyme.

**Figure 35.**
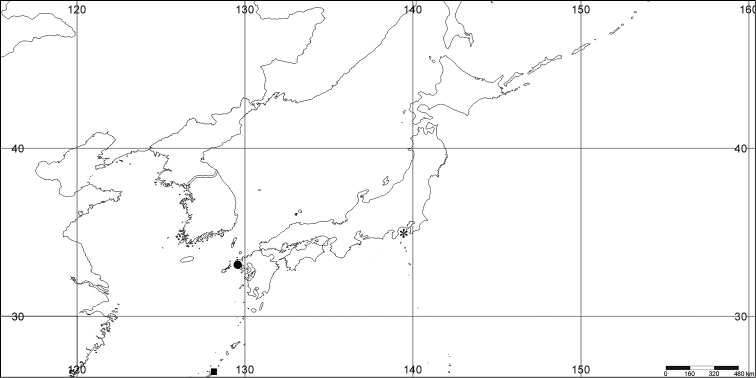
Distribution of *Melithaea doederleini* sp. n. (*), *Melithaea frondosa* (●), and *Melithaea isonoi* sp. n. (■).

Figures 28c , 33 , 34 , 35

?Acabaria sp. A: Aguilar-Hurtado et al. 2012: 63, fig. 7 (Okinawa).

#### Material examined.

Holotype **UMUTZ-CnidG-34**, Coral Reef, Cape Chinen, Okinawa Prefecture, Japan, 15 April 1901; paratype **UMUTZ-CnidG-256**, same data as holotype.

#### Description.

The holotype is 12 cm long and 11 cm wide, branching is in one plane and a holdfast is lacking (Fig. 28c). The stem is 10 mm wide, the end branches only 2 mm wide. The colony has no anastomoses. The polyps are situated biserially on the branches, the calyces are dome-shaped, and the polyps retracted.

Points with slightly bent spindles up to 0.20 mm long, distal end with leaves (Fig. 33a). Collaret with bent spindles up to 0.20 mm long, middle part with more developed tubercles (Fig. 33b). Tentacles with platelets, the larger ones crescent-shaped with irregular projections (Fig. 33c). These platelets are up to 0.10 mm long. Pharynx with straight spiny rods, up to 0.05 mm long (Fig. 33d). Coenenchyme with capstans (Fig. 34a), and unilaterally spinose spheroids, 0.05–0.10 mm long (Fig. 34b). Furthermore spindles are present, 0.10–0.25 mm long (Fig. 34c). All with simple and complex tubercles. The calyces with additional leaf clubs, up to 0.20 mm long (Fig. 33f). The axis has smooth and sparsely tuberculate rods (Fig. 33e).

#### Color.

The colony is orange as are most sclerites; a few are yellow colored.

#### Distribution.

Only known from Okinawa Prefecture (Fig. 35). The material is probably collected during the Ryukyu (= Okinawa) expedition by K. Mitsukuri and I. Ikeda, in April, 1901.

#### Etymology.

The species is named after the late Prof. Naohide Isono who has worked on Japanese zoological history from the Edo to Meiji period, in appreciation of informing the first author about the collectors data in this publication.

#### Remarks.

The species resembles *Melithaea japonica* but differs in having leaf clubs in the calyces. It resembles *Melithaea tenuis* regarding the unilaterally spinose spheroids, but it has longer spindles in the coenenchyme. It could be *Acabaria* sp. A. of Aguilar-Hurtado et al. (2012), but in that case we must accept that these authors did not illustrate the remarkable unilaterally spinose spheroids that we found among the sclerites. The “spines” of these spheroids are very rounded, hardly resembling spines. However, a more appropriate term than spinose spheroids is not available (Bayer et al. 1983). Moreover, all colonies of Aguilar-Hurtado et al. (2012) had anastomoses while *Melithaea isonoi* has none.

### Melithaea japonica

Taxon classificationAnimaliaAlcyonaceaMelithaeidae

(Verrill, 1865)

http://zoobank.org/DFD23E83-138A-48EA-8C34-27BEE76424FB

Figures 36 , 37 , 38 , 39 , 40 , 41 , 42 , 43 , 44 , 45 , 46 , 47 , 48 , 49 , 50 , 51 , 52 , 53 , 54 , 55

Mopsella japonica : Verrill 1865: 190; Verrill 1870: 80 (Simoda (=Shimoda), Japan).
Melitella japonica : Gray 1870: 7.
? Acabaria japonica : Ridley 1884: 361 (Darwin, Australia).
Acabaria japonica (in part): Kükenthal 1919: 188; 1924: 82.
Acabaria japonica : Hickson 1937: 178 (re-examination of type).
Pleurocorallium confusum : Moroff 1902a: 582; Moroff 1902b: 404, pl. 17 fig. 8, pl. 18 fig. 19.
Pleurocoralloides confusum : Kükenthal 1924: 53.
Pleurocoralloides formosum : Moroff 1902a: 583; Moroff 1902b: 406, pl. 17 fig. 10, pl. 18 fig. 20; Kükenthal 1924: 52, fig. 42.
Melitodes flabellifera : Kükenthal 1908: 190; Kükenthal 1909: 54, figs 50–54, pl. 4 fig. 22 (Japan); Kükenthal 1919: 143; 1924: 58; Aurivillius 1931: 23 (Sagami Bay); Hickson 1937: 122.
Melithaea flabellifera var. reticulata : Kükenthal 1908: 191; Kükenthal 1909: 55, pl. 4 fig. 23; Kükenthal 1924: 59.
Melithaea flabellifera var. cylindrata : Kükenthal 1908: 192; Kükenthal 1909: 57, pl. 4 fig. 24; Kükenthal 1924: 59.
Melitodes densa Kükenthal 1908: 192; 1909: 58, figs 59–60, pl. 5 fig. 25 (Japan); 1919: 143; 1924: 58; Hickson 1937: 122.
?Melithaea flabellifera : Rho et al. 1980: 48 (Korea Strait); Song 2000: 111 (Korea Strait, Sea of Japan).
?Melithaea densa : Rho et al. 1980: 50 (Korea Strait); Song 2000: 114 (Korea Strait).

#### Material examined.

Holotype of *Mopsella japonica* **BMNH 1946.1.14.207** dried sclerites of the type MCZ 4265, now MCZ Invertebrate Zoology ALCY-412 (microscopic slide only), Simoda (= Shimoda), Japan, coll. Prof. Hickson; **NHMW 8046** (A.N. 1156), Nagasaki, Japan, I. Erber in Wien (Dr. A. v. Roretz) (possibly collected between ca.1875–1879); **NHMW 8047**, Enoshima, Japan, coll. Dr. Richard. v. Drasche; **NHMW 12690** (A.N. 4837), Enoshima, Japan, Baron Eug. V. Ransonnet, Ostasiatisches Ex. (1873); *Pleurocoralloides formosum*, **ZSM 20051735**, type, Japan, Sagami Bay, leg. Haberer, 1901; *Melitodes flabellifera*: **ZMB 5822**, syntype, Japan, up to 20 m depth, coll. Doflein, 1904/05; **NHMW 2426**, Enoshima, Nagoya, coll. Drasche, Koerbl, 18–19 December 1877; **MZS-Cni52**, Sagami Bay, coll. Doederlein, 1882; **BMNH 1936.7.6.7**, Sagami Channel, Sagami Bay, purchased of Shibayama Nat. Sci. Laboratory Cat. No. 7c-7A, 7 August 1931; *Melitodes densa*: **ZMB 5801**, syntype, Sagami Bay, 60–250 m?, coll. Doflein 1904/05; **ZMB 5809**, syntype, Sagami Bay, littoral, coll. Doflein1904/05; previously unidentified museum material: **BMNH 1883.8.29.10**, Enoshima, Japan, coll. Res. by D?.F.J. Burge; **MZS-Cni 235**, Jogashima, Sagami Bay, Japan, coll. Doederlein; **ZMUC ANT-000591**, Misaki, Sagami Bay, Japan, 1–2 fms (2–4 m), coll. Dr. Th. Mortensen, 24 June 1914; **UMUTZ-CnidG- 257 (G-23b)**, gorgonia cave at Koajiro, Misaki, Sagami Bay, 16 July 1897; **UMUTZ-CnidG-258 (G-23c)**, same data as UMUTZ-CnidG-257 (G-23b); **UMUTZ-CnidG-259 (G-23d)**, same data as UMUTZ-CnidG-257 (G-23b); **UMUTZ-CnidG-35**, Misaki, Sagami Bay, Japan sp. no. 19; **UMUTZ-CnidG-260 (G-35b)**, same data as UMUTZ-CnidG-35; **UMUTZ-CnidG-261 (G-35c)**, same data as UMUTZ-CnidG-35; **UMUTZ-CnidG-262 (G-35d)**, same data as UMUT**Z-CnidG**-35; **UMUTZ-CnidG-36**, Misaki, Sagami Bay, Japan; **UMUTZ-CnidG-263 (G-36b)**, same data as UMUT**Z-CnidG**-36; **UMUTZ-CnidG-264 (G-36c)**, same data as UMUT**Z-CnidG**-36; **UMUTZ-CnidG-265 (G-36d)**, same data as UMUT**Z-CnidG**-36; **UMUTZ-CnidG-37**, Misaki, Sagami Bay, Japan, coll. K. Kinoshita, summer 1906; **UMUTZ-CnidG-266 (G-37b)**, same data as UMUTZ-CnidG-37; **UMUTZ-CnidG-267 (G-37c)**, same data as UMUT**Z-CnidG**-37; **UMUTZ-CnidG-269 (G-37e)**, same data as UMUTZ-CnidG-37; **UMUTZ-CnidG-38**, Cape Makurazaki, Kagoshima Prefecture, coll. M. Miyajima by diving, 7 August 1899; **UMUTZ-CnidG-42**, Misaki, Sagami Bay, Japan, 1–5 April 1917; **UMUTZ-CnidG-272 (G-42b)**, same data as UMUTZ-CnidG-42; **UMUTZ-CnidG-43**, Misaki, Sagami Bay, Japasp. n. no. 77; **UMUTZ-CnidG-273 (G-43b)**, same data as UMUT**Z-CnidG**-43; **UMUTZ-CnidG-274 (G-43c)**, same data as UMUTZ-CnidG-43; **UMUTZ-CnidG-275 (G-43d)**, same data as UMUTZ-CnidG-43; **UMUTZ-CnidG-276 (G-43e)**, same data as UMUTZ-CnidG-43; **UMUTZ-CnidG-44**, Shimoda Harbour, Izu Peninsula, Japan, vessel Ohnoura-maru cruise, coll. S. Hirota, 28 August 1893; **UMUTZ-CnidG-277 (G-44b)**, same data as UMUTZ-CnidG-44; **UMUTZ-CnidG-278 (G-44c)**, same data as UMUTZ-CnidG-44; **UMUTZ-CnidG-116**, near Misaki Marine biological Station, Sagami Bay, Japan, coll. I. Ijima by diving, 1913; **UMUTZ-CnidG-192**, Moroiso, Misaki, Sagami Bay, Japan, collected by diving, 12 August 1904; **UMUTZ-CnidG-196**, Shimo-Chikura, Kagoshima Prefecture, Japan, coll. M. Miyajima by coral net, 12 July 1899; **AKM 1633**, Kominato, Japan, April 1944, deposited in Aikappu Museum of Natural History, Akkeshi Marine Station, Field Science Center for Northern Biosphere Hokkaido University; **BIK-G224**, Saba-shima Is. Koga Bay, Oga Peninsula, Sea of Japan, 7 m, coll. Y. Sato. 14 July 1988; **BIK-G226**, same data as BIK-G224; **RMNH Coel. 41914 (AKM 594)**, Entrance of Otsuchi Bay, Otsuchi, Iwate Prefecture, 39°21.858'N, 141°59.972'E, 65.6 m, *R/V Yayoi*, coll. A.K. Matsumoto, 12 September 2005; **RMNH Coel. 41915 (AKM 614)**, same data as RMNH Coel.41914; **RMNH Coel. 41916 (AKM 615)**, Entrance of Otsuchi Bay, Iwate Prefecture, 39°21.917'N, 142°00.031'E, 77.6 m, *R/V Yayoi*, St.1(=St.2) 1 m biological dredge, coll. A.K. Matsumoto, 12 September 2005; **RMNH Coel. 41917 (AKM 618)**, same data as RMNH Coel.41916; **RMNH Coel. 41918 (AKM 619)**, off Ohako-zaki Cape, Otsuchi Bay, Iwate Prefecture, 39°21.428'N, 142°00.520'E, 69.2 m, *R/V Yayoi*, St.2(=St.5), 1 m biological dredge, coll. A.K. Matsumoto, 12 September 2005; **RMNH Coel. 41919 (AKM 622)**, same data as RMNH Coel.41918; **RMNH Coel. 41920 (AKM 1200)**, off Ohako-zaki Cape, Otsuchi Bay, Iwate Prefecture, ca. 39°21'N, 142°00'E, ca. 90 m, local fishery boat *Taku-maru*, gill-net, coll. K. Morita, ca. 21 March 2008; **RMNH Coel. 41921 (AKM 1201)**, same data as RMNH Coel.41920; **RMNH Coel. 41922 (AKM 1204)**, off Ohako-zaki Cape, Otsuchi Bay, Iwate Prefecture, ca. 39°21'N, 142°00'E, ca. 90 m, local fishery boat *Taku-maru*, gill-net, coll. K. Morita, 2 May. 2008; **RMNH Coel. 41923 (AKM 1252)**, off Ohako-zaki Cape, Otsuchi Bay, Iwate Prefecture, ca. 39°21'N, 142°00'E, ca.75 m, local fishery boat *Taku-maru*, gill-net, coll. K. Morita, 9 May 2008; **RMNH Coel. 41924 (AKM 1526)**, off Oshima Is. Entrance of Otsuchi Bay and Funakoshi Bay, Iwate Prefecture, 39°22.085'N, 142°01.152'E, 97 m, *R/V Yayoi*, 1 m biological dredge, coll. A.K. Matsumoto, 26 April 2010.

#### Re-description.

Colony bushy with few anastomoses. Branches flattened in the plane of branching and polyps arranged bilaterally. Points with slightly bent spindles up to 0.20 mm long, distal end with more developed tubercles (Fig. 38a). Collaret with bent spindles up to 0.25 mm long, middle part with more developed tubercles. Tentacles with platelets, the larger ones crescent-shaped with irregular projections (Fig. 38b). These platelets are up to 0.10 mm long. Pharynx with straight spiny rods, up to 0.05 mm long. Coenenchyme with capstans (Fig. 38c), 0.07–0.08 mm long and small clubs of similar length (Fig. 38d). Furthermore spindles (Fig. 38e), unilaterally spinose spindles (Fig. 38f) and unilaterally spinose spheroids (Fig. 38g) are present, 0.10–0.30 mm long. All with simple and complex tubercles. The calyces with additional clubs, up to 0.20 mm long (Fig. 38h). The axis has smooth and sparsely tuberculate rods (Fig. 38i).

**Figure 36.**
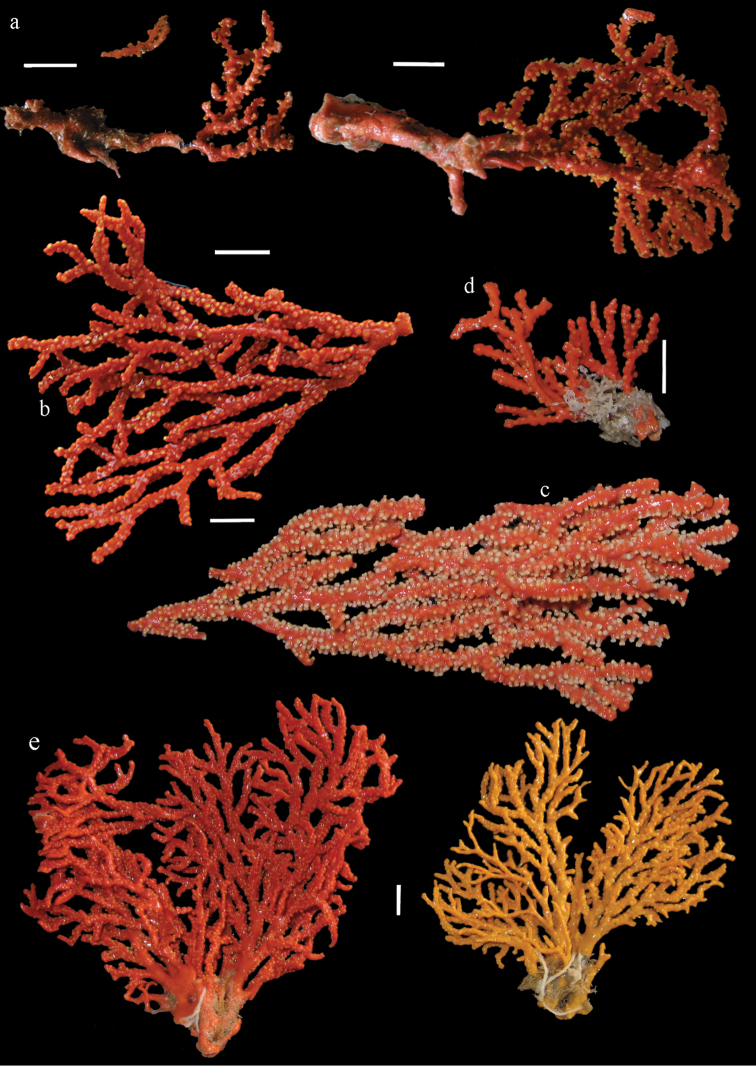
*Melithaea japonica*; **a** *Pleurocoralloides formosum*, ZSM 20051735 **b** *Melitodes flabellifera* ZMB 5822 **c** *Melitodes densa*, ZMB 5801 **d** *Melitodes densa*, ZMB 5809 **e** *Melitodes falbellifera*, NHMW 2426.

**Figure 37.**
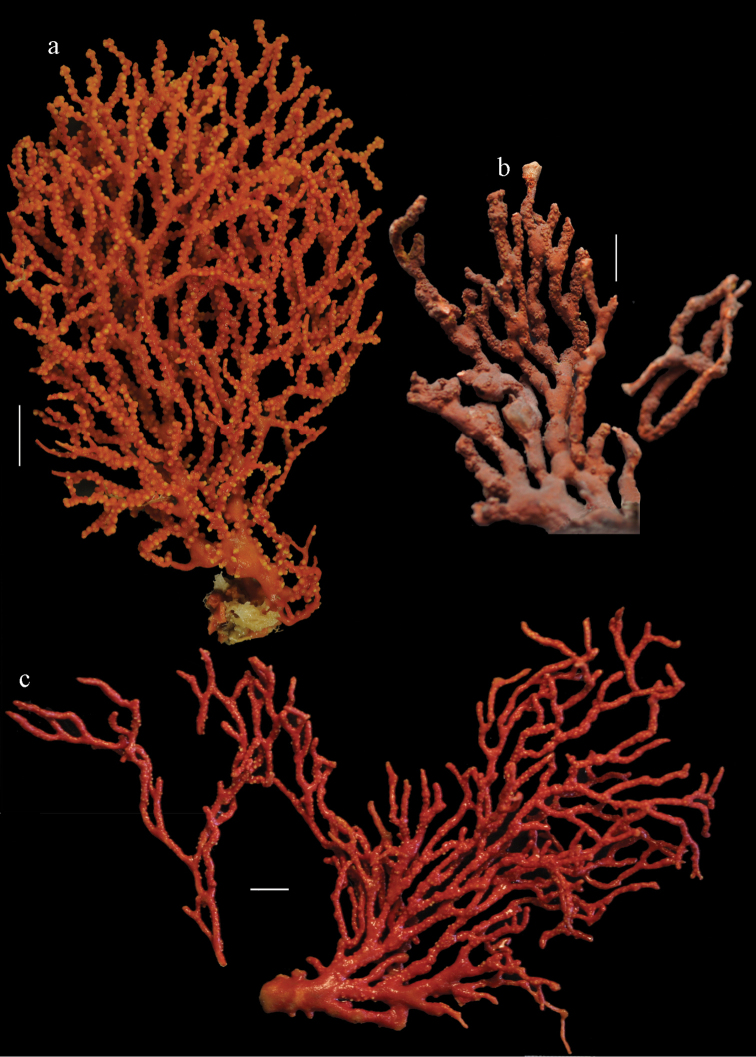
*Melithaea japonica*; **a** BIK-G 224 **b** NHMW 8047 **c** RMNH Coel. 41922.

**Figure 38.**
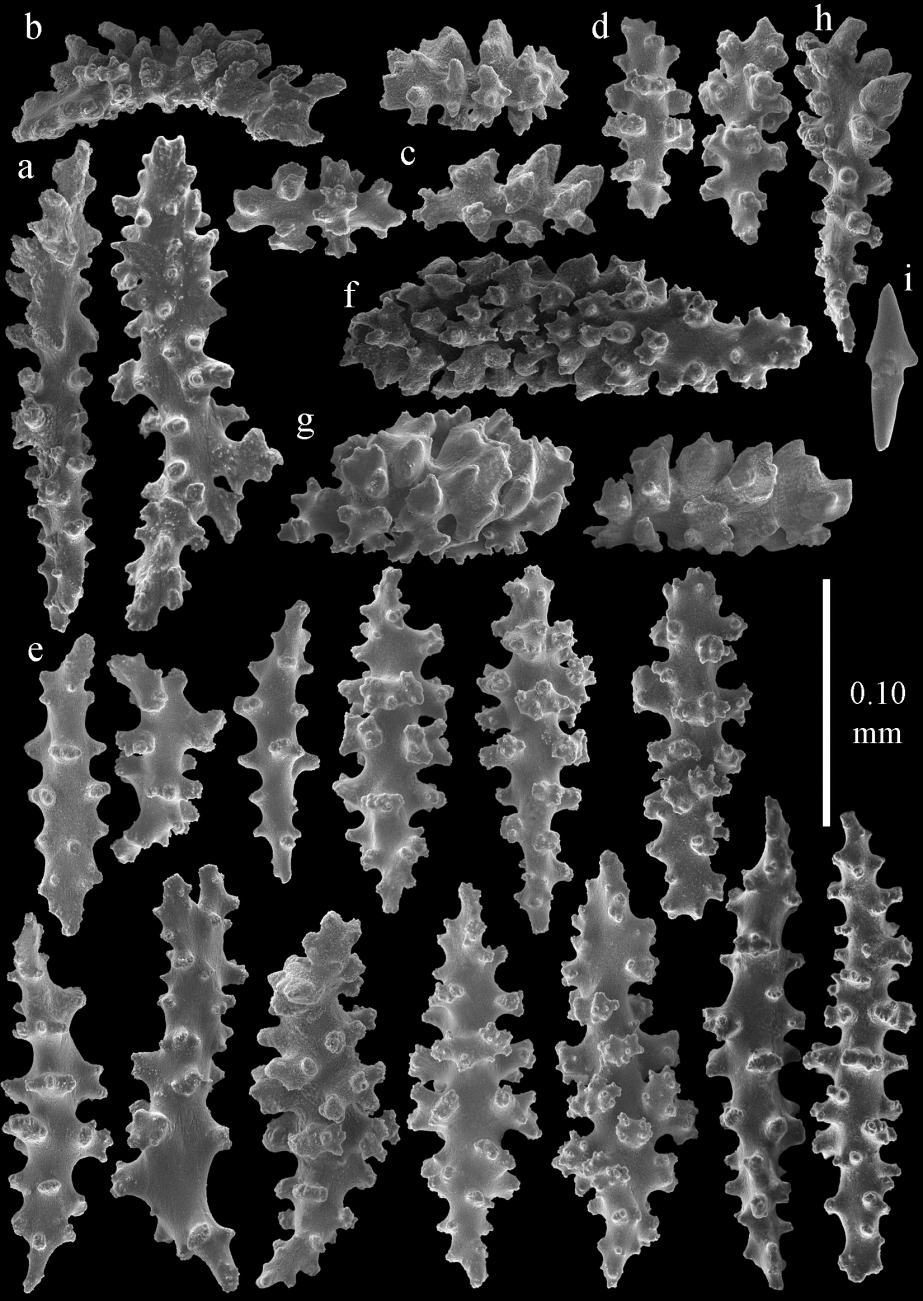
Sclerites of *Melithaea japonica*, BMNH 1946.1.14.207; **a** point spindles **b** tentacle sclerite **c** capstans **d** clubs of coenenchyme **e** spindles **f** unilateraly spinose spindle **g** unilaterally spinose spheroids **h** club of calyx; **i**, axial rod.

#### Color.

Red with yellow polyps. Variation: red (most colonies), pink, with yellow or white polyps, tentacle and pharynx sclerites colorless, all others orange; or colonies yellow with all sclerites yellow; or white with yellow polyps with polyp sclerites yellow and all others colorless. RMNH Coel. 41923 is rather unique in having a pale light brown colony, tentacle sclerites colorless, others colorless, partly white and partly yellow, or entirely yellow.

#### Distribution.

*Melithaea japonica* is found at the eastern Pacific side of Japan; Sagami Bay, Izu Peninsula, Boso Peninsula, Nagasaki (Kyushu Is.), Shimo-Chikura (Kagoshima Prefecture, Kyushu Is.), Cape Makurazaki (Kagoshima Prefecture, Kyushu Is.), Otsuchi Bay (Sanriku, Iwate Prefecture); and at the western Sea of Japan side; Oga peninsula(Akita Prefecture) (Fig. 55).

#### Remarks.

**Figure 39.**
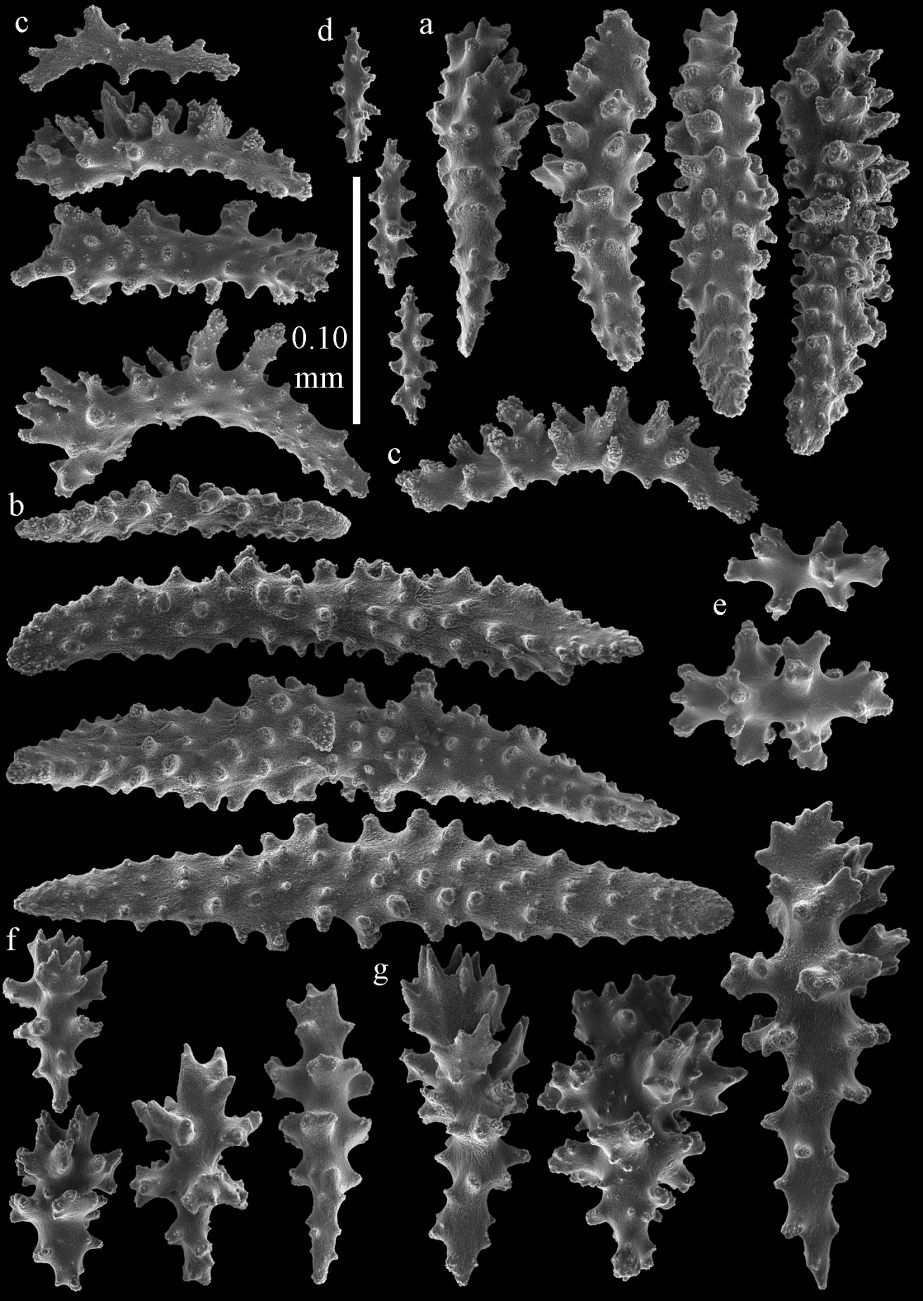
Sclerites of *Melithaea japonica*, NHMW 8047; **a** point spindle **b** collaret spindles **c** tentacle sclerites **d** pharynx rods **e** capstans **f** clubs of coenenchyme **g** clubs of calyx.

**Figure 40.**
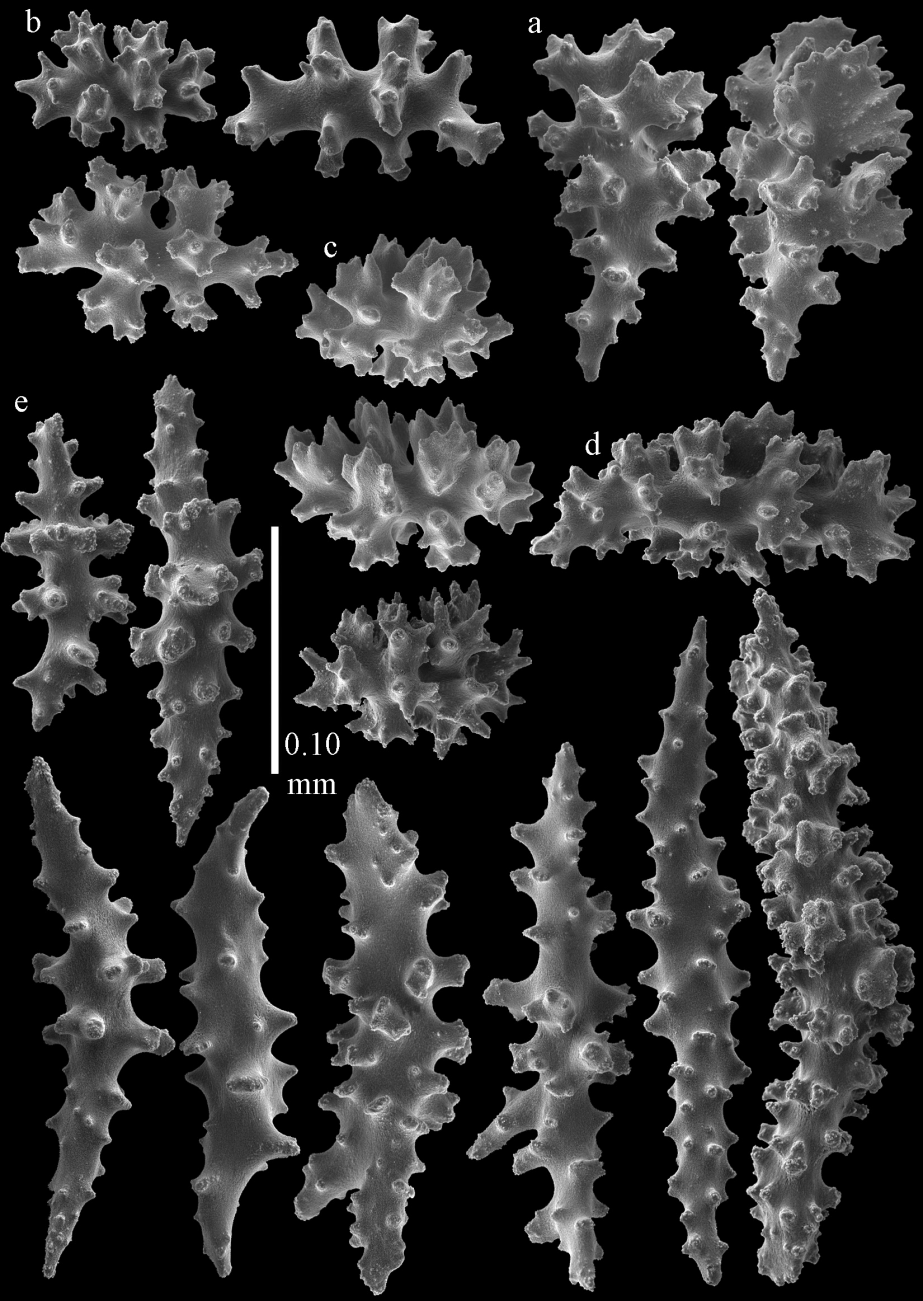
Sclerites of *Melithaea japonica*, NHMW 8047; **a** clubs of calyx **b** capstans **c** unilaterally spinose spheroids **d** unilaterally spinose spindle **e** spindles.

The examined type material of *Mopsella japonica* was fragmented. In Harvard we only found one microscope slide (MCZ 4265, ALCY-412), in London only dried sclerites of the Harvard material (BMNH 1946.1.14.207). The type colony seems to be lost. Only in Vienna we found complete specimens identified as *Melithaea japonica*; NHMW 8046, NHMW 8047 (Fig. 37b) and NHMW12690 identified as *Melithaea japonica*, the sclerites resemble those of the type material (Figs 39, 40). The collector of NHMW 8046, Dr. Albrecht von Roretz visited Japan as a medical attaché of Austria (= Hungarian legation) between 1875–1879 and possibly collected this material. NHMW 2426 (*Melithaea flabellifera*) and NHMW 8047 were collected by Dr. Richard. v. Drasche. He visited Japan during the Far East expedition 1875–1876. The Ostasiatisches Expedition by Baron Eug. V. Ransonnet, during which NHMW 12690 was collected, happened in 1873 (Matsumoto 2013, submitted).

**Figure 41.**
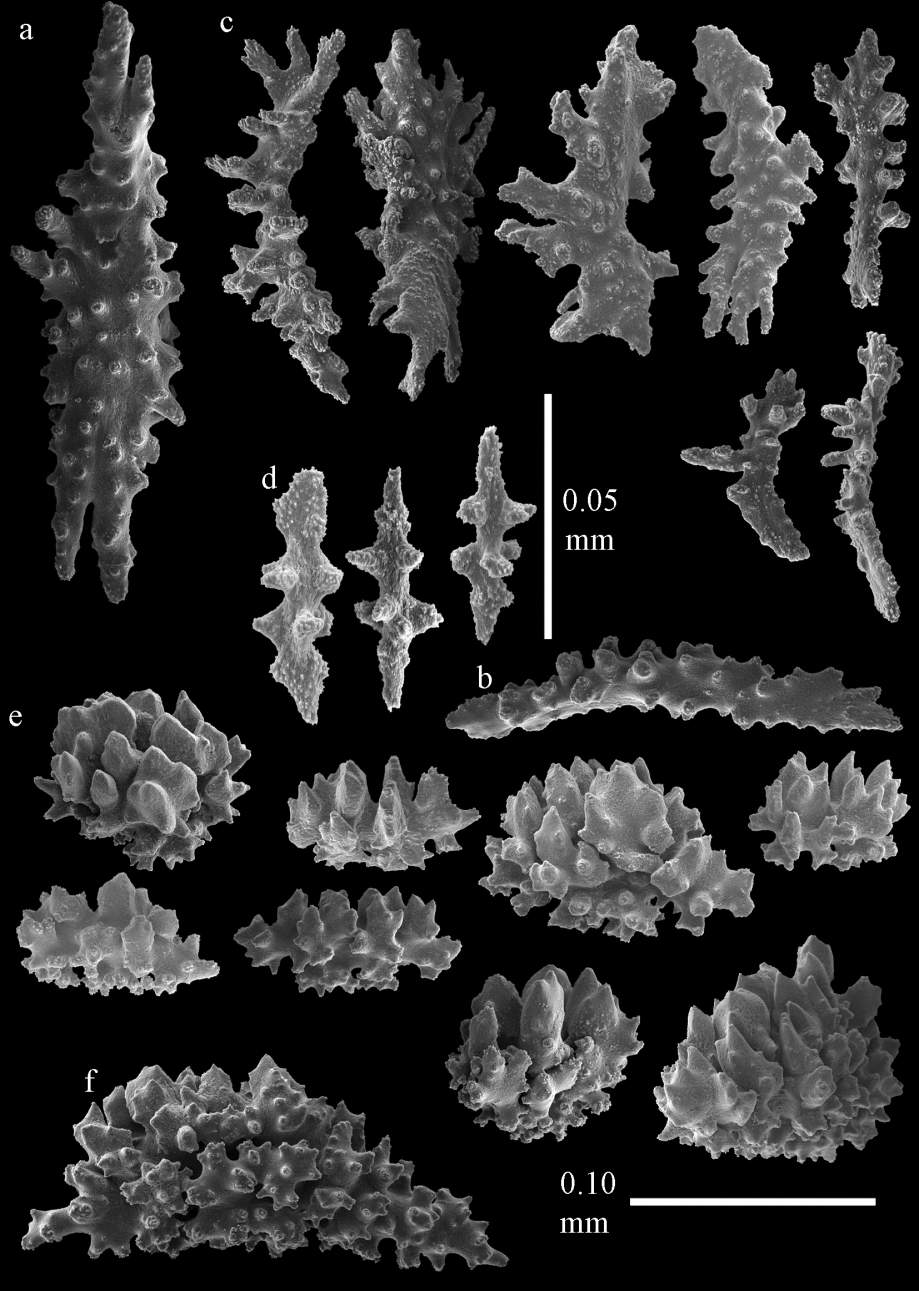
Sclerites of *Melitodes flabellifera*, ZMB 5822; **a** point spindle **b** collaret spindle **c** tentacle sclerites **d** pharynx rods **e** unilaterally spinose spheroids **f** unilaterally spinose spindle.

**Figure 42.**
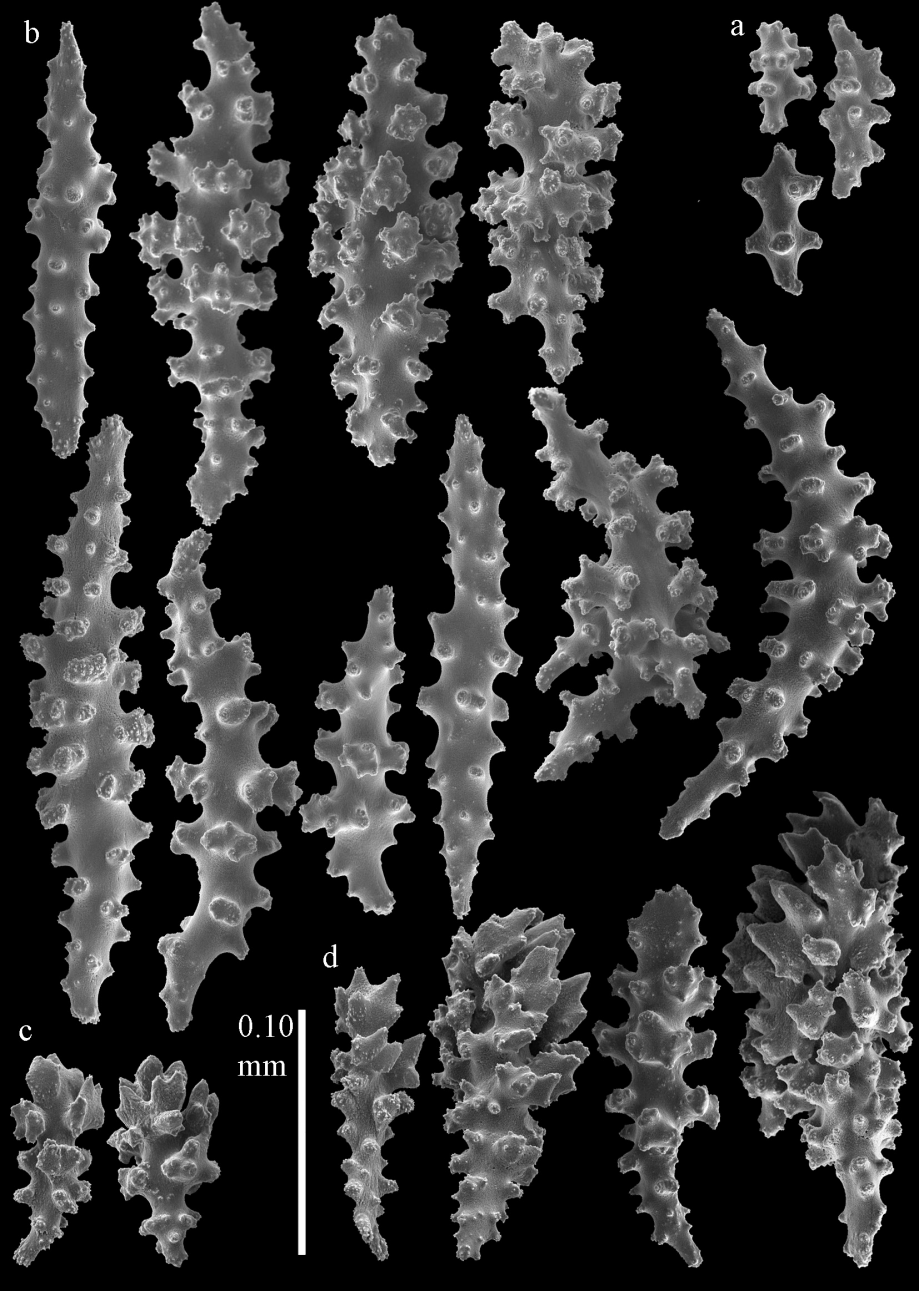
Sclerites of *Melitodes flabellifera*, ZMB 5822; **a** capstans **b** spindles **c** clubs of coenenchyme **d** clubs of calyx.

**Figure 43.**
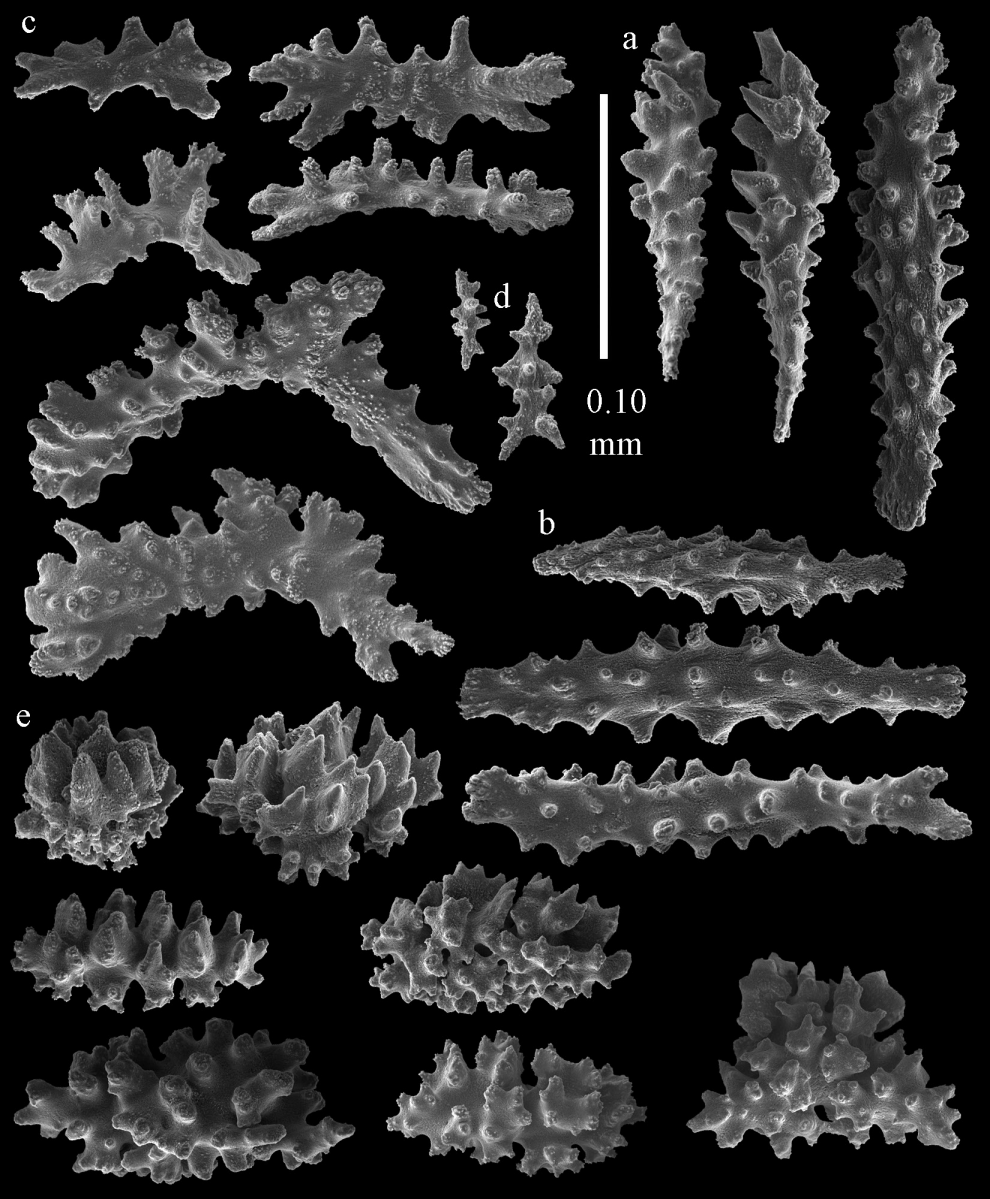
Sclerites of *Melitodes flabellifera*, NHMW 2426; **a** point spindles **b** collaret spindles **c** tentacle sclerites **d** pharynx rods **e** unilaterally spinose spheroids.

**Figure 44.**
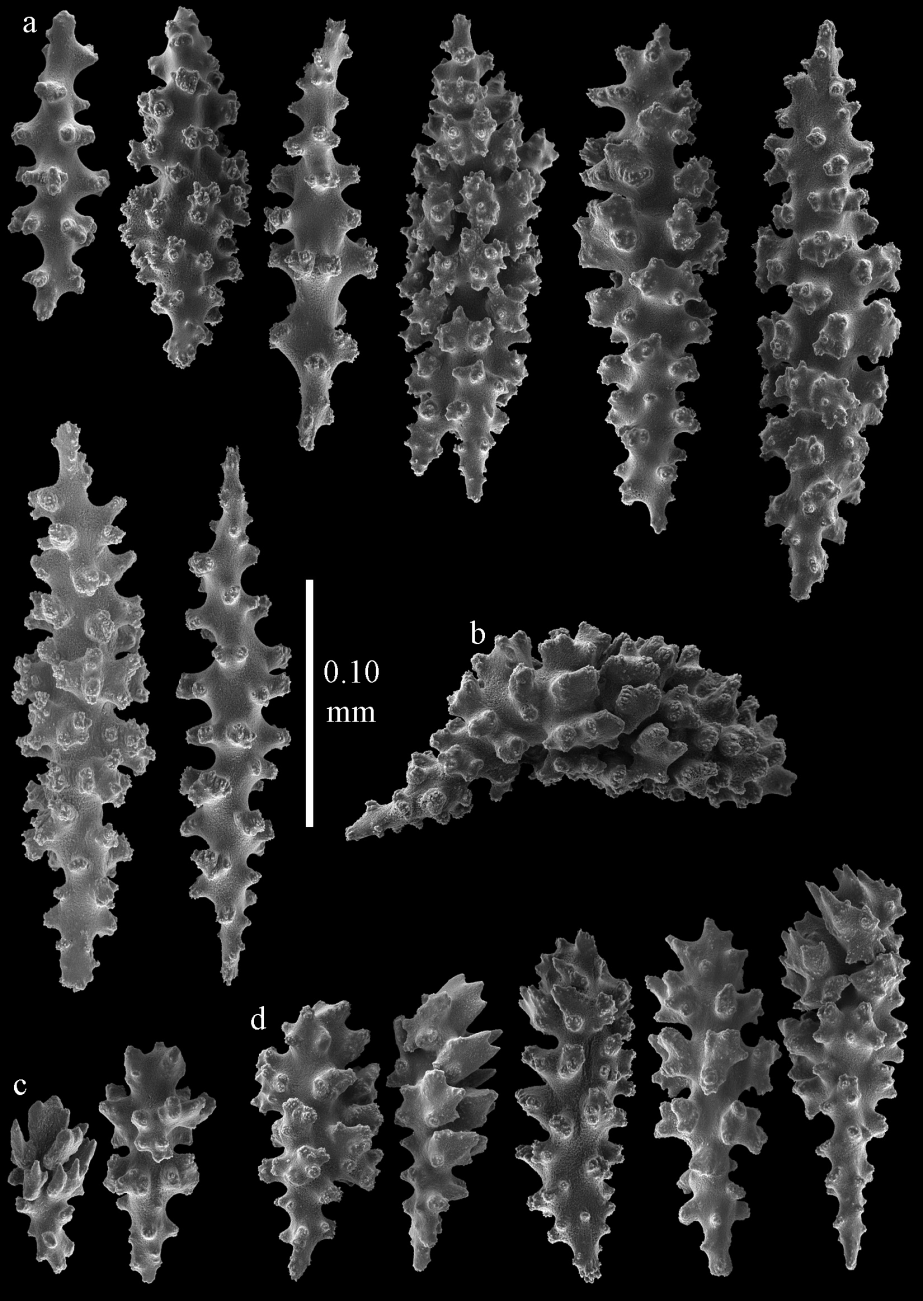
Sclerites of *Melitodes flabellifera*, NHMW 2426; **a** spindles **b** unilaterally spinose spindle **c** clubs of coenenchyme **d** clubs of calyx.

Despite the small remainder of the type we could link it with other species described from Sagami Bay. Apparently this is the most common shallow-water species of the region, Kükenthal (1908) mentioned 40 specimens for his *Melitodes flabellifera*. We examined ZMB 5822 (Figs 36b, 41, 42) and NHMW 2426 (Figs 36e, 43, 44). They both are similar to *Melithaea japonica*.

**Figure 45.**
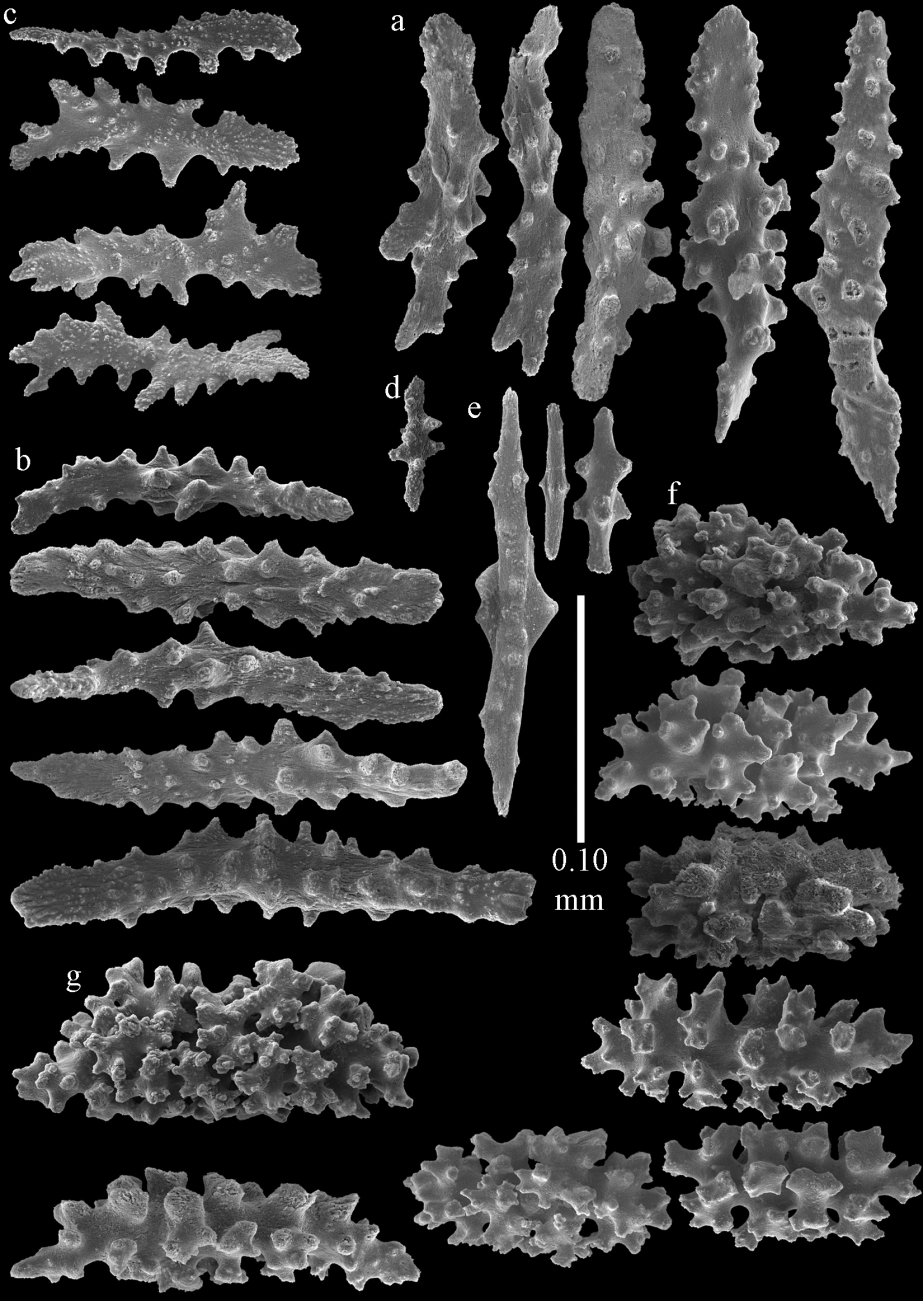
Sclerites of *Melitodes densa*, ZMB 5801; **a** point spindles **b** collaret spindles **c** tentacle sclerites **d** pharynx rods **e** axial rods **f** unilaterally spinose spheroids **g** unilaterally spinose spindles.

**Figure 46.**
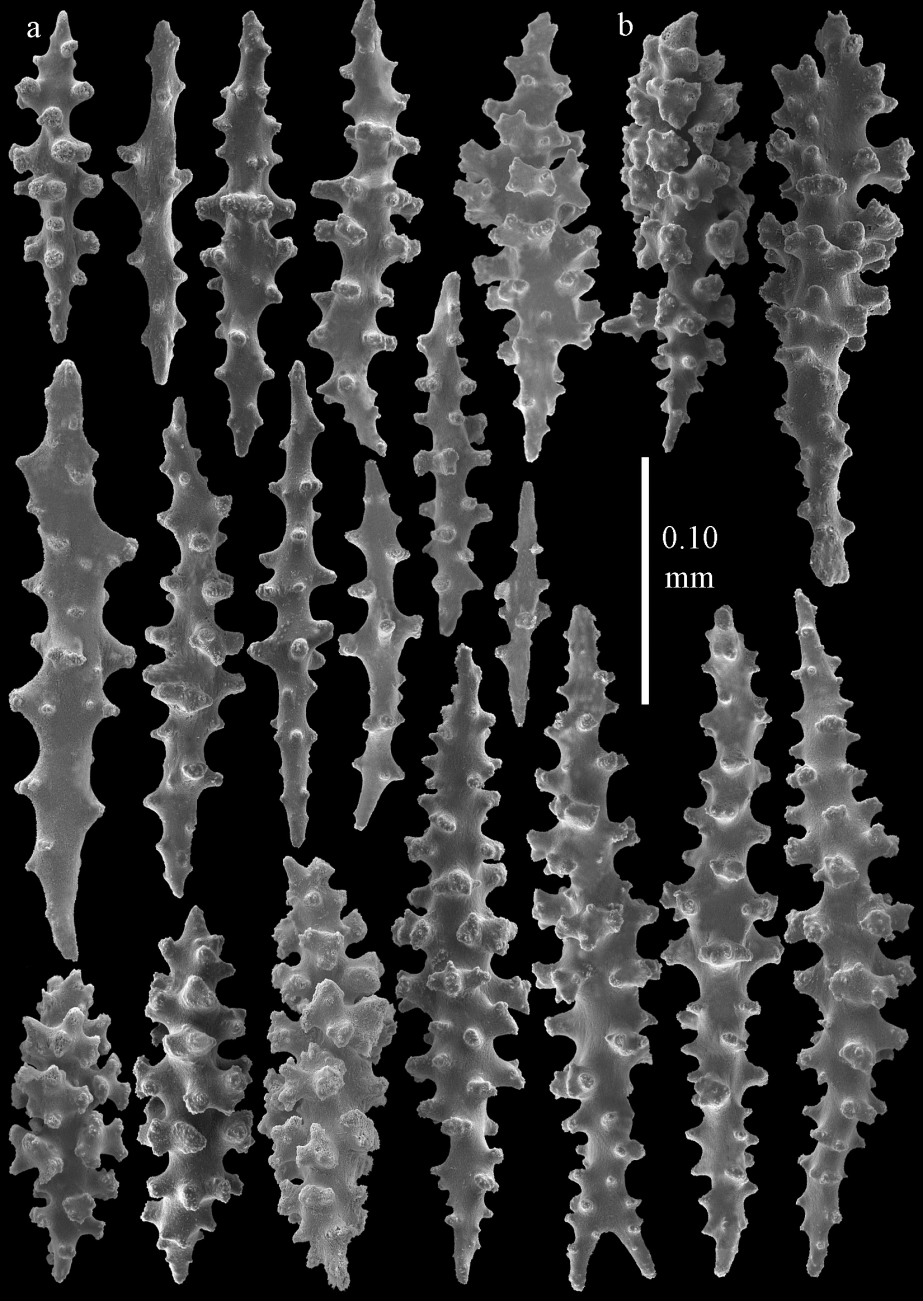
Sclerites of *Melitodes densa*, ZMB 5801; **a** spindles of coenenchyme **b** clubs of calyx.

**Figure 47.**
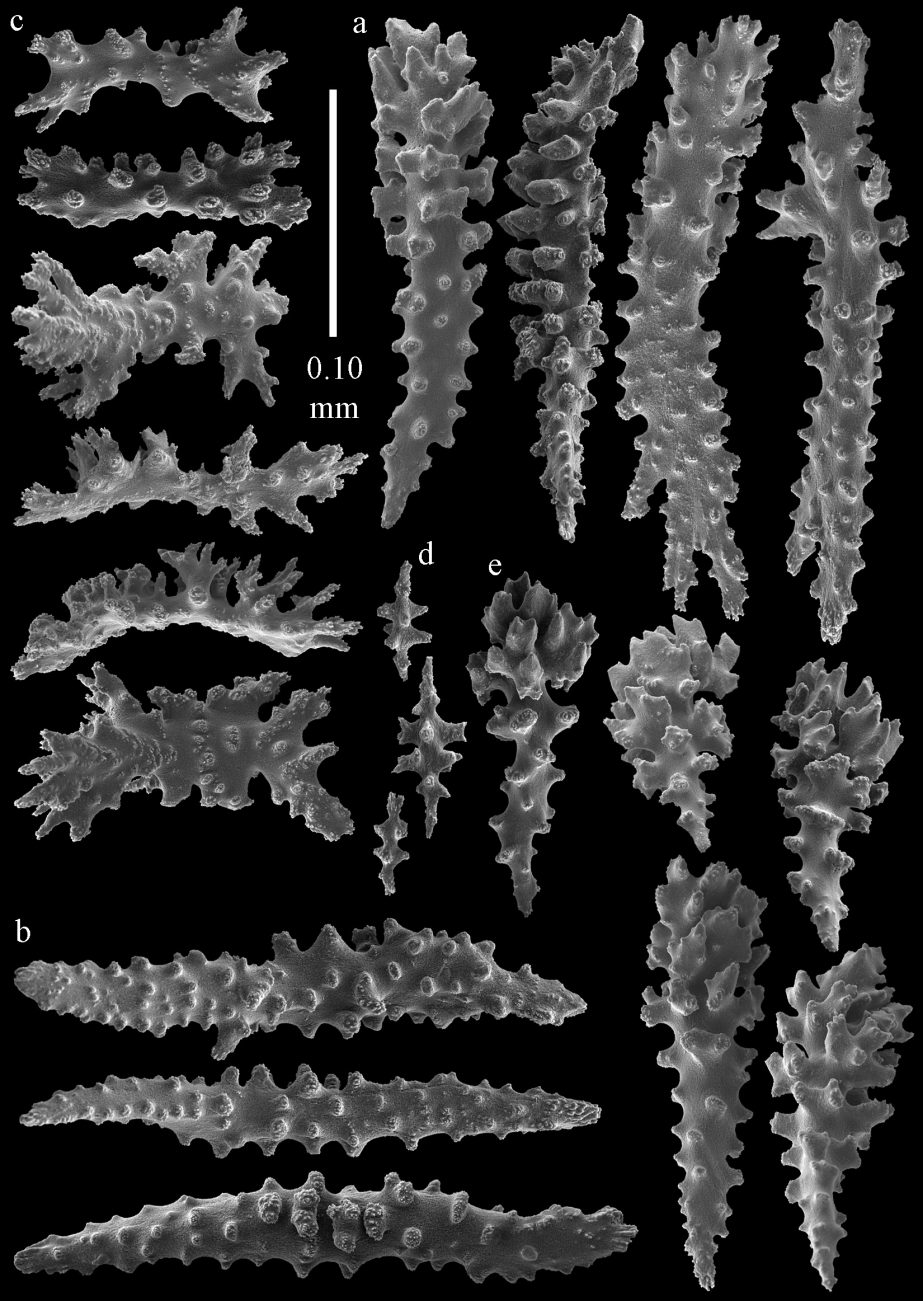
Sclerites of *Melitodes densa*, ZMB 5809; **a** point spindles **b** collaret spindles **c** tentacle sclerites **d** pharynx rod **e** clubs of calyx.

**Figure 48.**
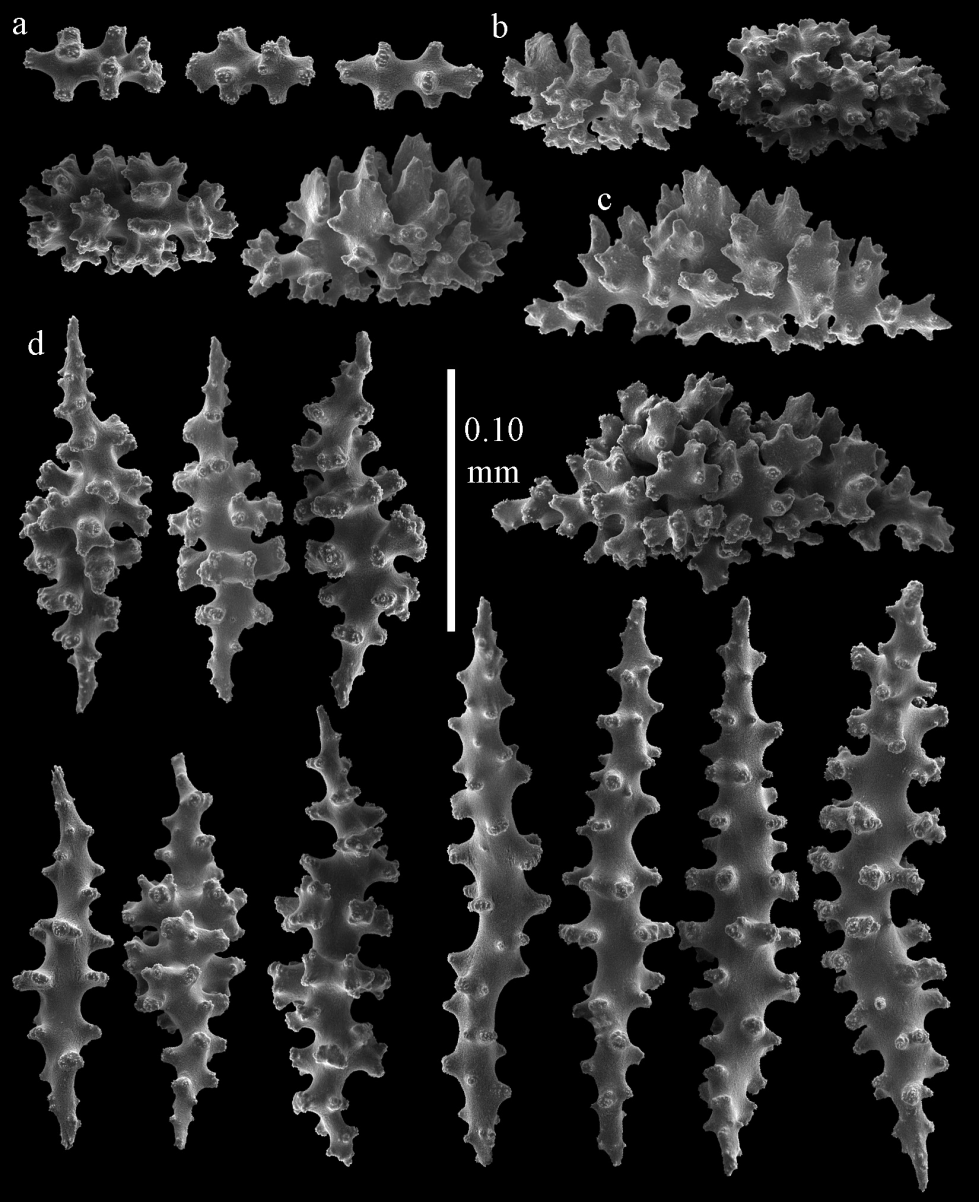
Coenenchymal sclerites of *Melitodes densa*, ZMB 5809; **a** capstans **b** unilaterally spinose spheroids **c** unilaterally spinose spindles **d** spindles.

*Melithaea densa* is also reported to occur in shallow water. According to Kükenthal (1909) it resembles *Melithaea flabellifera* very much but differs in having more spinose collaret and point sclerites, more densely, stronger ornamented coenenchymal sclerites, and the color always being red with yellow polyps. Later, he separated the two species with *Melithaea densa* having no clubs (Kükenthal 1924). The ZMB 5801 colony examined by us (Fig. 36c) showed many disintegrated sclerites (Figs 45, 46). As this mostly concerned the smaller sclerites we were unable to show the capstans and small clubs. But we found a few larger calyx clubs, apparently overlooked by Kükenthal (1909). Also most point sclerites were badly damaged. We also examined ZMB 5809 (Fig. 36d), which had its sclerites less disintegrated (Figs 47, 48).

Kükenthal (1924: 53) referred *Pleurocorallium confusum* to *Pleurocoralloides*. Bayer and Cairns (2003: 222) suggested that the species belongs to *Acabaria*. Moroff (1902a, b), in his descriptions of the species, mentioned flattened branches; polyps on one side of the colony; sclerites straight or bent 0.25 mm long spindles; also plate-like sclerites and crosses present; colony red with yellow polyps. The type seems to be lost. Because of its flattened branches we consider *Pleurocoralloides confusum* synonymous with *Melithaea japonica*.

**Figure 49.**
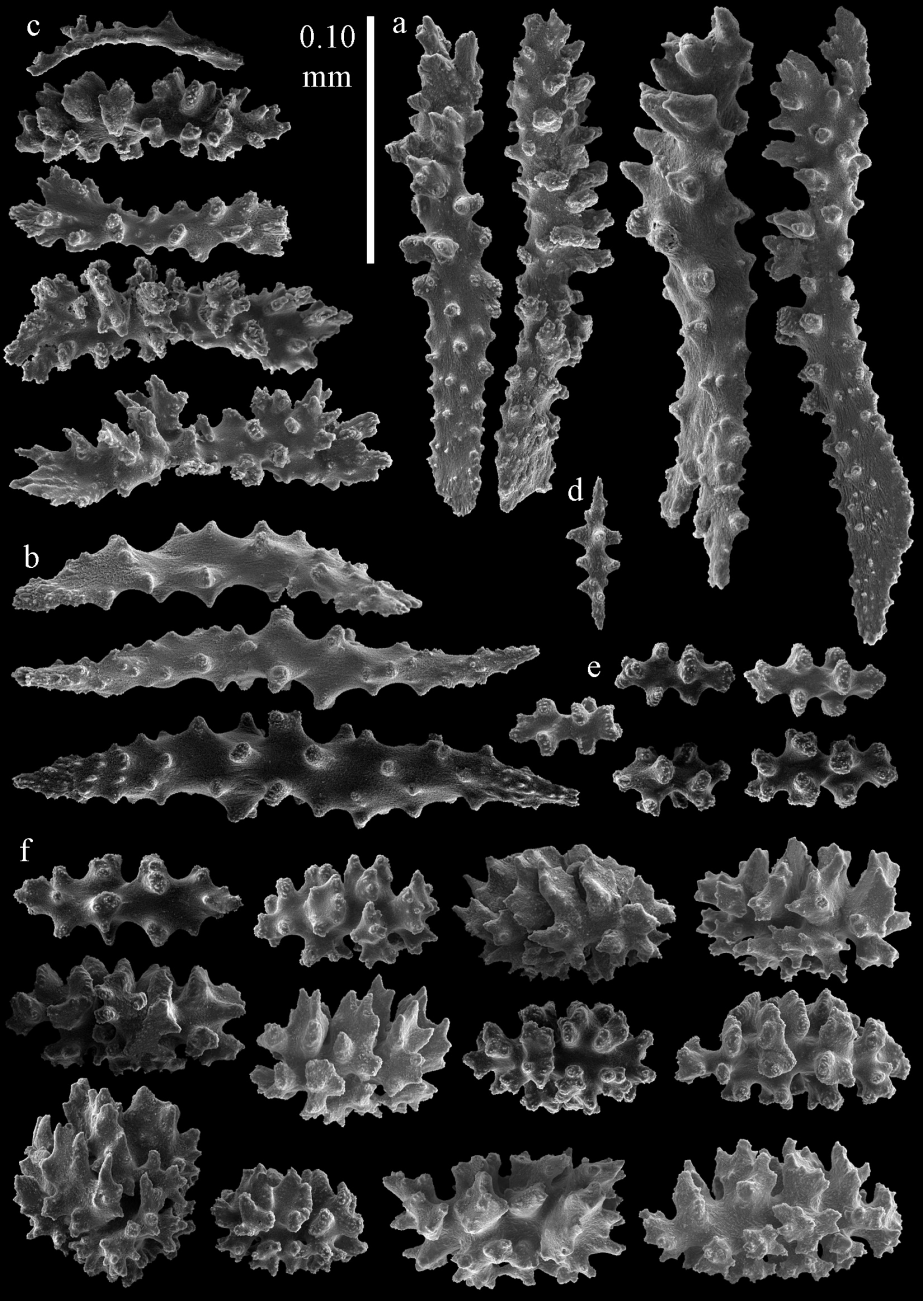
Sclerites of *Pleurocoralloides formosum*, ZSM 20051735; **a** point spindles **b** collaret spindles **c** tentacle sclerites **d** pharynx rod **e** capstans **f** unilaterally spinose spheroids.

**Figure 50.**
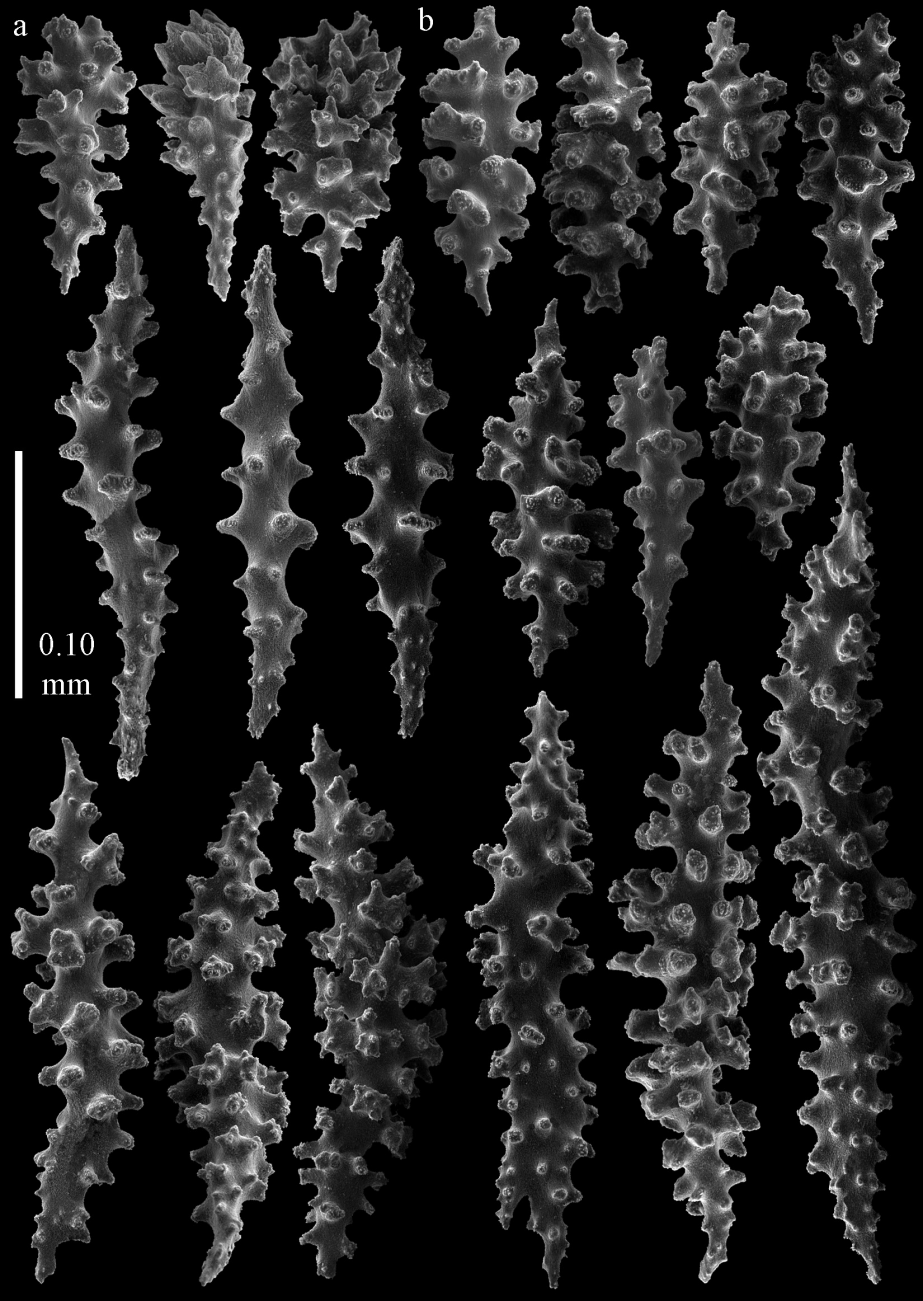
Sclerites of *Pleurocoralloides formosum*, ZSM 20051735 **a** clubs of calyx **b** spindles of coenenchyme.

Bayer and Cairns (2003: 222) suggested *Pleurocoralloides formosum* to belong to *Acabaria*. It was described as having polyps with 7 spindles per point and 6 rows in the collaret; sclerites orange, tentacles ones yellow, axis orange. We consider it a synonym of *Melithaea japonica*; the colonies are shown in Fig. 36a, its sclerites are depicted in Figs 49, 50.

*Melithaea flabellifera* has been described with two variations: Melithaea flabellifera var. reticulata from Sagami Bay, 80–250 m depth. It differs in having many anastomoses, and no flattened branches, color orange red, sclerites bigger and more spinose. Depository of material is unknown.

Melithaea flabellifera var. cylindrata from an unknown locality in Japan also lacks flattened branches, color red with yellow polyps, sclerites are less spinose. Two syntypes are reported to be in Frankfurt, SMF 1260 and 1262, but we could not find these specimens during a visit.

**Figure 51.**
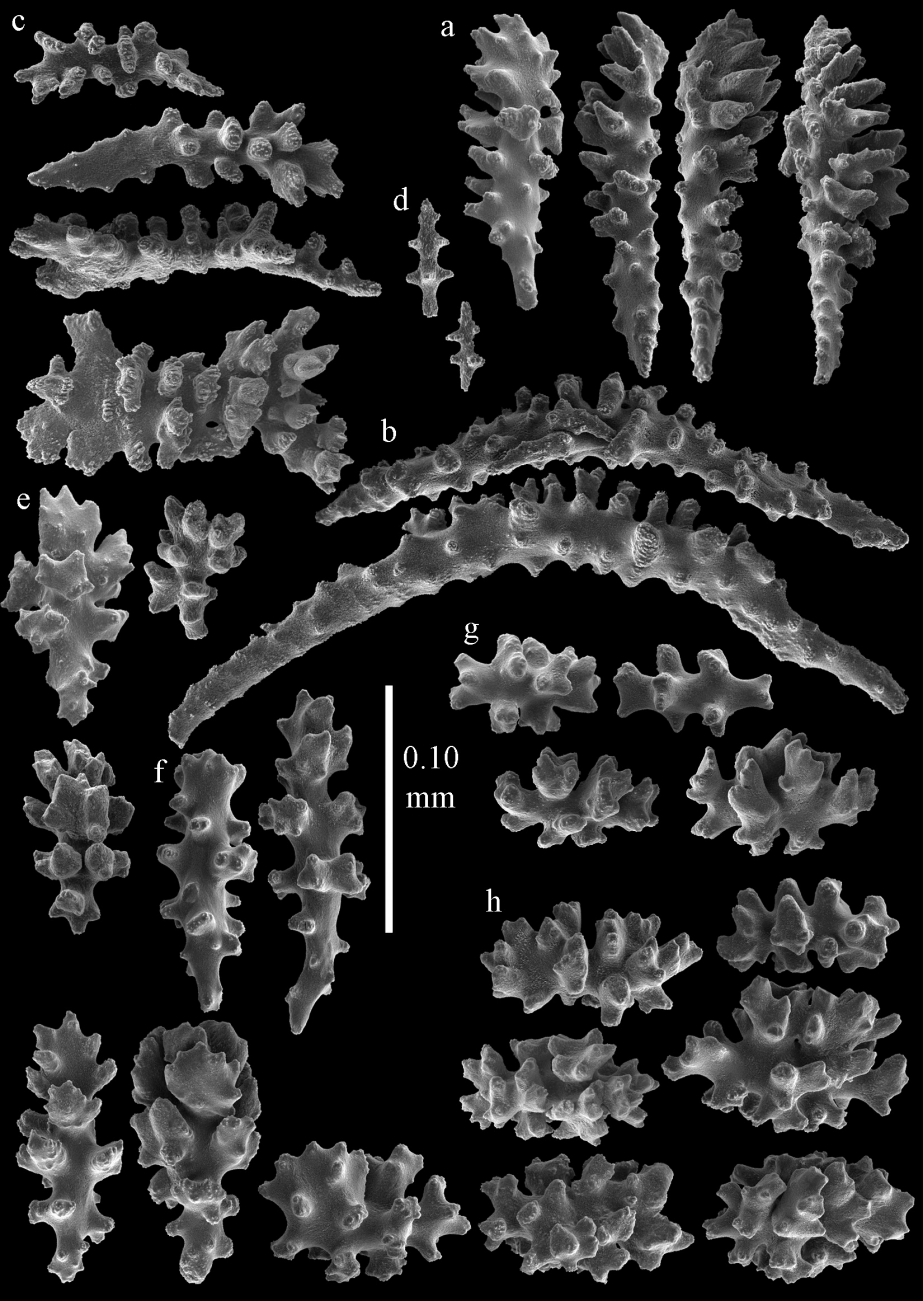
Sclerites of *Melithaea japonica*, RMNH Coel. 41922; **a** point spindles **b** collaret spindles **c** tentacle sclerites **d** pharynx rods **e** clubs of coenenchyme **f** clubs of calyx **g** capstans **h** unilaterally spinose spheroids.

**Figure 52.**
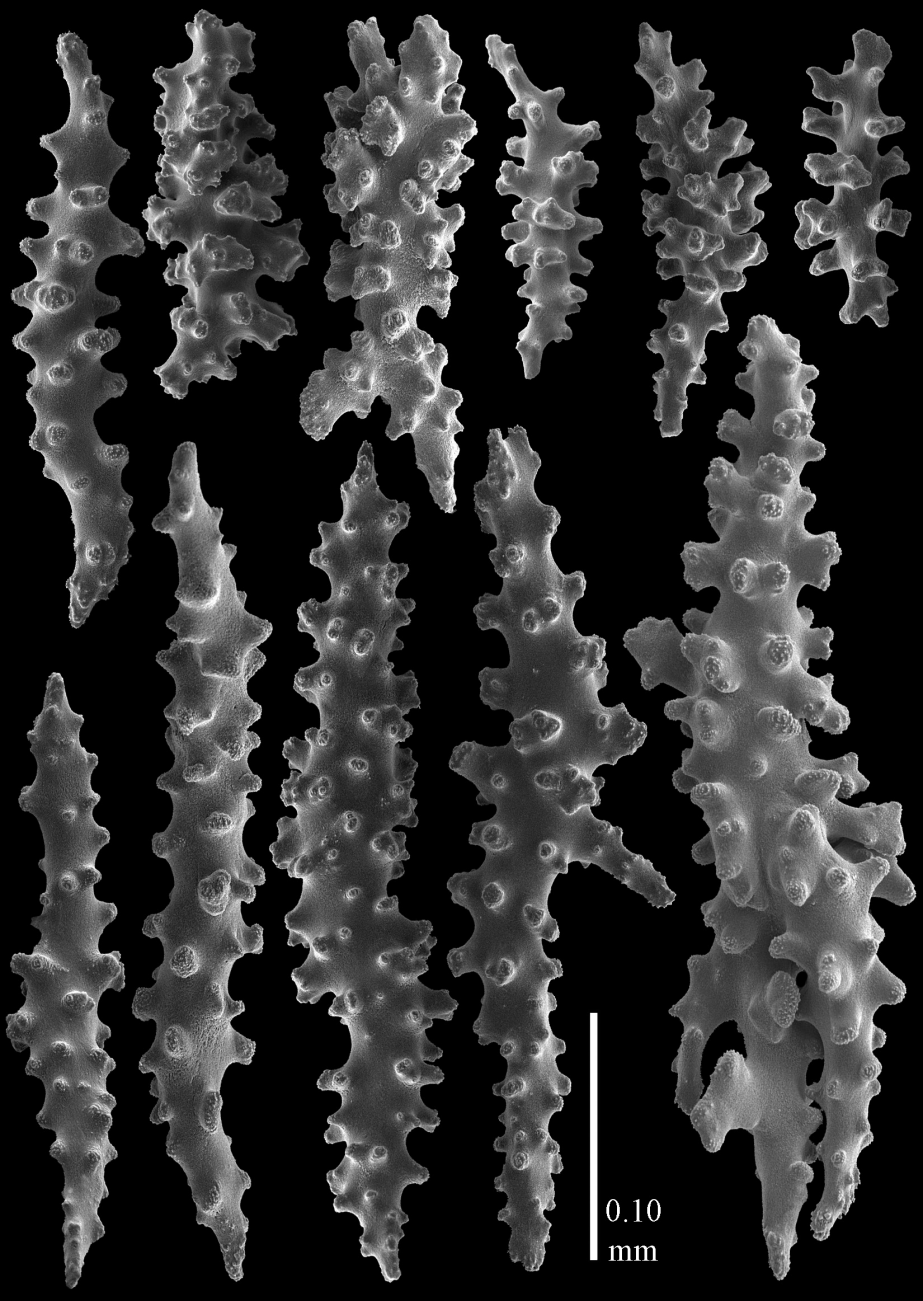
Coenenchymal sclerites of *Melithaea japonica*, RMNH Coel. 41922.

In northern Japan we found quite a number of specimens: RMNH Coel. 41915–41924. As an example we show the colony of RMNH Coel. 41922 (Fig. 37c) and its sclerites (Figs 51, 52). Here, the smallest colonies are white or yellow, only RMNH Coel. 41922 is red.

**Figure 53.**
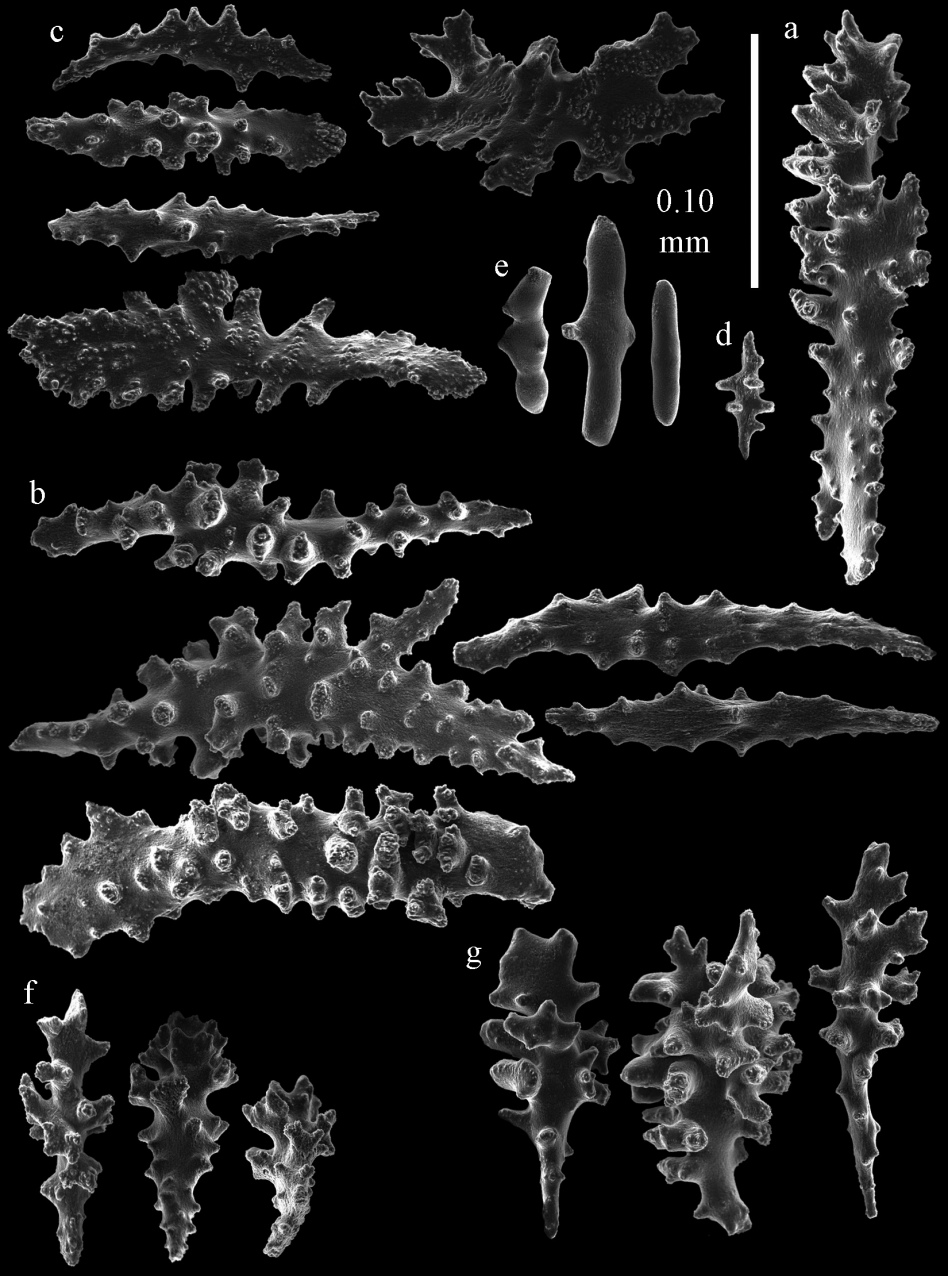
Sclerites of *Melithaea japonica*, BIK-G224; **a** point spindle **b** collaret spindles **c** tentacle sclerites **d** pharynx rod **e** axial rods **f** clubs of coenenchyme **g** clubs of calyx.

**Figure 54.**
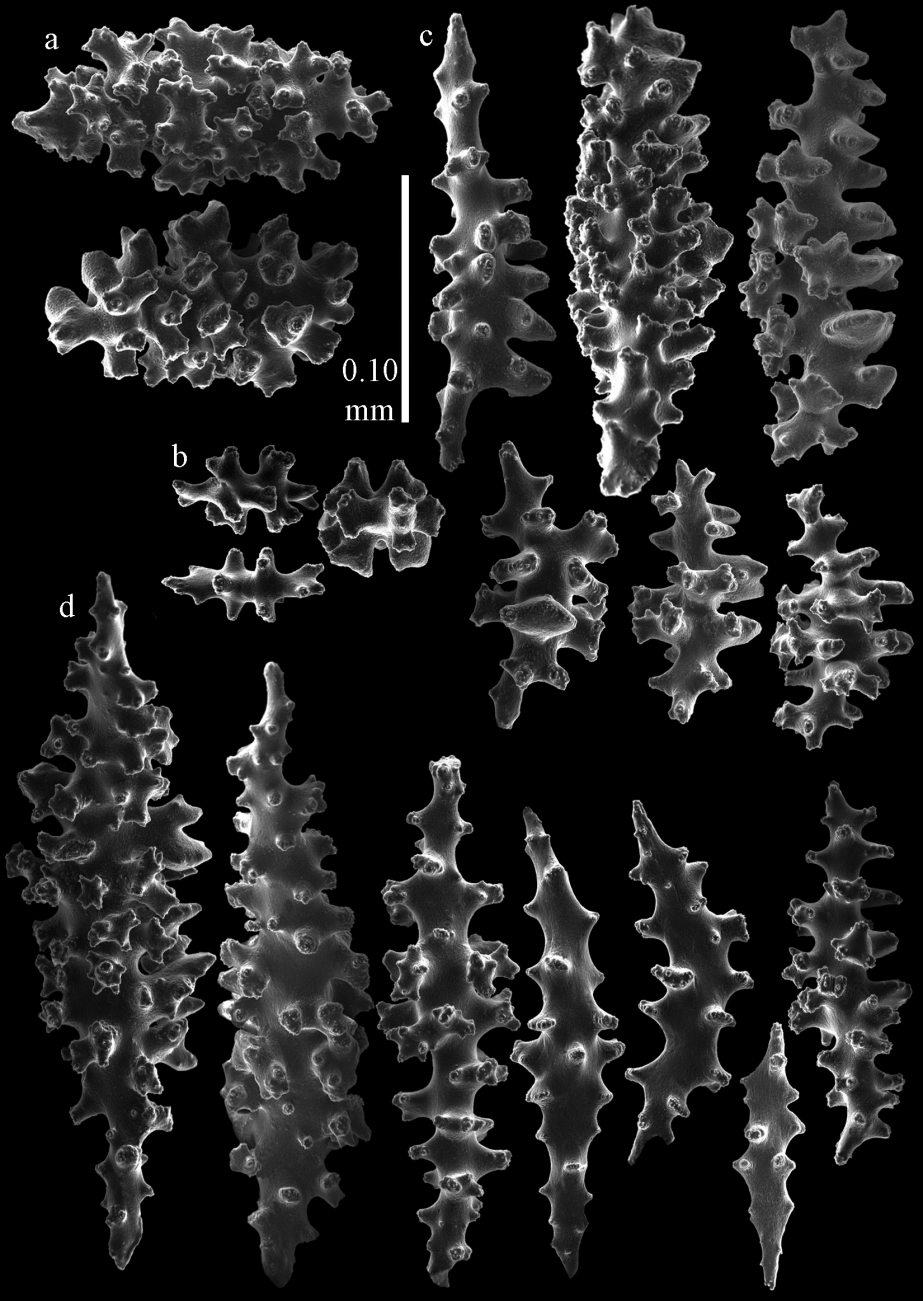
Sclerites of *Melithaea japonica*, BIK-G224; **a** unilaterally spinose spheroids **b** capstans **c** unilaterally spinose spindles **d** spindles.

We found three specimens with extreme slender sclerites: BIK-G224 (Fig. 37a; sclerites Figs 53, 54), BIK-G226, and RMNH Coel. 41914.

**Figure 55.**
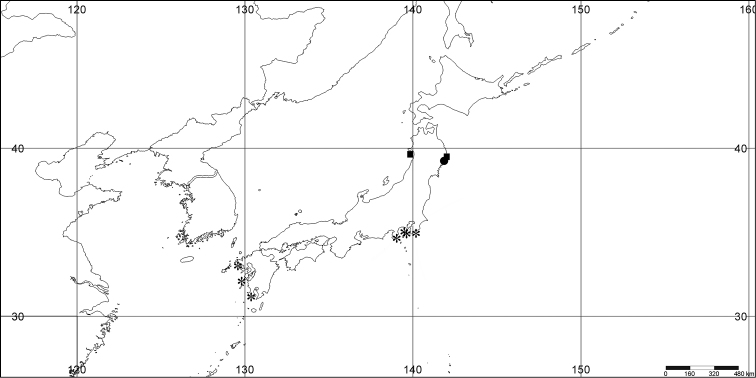
Distribution of *Melithaea japonica* (*), north *Melithaea japonica* (●), and slender *Melithaea japonica* (■).

### Melithaea keramaensis sp. n.

Taxon classificationAnimaliaAlcyonaceaMelithaeidae

http://zoobank.org/1D358214-BC23-4184-82CB-EDF230FD292B

**Figure 56.**
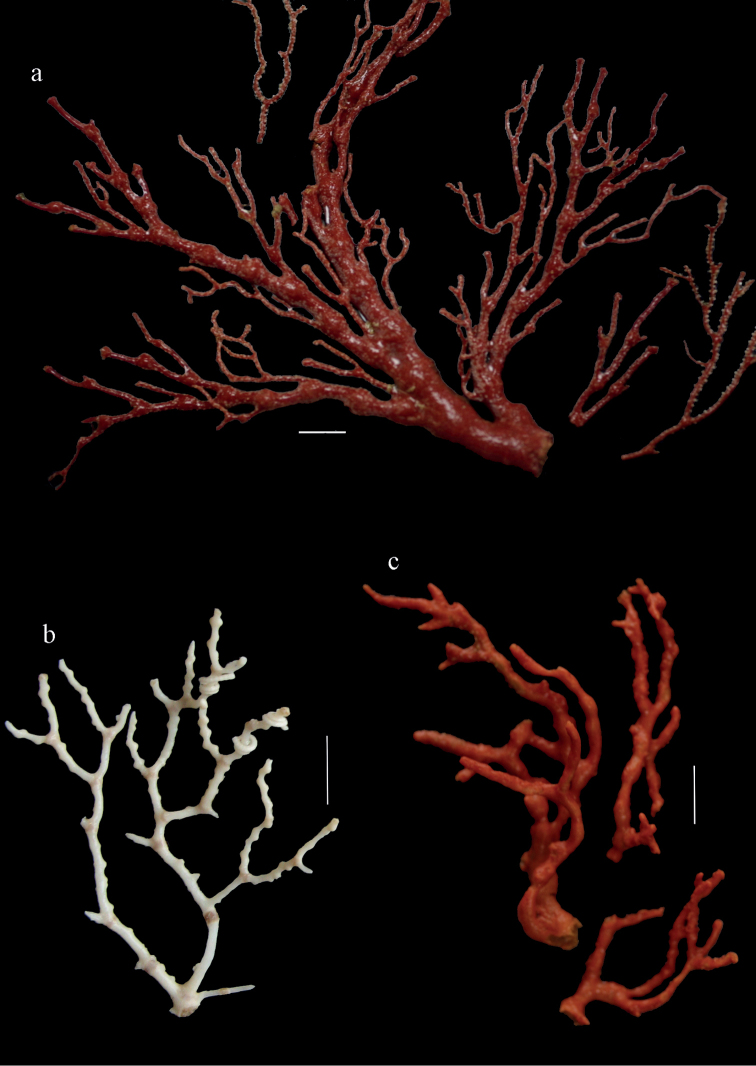
**a** *Melithaea keramaensis* sp. n., RMNH Coel. 41925, holotype **b** *Melithaea modesta*, ZMB 5807, syntype **c** *Melithaea mutsu* SMBL-Cni1017.

**Figure 57.**
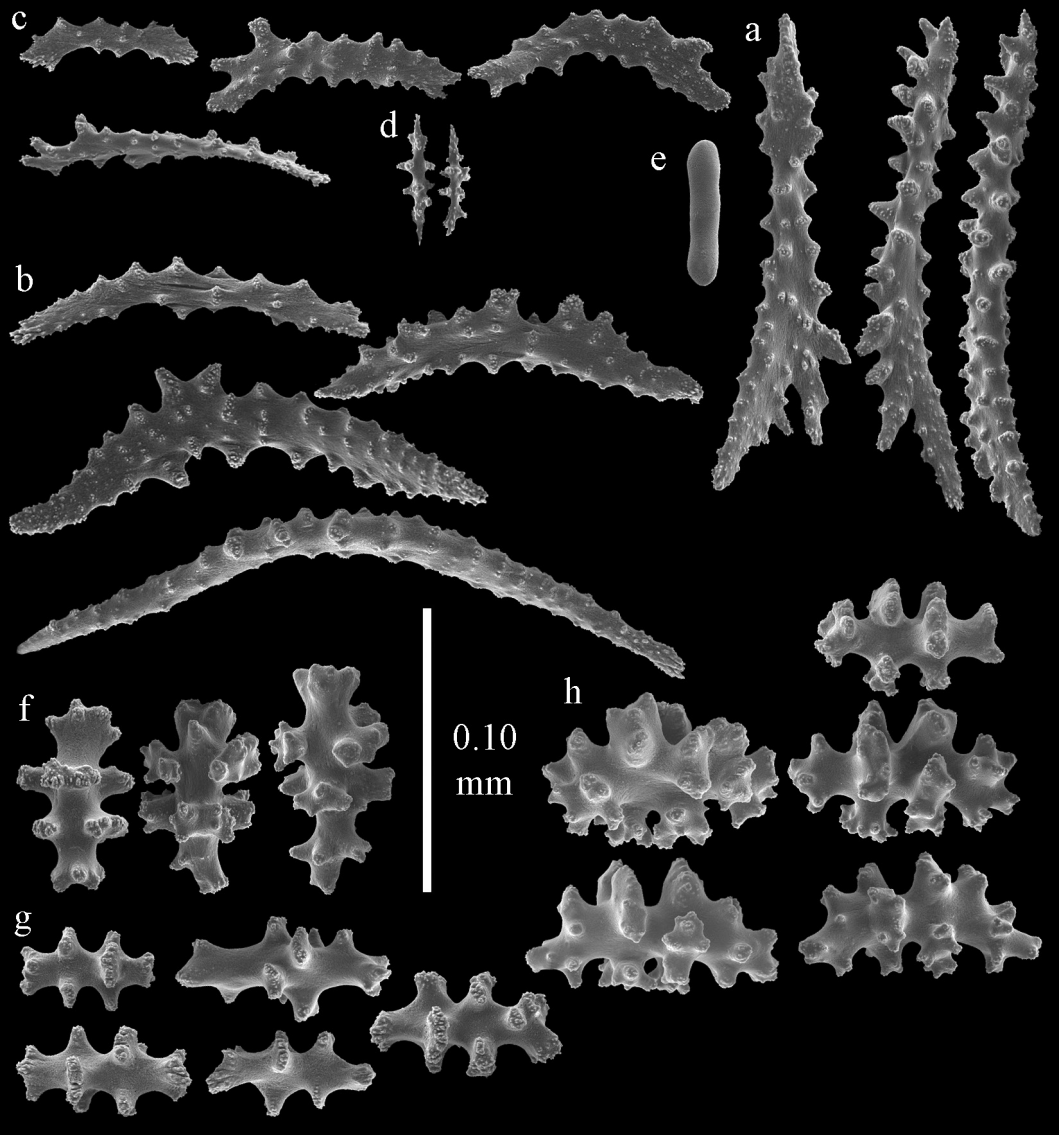
Sclerites of *Melithaea keramaensis* sp. n., RMNH Coel. 41925; **a** point spindles **b** collaret spindles **c** tentacle sclerites **d** pharynx rods **e** axial rod **f** clubs of coenenchyme **g** capstans **h** double disks.

**Figure 58.**
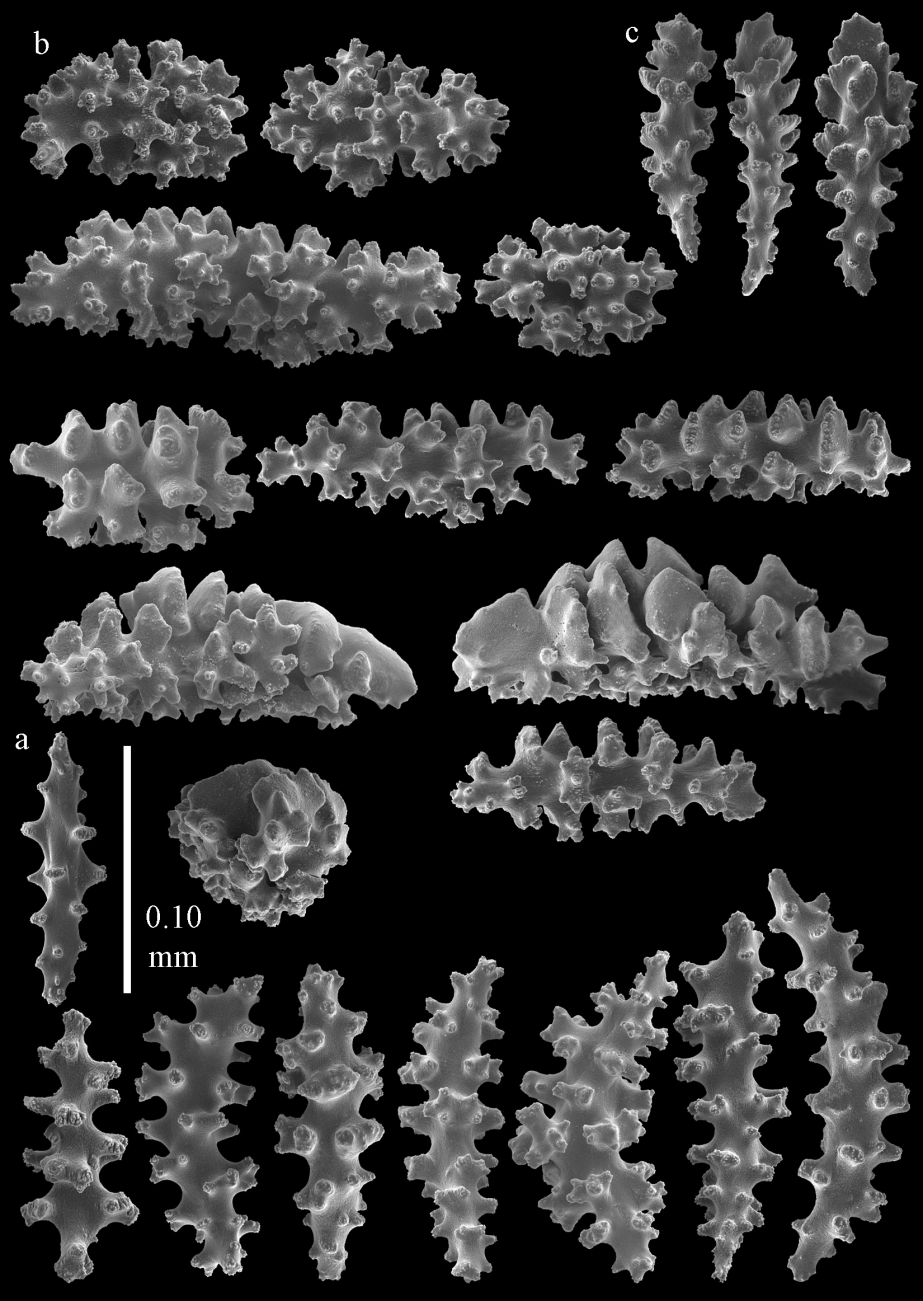
Sclerites of *Melithaea keramaensis* sp. n., RMNH Coel. 41925; **a** spindles **b** disk spindles, the top four seen from the underside **c** clubs of calyx.

Figures 56a , 57 , 58 , 65

#### Material examined.

Holotype **RMNH Coel. 41925 (AKM 1148)**, off Kerama Isls., Okinawa Prefecture, Japan, 26°09.45'N, 127°26.90'E – 26°09.65'N, 127°26.81'E, 85–71 m, *R/V Tansei-maru*, KT08-33, St. KR-09, Chain Bag Dredge, coll. A.K. Matsumoto, 15 December 2008; paratype **RMNH Coel. 41926 (AKM 1139)**, off Kerama Isls., Okinawa Prefecture, Japan, 26°12'N, 127°30'E, 56–51 m, *R/V Tansei-maru*, KT08-33, st. KR-10, coll. A.K. Matsumoto, 15 December 2008.

#### Description.

The holotype is a 12 cm long fragment without holdfast (Fig. 56a). At the base the stem is 1 cm wide, the end branches are only 1 mm wide. The polyps are situated laterally on the branches, the calyces hardly project beyond the coenenchyme and most polyps are retracted. Points with slightly bent spindles up to 0.20 mm long, distal end with more developed tubercles (Fig. 57a). Collaret with bent spindles up to 0.25 mm long, middle part with more developed tubercles (Fig. 57b). Tentacles with platelets, the larger ones crescent-shaped with irregular projections (Fig. 57c). These platelets are up to 0.15 mm long. Pharynx with straight spiny rods, up to 0.05 mm long (Fig. 57d). Coenenchyme with predominantly capstans (Fig. 57e), double disks (Fig. 57h), and small clubs (Fig. 57f), 0.05–0.08 mm long. Spindles (Fig. 58a) and disk spindles (Fig. 58b) are also common, 0.10–0.15 mm long. The calyces with additional leaf clubs, up to 0.15 mm long (Fig. 58c).

#### Color.

Colony red with white calyces and polyps. Coenenchymal sclerites orange, calyx and polyp sclerites colorless.

#### Variation.

The paratype is very much alike the holotype regarding color and sclerites.

#### Distribution.

The species is only known from the Kerama Islands (Fig. 65).

**Etymology.** The species is named after its type locality, the Kerama islands.

#### Remarks.

The species mostly resembles *Melithaea abyssicola* but differs in having longer disk spindles but shorter normal spindles. It also has more finely sculptured polyp sclerites.

### Melithaea modesta

Taxon classificationAnimaliaAlcyonaceaMelithaeidae

(Kükenthal, 1908)

**Figure 59.**
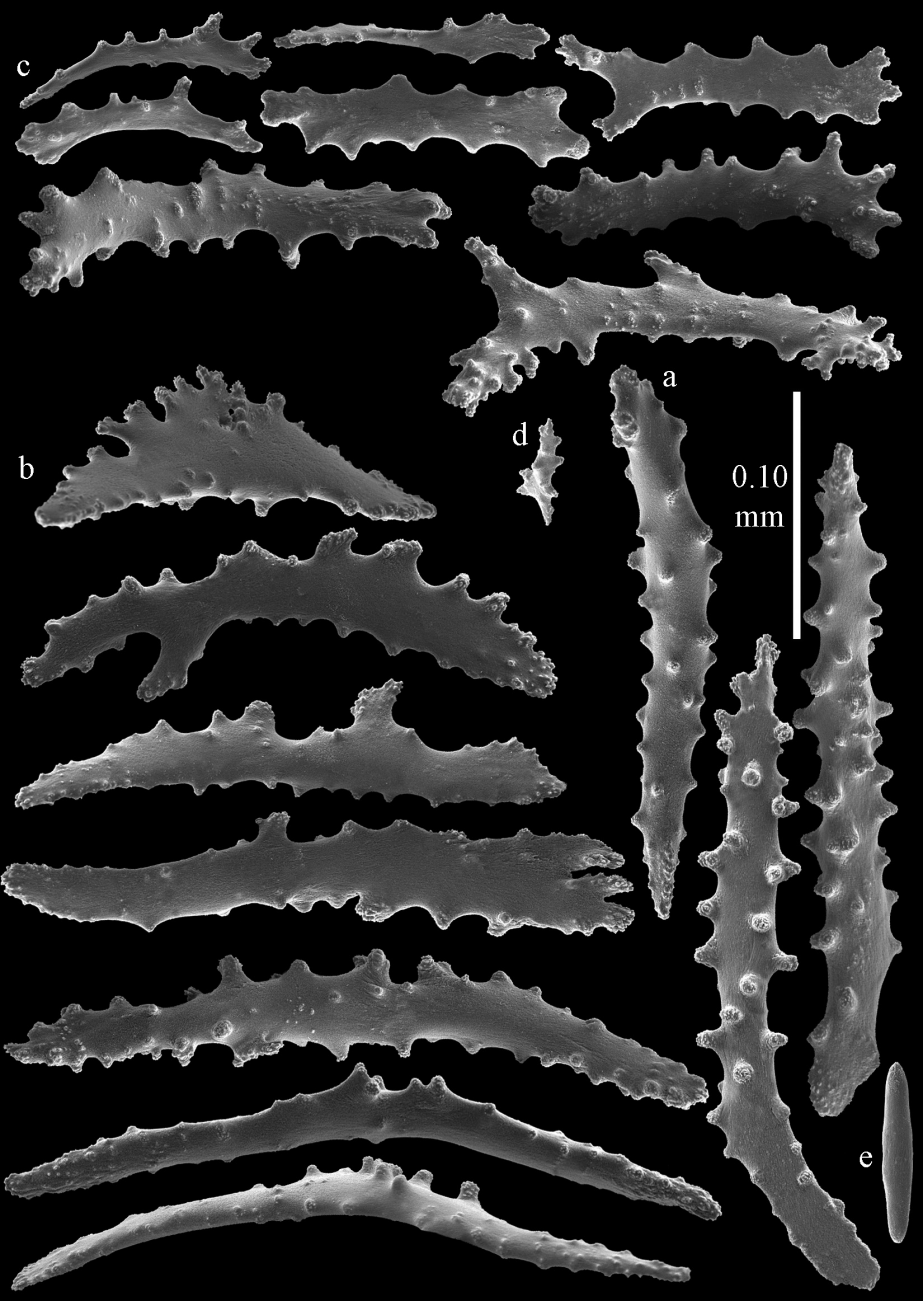
Sclerites of *Melithaea modesta*, ZMB 5807, syntype; **a** point spindles **b** collaret spindles **c** tentacle sclerites **d** pharynx rod **e** axial rod.

**Figure 60.**
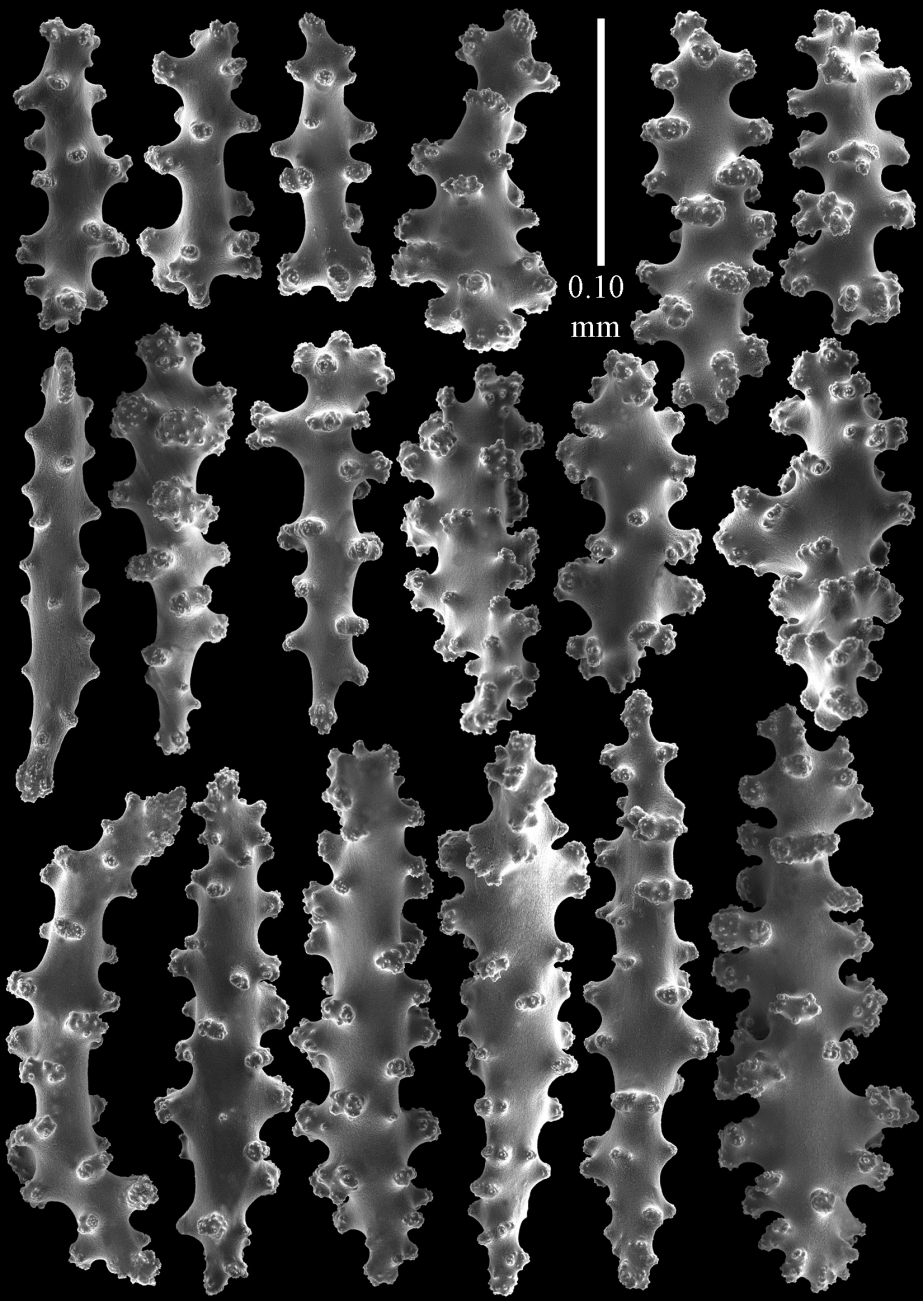
Coenenchymal sclerites of *Melithaea modesta*, ZMB 5807, syntype.

**Figure 61.**
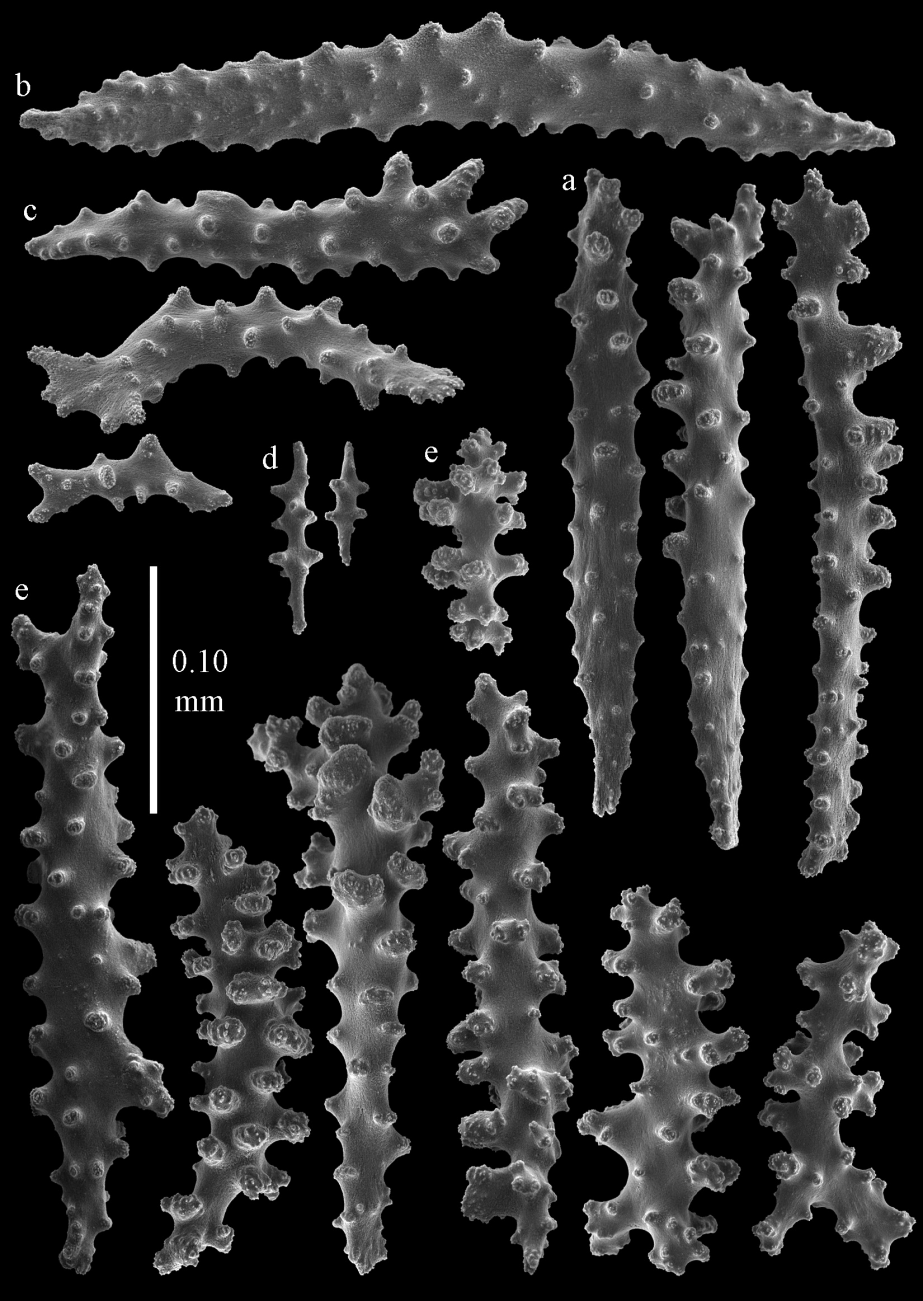
Sclerites of *Melithaea modesta*, ZMUC ANT-000649; **a** point spindles **b** collaret spindle **c** tentacle sclerites **d** pharynx rods **e** coenenchymal spindles.

**Figure 62.**
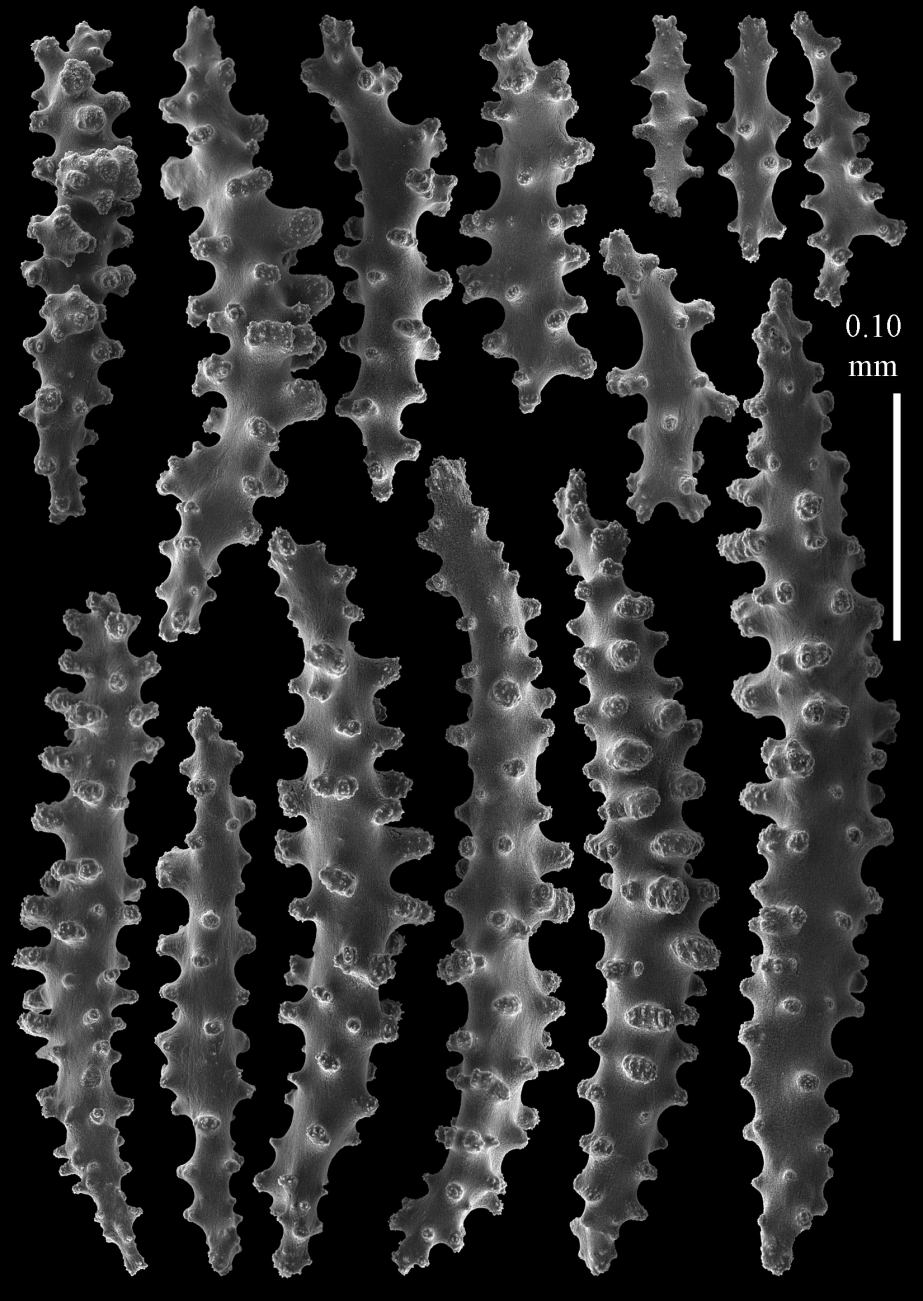
Coenenchymal sclerites of *Melithaea modesta*, ZMUC ANT-000649.

Figures 56b , 59 , 60 , 61 , 62 , 65

Acabaria modesta Kükenthal 1908: 197; 1909: 66, figs 73–75, pl. 5 fig. 30 (Sagami Bay, Japan); 1919: 183; 1924: 79; Hickson 1937: 181.
Not Melithaea modesta (Nutting, 1911) = *Melithaea planoregularis* (Kükenthal, 1910).

#### Material examined.

Syntype **ZMB 5807**, Sagami Bay (Japan), 80-250 m (label 600 m), coll. Doflein 1904/05; previously unidentified museum material: **ZMUC ANT-000595**, Okinose, Sagami Sea, 60 fms (86–110 m), coll. Dr. Th. Mortensen, 11 June 1914; **ZMUC ANT-000649**, same data as ZMUC ANT-000595; **UMUTZ-CnidG-19**, Kashiwa-jima Is. Tosa, Kochi Prefecture, Japan, probably collected by K. Kinoshita during his Kashiwa-jima Is. Coral Ground Expedition, June 1909; **UMUTZ-CnidG-27**, coral ground, Uji Isls. Satsuma, Kanogshima Prefecture, Japan, ca. 80 fms (114–121 m), Kinoshita leg. coll. K. Kinoshita, June 1908; **AKM 443**, Sakai Port, Minabe, Wakayama Prefecture, ca.33°7445'N, 135°3329'E, shallower than 100 m, lobster-net, coll. A.K. Matsumoto, 1 April 2003; **AKM 572**, Otsuki, Tosa, Kochi Prefecture, 32°43'N, 132°48'E, 84.75-83.1 m, local fishermen’s boat, *Kiryo-maru*, coral net, st. 3, coll. A.K. Matsumoto, 7 October 2004; **AKM 740**, off Sata-misaki Cape, Kagoshima Prefecture, 31°00.50'N, 130°35.09'E – 31°01.3211'N, 130°34.6509'E, 178-189 m, *R/V Tansei-maru*, KT07-1, st. SM-2, coll. A.K. Matsumoto, 23 February 2007; **AKM 904**, Hachijo Is. Izu Isls., 33°20.9082'N, 139°41.1841'E – 33°21.0775'N, 139°40.4931'E, 213-185 m, *R/V Tansei-maru*, KT07-31, st. 14, coll. A.K. Matsumoto, 26 November 2007; **AKM 1575**, Takase west, Izu Is., Japan, ca. 34°21'N, 138°52'E – 34°26'N, 139°07'E, 221–244 m, *R/V Tansei-maru*, KT-87-19 cruise, St. TW02, large cylindrical dredge, coll. Suguru Ohta, 8 December1987; **AKM 1594**, SE off Taito-Saki, Boso, Chiba Prefecture, 35°05.086'N, 140°51.718'E – 35°04.176'N, 140°50.921'E, 975–1027 m, *R/V Tansei-maru*, KT03-17, St. TS6-3, 3 m ORE beam trawl, coll. Suguru Ohta, 17 November 2003; **AKM 1601**, Otsuki, Tosa, Kochi pref., 32°37.66'N, 132°50.44'E –32°37.56'N, 132°47.88'E, 114 m, local fishermen’s boat, *Kiryo-maru*, St.1, coll. A.K. Matsumoto, 7 October 2004.

#### Re-description.

Colony branched in one plane with few anastomoses (Fig. 56b). Points with slightly bent spindles up to 0.25 mm long, distal end with more developed tubercles (Fig. 59a). Collaret with bent, rather smooth spindles up to 0.30 mm long, middle part with tubercles (Fig. 59b). Tentacles with platelets, the larger ones crescent-shaped (Fig. 59c). These platelets are up to 0.18 mm long. Pharynx with straight spiny rods, up to 0.05 mm long (Fig. 59d). Coenenchyme with spindles 0.10–0.25 mm long (Fig. 60), with simple or complex tubercles. The axis has smooth and sparsely tuberculate rods (Fig. 59e).

#### Color.

Colony white, sclerites colorless.

#### Variation.

ZMUC ANT-000649 showed somewhat longer spindles, up to 0.40 mm long (Figs 61, 62).

#### Distribution.

Pacific side of Japan (Fig. 65); northern limit is Sagami Bay.

#### Remarks.

The species is easily recognized by its white colony color, rather smooth polyp sclerites, and presence of spindles only.

### Melithaea mutsu

Taxon classificationAnimaliaAlcyonaceaMelithaeidae

Minobe, 1929

**Figure 63.**
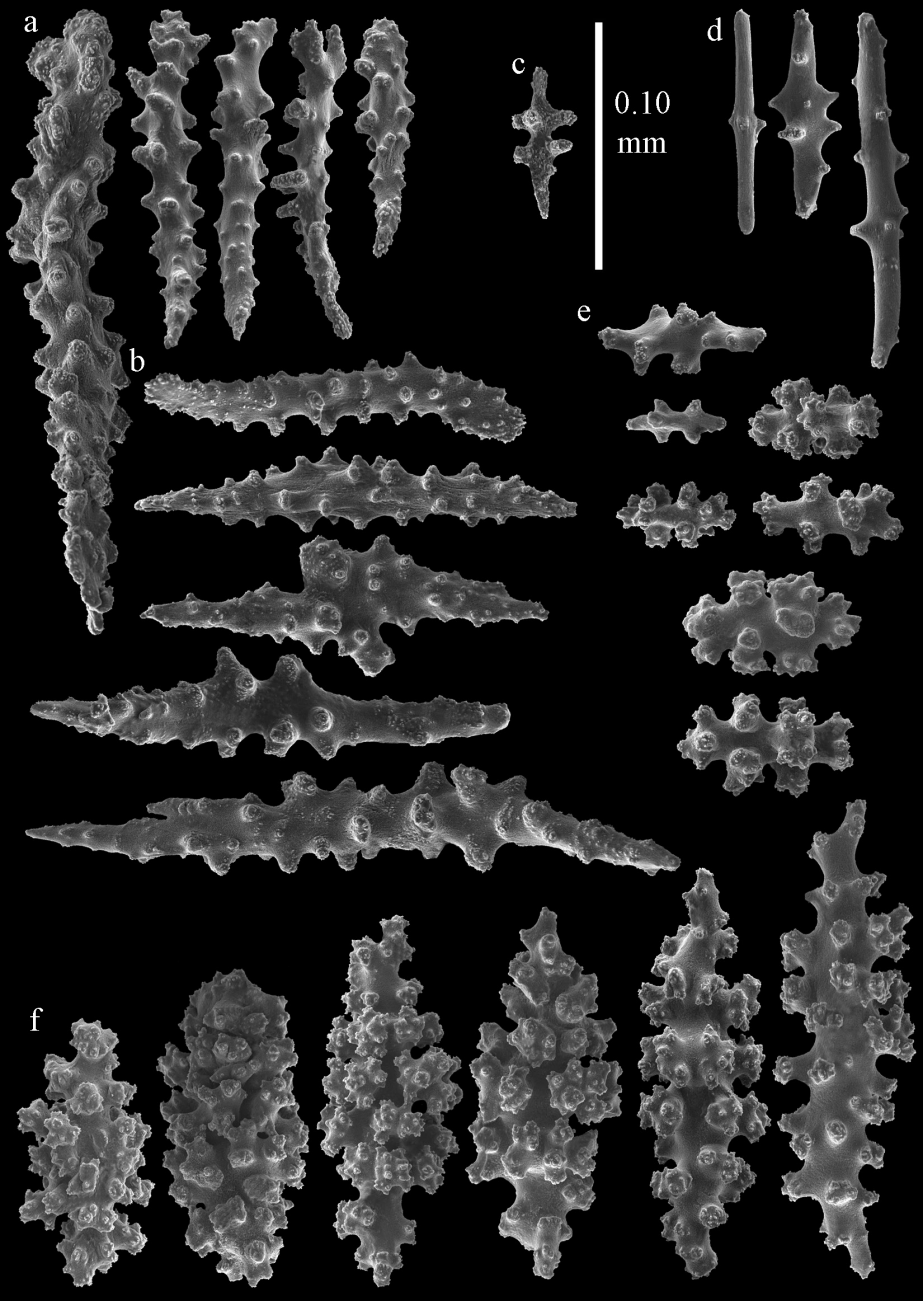
Sclerites of *Melithaea mutsu*, SMBL-Cni1017, neotype; **a** point spindles **b** collaret spindles **c** pharynx rod **d** axial rods **e** capstans **f** coenenchymal spindles.

**Figure 64.**
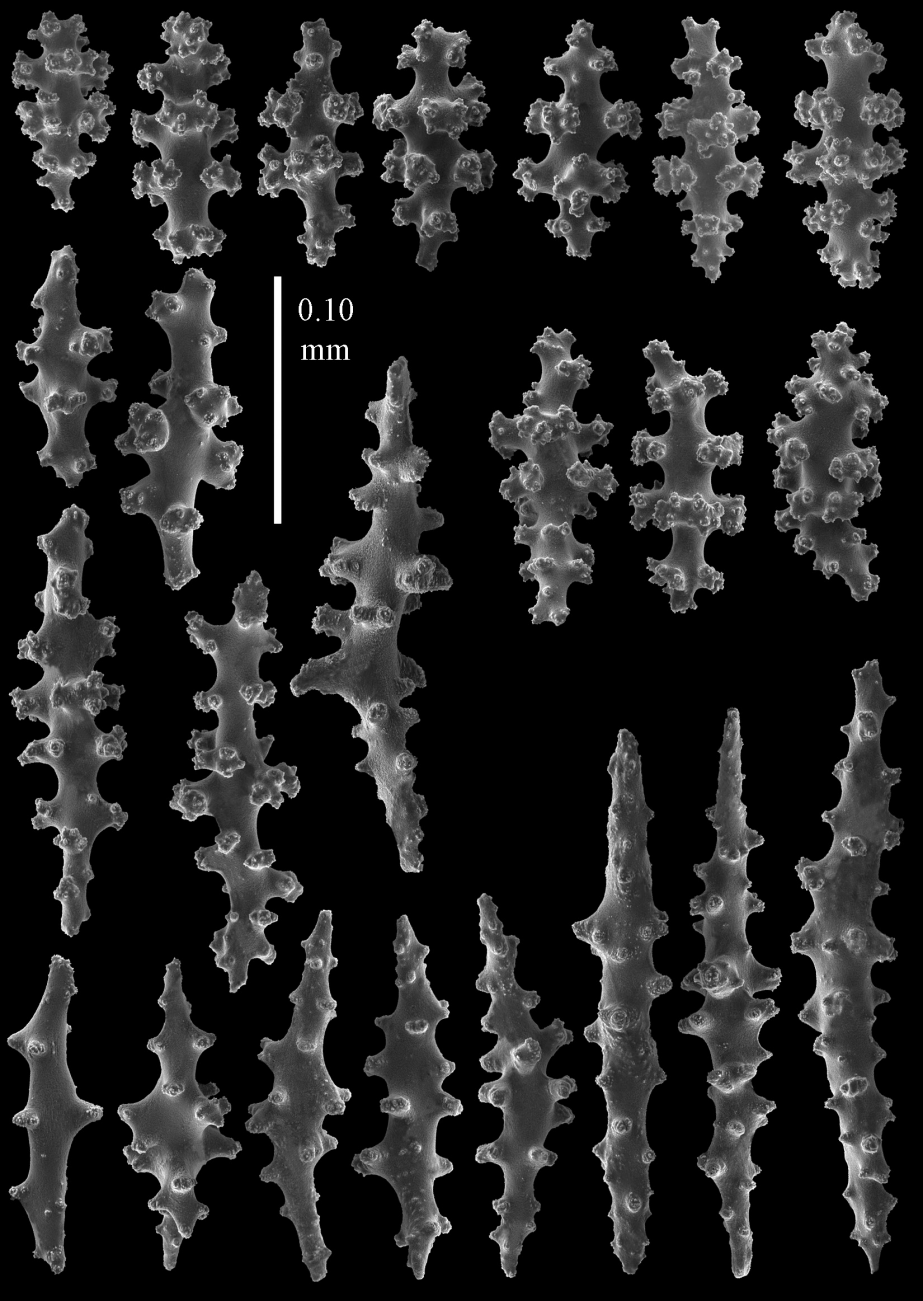
Coenenchymal sclerites of *Melithaea mutsu*, SMBL-Cni1017, neotype.

**Figure 65.**
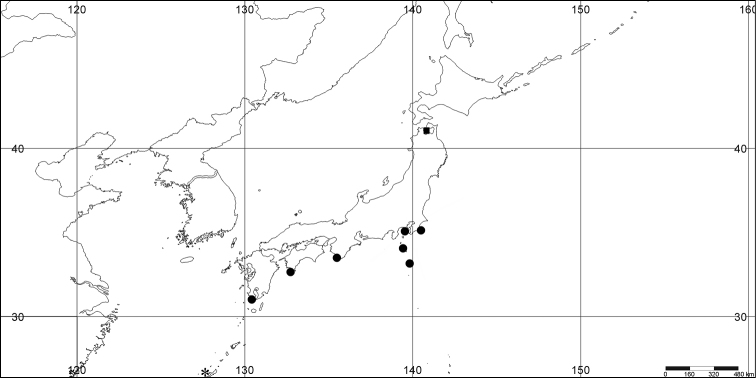
Distribution of *Melithaea keramaensis* sp. n. (*), *Melithaea modesta* (●), and *Melithaea mutsu* (■).

Figures 56c , 63 , 64 , 65

Melithaea mutsu Minobe 1929: 671, figs 1–2 (Mutsu Bay, Japan).

#### Material examined.

Neotype **SMBL-Cni1017**, Sai, Mutsu Bay, Aomori Prefecture, close to Tsugaru Straits between Sea of Japan and NW Pacific, 5 m, coll. M. Nishihira and Hoshiai, 13 August 1964.

#### Description.

Colony broken up, consisting of three fragments, bushy with few anastomoses. The largest fragment is 7.3 cm long without holdfast (Fig. 56c). At the base the stem is 7 mm wide, the end branches are 2 mm wide. The polyps are arranged randomly, the calyces hardly project beyond the coenenchyme and most polyps are retracted. Points with slightly bent spindles up to 0.30 mm long, distal end with more tubercles (Fig. 63a). Collaret with bent spindles up to 0.30 mm long, middle part with tubercles (Fig. 63b). Pharynx with straight spiny rods, up to 0.05 mm long (Fig. 63c). Coenenchyme with predominantly spindles, 0.10–0.25 mm long (Figs 63f, 64), with simple or complex tubercles. Capstans are also present, 0.05–0.10 mm long (Fig. 63e). The axis has smooth and sparsely tuberculate rods (Fig. 63d).

#### Color.

Colony red, sclerites orange.

#### Distribution.

Only known from the northern tip of the main island of Japan, Honshu Is. (Fig. 65).

#### Remarks.

The tentacle platelets were missing. The description and images provided by Minobe are inadequate to identify melithaeids and we were unable to find the depository of the material. As Minobe’s material was collected near Sai, as is our material, we concluded that we have the same species and designated a neotype here. The species is similar to *Melithaea corymbosa*, but lacks clubs in the calyces and disk spindles in the coenenchyme.

### Melithaea nodosa

Taxon classificationAnimaliaAlcyonaceaMelithaeidae

Wright & Studer, 1889

**Figure 66.**
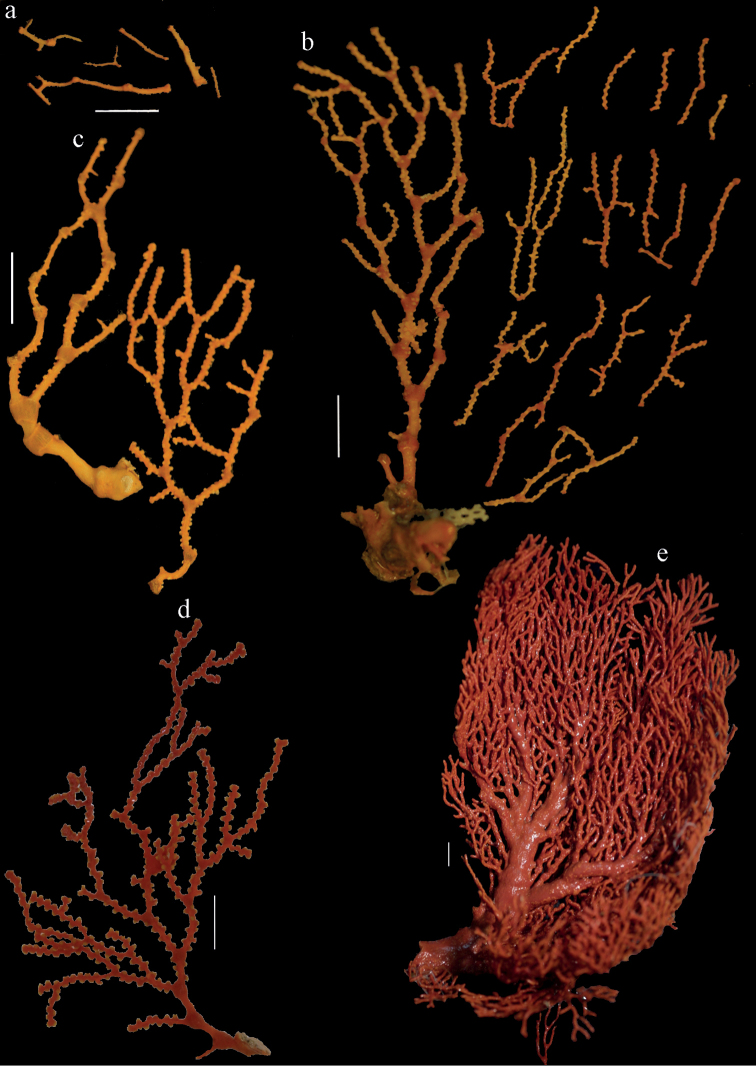
**a** *Melithaea nodosa*, BMNH 1947.3.22.6 **b** BMNH 89.5.27.117 **c** BMNH 89.5.27.118 **d** *Melithaea oyeni* sp. n., RMNH Coel. 41927 **e** *Melithaea ryukyuensis* sp. n., UMUTZ-CnidG-32.

**Figure 67.**
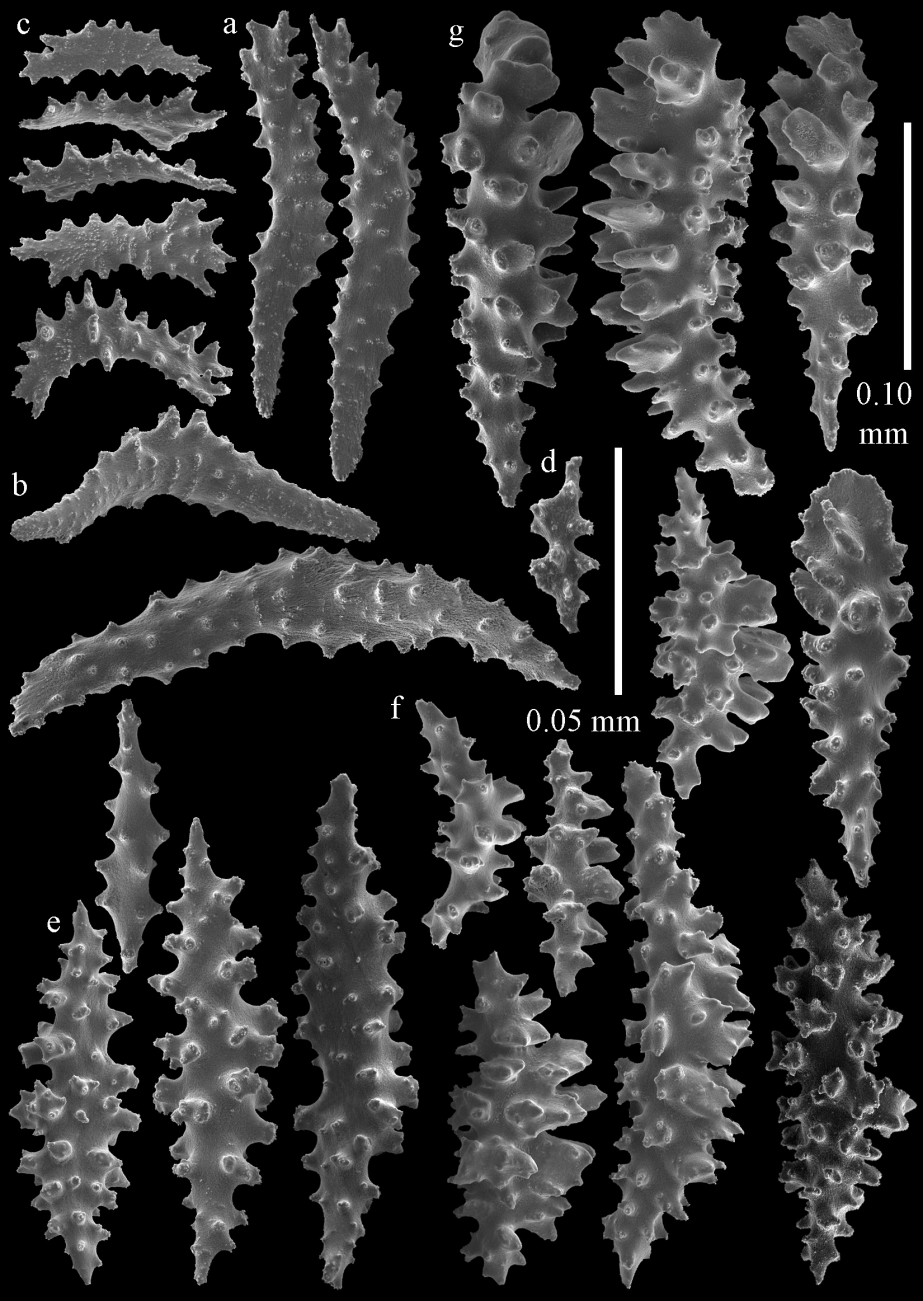
*Melithaea nodosa*, BMNH 89.5.27.117; **a** point spindles **b** collaret spindles **c** tentacle sclerites **d** pharynx rod **e** coenenchymal spindles **f** unilaterally spinose spindles **g** clubs of calyx.

**Figure 68.**
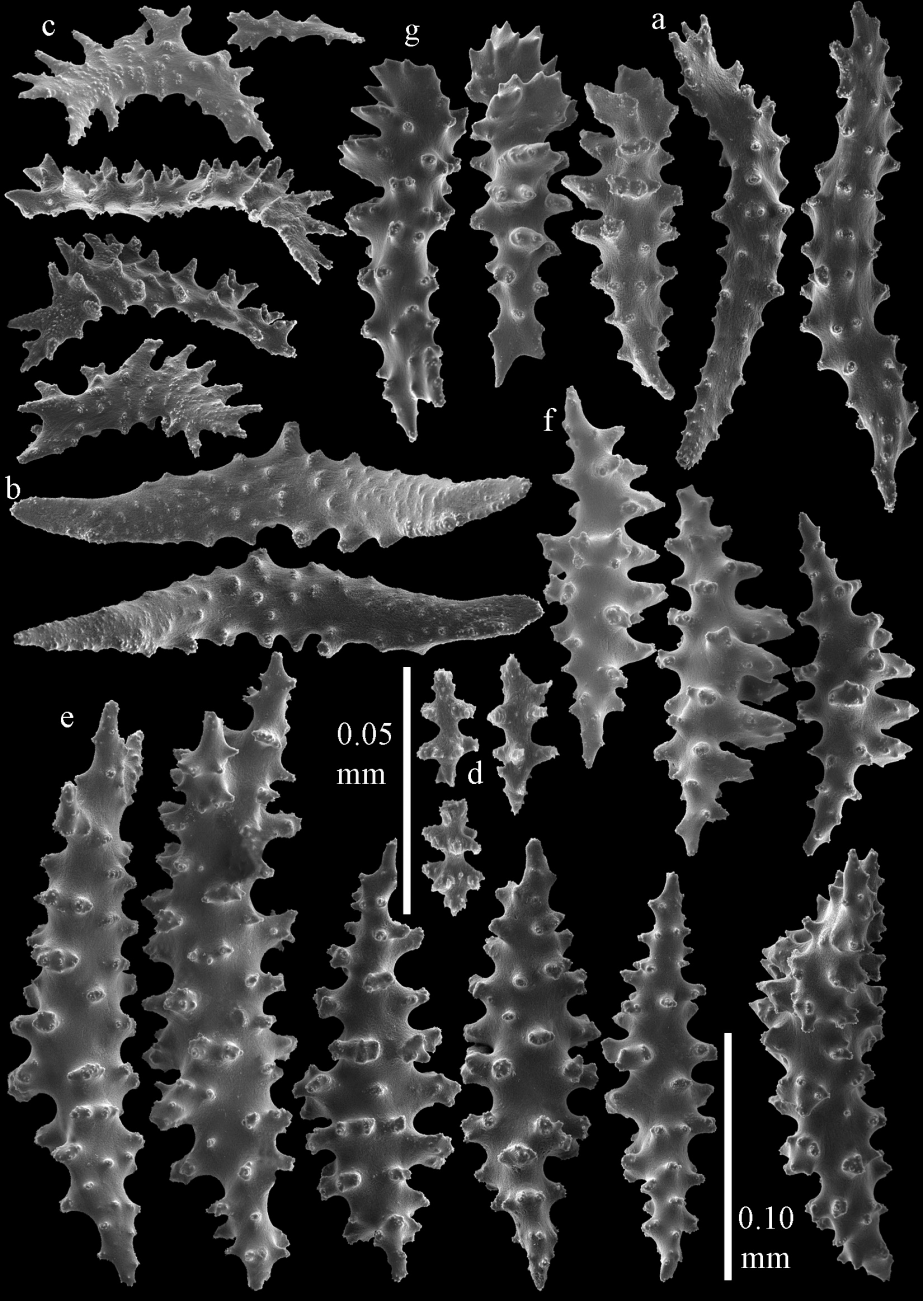
*Melithaea nodosa*, BMNH 89.5.27.118; **a** point spindles **b** collaret spindles **c** tentacle sclerites **d** pharynx rods **e** coenenchymal spindles **f** unilaterally spinose spindles **g** clubs of calyx.

**Figure 69.**
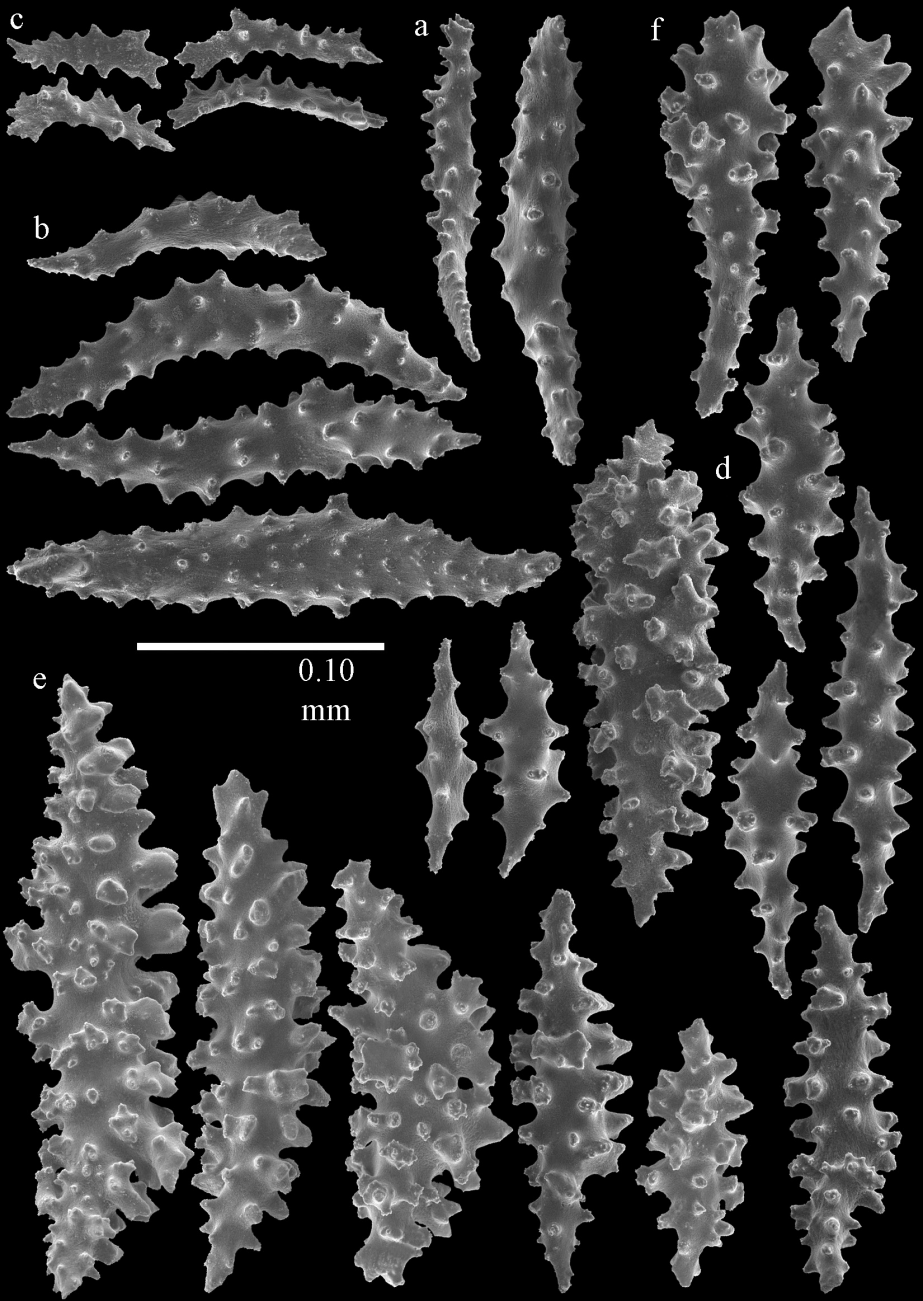
*Melithaea nodosa*, BMNH 1947.3.22.6; **a** point spindles **b** collaret spindles **c** tentacle sclerites **d** coenenchymal spindles **e** unilaterally spinose spindles **f** clubs of calyx.

Figures 66a–c , 67 , 68 , 69 , 74

Melitodes nodosa : Wright and Studer 1889: 178, pl. 40 fig. 10 (off New Hebrides, Hyalonema Ground Japan); Kükenthal 1919: 141; 1924: 57.
Acabaria nodosa : Hickson 1937: 178.
Not Melitodes nodosa : Thomson 1911: 876 (South Africa).

#### Material examined.

Syntype, **BMNH 1947.3.22.6**, New Hebrides, Challenger st. 177, 60-120 fms (86-219 m), dried sclerites, coll. Prof. S.J. Hickson; syntype, **BMNH 89.5.27.117**, *Hyalonema*-Ground (Nishi-no yodomi), South of Japan, 35°11'N, 139°28'E, Challenger st. 232, 345 fms (631 m), 12 May 1875, figured specimen (= *Acabaria nodosa* Prof. Hickson), old label book 2, p. 12; syntype, **BMNH 89.5.27.118** *Hyalonema*-ground (Nishi-no yodomi), south of Japan, 35°11'N, 139°28'E, Challenger st. 232, 345 fms (631 m), bottom green mud, 12 May 1875, figured specimen (= *Acabaria nodosa* Prof. Hickson), old label book 2, p. 12.

#### Re-description.

BMNH 89.5.27.117 (Fig. 66b): Points with slightly bent spindles up to 0.20 mm long, distal end with more developed tubercles (Fig. 67a). Collaret with bent spindles up to 0.25 mm long, middle part with more developed tubercles (Fig. 67b). Tentacles with platelets, the larger ones crescent-shaped with irregular projections (Fig. 67c). These platelets are up to 0.10 mm long. Pharynx with straight spiny rods, up to 0.035 mm long (Fig. 67d). Coenenchyme with spindles (Fig. 67e) and unilaterally spinose spindles (Fig. 67f), 0.08-0.23 mm long, with simple tubercles. The calyces with additional clubs, up to 0.20 mm long (Fig. 67g).

#### Color.

Reddish brown, polyps yellow, axis yellowish-red; reddish nodes. Sclerites yellow, those of the polyps a bit paler, the smallest tentacle and the pharynx sclerites colorless.

#### Variation.

BMNH 89.5.27.118 (Figs 66c; 68) and BMNH 1947.3.22.6 (Fig. 66a) have similar color patterns, BMNH 1947.3.22.6 has wider coenenchymal sclerites (Fig. 69).

#### Distribution.

New Hebrides, Sagami Bay (Fig. 74).

#### Remarks.

Wright and Studer (1889) mentioned that the colony may have been about 130 mm in height and 60 to 80 mm in diameter. Only BMNH 89.5.27.117 has this size (Fig. 66b), BMNH 89.5.27.118 is much smaller (Fig. 66c), BMNH 1947.3.22.6 consists of only a few fragments (Fig. 66a). Based on these colony sizes we conclude that these authors used BMNH 89.5.27.117 for their description.

### Melithaea oyeni sp. n.

Taxon classificationAnimaliaAlcyonaceaMelithaeidae

http://zoobank.org/B848183E-DBDF-4F34-8F82-020D4B2ED3FE

**Figure 70.**
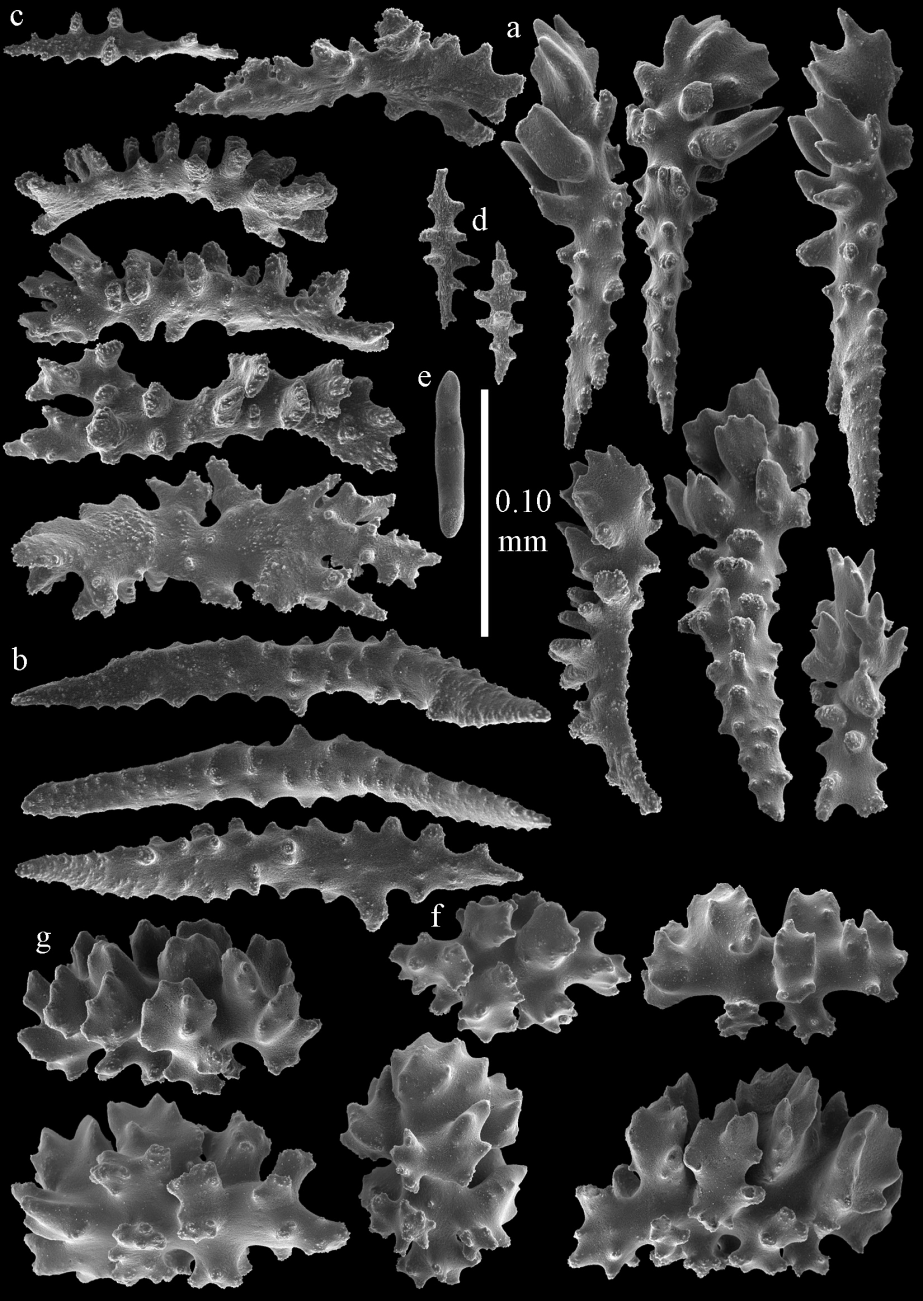
*Melithaea oyeni* sp. n., RMNH Coel. 41927; **a** point spindles **b** collaret spindles **c** tentacle sclerites **d** pharynx rods **e** axial rod **f** double disks **g** disk spindles.

**Figure 71.**
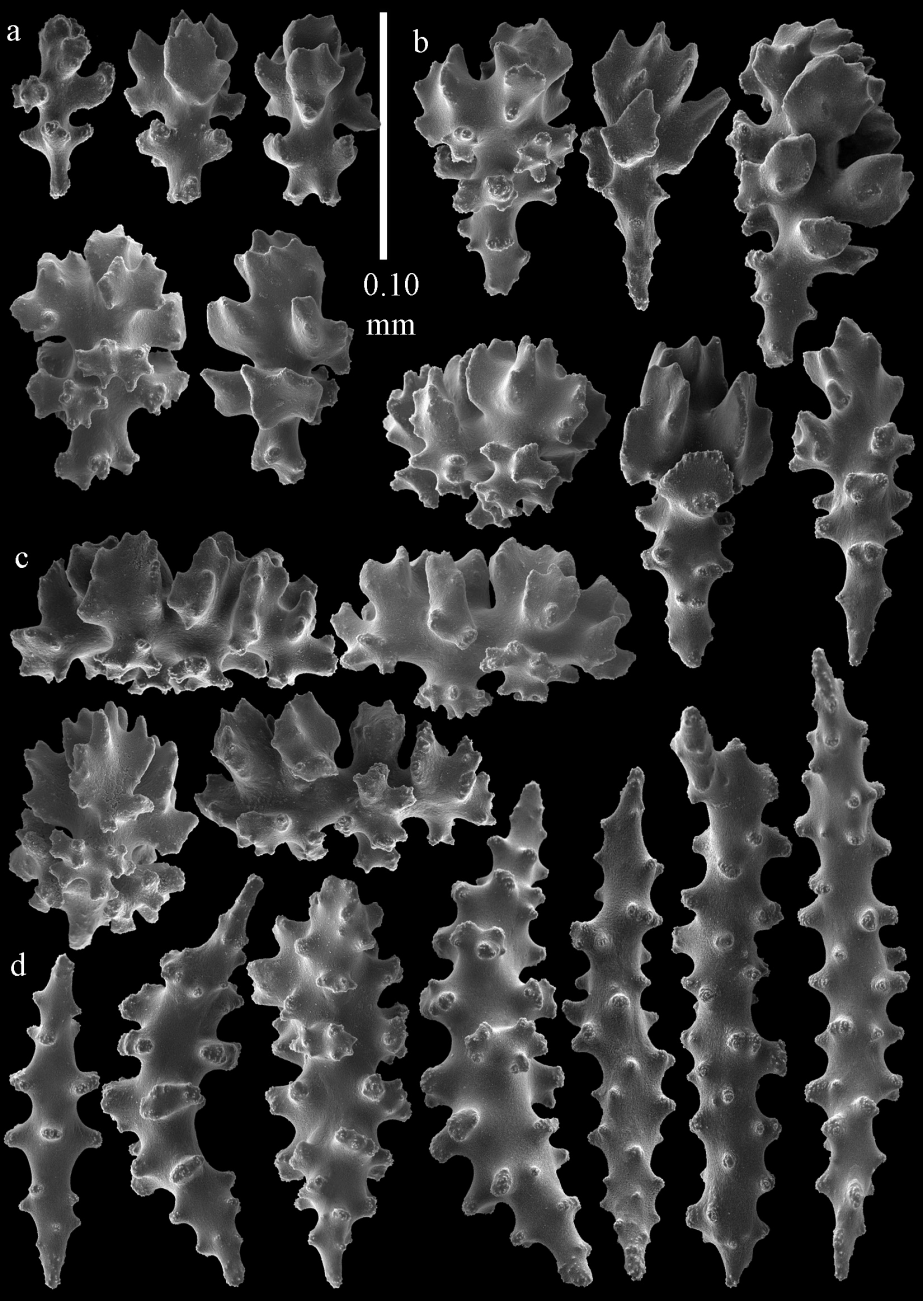
*Melithaea oyeni* sp. n., RMNH Coel. 41927; **a** clubs of coenenchyme **b** clubs of calyx **c** disk spindles **d** spindles.

Figures 66d , 70 , 71 , 74

#### Material examined.

Holotype **RMNH Coel. 41927 (AKM 408)**, Watari-se bank, off Izu Isls., 34°02.8620'N, 138°54.8090'E – 34°02.9190'N, 138°54.6810'E, 101.1-106.2 m, *R/V Tansei-maru*, KT04-06 cruise, St.WS-2, 1m ORI Dredge, coll. A.K. Matsumoto, 30 April 2004; paratype **RMNH Coel. 41928 (AKM 1606)**, Takase West, Izu Ridge, 34°26.5'N, 139°07.2'E, 104–127 m, *R/V Tansei-maru*, KT87-19, St. TW1, coll. S. Ohta, 8 December 1987.

#### Description.

The holotype is a 8 cm long fragment with holdfast (Fig. 66d). At the base the stem is 3 mm wide, the end branches are only 1 mm wide. The polyps are situated bilaterally on the branches, the calyces are dome-shaped, and most polyps are expanded. Points with slightly bent spindles up to 0.20 mm long, distal end with leaves (Fig. 70a). Collaret with bent spindles up to 0.30 mm long, middle part with more developed tubercles (Fig. 70b). Tentacles with platelets, the larger ones crescent-shaped with irregular projections (Fig. 70c). These platelets are up to 0.15 mm long. Pharynx with straight spiny rods, up to 0.05 mm long (Fig. 70d). Coenenchyme with predominantly capstans, double disks (Fig. 70f) and disk spindles (Figs 70g, 71c), 0.05–0.15 mm long, and small clubs (Fig. 71a), up to 0.10 mm long. Spindles are also present, 0.10-0.25 mm long, with simple tubercles (Fig. 71d). The calyces with additional clubs, up to 0.15 mm long (Fig. 71b).

#### Color.

Colony orange with yellow polyps, coenenchymal sclerites orange, polyp ones yellow.

#### Variation.

RMNH Coel. 41928 has similar sclerites and color.

#### Distribution.

Off the Izu Islands (Fig. 74)

#### Etymology.

The species is named after Mr. T.J.G.M. van Oyen (NBC) in appreciation of the many microscope slides he prepared for the second author.

#### Remarks.

The species mostly resembles *Melithaea abyssicola* but has overall somewhat larger sclerites, a difference difficult to notice when not having both species at hand. However, the double disks of *Melithaea oyeni* sp. n. are strikingly different, much wider than those of *Melithaea abyssicola* (compare Fig. 70f with Fig. 3b).

### Melithaea ryukyuensis sp. n.

Taxon classificationAnimaliaAlcyonaceaMelithaeidae

http://zoobank.org/40A013FB-10E2-4CD0-B899-F911524F59FF

**Figure 72.**
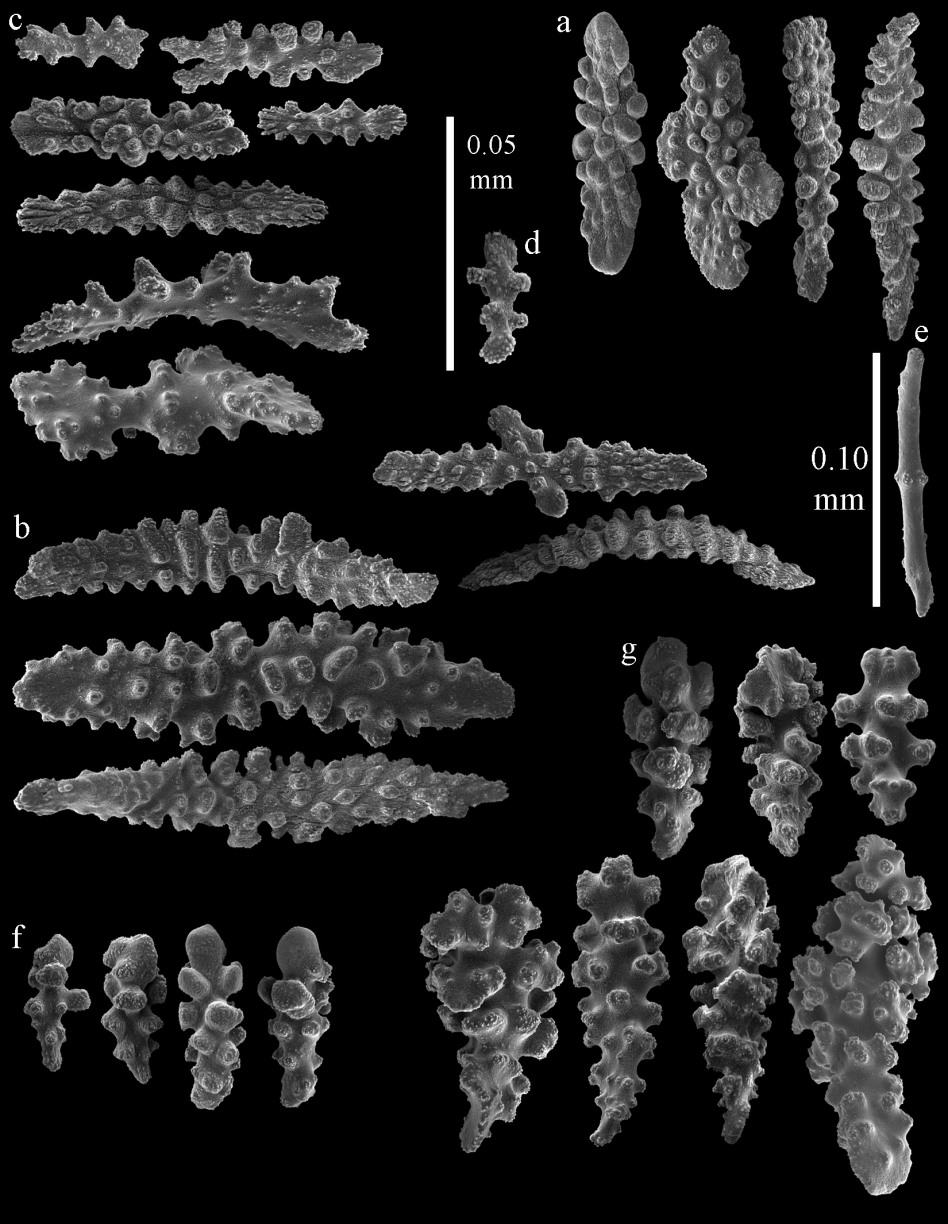
*Melithaea ryukyuensis* sp. n., UMUTZ-CnidG-32; **a** point spindles **b** collaret spindles **c** tentacle sclerites **d** pharynx rod **e** axial rod; **f** clubs of coenenchyme **g** clubs of calyx.

**Figure 73.**
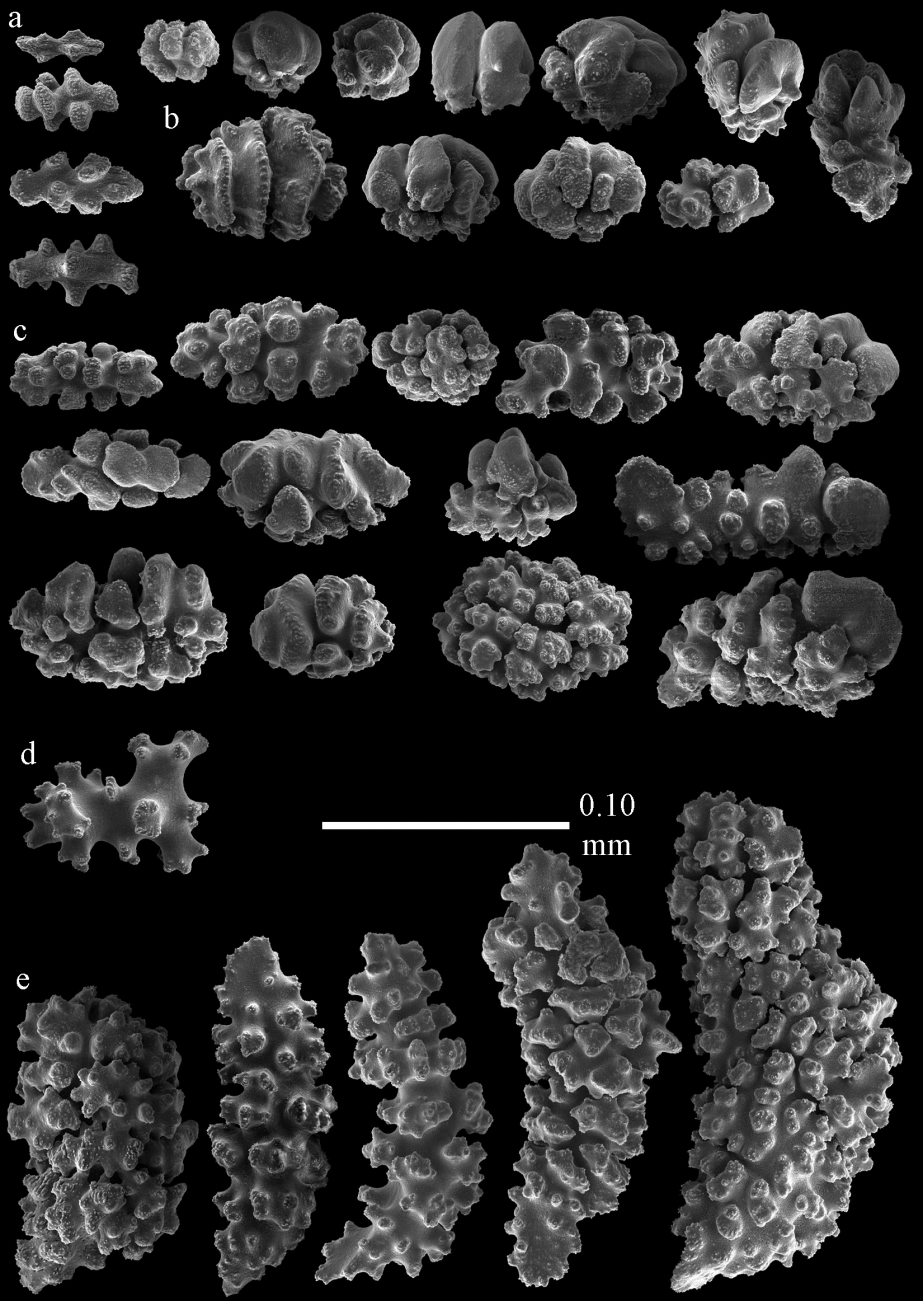
*Melithaea ryukyuensis* sp. n., UMUTZ-CnidG-32; **a** captans **b** double disks **c** disk spindles **d** irregular capstan **e** spindles.

**Figure 74.**
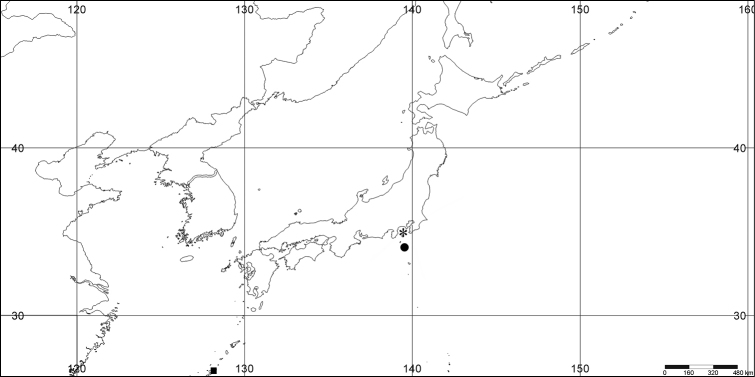
Distribution of *Melithaea nodosa* (*), *Melithaea oyeni* sp. n. (●), and *Melithaea ryukyuensis* sp. n. (■).

Figures 66e , 72 , 73 , 74

Melithaea sp. A: Aguilar-Hurtado et al. 2012: 62, fig. 5 (Okinawa).

#### Material examined.

Holotype **UMUTZ-CnidG-32**, Nakagusuku Bay, Okinawa Prefecture, Japan, diving, 16 April 1901; paratypes, **UMUTZ-CnidG-254**, same data as holotype; **UMZC I.36300**, S.W. Japan. T. Mizobuchi (purchased). Reg. Jan. 31.1903, 16.

#### Description.

The holotype is a 14.5 cm long fragment without holdfast (Fig. 66e). At the base the stem is 15 mm wide, the end branches are only 1 mm wide. The polyps are situated on one side of the colony, the calyces hardly project beyond the coenenchyme, and most polyps are retracted. Points with slightly bent spindles up to 0.10 mm long, distal end with more developed tubercles (Fig. 72a). Collaret with bent spindles up to 0.15 mm long, middle part with more developed tubercles (Fig. 72b). Tentacles with platelets, the larger ones crescent-shaped with irregular projections (Fig. 72c). These platelets are up to 0.10 mm long. Pharynx with straight spiny rods, up to 0.05 mm long (Fig. 72d). Coenenchyme with predominantly capstans (Fig. 73a, d), double disks (Fig. 73b) and disk spindles (Fig. 73c), 0.05–0.10 mm long, and small clubs with rounded heads, 0.05 mm long (Fig. 72f). Spindles are also present, 0.10–0.20 mm long, with complex tubercles (Fig. 73e). The calyces with additional clubs, up to 0.14 mm long (Fig. 72g). The axis has smooth and sparsely tuberculate rods (Fig. 72e).

#### Color.

Colony orange with yellow polyps. Part of calyx sclerites and all polyp sclerites yellow, all others orange.

#### Distribution.

Okinawa, Japan (Fig. 74).

#### Remarks.

The material of UMUTZ-CnidG-32 was probably collected during the Ryukyu (= Okinawa) expedition by K. Mitsukuri and I. Ikeda, in April, 1901. UMZC I.36300 is dried material. According to us this is *Melithaea* sp. A of Aquilar-Hurtado et al. (2012) as the sclerite images given by them resemble ours. *Melithaea ryukyuensis* mostly resembles *Melithaea abyssicola*, which has overall larger sclerites but much smaller spindles in the coenenchyme.

### Melithaea sagamiensis sp. n.

Taxon classificationAnimaliaAlcyonaceaMelithaeidae

http://zoobank.org/FD9CFBAD-842A-4D90-91D3-01DD11B687A4

Figures 75a–b , 76 , 77 , 78 , 79 , 80 , 81 , 86

#### Material examined.

Holotype **RMNH Coel. 41929 (AKM 840a)**, East of Jogashima Spur, 35°03.52'N, 139°37.43'E – 35°04.17'N, 139°37.52'E, 397–286 m, *R/V Tansei-maru*, KT07-31, st.8, coll. A.K. Matsumoto, 25 November 2007; paratypes: **ZMUC ANT-000657**, Sagami Bay, Okinose, 200 fms (286–366 m), Coll. Mortensen, 1 July 1914; **ZMUC ANT-000660**, same data as ZMUC ANT-000657; **RMNH Coel. 41930 (AKM 243)**, South of Mera-se-minami Knoll, Sagami Bay, 34°54.8'N, 139°39.7'E – 34°54.8'N, 139°39.9'E, 312–348 m, *R/V Shinyo-maru*, St. 10, coll. A.K. Matsumoto, 18 October 2003; **RMNH Coel. 41931 (AKM 244)**, same data as RMNH Coel. 41930; **RMNH Coel. 41932 (AKM 247)**, South of Mera-se Minami Knoll, 34°54.8'N, 139°39.7'E – 34°54.8'N, 139°39.9E, 315-365 m, *R/V Shinyo-maru*, coll. A.K. Matsumoto, 18 October 2003; **RMNH Coel. 41933 (AKM 593)**, Entrance of Otsuchi-bay, Iwate Prefecture, 39°21.858'N, 141°59.972'E, 65.6 m, *R/V Yayoi*, 1 m biological dredge, coll. A.K. Matsumoto, 12 September 2005; **RMNH Coel. 41934 (AKM 835)**, Off Sendai, Miyagi Prefecture, depth over 100 m, trawl, coll. Hagihara, June 2007; **RMNH Coel. 41935 (AKM 1605)**, West off Izu-Oshima, Sagami Sea, 410–440 m, *R/V Hakuho-maru*, KH-78-05, st. BS8, 2 m S.-A. beam trawl, coll. S. Ohta, 9 December 1978; **BMNH 62.7.16.62(61?)**, off Okushiri Is., 3 miles off shore, Sea of Japan, 41°59'N, 138°30'E, 25-30 feet.

#### Description.

**Figure 75.**
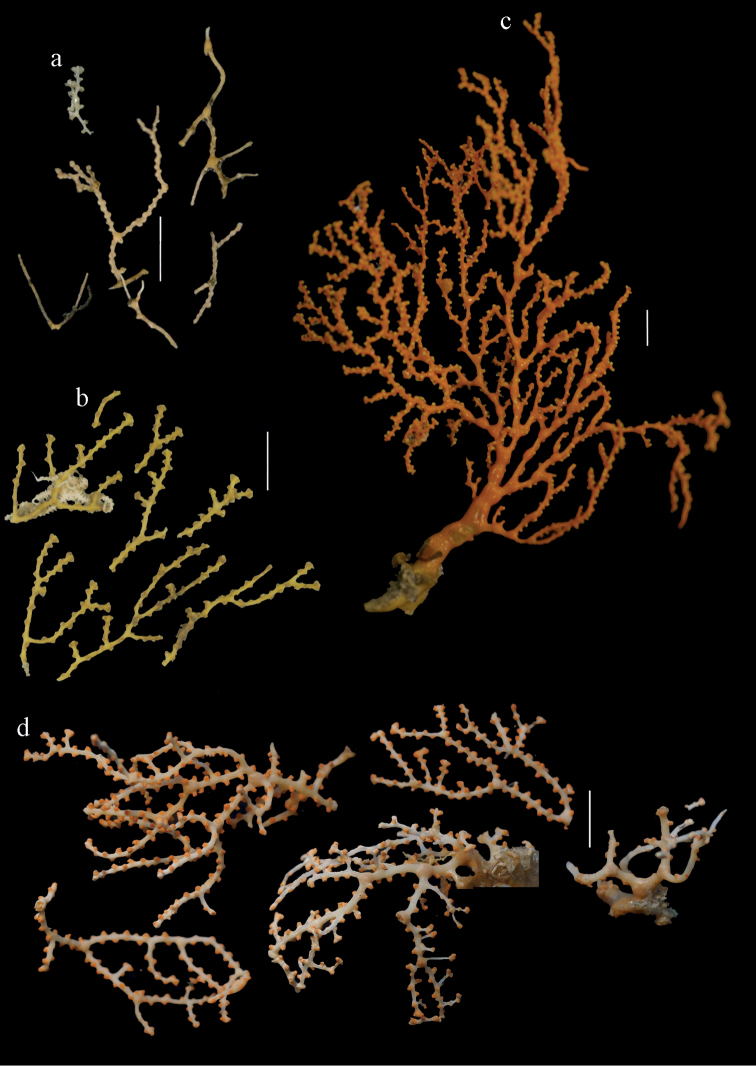
**a** *Melithaea sagamiensis* sp. n., RMNH Coel. 41929 **b** RMNH Coel. 41931 **c** *Melithaea satsumaensis* sp. n. RMNH Coel. 41936 **d** *Melithaea suensoni* sp. n. ZMUC ANT-000565.

**Figure 76.**
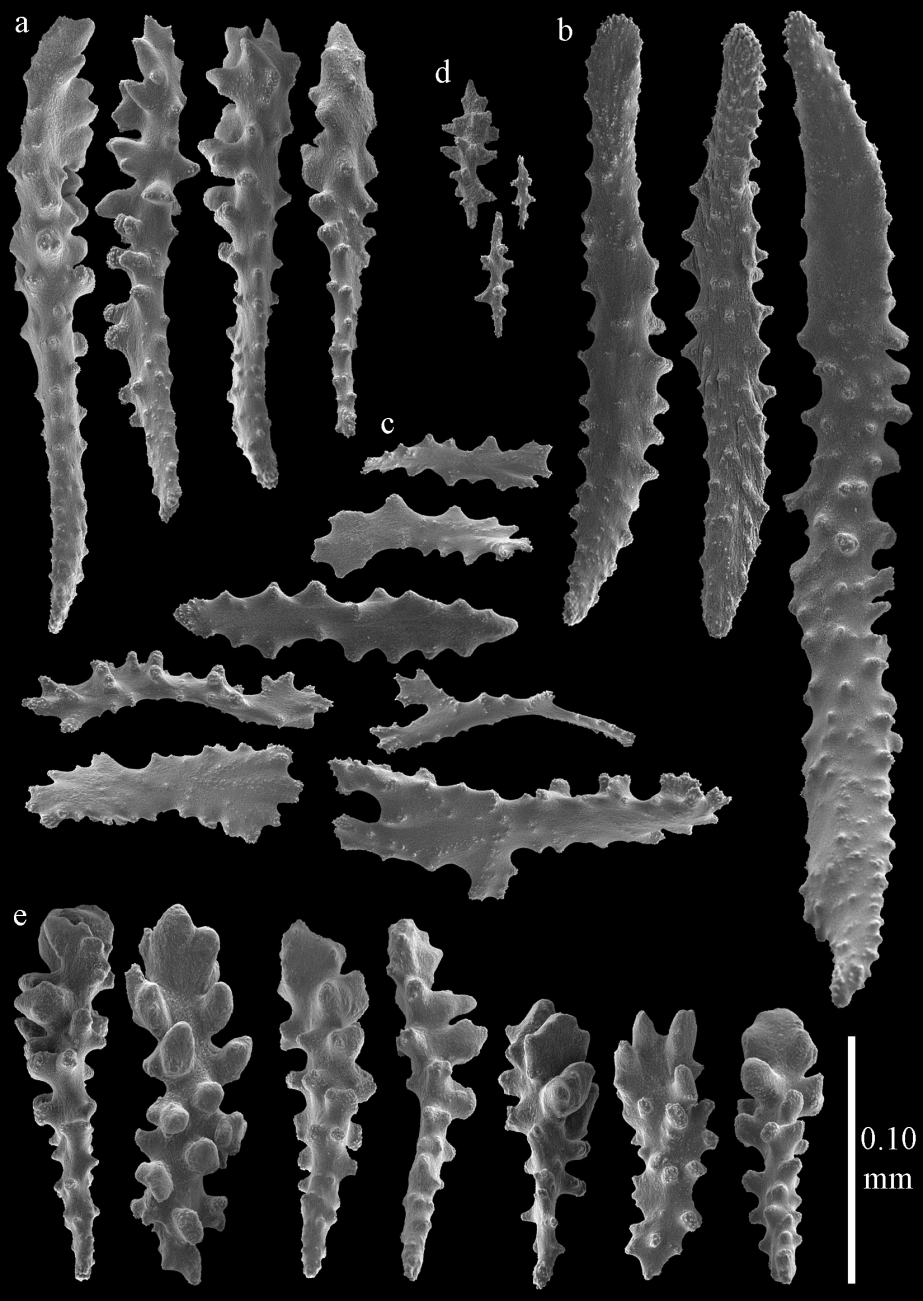
Sclerites of *Melithaea sagamiensis* sp. n., RMNH Coel. 41929; **a** point spindles **b** collaret spindles **c** tentacle sclerites **d** pharynx rods **e** clubs of calyx.

**Figure 77.**
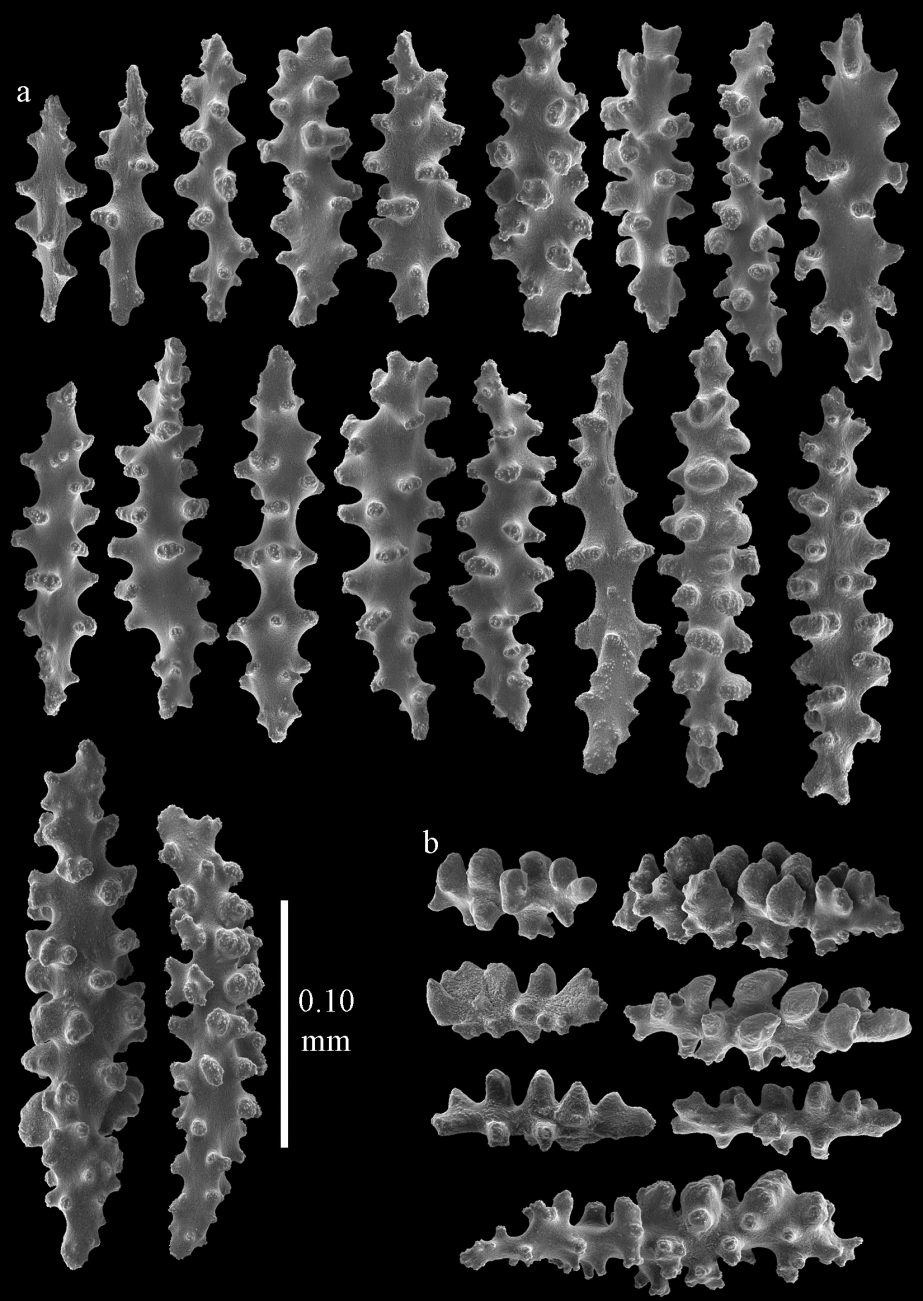
Sclerites of *Melithaea sagamiensis* sp. n., RMNH Coel. 41929; **a** spindles **b** unilaterally spinose spindles.

The holotype (RMNH Coel. 41929) consists of a number of branches probably belonging to one colony broken up while collecting (Fig. 75a). The largest fragment is 2.8 cm long. A holdfast or anastomoses are not present. The polyps are situated on one side of the colony. The calyces are dome-shaped, about 0.5 mm high and 1 mm wide; most polyps are retracted. Points with slightly bent spindles up to 0.25 mm long, distal end with more developed tubercles (Fig. 76a). Collaret with bent spindles up to 0.40 mm long, middle part with more developed tubercles (Fig. 76b). Tentacles with platelets, the larger ones crescent-shaped (Fig. 76c). These platelets are up to 0.15 mm long and have almost no tuberculation. Pharynx with straight spiny rods, up to 0.05 mm long (Fig. 76d). Coenenchyme with capstans 0.05-0.07 mm long, unilaterally spinose spindles (Fig. 77b), small clubs of the same length as the capstans, and spindles 0.10-0.20 mm long (Fig. 77a); all with simple tubercles. The calyces with additional leaf clubs, up to 0.15 mm long (Fig. 76e).

#### Color.

White, all sclerites colorless.

#### Variation.

**Figure 78.**
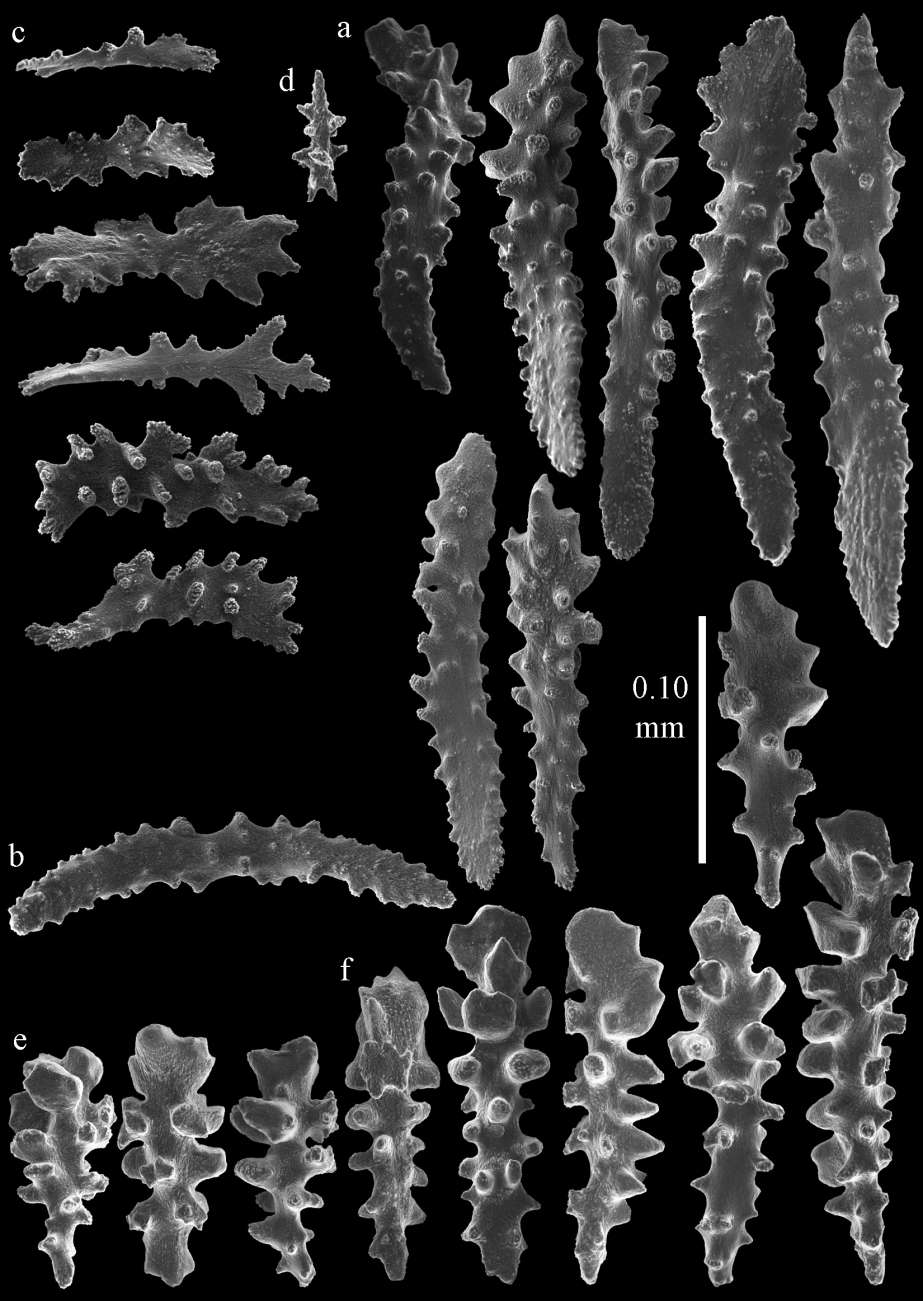
Sclerites of *Melithaea sagamiensis* sp. n., RMNH Coel. 41931; **a** point spindles **b** collaret spindle **c** tentacle sclerites **d** pharynx rod **e** clubs of coenenchyme **f** clubs of calyx.

**Figure 79.**
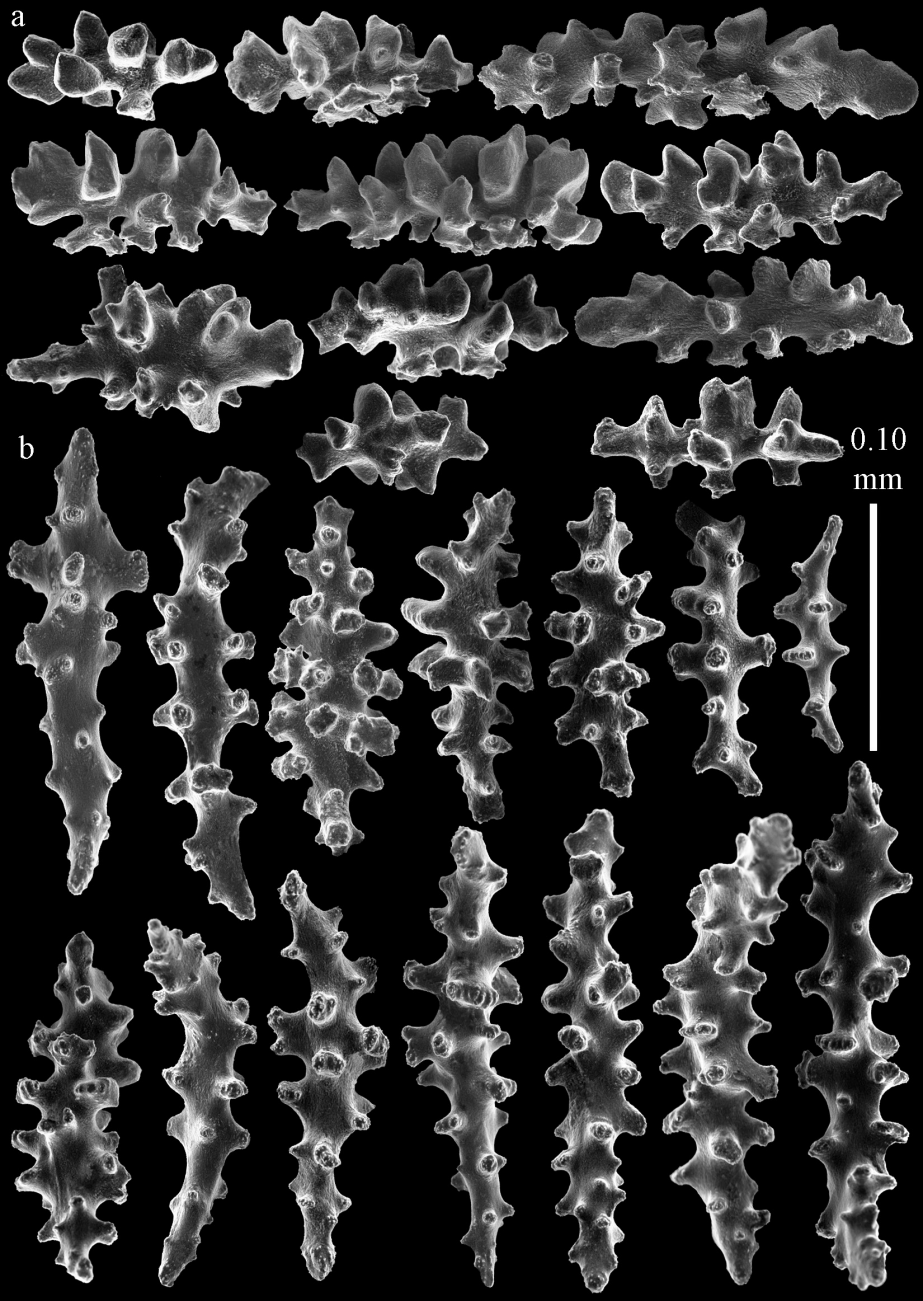
Sclerites of *Melithaea sagamiensis* sp. n., RMNH Coel. 41931; **a** unilaterally spinose spindles **b** spindles.

The paratypes are also fragmented. RMNH Coel. 41930, ZMUC ANT-000657 and ZMUC ANT-000651 are also white. ZMUC ANT-000660, RMNH Coel. 41931 and RMNH Coel. 41932 are yellow with yellow sclerites. BMNH 62.7.16.62(61?), RMNH Coel. 41934 and RMNH Coel. 41933 came from northern Japan, all three being red and from shallower depth. RMNH Coel. 41931 has somewhat more developed sclerites (Figs 78, 79).

#### Distribution.

Pacific side of Japan; Sagami Bay, off Sendai (Miyagi Prefecture), Otsuchi Bay (Sanriku, Iwate Prefecture); and Sea of Japan side, off Okushiri Is. (Fig. 86).

#### Etymology.

The species is named after the type locality, Sagami Bay.

#### Remarks.

**Figure 80.**
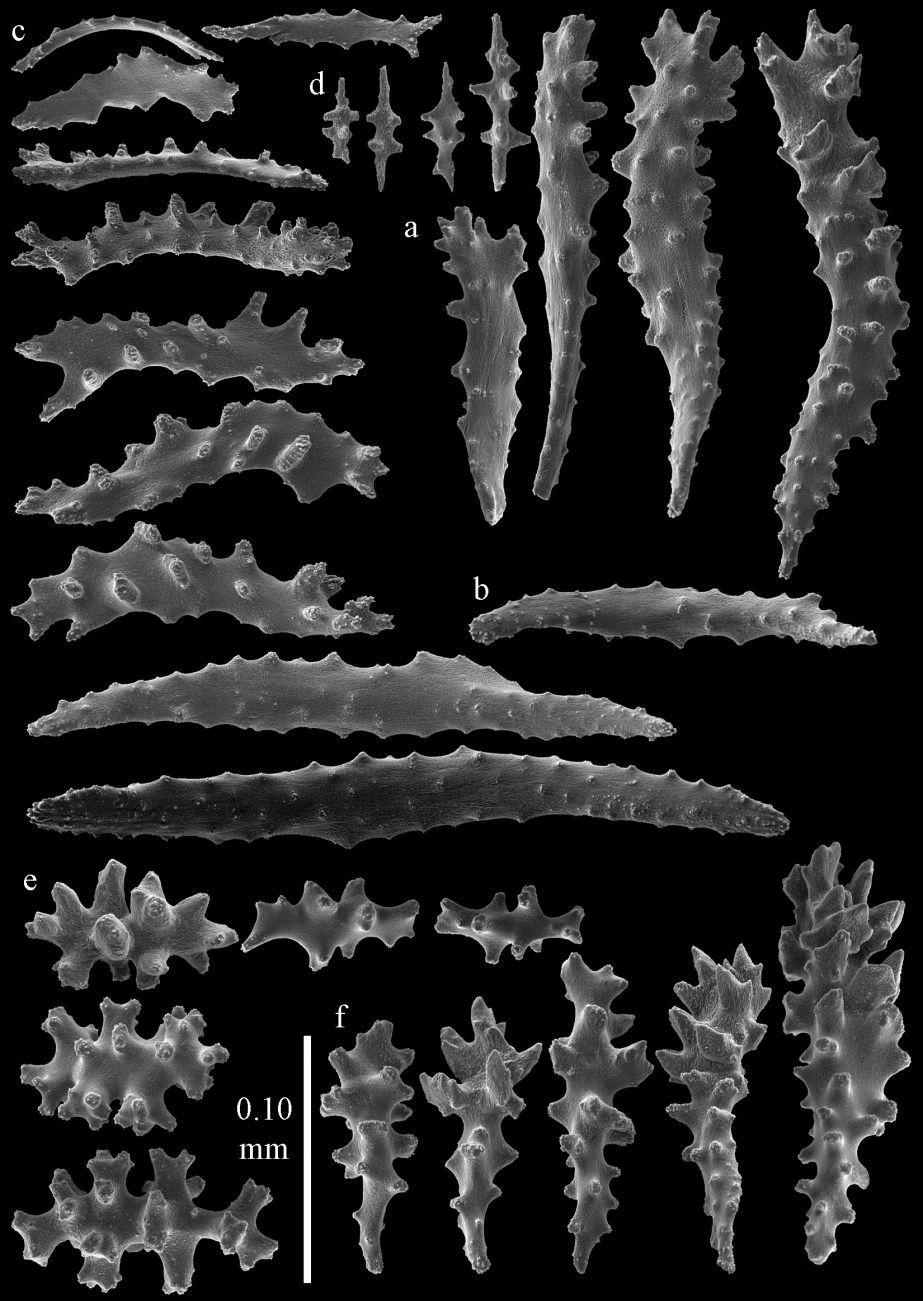
Sclerites of *Melithaea sagamiensis* sp. n., BMNH 62.7.16.62(61?); **a** point spindles **b** collaret spindles **c** tentacle sclerites **d** pharynx rod **e** capstans **f** clubs of calyx.

**Figure 81.**
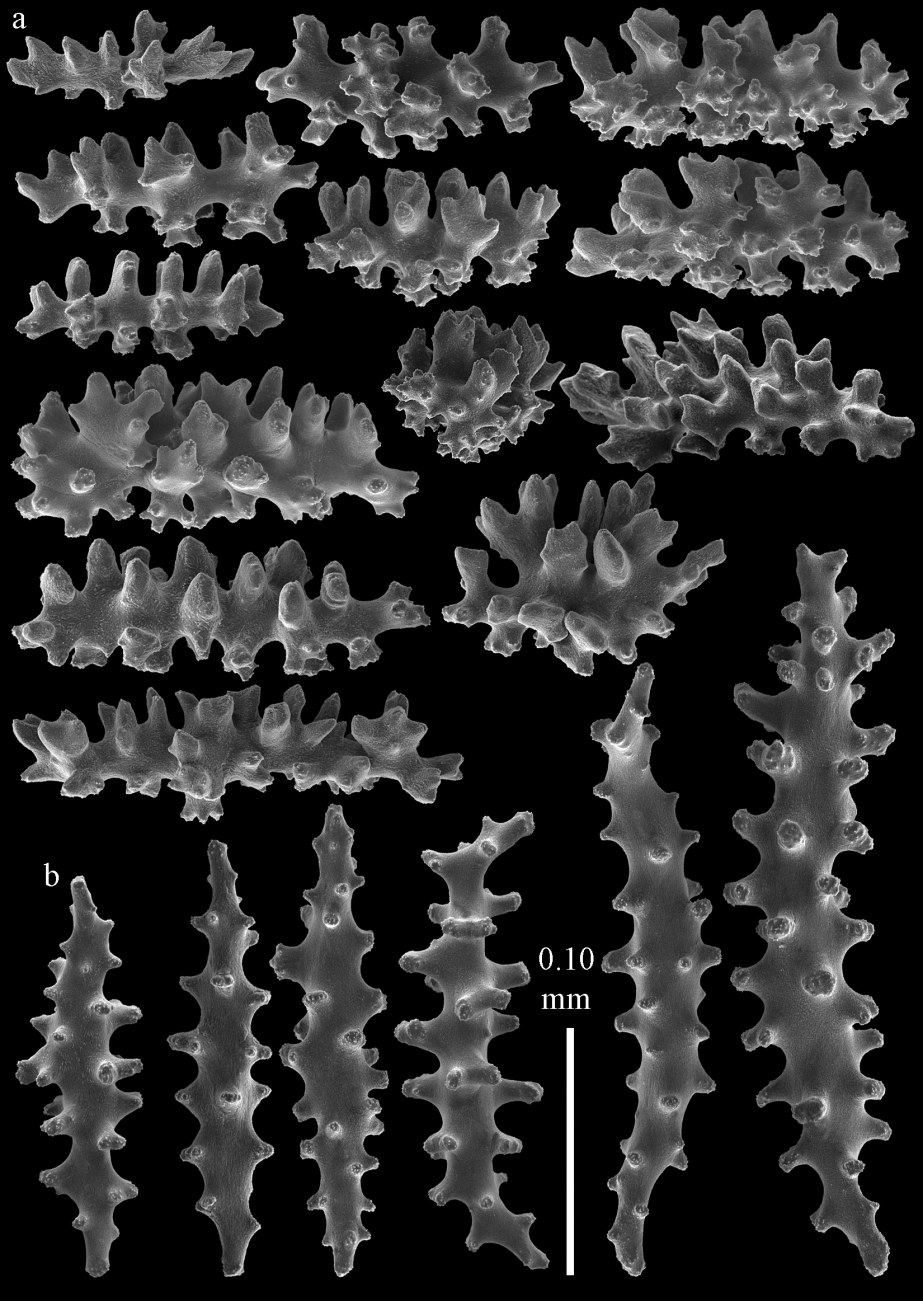
Sclerites of *Melithaea sagamiensis* sp. n., BMNH 62.7.16.62(61?); **a** unilaterally spinose spindles **b** spindles.

We included BMNH 62.7.16.62(61?) in *Melithaea sagamiensis* sp. n. despite its slightly different sclerites (Figs 80, 81). We were not certain about the collection number, hence the “62(61)?”.

### Melithaea satsumaensis sp. n.

Taxon classificationAnimaliaAlcyonaceaMelithaeidae

http://zoobank.org/5A1437CD-CE23-4E8F-B4AE-912F92548718

**Figure 82.**
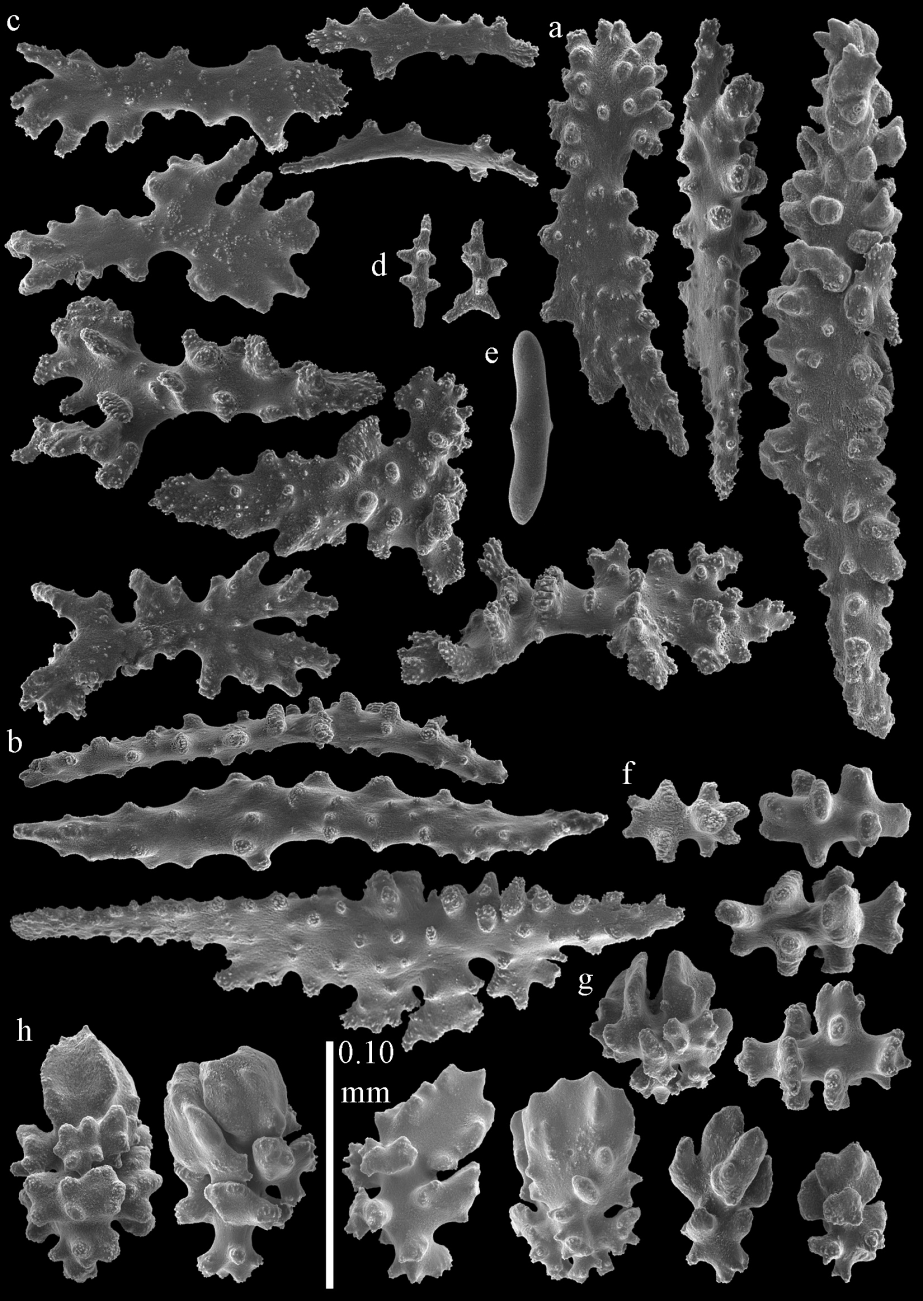
Sclerites of *Melithaea satsumaensis* sp. n., RMNH Coel. 41936; **a** point spindles **b** collaret spindles **c** tentacle sclerites **d** pharynx rods **e** axial rod **f** capstans **g** double disks **h** clubs of coenenchyme.

**Figure 83.**
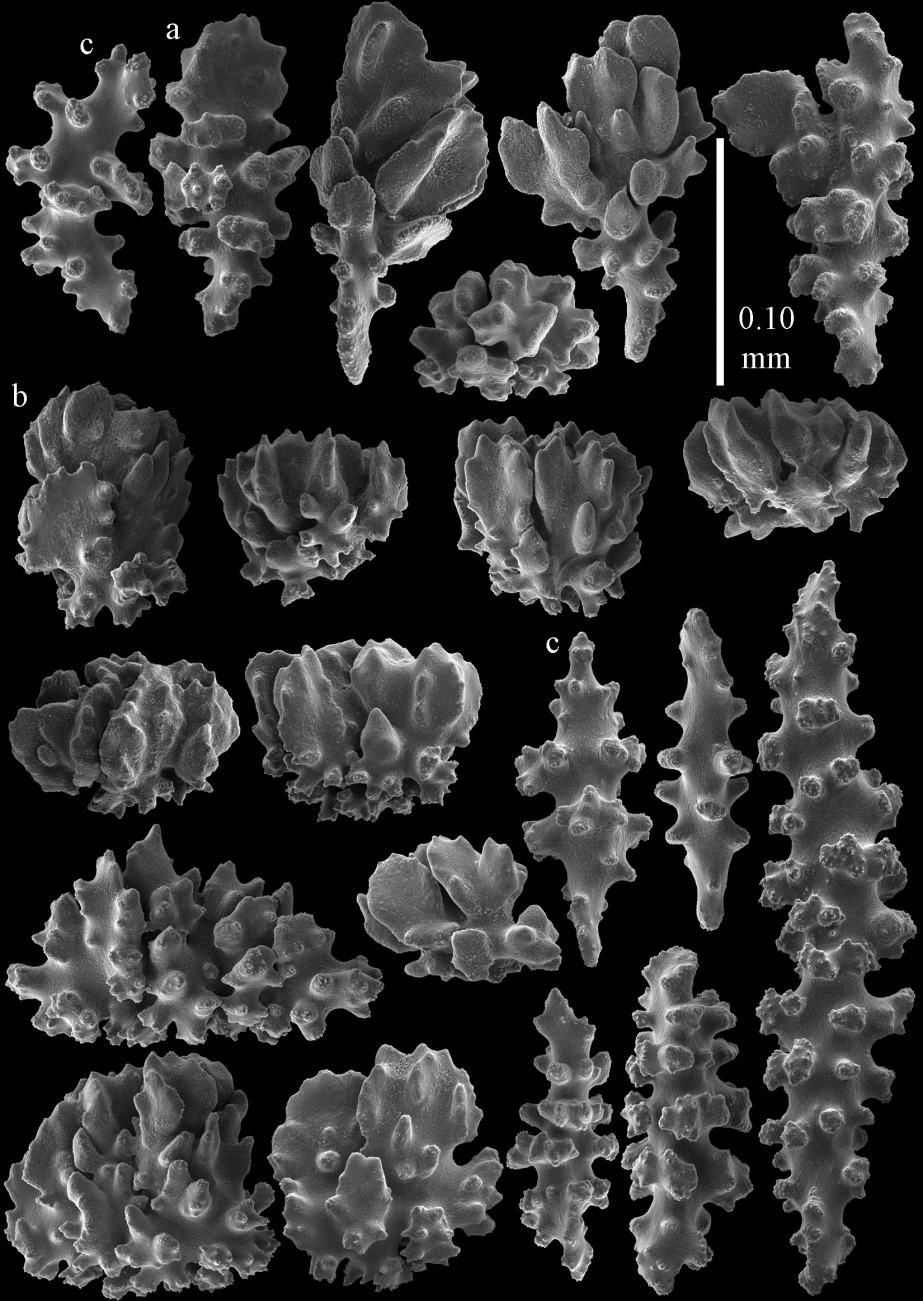
Sclerites of *Melithaea satsumaensis* sp. n., RMNH Coel. 41936; **a** clubs of calyx **b** unilaterally foliate spheroids **c** spindles.

Figures 75c , 82 , 83 , 86

#### Material examined.

Holotype **RMNH Coel. 41936 (AKM 743)**, Off Sata-misaki Cape, Kagoshima Prefecture, Japan, 30°56.0025'N, 130°44.2299'E – 30°56.2953'N, 130°43.3981'E, 116-120 m, *R/V Tansei-maru*, KT07-1 cruise, St. SM-1, Chain Bag Dredge, coll. A.K. Matsumoto, 23 February 2007.

#### Description.

The holotype is a 16.5 cm long colony with holdfast (Fig. 75c). At the base the stem is 10 mm wide, the end branches are only 1 mm wide. On the lower half of the colony the polyps are situated on one side of the colony; the upper part has polyps all around the branches. The calyces are dome-shaped, and most polyps are expanded. Points with slightly bent spindles up to 0.20 mm long, distal end with more developed tubercles (Fig. 82a). Collaret with bent spindles up to 0.30 mm long, middle part with more developed tubercles (Fig. 82b). Tentacles with platelets, the larger ones crescent-shaped with irregular projections (Fig. 82c). These platelets are up to 0.15 mm long. Pharynx with straight spiny rods, up to 0.05 mm long (Fig. 82d). Coenenchyme with predominantly capstans (Fig. 82f), double disks (Fig. 82g), and unilaterally foliate spheroids (Fig. 83b), 0.05–0.15 mm long, and small clubs (Fig. 82h), up to 0.10 mm long. Spindles are also present, 0.10-0.25 mm long, with simple or complex tubercles (Fig. 83c). The calyces with additional clubs, up to 0.15 mm long (Fig. 83a). The axis has smooth and sparsely tuberculate rods (Fig. 82e).

#### Color.

Colony orange with yellow polyps, coenenchymal sclerites orange, polyp ones yellow.

#### Distribution.

Off Cape Sata misaki, Kagoshima Prefecture (Fig. 86).

#### Etymology.

The species is named after the type locality, Satsuma, the old name of Kagoshima Prefecture.

#### Remarks.

This species is unique by its unilaterally foliate spheroids and spindles with complex tubercles.

### Melithaea suensoni sp. n.

Taxon classificationAnimaliaAlcyonaceaMelithaeidae

http://zoobank.org/75964162-7435-4643-BE6B-A8837CA1C91D

**Figure 84.**
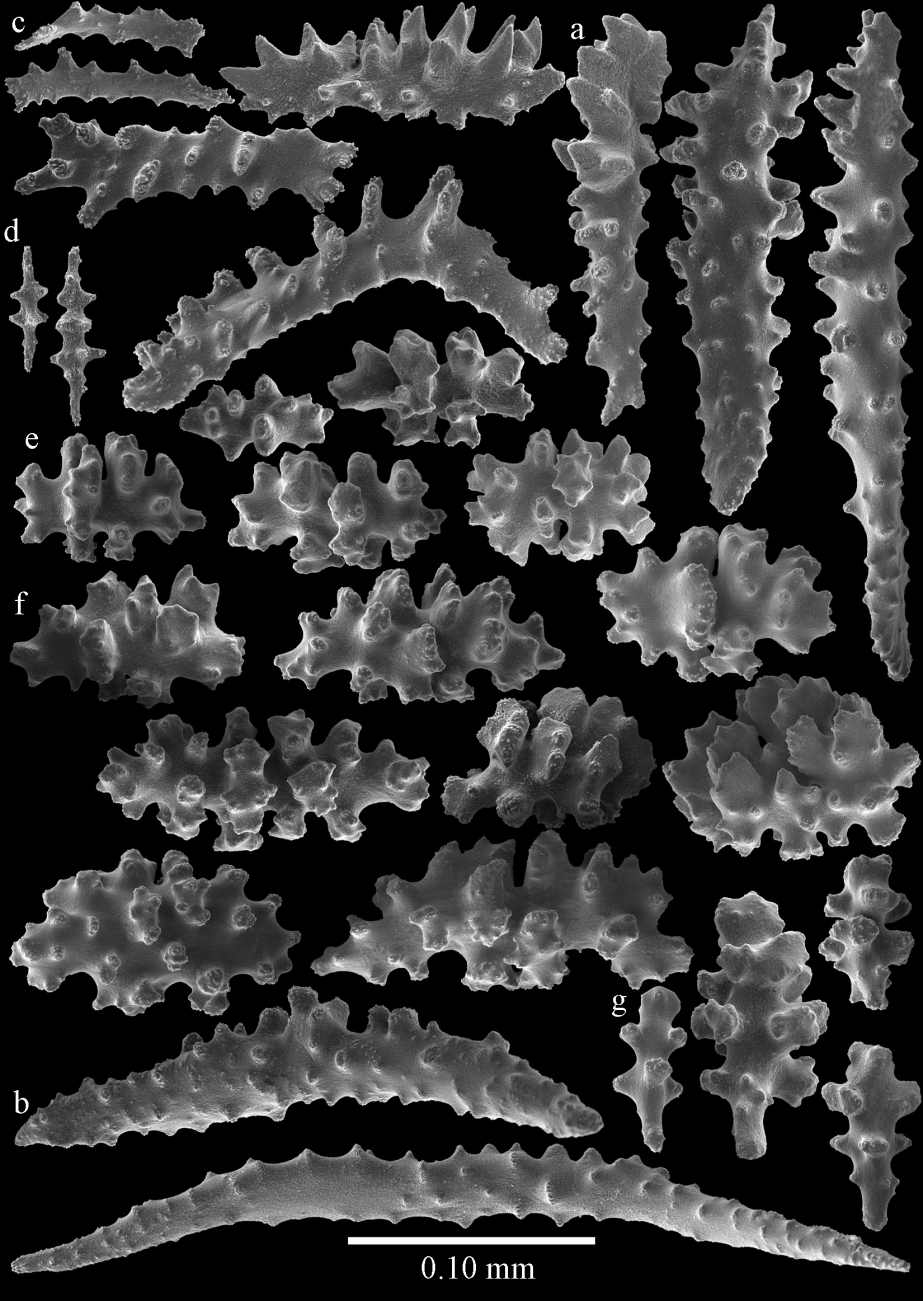
Sclerites of *Melithaea suensoni* sp. n., ZMUC ANT-000565; **a** point spindles **b** collaret spindles **c** tentacle sclerites **d** pharynx rods **e** capstans **f** disk spindles **g** clubs of coenenchyme.

**Figure 85.**
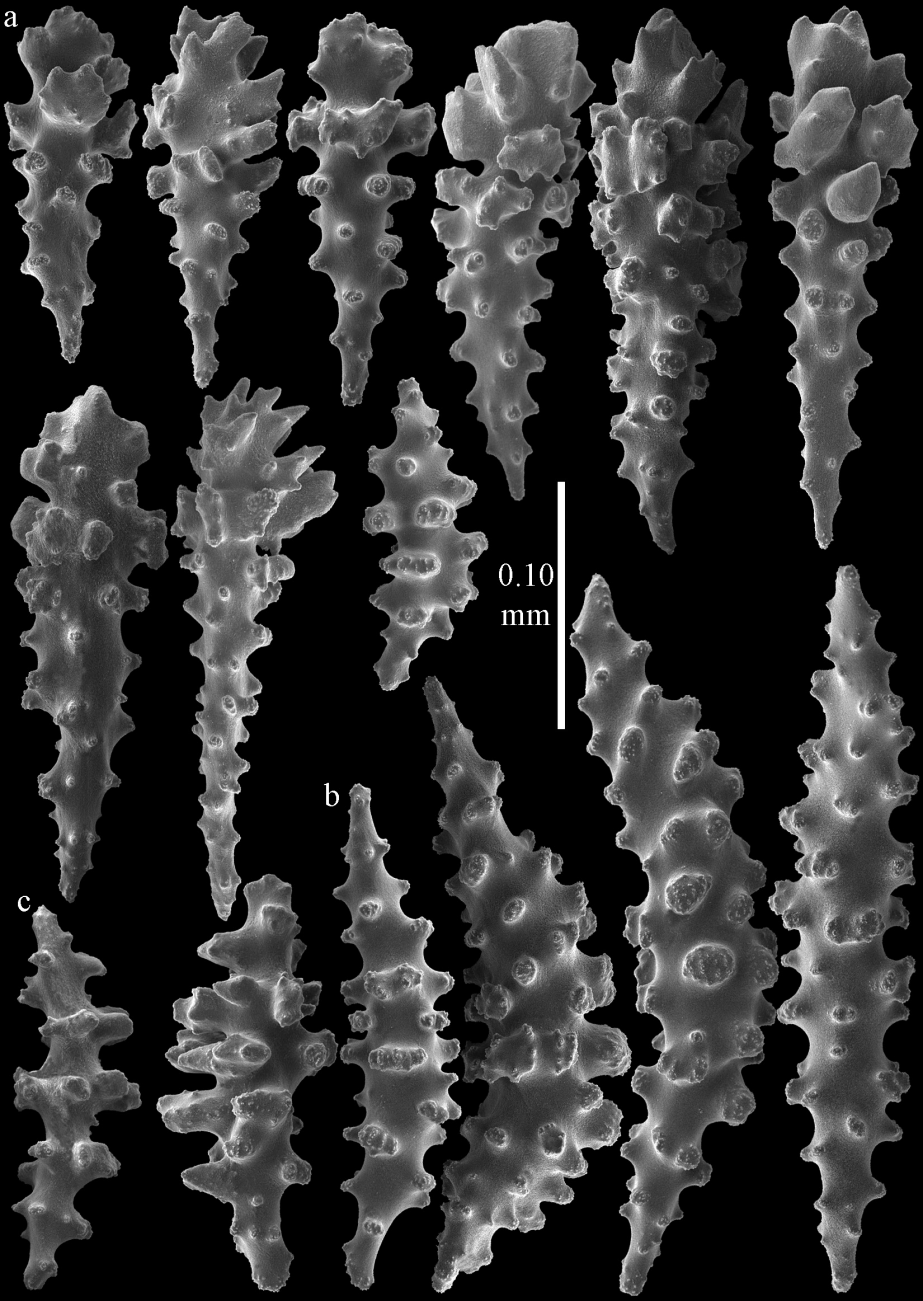
Sclerites of *Melithaea suensoni* sp. n., ZMUC ANT-000565; **a** clubs of calyx **b** spindles **c** disk spindles.

**Figure 86.**
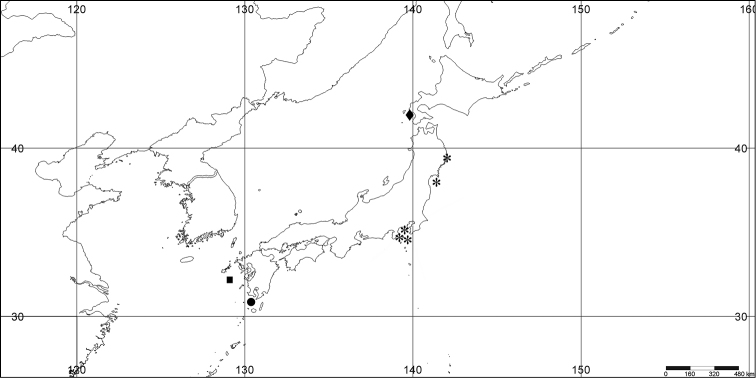
Distribution of *Melithaea sagamiensis* sp. n. (*), BMNH 62.7.16.62(61?) (♦), *Melithaea satsumaensis* sp. n. (●), and *Melithaea suensoni* sp. n. (■).

Figures 75d , 84 , 85 , 86

#### Material examined.

Holotype **ZMUC ANT-000565**, off Nagasaki, 32°22'N, 128°42'E, 170 fms (311 m), 25 December 1900, coll. Suenson.

#### Description.

Colony branched in one plane, with slender branches (Fig. 75d). Points with slightly bent spindles up to 0.30 mm long, distal end with more developed tubercles or leaves (Fig. 84a). Collaret with bent spindles up to 0.35 mm long, middle part with more developed tubercles (Fig. 84b). Tentacles with platelets, the larger ones crescent-shaped with irregular projections (Fig. 84c). These platelets are up to 0.15 mm long. Pharynx with straight spiny rods, up to 0.05 mm long (Fig. 84d). Coenenchyme with capstans (Fig. 84e) and disk spindles (Figs 84f, 85c), 0.05–0.15 mm long, and small clubs of similar length (Fig. 84g). Spindles are also present, 0.15–0.30 mm long, with simple or complex tubercles (Fig. 85b). The calyces with additional clubs, up to 0.20 mm long (Fig. 85a).

#### Color.

White with orange calyces and polyps. Sclerites of calyces and polyps faint pink, all others colorless.

#### Distribution.

Off Nagasaki, East China Sea (Fig. 86).

#### Etymology.

The species is named after the collector, E. Suenson, who belonged to the Telegraph company Great Nordic Ltd. (Store Nordiske), established in 1869 at Denmark (Matsumoto 2014, 2015).

#### Remarks.

This species resembles *Melithaea sagamiensis* sp. n., but differs by its thicker coenenchymal spindles.

### Melithaea tanseii sp. n.

Taxon classificationAnimaliaAlcyonaceaMelithaeidae

http://zoobank.org/8D2D051B-0B90-4BF4-9716-7C83B5652A47

**Figure 87.**
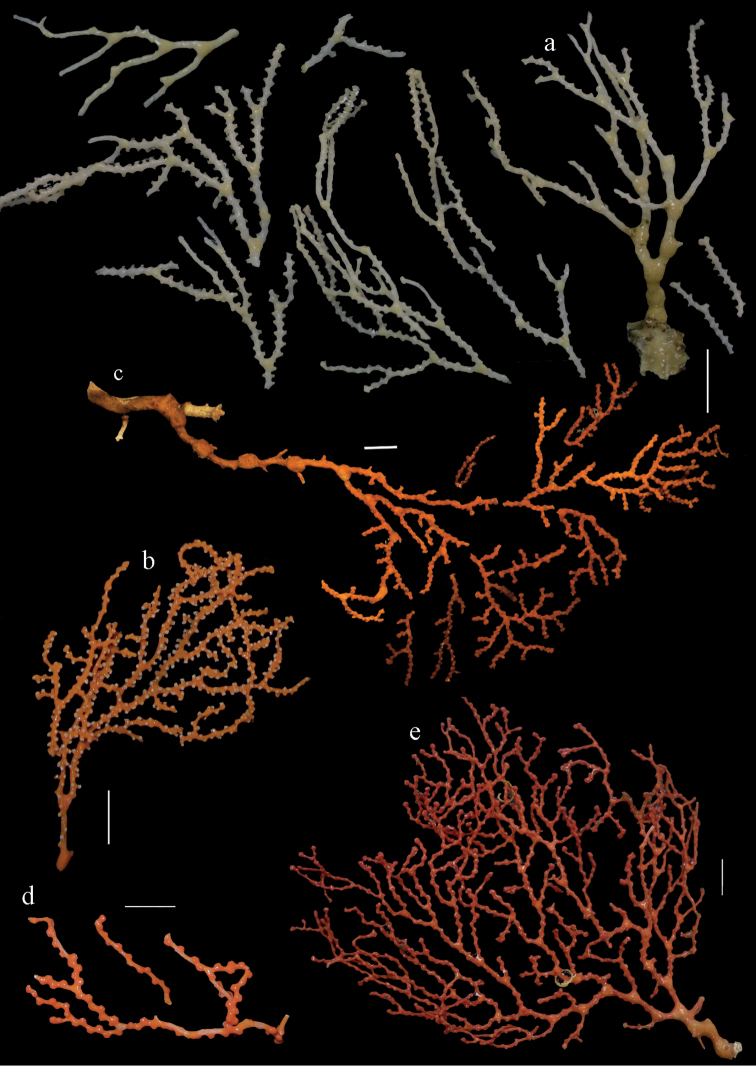
**a** *Melithaea tanseii* sp. n., holotype RMNH Coel. 41937 **b** *Melithaea tenuis* syntype ZMB 5799 **c** *Melithaea tokaraensis* sp. n. RMNH Coel. 41941 **d** *Melithaea undulata* holotype ZMB 5803 **e** RMNH Coel. 42030.

**Figure 88.**
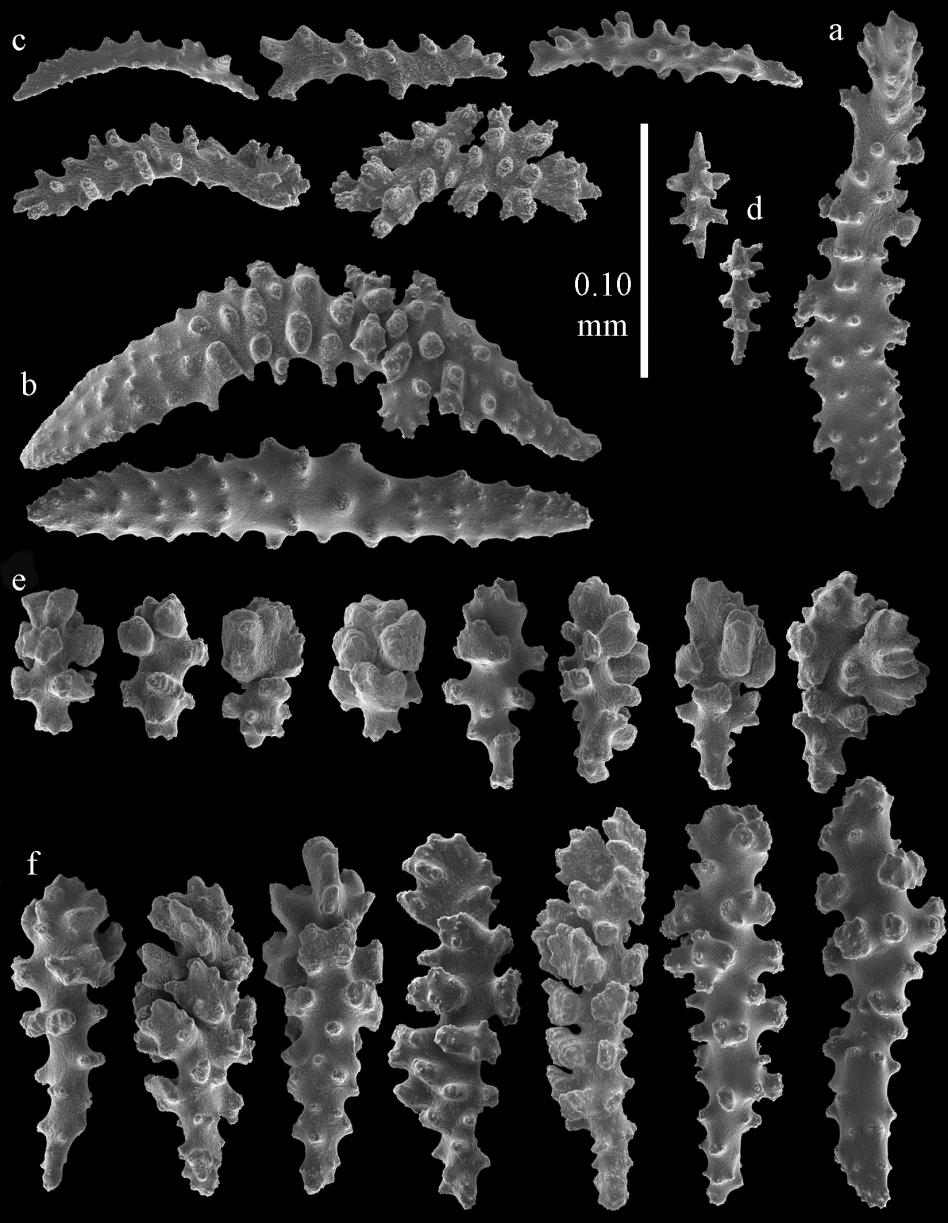
Sclerites of *Melithaea tanseii* sp. n., RMNH Coel. 41937; **a** point spindle **b** collaret spindles **c** tentacle sclerites **d** pharynx rods **e** clubs of coenenchyme **f** clubs of calyx.

**Figure 89.**
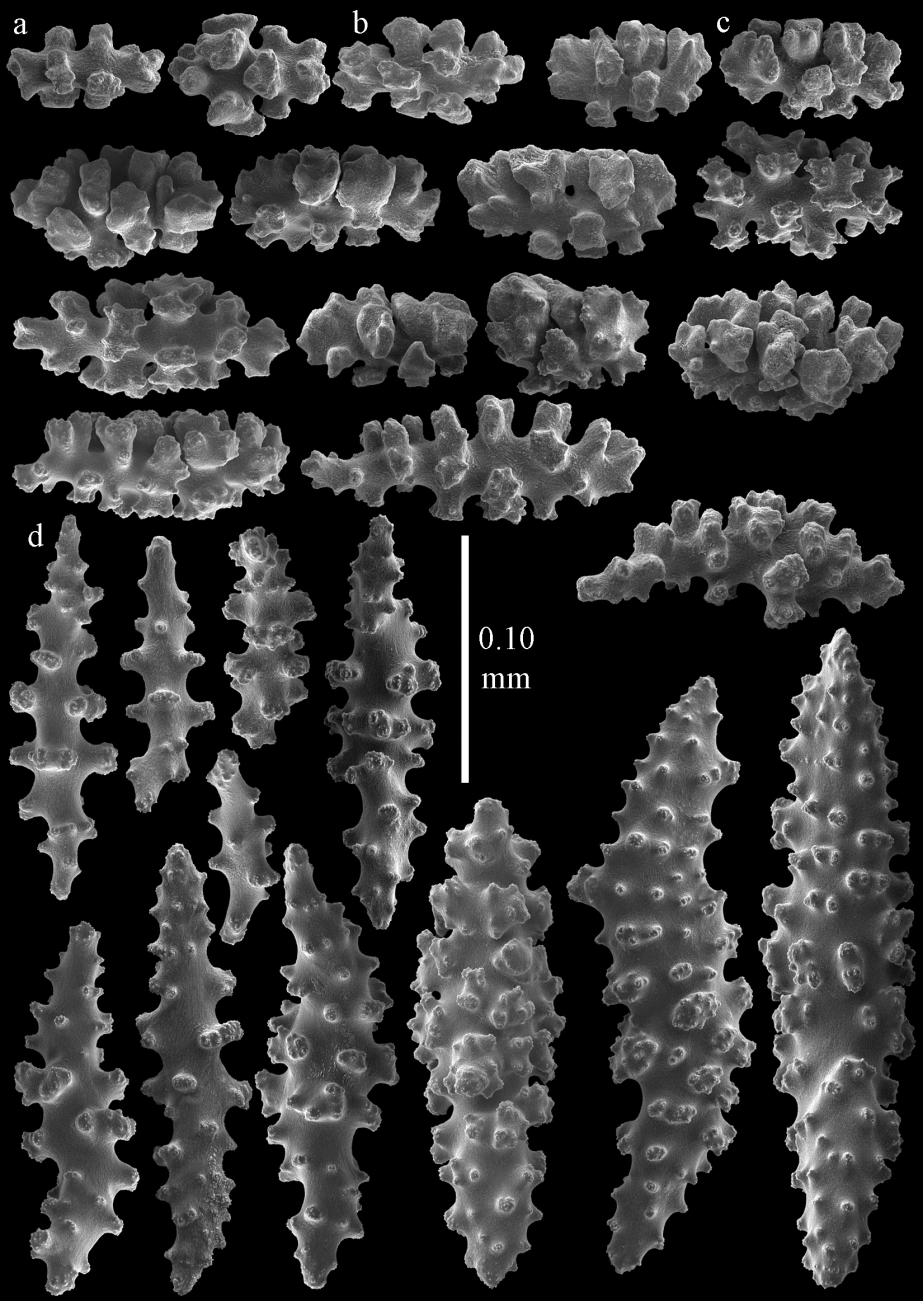
Sclerites of *Melithaea tanseii* sp. n., RMNH Coel. 41937; **a** capstans **b** unilaterally foliate capstans **c** unilaterally foliate spindles **d** spindles.

Figures 87a , 88 , 89 , 98

#### Material examined.

Holotype **RMNH Coel. 41937 (AKM 948)**, Toshima Is., Izu Isls., 34°33.1102'N, 139°17.4102'E – 34°33.6524'N, 143 m, *R/V Tansei-maru*, KT07-31 (Kuramochi leg.) St. 22 (L-3-100), coll. A.K. Matsumoto; paratype: **RMNH Coel. 41938 (AKM 941)** Toshima Is., Izu Isl, 34°34.4640'N, 139°18.3760'E – 34°33.5601'N, 139°17.8631'E, 152–198 m, *R/V Tansei-maru*, KT07-31 (Kuramochi leg.) St. 21 (L-3-200), coll. A.K. Matsumoto.

#### Description.

The holotype (RMNH Coel. 41937) consists of a large number of branches probably belonging to one colony broken up while collecting (Fig. 87a). The largest fragment, with the holdfast, is 6.5 cm long. The stem is 10 mm long and 3 mm wide; branching is in one plane. A few anastomoses are present. The polyps are situated on one side of the colony. The calyces are dome-shaped, about 0.5 mm high and 1 mm wide. Many polyps are expanded. Points with slightly bent spindles up to 0.20 mm long, distal end with more developed tubercles (Fig. 88a). Collaret with bent spindles up to 0.25 mm long, middle part with more developed tubercles (Fig. 88b). Tentacles with platelets, the larger ones crescent-shaped (Fig. 88c). These platelets are up to 0.15 mm long. Pharynx with straight spiny rods, up to 0.05 mm long (Fig. 88d). Coenenchyme with capstans (Fig. 89a) 0.05–0.07 mm long, several slightly unilaterally foliate (Fig. 89b), unilaterally foliate spindles (Fig. 89c), small clubs of the same length as the capstans (Fig. 88e); spindles 0.10–0.25 mm long (Fig. 89d); all with simple tubercles. The calyces with additional clubs, up to 0.20 mm long (Fig. 88f).

#### Color.

Colony white with the larger yellowish nodes shining through the coenenchyme. All sclerites colorless.

#### Distribution.

Izu Isls (Fig. 98).

**Etymology.** The species is named after the *R/V Tansei-maru*.

#### Remarks.

The paratype (RMNH Coel. 41938) is just a fragment of colony, only three cm long with characters alike those of the holotype. The species mostly resembles *Melithaea tenuis* but differs in having much wider coenenchymal spindles, up to 0.07 mm wide, twice as wide as in *Melithaea tenuis*, in which they are only 0.03 mm wide. A few spindles in *Melithaea tenuis* are wider, mainly caused by the spindles having bigger tubercles. Another difference is the slightly bigger capstans in the new species, but this is only noticeable when having both species.

### Melithaea tenuis

Taxon classificationAnimaliaAlcyonaceaMelithaeidae

(Kükenthal, 1908)

**Figure 90.**
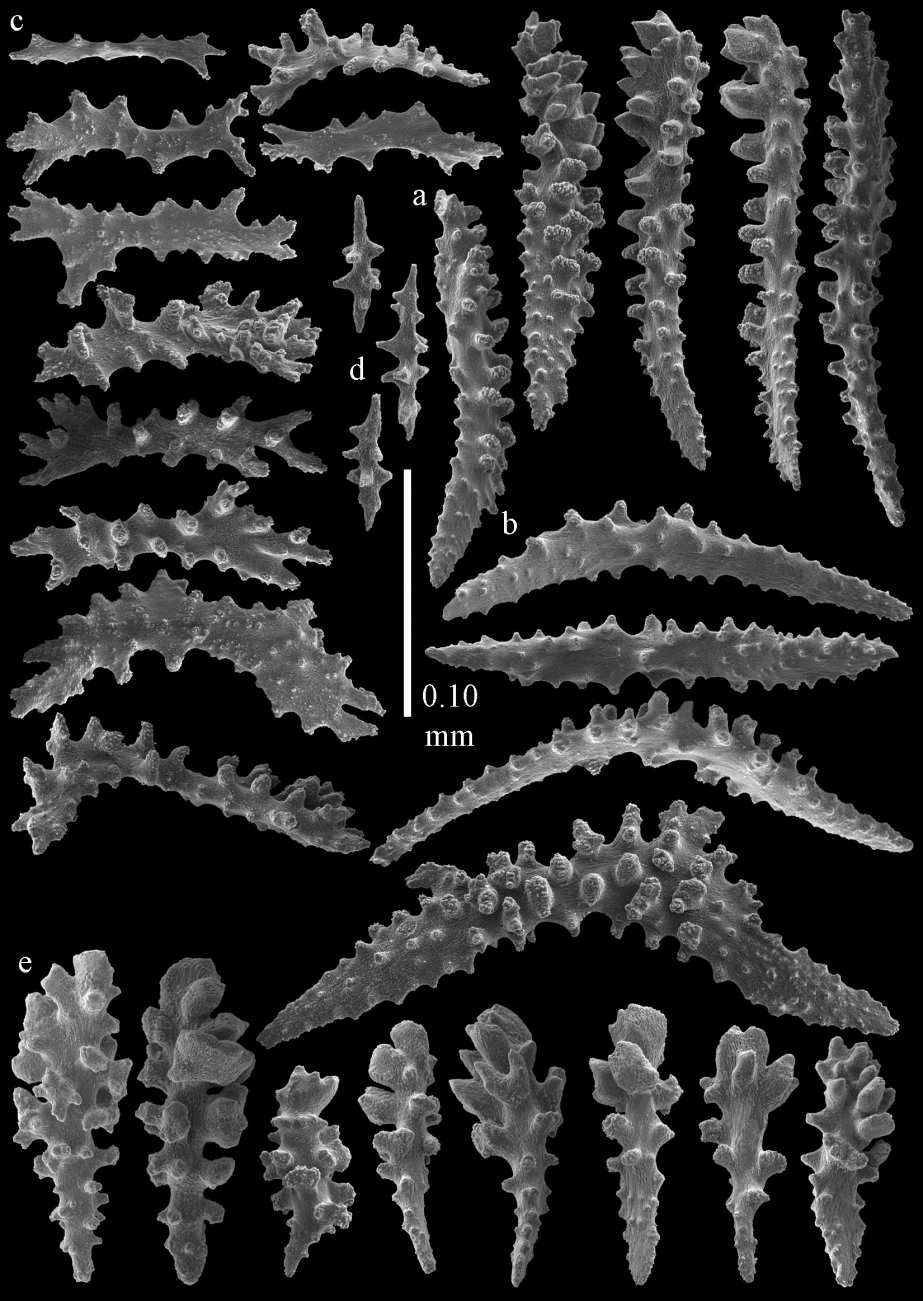
Sclerites of *Melithaea tenuis*, ZMB 5799; **a** point spindles **b** collaret spindles **c** tentacle sclerites **d** pharynx rods **e** clubs of calyx.

**Figure 91.**
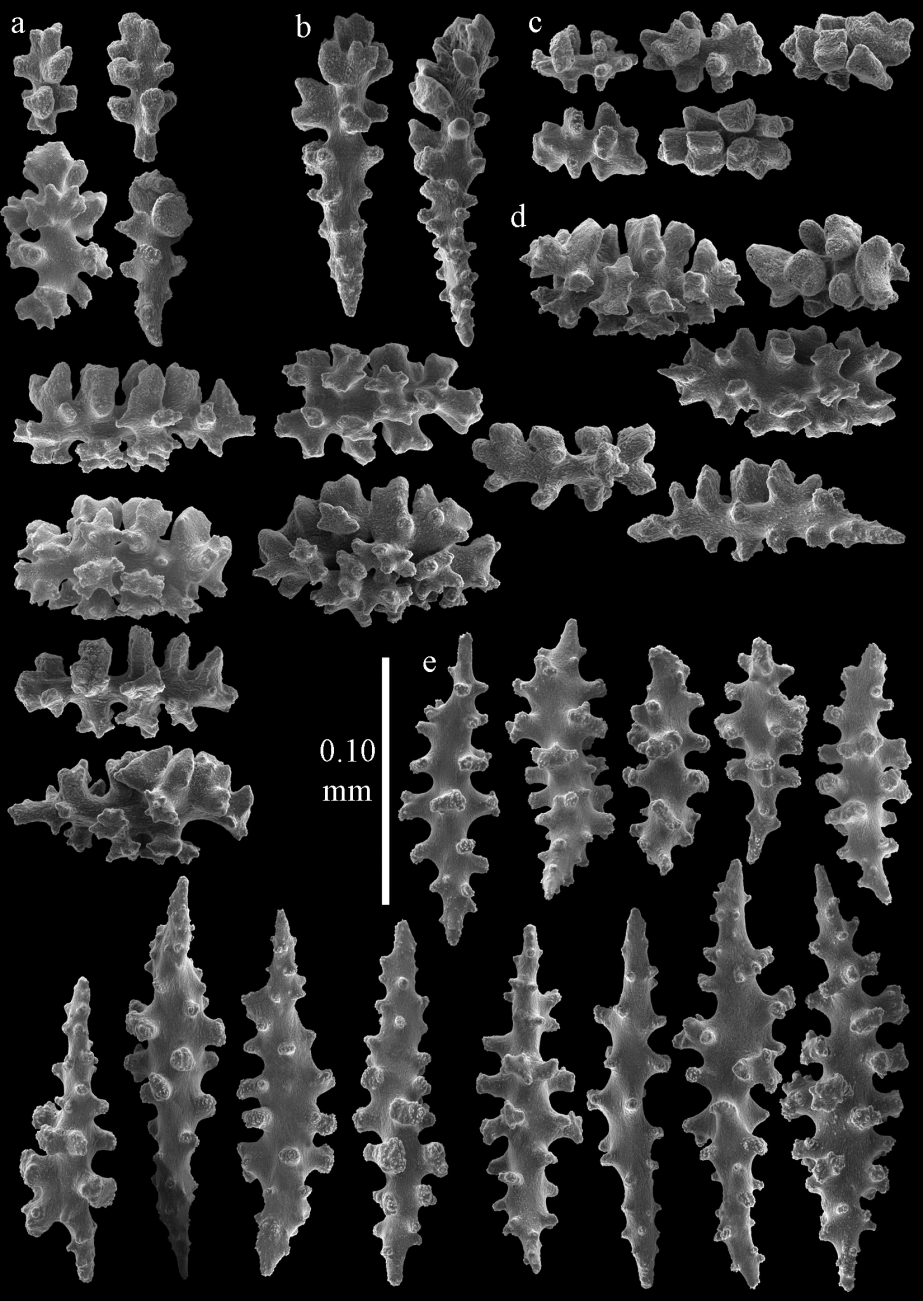
Sclerites of *Melithaea tenuis*, ZMB 5799; **a** clubs of coenenchyme **b** clubs of calyx **c** capstans **d** unilaterally foliate spheroids and spindles **e** spindles.

Figures 87b , 90 , 91 , 98

Acabaria tenuis : Kükenthal 1908: 195; Kükenthal 1909: 61, figs 64–67, pl. 5 fig. 27 (Sagami Bay, Okinose Bank); Kükenthal 1919: 183; Kükenthal 1924: 78; Aurivillius 1931: 24 (Sagami Bay, 400 fms); Hickson 1937: 177.
Acabaria sp. aff. tenuis : Aurivillius 1931: 25, fig. 3 (Sagami Bay, 200 m).
NotAcabaria tenuis : Nutting 1911: 45 (Indonesia).
? Acabaria tenuis : Rho et al. 1980: 53 (Korea Strait).

#### Material examined.

Syntype **ZMB 5799**, Sagami Bay (Japan), 600 m (label 60-250 m), coll. Doflein,1904/05; previously unidentified museum material: **BMNH 1921.10.26.9-2**, Misaki, Sagami Bay, 200 fms (286–366 m), coll. A.V. Insole, May 1921; **BMNH 1921.10.26.7**, same data as BMNH1921.10.26.9-2; **ZMUC ANT-000593**, Sagami Bay, 400 fms, coll. Dr. Th. Mortensen, 2 July 1914; **ZMUC ANT-000594**, Sagami Bay, Japan, 80–120 fms (114–219 m), coll. Dr. Th. Mortensen, 6–19 June 1914; **ZMUC ANT-000661**, Sagami Sea, 300 fms (429–549 m), coll. Dr. Th. Mortensen, 29 June 1914; **ZMUC ANT-000656**, off Misaki Biological Station, Sagami Bay, 200 fms (286–366 m), coll. Dr. Th. Mortensen, 30 June 1914; **ZMUC ANT-000651**, Off Misaki, Sagami Bay, ca. 250 fms (ca.358–457 m), coll. Dr. Th. Mortensen, 10 June 1914; **UMUTZ-CnidG-16**, near Doketsuba, Sagami Bay, 170-180 hiro (243–272 m), coll. Kuma Aoki, 12 August 1895; **UMUTZ-CnidG-198**, Mera-no-hai-dashi-Oise line, Sagami Bay, 350 fms (500–529 m), coll. H. Matsumoto and H. Chiba, 21 July 1913; **UMUTZ-CnidG-233**, Gokeba, Sagami Bay, 150-20 hiro (227–29 m), coll. Kuma Aoki, 18 June 1902; **AKM 142**, East China Sea, *R/V Tanisei-maru*, KT02-03, st.E-5-1, 1 m Dredge, 19 April 2002; **AKM 234**, South of Mera-se Bank, Sagami-bay, 34°60.0'N, 139°40.2'E – 35°0.0'N, 139°40.3'E, 97–108 m, 17 October 2003; **AKM 235**, same data as AKM 234; **AKM 413**, Watari-se bank, Off Izu Isls., 34°02.8620'N, 138°54.8090'E – 34°02.9190'N, 138°54.6810'E, 101.1-106.2 m, *R/V Tansei-maru*, KT04-06, st. WS-2, 1 m ORI dredge, coll. A.K. Matsumoto, 30 April 2004; **RMNH Coel. 41939 (AKM 414)**, same data; **AKM 415**, same data; **AKM 421**, same data; **AKM 422**, same data; **RMNH Coel. 41940 (AKM 521**), Otsuki, Tosa, Kochi prefecture, 132°50.44'E 32°37.66'N, – 132°47.88'E 32°37.56'N, 114 m, local fishermen’s boat *Kiryo-maru*, St. 1, coral net, coll. A.K. Matsumoto, 7 October 2004; **AKM 663**, off Tanabe, Wakayama Prefecture, 33°39.05'N, 135°09.89'E – 33°38.96'N, 135°10.16'E, 170.3-173.1 m, *R/V Tansei-maru*, KT05-30, st. TN-1 (1), coll. A.K. Matsumoto 26 November 2005; **RMNH Coel. 41942 (AKM 664)**,same data as AKM663; **AKM 670**, off Tanabe, Wakayama Prefecture, 33°39.02'N, 135°09.89'E – 33°39.03'N, 135°09.07'E, 169.8-172.5 m, *R/V Tansei-maru*, KT05-30, TN-1 (2), coll. A.K. Matsumoto, 26 November 2005; **AKM 745**, off Satamisaki Cape, Kagoshima Prefecture, 30°56.0025'N, 130°44.2299'E – 30°56.2953'N, 130°43.3981'E, 116-120 m, *R/V Tansei-maru*, KT07-1, st.SM-1, Chain Bag Dredge, coll. A.K. Matsumoto, 23 February 2007; **RMNH Coel. 41943 (AKM 980)**, Tsukura-se bank, Kagoshima Prefecture, East China Sea, 31°18.95'N, 129°46.15'E – 31°18.50'N, 129°45.96'E, 154–155 m, *R/V Tansei-maru*, KT08-3, St. NM05, ORI-TI Chain Bag Dredge, coll. A.K. Matsumoto, 6 March 2008; **AKM 983**, same data as RMNH Coel. 41943; **AKM 1222**, off Takarajima Is., Tokara Isls, East-China Sea, 29°05.29'N, 129°10.43'E, 334 m, *R/V Tansei-maru*, KT07-21, st. DT0203-1, Chain Bag Dredge, coll. Yokose, 31 August 2007; **AKM 1230**, same data as AKM1222; **AKM 1598**, Otsuki, Tosa, Kochi Prefecture, 32°37'N, 132°50'E, 114 m, local fishermen’s boat *Kiryo-maru*, st. 1, coll. A.K. Matsumoto, 7 October 2004; **AKM 1600**, Otsuki, Tosa, Kochi Prefecture, 32°43.08'N, 132°48.06'E – 32°43.12'N, 132°47.68'E, 84.75-83.1 m, local fishermen’s boat *Kiryo-maru*, st. 3, coll. A.K. Matsumoto, 7 October 2004.

#### Re-description.

Colony branched in one plane, with slender branches (Fig. 87b). Points with slightly bent spindles up to 0.15 mm long, distal end with more developed tubercles (Fig. 90a). Collaret with bent spindles up to 0.20 mm long, middle part with more developed tubercles (Fig. 90b). Tentacles with platelets, the larger ones crescent-shaped with irregular projections (Fig. 90c). These platelets are up to 0.15 mm long. Pharynx with straight spiny rods, up to 0.05 mm long (Fig. 90d). Coenenchyme with capstans (Fig. 91c), about 0.05 mm long, small clubs of the same length (Fig. 91a); unilaterally spinose spheroids and unilaterally spinose spindles up to 0.10 mm long (Fig. 91d); spindles are also present, 0.10–0.18 mm long (Fig. 91e). The calyces with additional clubs, up to 0.15 mm long (Figs 90e, 91b).

#### Color.

Colony red with white/yellow polyps, polyp sclerites colorless or yellow, all others orange.

#### Variation.

Colonies can be yellow with all sclerites yellow or white with all sclerites colorless.

#### Distribution.

Sagami Bay, and now South to East China Sea (Fig. 98).

#### Remarks.

We did not examine ZMB 5805, syntype, Okinose Bank (Japan), 80–250 m, coll. Doflein 1904/05 because the material consists of only small fragments.

### Melithaea tokaraensis sp. n.

Taxon classificationAnimaliaAlcyonaceaMelithaeidae

http://zoobank.org/C8884567-77B9-49B1-82DA-155636E44F72

**Figure 92.**
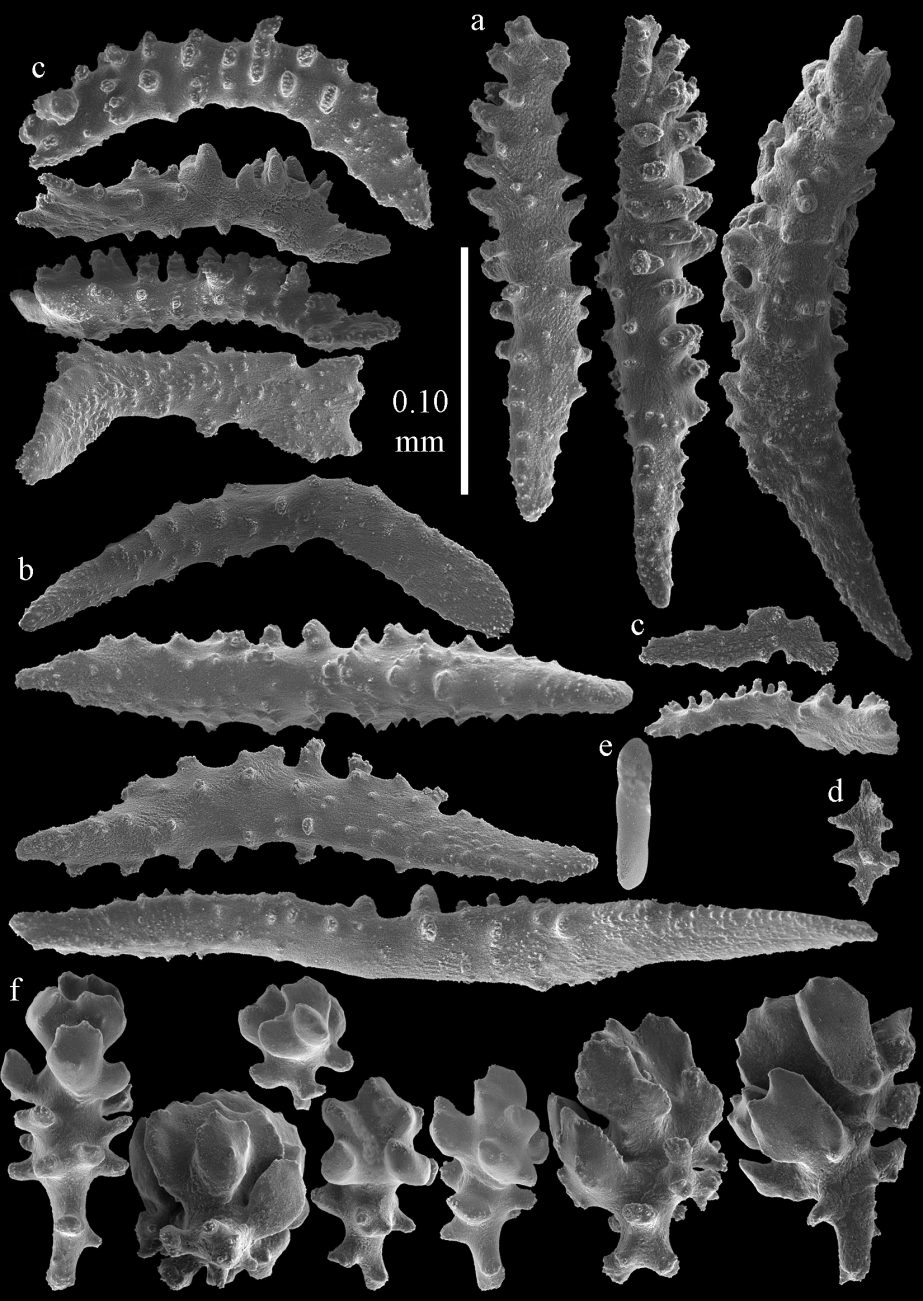
Sclerites of *Melithaea tokaraensis* sp. n., RMNH Coel. 41941; **a** point spindles **b** collaret spindles **c** tentacle sclerites **d** pharynx rod **e** axial rod **f** clubs of coenenchyme.

**Figure 93.**
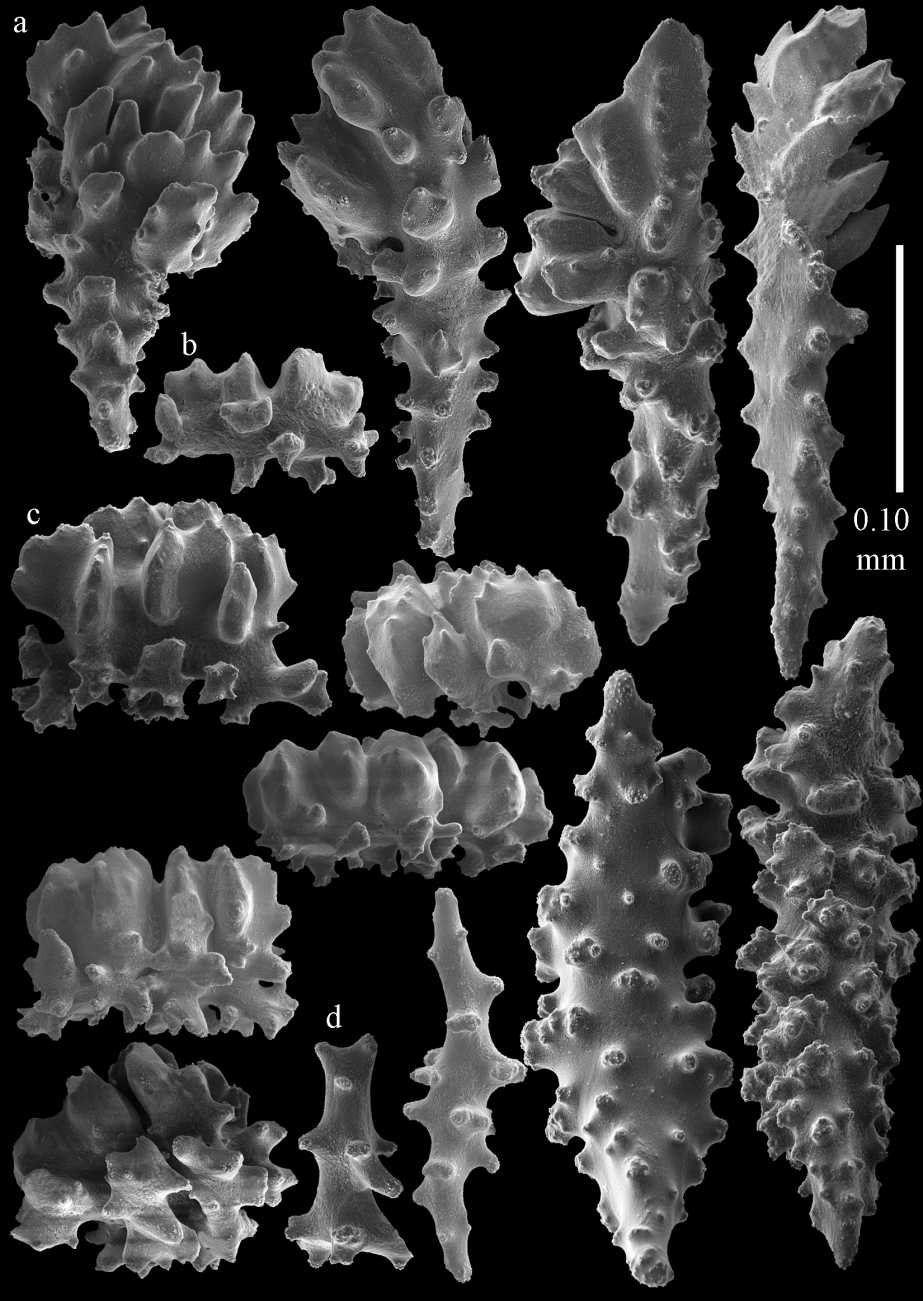
Sclerites of *Melithaea tokaraensis* sp. n., RMNH Coel. 41941; **a** clubs of calyx **b** double disk **c** disk spindles **d** spindles.

Figures 87c , 92 , 93 , 98

#### Material examined.

Holotype **RMNH Coel. 41941 (AKM 1212)**, 28°54.90'N, 129°04.09'E, off Yokoate-jima, Tokara Islands, East China Sea, 395 m, *R/V Tansei-maru*, KT07-21, St. DY0205, Chain Bag Dredge, coll. Dr. H. Yokose, 30 August 2007.

#### Description.

Colony branched in one plane, broken up while collecting (Fig. 87c). The largest fragment being 12 cm long. The larger nodes are swollen and clearly visible. The polyps are situated on one side of the colony. The calyces are dome-shaped, about 0.5 mm high and 1 mm wide. Points with slightly bent spindles up to 0.25 mm long, distal end more tuberculate (Fig. 92a). Collaret with bent spindles up to 0.35 mm long, middle part with more developed tubercles (Fig. 92b). Tentacles with platelets, the larger ones crescent-shaped with irregular projections (Fig. 92c). These platelets are up to 0.15 mm long. Pharynx with straight spiny rods, up to 0.05 mm long (Fig. 92d). Coenenchyme with predominantly capstans, double disks (Fig. 93b) and disk spindles (Fig. 93c), 0.05–0.15 mm long, and small clubs (Fig. 92f), up to 0.10 mm long. Spindles are also present, 0.10–0.30 mm long, mostly with simple tubercles (Fig. 93d). The calyces with additional clubs, up to 0.25 mm long (Fig. 93a). The axis has smooth and sparsely tuberculate rods (Fig. 92e).

#### Color.

Colony red, all sclerites orange.

#### Distribution.

Only known from off the Tokara Islands. (Fig. 98).

**Etymology.** The species is named after the type locality, the Tokara Islands.

#### Remarks.

The species resembles *Melithaea abyssicola* sp. n., *Melithaea oyeni* sp. n., and *Melithaea satsumaensis* sp. n., but differs in having rather smooth polyp sclerites and long calyx clubs.

### Melithaea undulata

Taxon classificationAnimaliaAlcyonaceaMelithaeidae

(Kükenthal, 1908)

**Figure 94.**
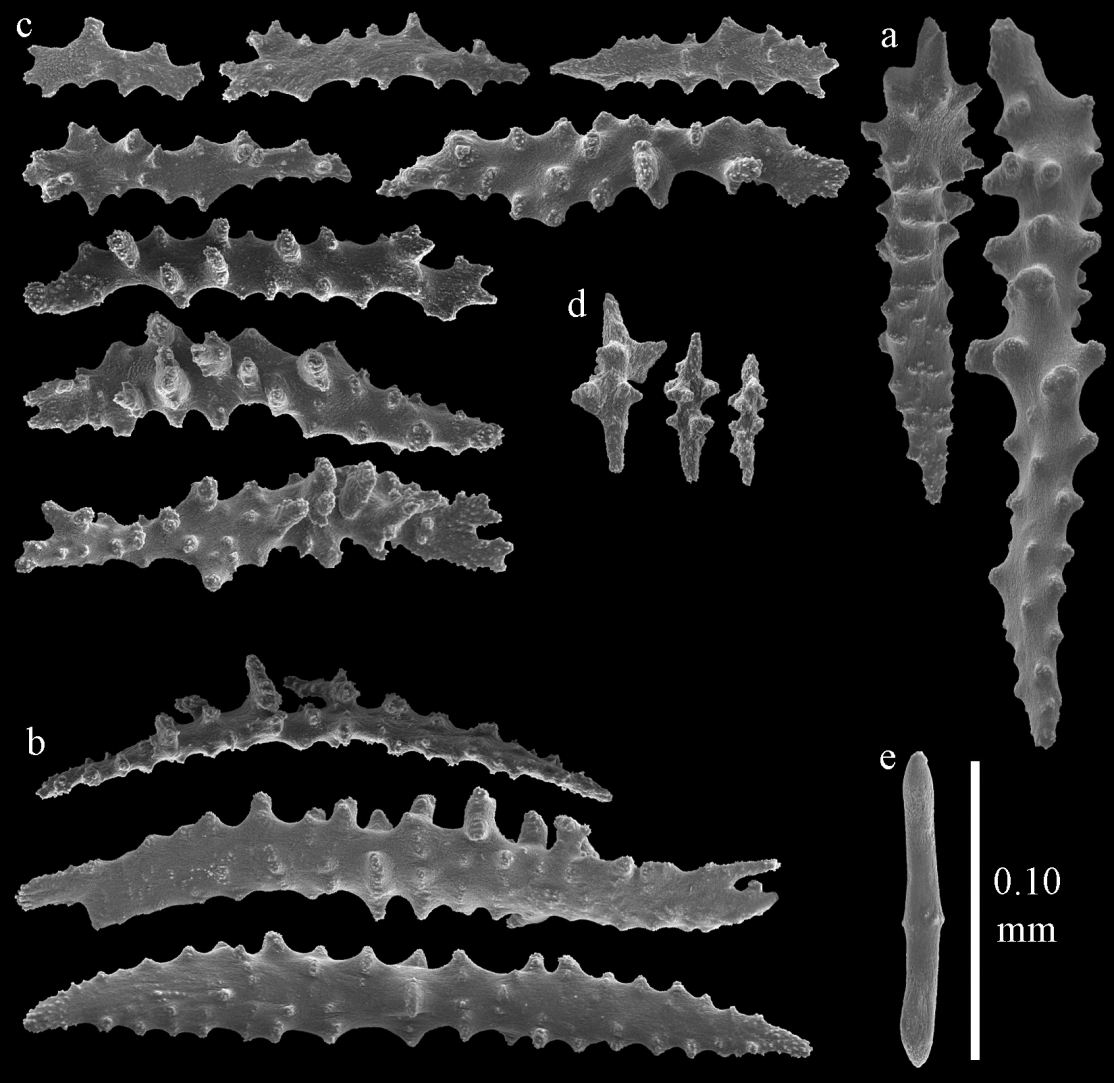
Sclerites of *Melithaea undulata*, ZMB 5803; **a** point spindles **b** collaret spindles **c** tentacle sclerites **d** pharynx rods **e** axial rod.

**Figure 95.**
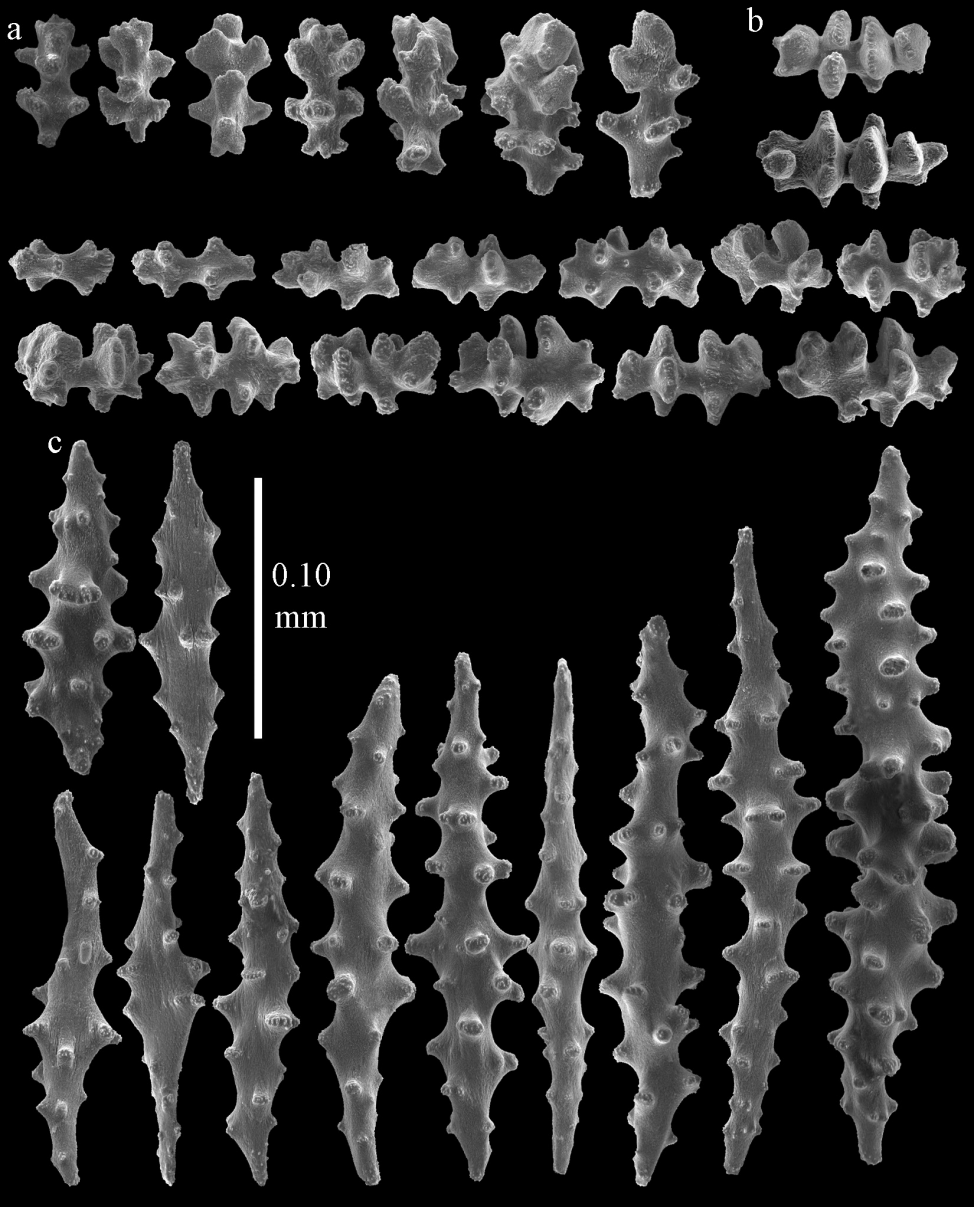
Coenenchymal sclerites of *Melithaea undulata*,ZMB 5803; **a** clubs **b** capstans **c** spindles.

**Figure 96.**
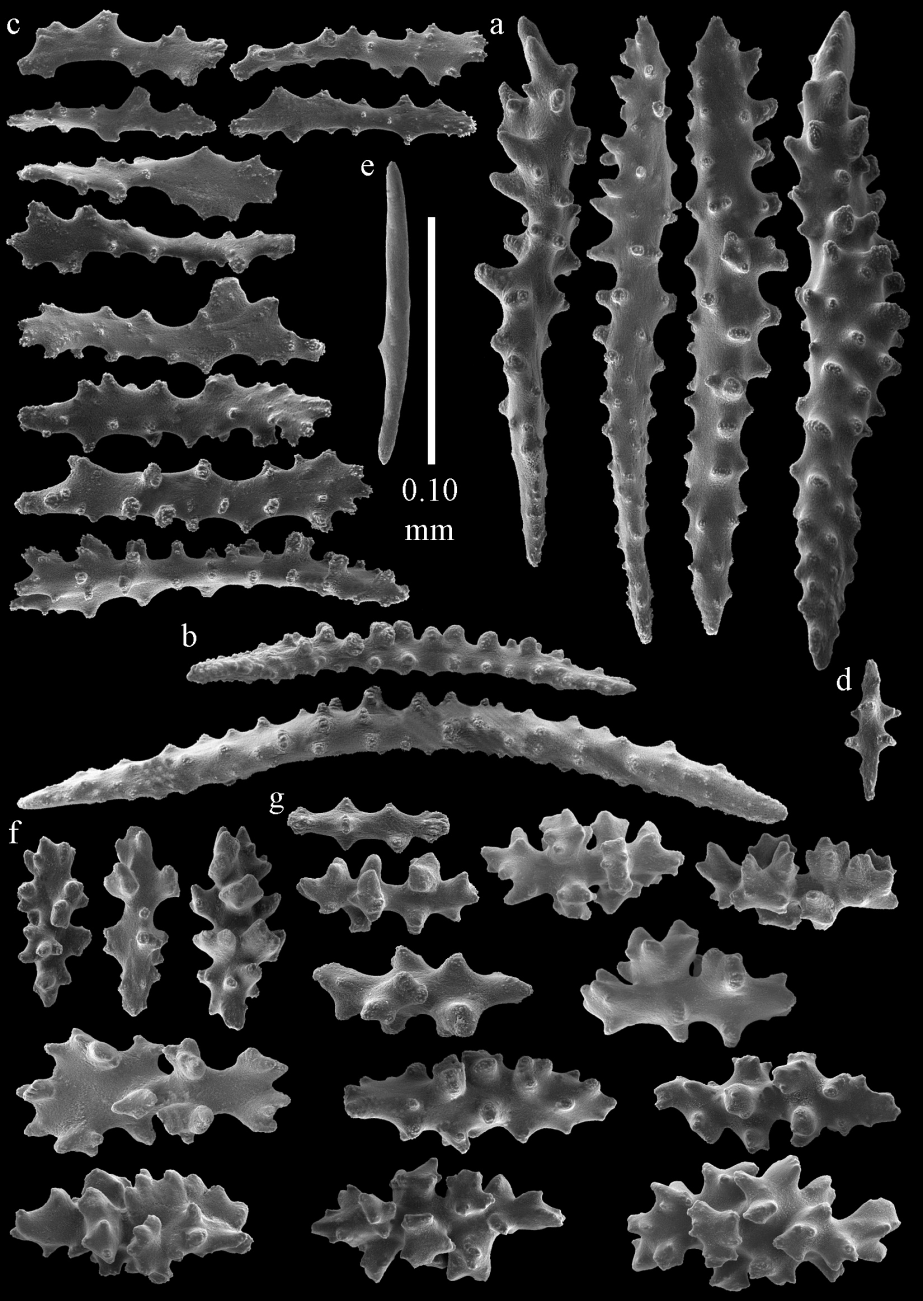
Sclerites of *Melithaea undulata*, RMNH Coel. 42030; **a** point spindles **b** collaret spindles **c** tentacle sclerites **d** pharynx rod **e** axial rod **f** clubs of coenenchyme **g** capstans of coenenchyme.

**Figure 97.**
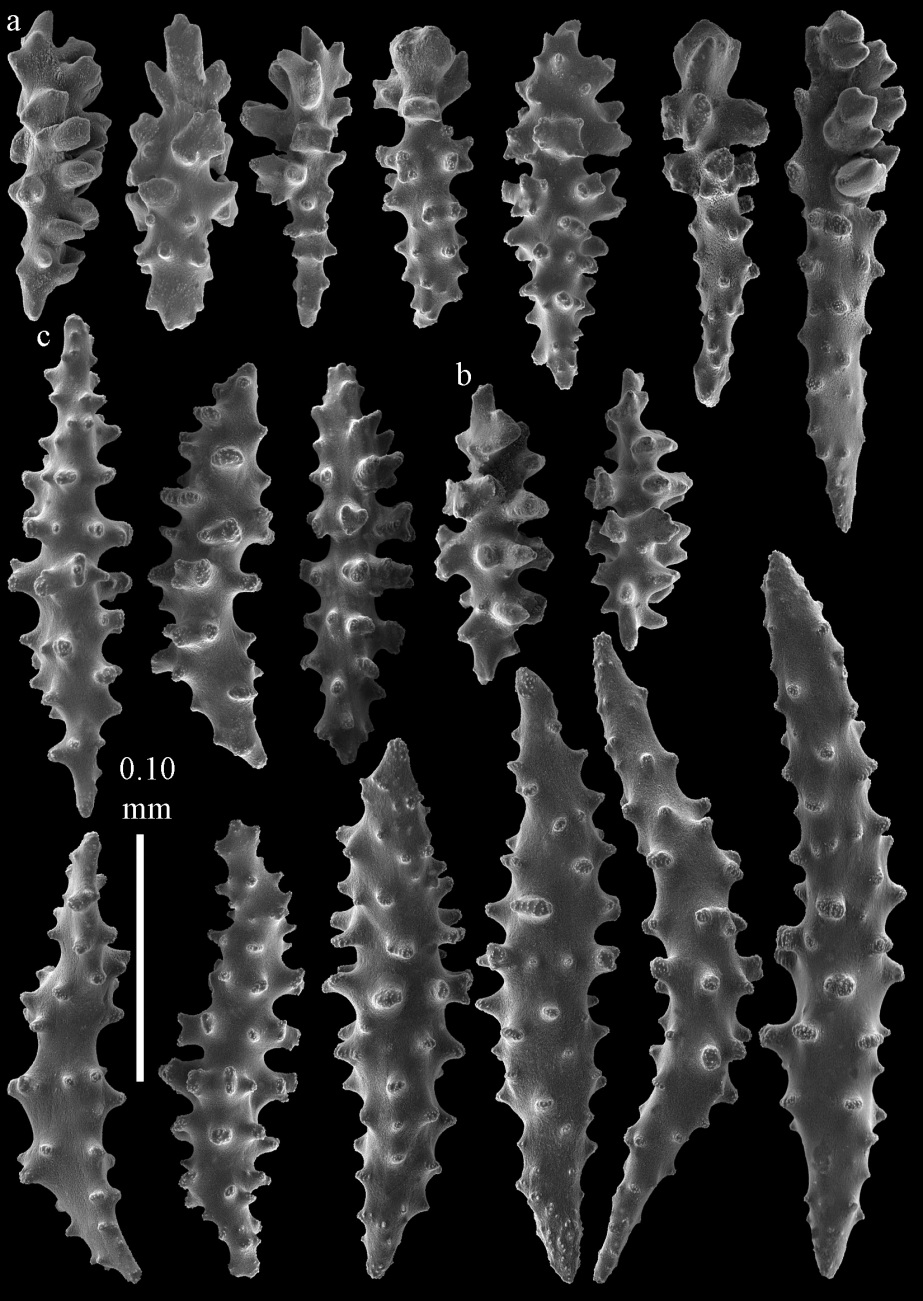
Sclerites of *Melithaea undulata*, RMNH Coel. 42030; **a** clubs of calyx **b** unilaterally spinose spindles **c** spindles.

**Figure 98.**
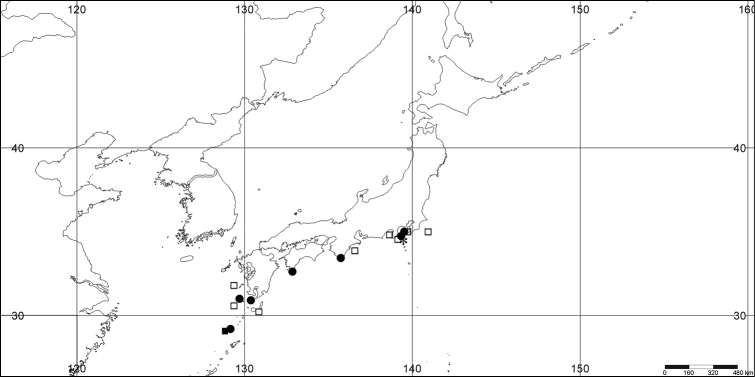
Distribution of *Melithaea tanseii* sp. n. (*), *Melithaea tenuis* (●), *Melithaea tokaraensis* sp. n. (■), and *Melithaea undulata* (□).

Figures 87d–e , 94 , 95 , 96 , 97 , 98

Acabaria undulata Kükenthal 1908: 196; 1909: 63, figs 68–69, pl. 5 fig. 28, pl. 7 figs 40–43 (Japan); 1919: 179; 1924: 76; Hickson 1937: 181.
? Acabaria undulata ; Rho et al. 1980: 54 (Korea Strait); Song 2000: 121 (Korea Strait).

#### Material examined.

Holotype **ZMB 5803**, Sagami Bay, Japan, 700 m, coll. Doflein, 1904/05; previously unidentified museum material: **ZMUC ANT-000592**, Sagami Sea, Japan, 500 fms (715-914 m), coll. Dr. Th. Mortensen, 25 June 1914; **BMNH 1921.10.26.9-1**, **BMNH 1921.10.26.9-2**, Misaki, Sagami Bay, 200 fms (286-366 m), coll. A.V. Insole, May 1921; **MZS-Cni57**, Sagami Bay, 200 m, coll. Doederlein**. UMUTZ-CnidG-14**, Niijima Is., Izu Isls., coll. sp. no. 80, 22 April; **UMUTZ-CnidG-20**, Dokesuba, Sagami Bay, 130 hiro (186–196 m), coll. Kuma Aoki, 9 August 1897; **UMUTZ-CnidG-234**, Mochiyama, Sagami Bay, 400 hiro (572–604 m), possibly coll. Kuma Aoki; **AKM 502**, Ose-zaki, Suruga Bay, 137–155 m, coll. K. Kitazawa, 22 July, 2004; **RMNH Coel. 42030 (AKM 724)**, off Tanega-shima Is., East China Sea, 30°24.62'N, 131°08.46'E – 30°24.95'N, 131°08.32'E, 468-502 m, *R/V Tansei-maru*, KT07-1, st. TN-3, 1 m biological dredge, coll. A.K. Matsumoto, 23 February 2007; **AKM 1034**, Koshiki Knoll, off Kagoshima, East China Sea, 31°36'N, 129°19'E, 497-535 m, *R/V Tansei-maru*, KT08-3, st. KS-01, coll. A.K. Matsumoto, 6 March, 2008; **AKM 1591**, Danzyo-Basin, East China Sea, 31°58.02'N, 129°02.28'E – 31°59.30'N, 129°01.19'E, 711-801 m, *R/V Tansei-maru*, KT00-17, St. DZ-1, ORE Beam Trawl of 3 m span, coll. Suguru Ohta, 12 December 2000; **AKM 1592**, same data as AKM1591; **AKM 1593**, South East off Taito-saki Cape, Boso Peninsula, 35°05.086'N, 140°51.718'E – 35°04.176'N, 140°50.92'E, 975-1027 m, *R/V Tansei-maru*, KT03-17, st. TS6-3, coll. S. Ohta, 17 November 2003; **AKM 1599**, South off Daio-zaki Cape, Kumano-nada, 34°05'N, 136°51'E, *R/V Tanisei-maru*, KT94-07, st. KN25, 25 May 1994, 475-494 m, coll. S. Ohta; **AKM 1604**, west off Izu-Oshima, Sagami Bay, 139°15.0'E 34°40.4'N, 415-440 m, *R/V Hakuho-maru*, KH-78-05, st. BS8, 2 m S.-A. beam trawl (on label), 7 December 1978.

#### Re-description.

Colony branched in two parallel planes. Points with slightly bent spindles up to 0.25 mm long, distal end with more developed tubercles (Fig. 94a). Collaret with bent spindles up to 0.30 mm long, middle part with more developed tubercles (Fig. 94b). Tentacles with platelets, the larger ones crescent-shaped (Fig. 94c). These platelets are up to 0.15 mm long. Pharynx with straight spiny rods, up to 0.05 mm long (Fig. 94d). Coenenchyme with capstans (Fig. 95b) about 0.05 mm long, several slightly unilaterally spinose, small clubs of the same length (Fig. 95a); spindles 0.10–0.30 mm long (Fig. 95c); all with simple tubercles. The calyces with additional clubs, up to 0.12 mm long (hardly present in type material). The axis has smooth and sparsely tuberculate rods (Fig. 94e).

#### Color.

Colony red, tentacle sclerites colorless, all others pink.

#### Variation.

AKM 502 is yellow with yellow sclerites.

#### Distribution.

Southern Pacific coast of Japan, north to Sagami Bay, south to East China Sea; Suruga Bay, off Taito-Saki Cape, Boso Peninsula; off Tanega-shima Is., Koshiki Knoll., Danzyo Basin, off Kagoshima (Fig. 98).

#### Remarks.

The type specimen clearly has damaged sclerites, probably caused by formalin. Recently collected material shows less rounded sclerites (Figs 87e, 96, 97). Double disks are present but so poorly developed that they can hardly be recognized as such.

The species resembles *Melithaea tenuis* but differs in having longer spindles (up to 0.30 mm long versus up to 0.18 mm long in *Melithaea tenuis*), and lacking unilaterally spinose spheroids. It also resembles *Melithaea corymbosa*, but that species has mostly more slender, shorter spindles. Moreover, *Melithaea undulata* has poorly developed tentacle sclerites compared with the other two species.

Apparently the type material is mostly lost, only a small fragment remained (Fig. 87d) of a colony described as being 21 cm long (Kükenthal 1909).

### Unidentified material (disintegrated sclerites)

**BMNH1921.10.26.24-1**, Misaki, Sagami Bay, 500–600 fms, coll. A.V. Insole, No. 45; **ZMUC ANT-000648**, Sagami Bay, Okinose, 60 fms (86–110 m), 11 June 1914, coll. Dr. Th. Mortensen, hard bottom, Gear: swabs; **ZMUC ANT-000589**, Okinose, Sagami Sea, 60 fms (86–110 m), 11 June 1914, coll. Dr. Th. Mortensen; **ZMUC ANT-000652**, 34°20'N, 130°10'E, 60 fms (86–110 m), 18 May 1914, coll. Dr. Th. Mortensen; **UMUTZ-CnidG-13**, Doketsuba, Sagami Bay, Japan, 60 fms (86–91 m), coll. K. Kinoshita, October 1908; **UMUTZ-CnidG-18**, Kashiwajima Is., Tosa, Kochi Prefecture, Japan, coll. K. Kinoshita, June 1909; **UMUTZ-CnidG-26**, Shikine Is., Izu Isls., Japan; **UMUTZ-CnidG-39**, Kashiwajima Is., Tosa, Kochi Prefecture, Japan, June 1909 (possibly collected by K. Kinoshita as same as UMUTZ-CnidG-18); **UMUTZ-CnidG-40**, Cape Matsu-zaki, east of Izu Peninsula, Sagami Bay, coll. M. Miyajima, 29 August 1897; **UMUTZ-CnidG-270 (G-40b)**, same data as UMUTZ-CnidG-40; **UMUTZ-CnidG-191**, same data as UMUTZ-CnidG-18; **UMUTZ-CnidG-206**, Ogasawara Isls., coll. S. Hirota and Sekiguchi, 11 April 1894.

## Discussion

We compared Japanese melithaeids with those described from other parts of the Indo-Pacific (Hickson 1937, Ofwegen 1987, Ofwegen et al. 2000, Reijnen et al. 2014) and never found a match based on examination of sclerites. Therefore we conclude they are all endemic to Japan.

The only Japanese melithaeid species described by Kükenthal (1908) which we could not check was *Melithaea habereri*. The material of that species seems to be lost. As well we could not find the two varieties described by Kükenthal Melithaea flabellifera var. reticulata Kükenthal, 1908, and Melithaea flabellifera var. cylindrata Kükenthal, 1908. The type material of *Pleurocorallium confusum* Moroff, 1902, was not found but from the original description, i.e. colony with flattened branches, it was obvious this species could be synonymized with *Melithaea japonica*. The material used to describe *Melithaea mutsu* Minobe, 1929 has also been lost but for this species we designated a neotype (Figs 56c, 63–65).

Most of the already described melithaeid species could be identified again from newly collected material. However, specimens of *Melithaea arborea* Kükenthal, 1908 (Figs 8, 9a, 10, 11), *Melithaea frondosa* (Brundin, 1896) (Figs 28b, 31, 32, 35), and *Melithaea nodosa* Wright and Studer, 1889 (Figs 66abc, 67–69, 74) could not be retrieved. We do not exclude the possibility that *Melithaea arborea* is nothing else than *Melithaea japonica* but somehow with its clubs lost. It is puzzling why we could not find *Melithaea frondosa* and *Melithaea nodosa*, as we did examine material from the regions from which these species were described, Hirado Strait (Fig. 35) and Sagami Bay (Fig. 74), respectively.

We have tentatively included BMNH 1921.10.26.24-2 in *Melithaea abyssicola*. It had sclerite damage caused by formalin (Figs 6, 7).

We included a number of problematic specimens in *Melithaea corymbosa* that showed differences. Because of the limited material and rather small differences we refrain from describing these specimens as new species: RMNH Coel. 41903(Figs 14c, 21, 22); RMNH Coel. 41908 (Figs 14d, 23, 24) and RMNH Coel. 41909; RMNH Coel. 41910 and RMNH Coel. 41911 (Figs 14e, 25, 26); and ZMUC ANT-000646. They all were collected from South Japan (Fig. 27).

We included BMNH 62.7.16.62(61?) in *Melithaea sagamiensis* sp. n. despite its slightly different sclerites (Figs 80, 81). It was collected in quite shallow water, 25 feet (= 7.62 m) -30 feet (= 9.14 m) from off Okushiri, Sea of Japan (Fig. 86). This specimen is the northernmost melithaeid coral ever found.

*Melithaea japonica*, *Melithaea corymbosa* and *Melithaea sagamiensis* sp. n. were collected together. These species could not be separated on colony form but only based on their sclerites. *Melithaea japonica* mostly occurs in shallow water and is the only melithaeid species of which the spawning season and growth rate is known because of their accessibility for long-term study (formerly *Melithaea flabellifera* in Matsumoto 2004).

Reijnen et al. (2014) used eight Japanese specimens in their molecular study. At that time only RMNH Coel. 41942 was identified, as *Melithaea tenuis*. Here the other seven specimens are also identified, or described as new species, and their affinities are examined. *Melithaea keramaensis* sp. n. appears to be genetically identical to specimens from Indonesia, Vietnam, and Malaysia (Reijnen et al. 2014: Fig. 2). Of these it morphologically mostly resembles RMNH.Coel.41142 from Malaysia but clearly differs from that species in having disk spindles. *Melithaea satsumaensis* sp. n. is the only other species genetically somewhat different from the other Japanese specimens, which is supported by its sclerites looking like those belonging to the now abandoned genus *Mopsella*, while the other Japanese specimens in the tree could be abandoned genus *Acabaria* or valid genus *Melithaea*. The remaining six specimens did not differ genetically from each other while we identified them as three different species, *Melithaea corymbosa*, *Melithaea japonica*, and *Melithaea tenuis*. This result could be explained by imagining deep water *Melithaea corymbosa* and *Melithaea tenuis* show different sclerites and colony shape from shallow water *Melithaea japonica*, and the differences noted are merely reflecting ecophenotypic variation instead of interspecific variation. Ofwegen (1987: 20) and Ofwegen et al. (2000: 291) already reported species including specimens with different sclerites. The main sclerite difference between *Melithaea corymbosa* and *Melithaea tenuis* is in the presence of capstans and derivatives of those, many present in *Melithaea tenuis* and only few in *Melithaea corymbosa*. We can imagine this also reflecting variability and these two species actually being one and the same. If this is the case *Melithaea japonica*, *Melithaea tenuis* and *Melithaea corymbosa* all could represent one and the same species. However, many specimens of these three species are not included in the molecular study and these complicate the above possibility of variability. The *Melithaea corymbosa* specimen used in not typical of that species (see Remarks *Melithaea corymbosa*), most specimens come from Sagami Bay and have slightly different sclerites. The *Melithaea japonica* specimens used for the molecular work all came from the Pacific of northern Japan, with the deepest occurrence, while this species is most common in Sagami Bay, with slightly different sclerites than the northern specimens (see Remarks *Melithaea japonica*). With two species of the three used in the molecular study presented by rather atypical specimens we decide to keep all three species separate till more study has been done.

Sagami Bay and adjacent waters produced four new species: *Melithaea doederleini* sp. n. (60–100 fms (108–183 m); Figs 28a, 29, 30, 35), *Melithaea sagamiensis* sp. n. (286–440 m; Figs 75a, 75b, 76–81, 86), *Melithaea oyeni* sp. n. (101.1–127 m; Figs 66d, 70, 71, 74) and *Melithaea tanseii* sp. n. (143–198 m; Figs 87a, 88, 89, 98) bringing the total number of *Melithaea* species from Sagami Bay to 13, making it the richest melithaeid region of Japan. All four were collected from deep water (101.1–440 m). The depth between 100–200 m is known as having the highest octocoral species diversity in Japan and adjacent waters (Matsumoto et al. 2007). In total five new species are here described from this depth region.

Northern Japan shows the melithaeids to have a more shallow distribution: *Melithaea sagamiensis* sp. n. shows a shallower distribution here than in Sagami Bay, 25–30 feet (7.62–9.14 m) (Fig. 86); four species (*Melithaea japonica*, *Melithaea corymbosa*, *Melithaea mutsu*, *Melithaea sagamiensis* sp. n.) are distributed in the northern region of Japan with maximum depth ca.100 m; three specimens of *Melithaea japonica* from the northern region have extreme slender sclerites but are still identified as *Melithaea japonica* (Figs 53–55); *Melithaea mutsu* collected from the area between the Sea of Japan and the Pacific at 5 m depth (Fig. 65). No deep water Melithaeidae species were found in the Sea of Japan.

Other new species were collected from the Ogasawara Isls. (= Bonin Islands) (*Melithaea boninensis* sp. n.; Fig. 8)).The Bonin Islands are isolated from main Japan by currents such as warm Kuroshio Current. We also described five new species from the East China Sea: *Melithaea isonoi* sp. n. (found on coral reef; Fig. 35), *Melithaea keramaensis* sp. n. (51–85 m; Fig. 65), *Melithaea ryukyuensis* sp. n. (shallow diving depth; Fig. 74), *Melithaea suensoni* sp. n. (311 m; Fig. 86) and *Melithaea tokaraensis* sp. n. (395 m; Fig. 98), and one new species from Osumi Peninsula of southernmost Kyushu Is.: *Melithaea satsumaensis* sp. n. (116–120 m; Fig. 86).

Only two melithaeids are known from the Sea of Japan (*Melithaea japonica*, *Melithaea sagamiensis* sp. n.), much less than the melithaeid species number of the Pacific side of Japan (14 species: *Melithaea abyssicola*, *Melithaea arborea*, *Melithaea boninensis* sp. n., *Melithaea corymbosa*, *Melithaea doederleini* sp. n., *Melithaea japonica*, *Melithaea modesta*, *Melithaea nodosa*, *Melithaea oyeni* sp. n., *Melithaea sagamiensis* sp. n., *Melithaea satsumaensis* sp. n., *Melithaea tanseii* sp. n., *Melithaea tenuis*, and *Melithaea undulata*) or of the East China Sea (11 species: *Melithaea frondosa*, *Melithaea isonoi* sp. n., *Melithaea japonica*, *Melithaea keramaensis* sp. n., *Melithaea modesta*, *Melithaea ryukyuensis* sp. n., *Melithaea satsumaensis* sp. n., *Melithaea suensoni* sp. n., *Melithaea tenuis*, *Melithaea tokaraensis* sp. n., and *Melithaea undulata*). *Melithaea satsumaensis* sp. n. was only found once but was counted twice, in the Pacific species and the East China Sea species because it was collected from off Sata-misaki cape at Kagoshima prefecture (Fig. 86) located between the Pacific Ocean and the East China Sea.

Nutting (1912) reported *Mopsella dichotoma* (Linnaeus, 1758) from Cape Tsiuka (unknown Japanese locality name; 41°35.50'N, 140°36.45'E, Tsugaru Strait between the Sea of Japan and the Pacific Ocean), but this is likely to be a misidentification because the type locality of *Melithaea dichotoma* is South Africa. A previous study did not report the existence of melithaeid corals in the Sea of Japan (Dautova 2007). Song (2000) and Rho et al. (1980) reported melithaeid corals previously described from Japan in Korean waters, the Sea of Japan and the East China Sea. We regard them as doubtful identifications as their descriptions lack detail for comparison.

Depth limitation in the Sea of Japan and the low species richness here, can probably be explained by the geographic history of Sea of Japan. The Sea of Japan is a marginal sea. It originated 15 million years ago during the last glacial maximum (LGM). It was almost totally closed off by a sea-level drop of ca.130 m. Approximately 12.000 years BP, the warm Tsushima Current, a branch of the warm Kuroshio current, started to flow into the Sea of Japan from the South (Oba et al. 1991, Ishiwatari et al. 2001, Yokoyama et al. 2000). It was a geographical barrier for the distribution of the marine organisms with a planktonic larvae stage, such as corals. Considering the highest species diversity depth range at Sagami Bay (100–200 m) the age of the Sea of Japan and the LGM barrier between 0–130 m could have restricted the warm Indo-Pacific species from the South to enter the Sea of Japan.

We synonymized four species and described 11 new species from Japanese waters. In total Japan now has 23 Melithaeidae species (including *Melithaea habereri*), and two varieties.

## Supplementary Material

XML Treatment for Melithaea abyssicola

XML Treatment for Melithaea arborea

XML Treatment for Melithaea boninensis

XML Treatment for Melithaea corymbosa

XML Treatment for Melithaea doederleini

XML Treatment for Melithaea frondosa

XML Treatment for Melithaea habereri

XML Treatment for Melithaea isonoi

XML Treatment for Melithaea japonica

XML Treatment for Melithaea keramaensis

XML Treatment for Melithaea modesta

XML Treatment for Melithaea mutsu

XML Treatment for Melithaea nodosa

XML Treatment for Melithaea oyeni

XML Treatment for Melithaea ryukyuensis

XML Treatment for Melithaea sagamiensis

XML Treatment for Melithaea satsumaensis

XML Treatment for Melithaea suensoni

XML Treatment for Melithaea tanseii

XML Treatment for Melithaea tenuis

XML Treatment for Melithaea tokaraensis

XML Treatment for Melithaea undulata
